# Concordant Gene Expression and Alternative Splicing Regulation under Abiotic Stresses in Arabidopsis

**DOI:** 10.3390/genes15060675

**Published:** 2024-05-23

**Authors:** Aala A. Abulfaraj, Sahar A. Alshareef

**Affiliations:** 1 Biological Sciences Department, College of Science & Arts, King Abdulaziz University, Rabigh 21911, Saudi Arabia; 2 Department of Biology, College of Science and Arts at Khulis, University of Jeddah, Jeddah 21921, Saudi Arabia; salshareef2@uj.edu.sa

**Keywords:** pre-mRNA, DAS, SF, splicing type, novel isoform, domain, motif, heat stress, intensive light stress, SR gene family

## Abstract

The current investigation endeavors to identify differentially expressed alternatively spliced (DAS) genes that exhibit concordant expression with splicing factors (SFs) under diverse multifactorial abiotic stress combinations in Arabidopsis seedlings. SFs serve as the post-transcriptional mechanism governing the spatiotemporal dynamics of gene expression. The different stresses encompass variations in salt concentration, heat, intensive light, and their combinations. Clusters demonstrating consistent expression profiles were surveyed to pinpoint DAS/SF gene pairs exhibiting concordant expression. Through rigorous selection criteria, which incorporate alignment with documented gene functionalities and expression patterns observed in this study, four members of the serine/arginine-rich (SR) gene family were delineated as SFs concordantly expressed with six DAS genes. These regulated SF genes encompass *cactin*, *SR1*-like, *SR30*, and *SC35*-like. The identified concordantly expressed DAS genes encode diverse proteins such as the 26.5 kDa heat shock protein, chaperone protein DnaJ, potassium channel GORK, calcium-binding EF hand family protein, DEAD-box RNA helicase, and 1-aminocyclopropane-1-carboxylate synthase 6. Among the concordantly expressed DAS/SF gene pairs, *SR30*/*DEAD*-box RNA helicase, and *SC35*-like/*1-aminocyclopropane-1-carboxylate synthase 6* emerge as promising candidates, necessitating further examinations to ascertain whether these SFs orchestrate splicing of the respective DAS genes. This study contributes to a deeper comprehension of the varied responses of the splicing machinery to abiotic stresses. Leveraging these DAS/SF associations shows promise for elucidating avenues for augmenting breeding programs aimed at fortifying cultivated plants against heat and intensive light stresses.

## 1. Introduction

RNA splicing is a pivotal post-transcriptional phenomenon that orchestrates the maturation of precursor messenger RNA (pre-mRNA) transcripts into mature messenger RNA (mRNA) by excising intervening sequences, termed introns [1]. This process predominantly unfolds within pre-mRNA molecules through a series of reactions mediated by the spliceosome—a multiprotein complex composed of five small nuclear ribonucleoproteins (snRNPs) [2]. Types of spliceosomes can be major or minor, differing in the structure of snRNPs, where they are composed of U1, U2, U4, U5, and U6 in the first type, while, respectively, composed of U11, U12, U4atac, and U6atac in the second [3]. Essential for splicing are three intron recognition sites: the 5′ donor site, the branch site proximal to the 3′ terminus, and the 3′ acceptor site [4,5]. These sites are delineated by consensus sequences, including G-G-[cut]-G-U-R-A-G-U…intron, intron…Y-U-R-A-C…intron (situated 20–50 nucleotides upstream of the acceptor site), and intron…Y^rich^-N-C-A-G-[cut]-G [6,7,8]. In some rare events, certain pre-mRNA introns undergo self-splicing, obviating the necessity for spliceosomal involvement and leading to the classification of these RNA molecules as ribozymes [9].

Alternative splicing (AS) dynamically responds to developmental cues based on spatiotemporal requirements, including tissue specificity and environmental stimuli [10]. AS engenders multiple isoforms from a single multiexonic gene, thereby augmenting proteome diversity as an evolutionary mechanism. Major modes of AS encompass intron retention, exon skipping, an alternate 5′ donor site, and an alternate 3′ acceptor site [11]. Functionally, AS modulates protein or protein domain sequences [12], facilitates the emergence of novel protein–protein interactions [13], influences mRNA turnover including RNA stability and decay [14], and impacts translational processes [15]. Consequently, generated isoforms often exhibit distinct functions, occasionally displaying opposing functionalities [16,17,18].

The equilibrium between expression levels and functionalities of diverse gene isoforms is finely tuned [19,20], with variations across tissues, developmental stages, and environmental conditions [21,22]. Empirically, discerning the function of novel isoforms proves challenging, given that many of them exhibit distinctions as subtle as a single amino acid alteration [17,18]. Structural approaches, primarily leveraging protein structure prediction methods based on amino acid sequences and conserved protein domains, offer insights into isoform functionalities [17,23,24]. Notably, the majority of isoforms of a given gene retain common active domains [17], thereby identifying isoforms harboring distinct or absent active domains as likely artifacts.

The nascent methodologies within high-throughput mRNA sequencing and computational biology facilitate the detection and quantification of the prevalence of alternative splicing isoforms [25]. These advancements enable the prediction of functions attributed to the newly generated differentially expressed alternatively spliced (DAS) gene isoforms, predicated upon their expression profiles within specific stress conditions. Integration of transcriptomic datasets stemming from disparate environmental conditions, facilitated by suitable bioinformatics tools, shows promise in discerning the authenticity of novel isoforms versus artifacts. Moreover, employing cluster analysis on RNA-Seq datasets may unveil regulatory elements influencing the architectures of both previously annotated and novel isoforms, contingent upon their synchronized expression across diverse environmental conditions [16,17,18].

In this study, we utilized clean RNA-Seq datasets retrieved from National Center for Biotechnology Information (NCBI), derived from stress experiments conducted on *Arabidopsis thaliana* seedlings subjected to various stress combinations, including salt, intensive light, and heat stresses [26]. Our investigation aimed to elucidate DAS genes under diverse environmental stresses and identify splicing factors (SFs) exhibiting concordant expression with isoforms of these DAS genes, with the intention of subsequent experimental validation of their inter-relationship. We expect this investigation to enhance our understanding of the diverse reactions exhibited by the splicing apparatus under abiotic stresses. Harnessing the associations between DAS and SF offers potential pathways for elucidating strategies to enhance breeding programs targeted at fortifying cultivated plants against abiotic stresses.

## 2. Materials and Methods

RNA sequencing datasets were acquired from the publicly available repository of BioProject PRJNA622644 within the NCBI, detailing a recent multifactorial stress experiment [26]. Accession numbers corresponding to distinct samples and replicates under varying abiotic stress conditions are itemized in Table 1.

High-throughput RNA sequencing reads were retrieved and subsequently aligned to the *A. thaliana* reference genome (TAIR10) utilizing HISAt2 software (version 2.2.1), following established protocols [27]. This alignment process facilitates the precise mapping of reads to known genomic loci. Subsequent analysis of the mapped reads was conducted using StringTie software (version 1.3.3b), enabling the identification of transcripts potentially absent in the existing gene annotation repository of the NCBI. These newly identified transcripts, denoted as “new isoforms”, were compared with previously annotated isoforms within the same loci using GffCompare software (v0.11.2) to discern novel alternative splicing events. Then, transcript sequences corresponding to both previously annotated and newly discovered isoforms were extracted from the *A. thaliana* TAIR10 genome using a Perl script, agat_sp_extract_sequences.pl. The merged sequences were compiled into a unified FASTA file, and transcript abundance for both isoform types was quantified utilizing RSEM software (v1.1.17) based on the RNA sequencing reads shown in Table 1. Differential expression analysis was executed employing EdgeR (R version 2.1.5) employing stringent criteria, including a fold change of ≥4 (log2(fpkm^+1^)) and a false discovery rate (FDR) of ≤10^−3^ [28], to identify transcripts exhibiting significant alterations in expression across diverse stress conditions. The fold change of ≥4 is typically not log2-transformed. Subsequently, differential gene expression profiles underwent Blastx analysis, with the establishment of significant Pearson correlations corroborated through permutation analysis. Following differential expression profiling, a focused inquiry targeted differentially expressed alternatively spliced (DAS) genes exhibiting consistent expression patterns and harboring multiple regulated isoforms, aiming to discern potential concordant expression with splicing factors (SFs). Clusters featuring DAS/SF gene pairs meeting the aforementioned criteria were subjected to rigorous quality control assessments, with candidate DAS/SF pairs surveyed for conceivable relationships.

## 3. Results

### 3.1. Hierarchical Clustering Analysis

**Figure 1:**
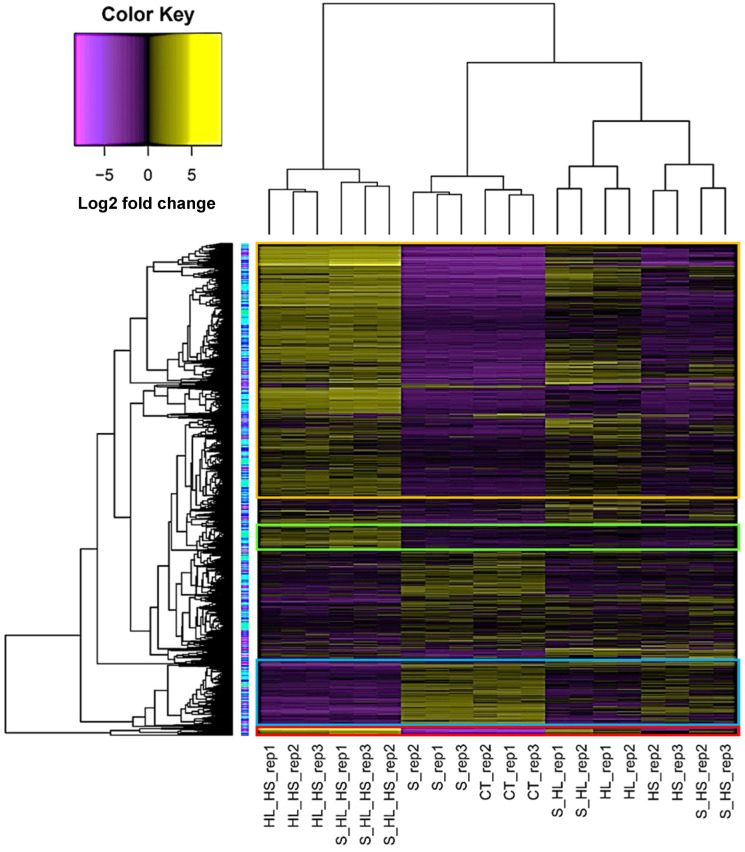
Heatmap referring to hierarchical clusters of gene expression generated from transcriptome datasets of 10-day-old *A. thaliana* (wild-type Col-0) seedlings exposed to different multifactorial stress combinations. CT = control (0 mM NaCl, 21 °C, 50 µmol m^−2^ s^−1^), S = salt stress (50 mM NaCl, 21 °C, 50 µmol m^−2^ s^−1^), H = heat stress (0 mM NaCl, 33 °C, 50 µmol m^−2^ s^−1^), L = intensive light stress (0 mM NaCl, 21 °C, 700 µmol m^−2^ s^−1^). Further information is available in Table S1. Further growth and abiotic stress conditions were recently reported [26]. The red box refers to the expression pattern H/L/HL↑, the bright blue box refers to the expression pattern C/S↑, the bright green box refers to HL↑, and the orange box refers to L↑. The log2 fold change was computed based on the delta Ct value in comparison to the control samples, where the yellow color in the legend indicates heightened expression, whereas the blue color signifies diminished expression. ↑ = upregulation.

**Figure 2:**
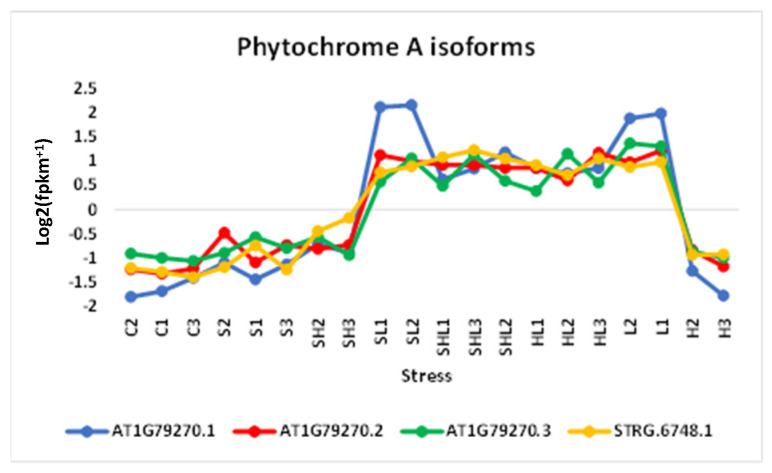
Expression profiles of the light-responsive *phytochrome A* gene located at locus XLOC_003272, along with its diverse isoforms derived from transcriptomic datasets obtained from 10-day-old *A. thaliana* (wild-type Col-0) seedlings subjected to various combinations of multifaceted stress stimuli. C = control (0 mM NaCl, 21 °C, 50 µmol m^−2^ s^−1^), S = salt stress (50 mM NaCl, 21 °C, 50 µmol m^−2^ s^−1^), H = heat stress (0 mM NaCl, 33 °C, 50 µmol m^−2^ s^−1^), L = intensive light stress (0 mM NaCl, 21 °C, 700 µmol m^−2^ s^−1^). Further information is available in Table S1.

In this investigation, 1D and 2D hierarchical clustering heatmaps were constructed to delineate the transcriptomic responses of Arabidopsis subjected to diverse stress conditions (Figure 1 and Figure S1, respectively). The 1D heatmap served to discern prevailing expression patterns within these transcriptomic datasets, while the 2D heatmap facilitated the identification of closely associated stress combinations at the level of gene regulation. Figure 1 illustrates the prevalent occurrence of upregulated transcript expression patterns in response to individual and combined heat and light stresses (H/L/HL↑), control conditions and salt stress (C/S↑), combined heat and light stresses (HL↑), and intensive light stress (L↑). C/S↑ and C/S↓, respectively, refer to downregulation and upregulation at all individual and combined stresses except for salt stress. The heatmap in Figure S1 reveals overlapping clusters of differential gene expression in response to various stresses and stress combinations. The fact that there is little change in response to salt stress is likely due to the low concentrations of added salt (50 mM NaCl). Expounding upon the expression profiling, an abundance of gene clusters exhibiting regulatory dynamics under conditions of heightened luminosity was discerned. To further investigate these observations, we searched transcripts to detect light-responsive genes, notably showcasing the upregulation of four isoforms of the gene encoding phytochrome A under conditions of elevated luminosity stress and its combinations (depicted in Figure 2). The upregulation of this gene underscores the plant’s adaptive resilience to cope with the intense luminosity stress.

### 3.2. Detection of Concordantly Expressed DAS/SF Genes

**Figure 3:**
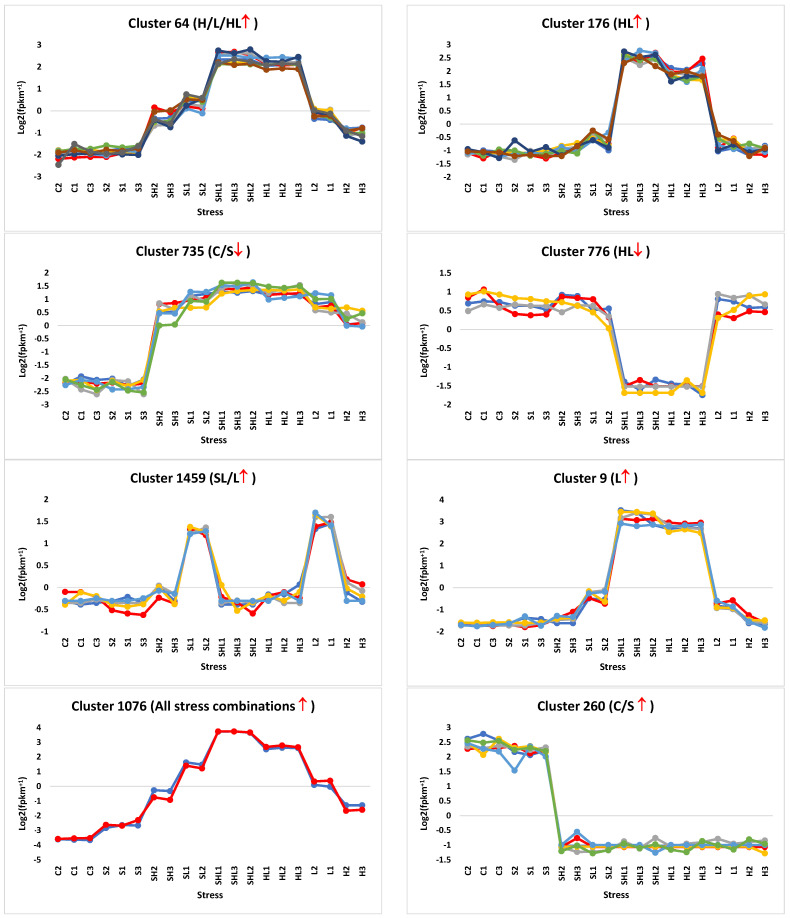
Models of clusters with the most consistent and informative expression patterns (9) generated from transcriptome datasets of 10-day-old *A. thaliana* (wild-type Col-0) seedlings exposed to different multifactorial stress combinations. C = control (0 mM NaCl, 21 °C, 50 µmol m^−2^ s^−1^), S = salt stress (50 mM NaCl, 21 °C, 50 µmol m^−2^ s^−1^), H = heat stress (0 mM NaCl, 33 °C, 50 µmol m^−2^ s^−1^), L = intensive light stress (0 mM NaCl, 21 °C, 700 µmol m^−2^ s^−1^). Further information is available in Table S1. ↑ = upregulation, ↓ = downreguation. Colored lines refer to regulated transcripts.

**Figure 4:**
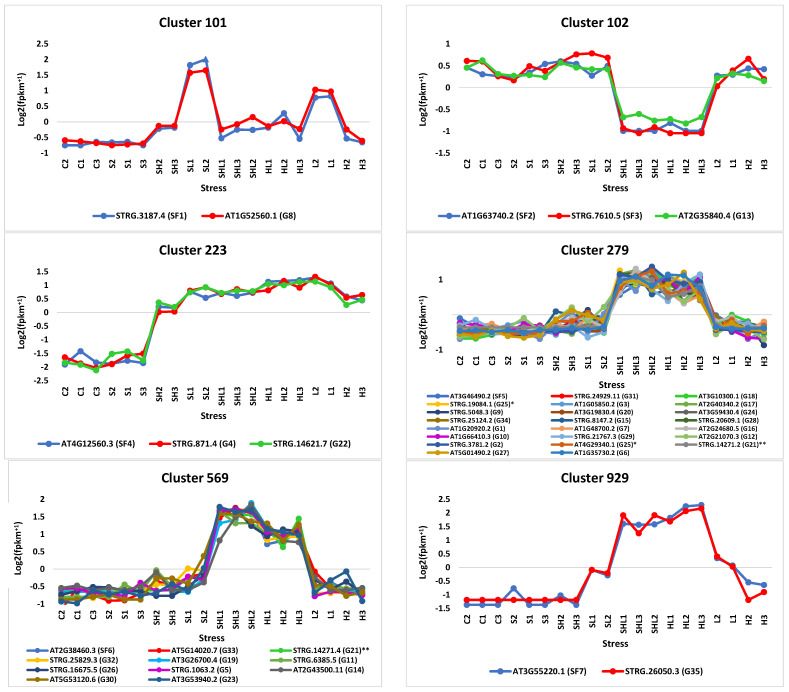
Expression profiling of genes concordantly expressed with splicing factors (SFs) within the most consistent expression patterns generated from transcriptome datasets of 10-day-old *A. thaliana* (wild-type Col-0) seedlings exposed to different multifactorial stress combinations. C = control (0 mM NaCl, 21 °C, 50 µmol m^−2^ s^−1^), S = salt stress (50 mM NaCl, 21 °C, 50 µmol m^−2^ s^−1^), H = heat stress (0 mM NaCl, 33 °C, 50 µmol m^−2^ s^−1^), L = intensive light stress (0 mM NaCl, 21 °C, 700 µmol m^−2^ s^−1^). Detailed information on gene-SF concordant expression within the selected clusters is available in Table S2, while detailed information on the DAS or SFs gene isoforms is shown in Tables S3 and S4, respectively. Single or double asterisks refer to isoforms of the same genes.

The analysis of RNA-Seq datasets revealed the existence of 1974 clusters (Table S1), among which 250 exhibited consistent or discernible expression patterns (Figure S2 and Table S1). Consistent expression, denoting similar expression levels across sample replicates under the same stress condition within a given cluster, was a criterion for the selection of these clusters. These selected clusters encompassed the eight most prominent expression patterns observed in our stress experiment (Figure 3). Subsequently, these clusters were surveyed for the presence of splicing factors (SFs) exhibiting concordant expression with differentially expressed genes, resulting in the identification of six clusters meeting this criterion, i.e., 101, 102, 223, 279, 569, and 929. Within these latter assemblages, a comprehensive examination was conducted to pinpoint differentially expressed genes exhibiting regulatory variation across isoforms within the same or disparate clusters. This discernment effectively shortened the transcriptomic repertoire within these six delineated clusters to 71 distinct differentially expressed alternatively spliced (DAS) gene isoforms (Figure 4 and Table S2). These gene isoforms collectively correspond to a number of 35 DAS genes. Through the utilization of StringTie and GffCompare computational tools, loci harboring either previously annotated or novel isoforms of these genes were strictly delineated, with their respective expression profiles illustrated in Figure S3 and detailed in Table S3. Intriguingly, amidst the cohort of selected SFs, a subset of three exhibited discernible alternative splicing patterns, in which they harbored multiple isoforms of their respective encoding genes, as delineated in Figure S4 and detailed in Table S4.

### 3.3. Analysis of DAS and SF Gene Isoforms

The array of isoforms across all loci within Arabidopsis, as cataloged by the aforementioned computational tools, is exhaustively detailed in Table S5, whereas those corresponding to the concordantly expressed DAS and SF gene variants are elaborated upon in Table S6. The surveyed results shown in Figures S3 and S4 delineate instances where certain isoforms of DAS and SF genes exhibit a lack of distinct regulatory modulation amidst the multifarious stress milieu. Nevertheless, we deemed it imperative to include these inconsistently expressed gene variants within our analysis to discern the prevailing splicing modalities governing their regulated isoforms under stress conditions. Subsequently, a comprehensive examination was undertaken to survey the splicing architectures governing both previously annotated and novel isoforms across the spectrum of 38 disparate DAS and SF genes, thereby providing insights into the prevailing splicing preferences of DAS genes amidst stress conditions (Figures S5–S42). Across these divergent isoforms, encompassing both pre-existing and novel variants, we discerned four types of alternative splicing, namely intron retention, exon skipping, an alternate 5′ donor site, and an alternate 3′ acceptor site, each manifested at varying frequencies.

### 3.4. Validation of New Isoforms and Documented Functionalities of DAS and SF Genes

Among the 35 DAS genes under study, a subset of seven genes underwent further analysis, predicated upon the alignment between their expression profiles in this experimental milieu and their documented functionalities (as depicted in Table S2). The ensuing exploration of isoforms pertaining to these selected genes unveiled a prevalence of six exon skipping events, alongside instance occurrences of five, two, and one of an alternate 3′ acceptor site, an alternate 5′ donor site, and intron retention, respectively (refer to Figures S5, S9, S13, S24, S29, S30 and S38).

**Figure 5:**
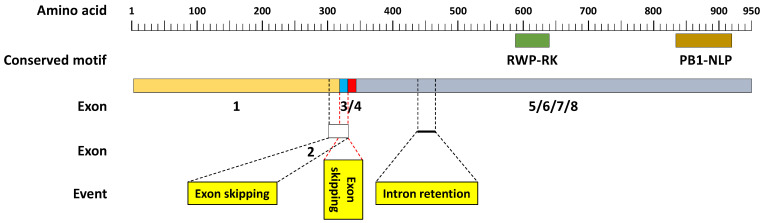
A schematic overview delineating the alternative splicing outcomes discerned within the annotated and novel isoforms originating from the *A. thaliana* locus XLOC_008527, manifested across varying multifaceted stress contexts. Herein, the gene isoform AT2G43500.11, encoding the ABC transporter B family member 11, serves as the foundational sequence for comparative analysis. Within this context, three distinctive splicing events transpired, encompassing the skipping of exons 2 and 3, alongside intron retention occurring within exon 5. Scrutiny at the amino acid sequence level of disparate exons suggests a probable exon skipping event within exon 3, while the remaining events (highlighted in yellow boxes) appear indicative of artifacts. Noteworthy are the active conserved motifs, notably RWP-RK and PB1-NLP, identified within the resultant protein, as informed by recent scholarly contributions. The RWP-RK motif (pfam02042), localized at the C-terminus of this transporter protein, plays a pivotal role in nitrogen-mediated developmental processes, as corroborated by extant literature. Similarly, the PB1 motif (cd06407) is characteristic of NIN-like proteins (NLP), pivotal regulators involved in mediating symbiotic relationships between legumes and nitrogen-fixing bacteria, alongside other critical biological processes. Detailed elucidation pertaining to the functional attributes and expression profiles of distinct isoforms of this gene can be found in Tables S2 and S3, while insights regarding isoform structure are expounded upon in Figures S20 and S43.

Nevertheless, we approached the outcomes pertaining to the novel isoforms with caution, recognizing a tendency for several among them, across a multitude of genes, to potentially harbor artifacts. This speculation is rooted in the intrinsic limitations of the analytical software, which fails to discern both frameshift mutations and premature termination codons within transcript open reading frames (ORFs). This inference was drawn following an exhaustive survey of isoforms derived from a singular gene, namely the ABC transporter B family member 11 (AT2G43500), localized within locus XLOC_008527 (Figure S20). Extensive examination of the aligned amino acid sequences encompassing the five distinct isoforms of this gene revealed instances wherein certain splicing events within the novel isoforms were deemed artifactual (refer to Figure 5 and Figures S43–S48).

**Figure 6:**
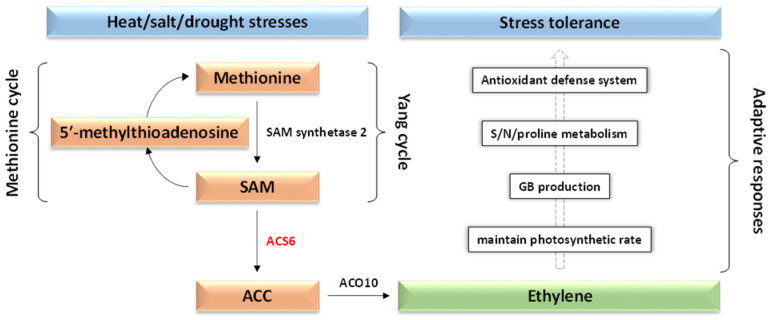
Metabolic pathway emphasizing the role of the DAS gene encoding 1-aminocyclopropane-1-carboxylate synthase 6 (ACS6) in the production of ethylene and tolerance to abiotic stresses. SAM = S-adenosyl-methionine, ACC = 1-aminocyclopropane-1-carboxylate, ACO10 = 1-aminocyclopropane-1-carboxylate oxidase 10, GB = glycine betaine, S = sulfur, N = nitrogen.

Furthermore, our investigation identified the upregulation of the 1-aminocyclopropane-1-carboxylate synthase 6 (ACS6) gene under heat/light stress; this gene plays a pivotal role in ethylene biosynthesis pathway, thereby facilitating adaptive responses and environmental stress tolerance (Figure 6). Additionally, we uncovered two other genes (AT5G40910 and AT4G01850) involved in the ethylene biosynthesis pathway exhibiting expression patterns supportive of upregulation under various stress conditions (Figures S49 and S50). While AT5G40910 displayed alternative splicing with two regulated isoforms, AT4G01850 remained unaltered and non-alternatively spliced despite its confirmed involvement in ethylene biosynthesis. Interestingly, the AS event observed in AT5G40910 did not affect the protein structure/size at the 5′ untranslated region (UTR) of the gene (Figure S50). Such instances, where AS events occur at UTRs without altering protein structure, were recurrently observed across isoforms of various genes (Figures S6, S12, S14, S19–S21, S25, S27, S31, S36, S37, and S40).

## 4. Discussion

The process of precursor-mRNA (pre-mRNA) splicing in plants is intricately linked to the epigenetic chromatin landscape, which influences splice site selection and subsequent post-transcriptional alternative splicing events [29,30,31].

In the realm of Arabidopsis genetics, the prevalence of alternative splicing (AS) phenomena has been previously documented, with estimates suggesting its occurrence in approximately 42% of genes housing intronic sequences, a subset constituting 11.6% of the entire genome [32,33]. However, recent investigations have unveiled a significant augmentation in the AS prevalence, surpassing the 60% threshold within intron-containing genes [34]. Based on the results of the present study, we can claim that the AS apparatus can extend its reach beyond coding sequences, occasionally targeting non-coding regions across diverse isoforms, while maintaining similarity in their encoded sequences (Figures S6, S12, S14, S19–S21, S25, S27, S31, S36, S37, and S40). Hence, it is imperative to authenticate the novel isoform before drawing definitive conclusions regarding the splicing patterns of a particular DAS gene. Exemplifying this phenomenon within our current study, we observe instances such as the isoforms of the *SBT23* gene (AT1G63010) localized within the locus XLOC_005837 (Figure S14).

### 4.1. Cluster Selection and Concordant Expression of DAS/SF Gene Pairs

Cluster analysis of RNA-Seq datasets yielded 250 clusters with consistent expression patterns out of a total of 1974 (Figure S2 and Table S1). Predominant expression patterns within this selection encompassed upregulation under heat, intensive light, and combined heat/intensive light stress (H/L/HL↑), upregulation under combined heat/intensive light stress (HL↑), and no regulation under control and salt stress (C/S↓) (Figure 3). The subsequent tier of cluster curation encompasses those manifesting one among the eight expression patterns elucidated in Figure 3. Globally, it is evident that heightened luminosity stress imposes the most pronounced perturbation in Arabidopsis seedlings, followed by thermal stress. Notably, salinity stress at the prescribed concentration (50 mM NaCl) appears to exert minimal impact, akin to the baseline non-stress condition (Figure S2).

Light stress, particularly the perception of light by red/far-red-absorbing phytochrome photoreceptors, exerts profound effects on plant growth and development [35]. In Arabidopsis, the phytochrome family comprising phyA–phyE plays crucial roles, with phyA prominently involved in seedling de-etiolation and sensing continuous far-red light (cFR) [36,37,38,39]. Light modulates the transcription kinetics of numerous genes and influences AS incidence by favoring specific gene isoforms conducive to optimal stress responses [40,41]. Consistent with this, our RNA-Seq data revealed the induction of four gene isoforms encoding phytochrome A under intensive light and related conditions (Figure 2).

Further refinement of clusters focused on identifying concordant expression of differentially alternatively spliced (DAS) genes and splicing factor (SF) genes. Six clusters, including 101, 102, 223, 279, 569, and 929, with 71 DAS genes, exhibited such concordant expression (Figure 4 and Figure S2 and Table S2). Notably, these clusters predominantly showcased positive transcript responses to intensive light stress, except for cluster 102, which exhibited a negative response to combined intensive light and heat stresses. Subsequent analysis highlighted DAS genes with isoforms distributed across various clusters under distinct stress conditions, totaling 35 genes for further scrutiny (Figure 4 and Table S2). Within the spectrum of the six discerned clusters, the DAS/TF gene isoforms within Cluster 101 exhibited an SL/L↑ expression pattern, while those in Clusters 102, 223, and 929, respectively, showcased HL↓, C/S↓, and H/L/HL↑ profiles, with Clusters 279 and 569 evincing HL↑ dynamics (Figure 4). Predominantly, DAS isoforms across other clusters displayed analogous expression patterns, except for a few isoforms (locus XLOC_008527 as an example), which exhibited variable expression profiles. However, noteworthy are the loci housing some unregulated isoforms under diverse stress conditions (locus XLOC_003623 as an example) (Figure S3 and Table S3). Additionally, three of the six Arabidopsis SFs were observed to possess isoforms (as depicted in Figure S4). Regarding the loci housing these SF isoforms, our findings revealed differential expression patterns within XLOC_001540 and XLOC_012492, whereas the singular isoform within locus XLOC_007097 exhibited no discernible consistent expression patterns under stress conditions (Figure S4 and Table S4).

### 4.2. Fidelity of New DAS Gene Isoforms under Stress

Previously annotated isoforms typically depict only the coding sequences, further complicating the detection of new isoforms (Figures S5–S42). Moreover, a single locus often houses multiple previously annotated genes, necessitating careful analysis to differentiate genuine new isoforms from artifacts (Figure S6 as an illustrative example). An extra stringent layer of DAS selection mandates that new isoforms of a given gene must co-occur with previously annotated isoforms within the locus to bolster their authenticity. Additionally, in cases where a locus encompasses multiple genes, individual gene isoforms within the locus necessitate separate scrutiny as an extra layer of DAS selection. These stringent selection criteria led to the exclusion of isoform investigations for loci XLOC_001851 (Figure S7) and XLOC_019807 (Figure S34).

Prior studies have underscored intron retention (IR) as the predominant form of AS in Arabidopsis during development and under stress conditions [32,34,42,43]. Across the 35 DAS and three SF genes examined in our study, exon skipping and an alternate 3′ site were the favored splicing events under multifactorial stress conditions (Figures S5–S42). Notably, our investigation of isoforms of the gene encoding ABC transporter B family member 11 (AT2G43500) at locus XLOC_008527 revealed a prevalence of AA and AD splice sites under various multifactorial stress conditions (Figure S20). IR events often result in isoforms containing premature termination codons and truncated proteins, while AA and AD splice sites predominantly lead to downstream frameshifts and proteins with altered functions [44,45]. However, our investigation searched the speculation surrounding an intronic presence within exon 5 of the novel gene isoform (i.e., STRG.10463.14) situated within locus XLOC_008527, encoding the ABC transporter B family member 11 and yielding disparate outcomes. Contrary to expectations, the retention of this purported intron in the aforementioned isoform did not engender either the premature stop codons or downstream frameshifts across the four alternative isoforms (Figures S20 and S47). Upon careful examination of the speculated intron’s splicing within isoform STRG.10463.14, a consequential frameshift manifested immediately downstream of the splicing site, with stop codons emerging a mere 20 amino acids post-splice site (Figure S47). Consequently, the hypothesis regarding intronic presence within this exon is invalidated, thereby classifying this gene isoform as an artifact. To substantiate this assertion, we surveyed the active, conserved domains of the resultant protein (Figure S48). Structure and function of these two motifs were previously describes [46,47,48,49]. Active conserved domains in the generated protein were detected based on recent information [48]. Notably, isoforms AT2G43500.11 and STRG.10463.9 displayed an expression pattern characterized by heightened luminosity (HL↑), whereas isoform STRG.10463.14 exhibited a distinctive expression profile across all stress combinations (all stress combinations↑).

Prior studies have suggested that isoforms of a given gene predominantly share identical active domains [17]. Upon surveying the extant active domains within the ABC transporter B family protein, we observed the presence of two such domains localized within exons 7 and 8 across the remaining four isoforms, characterized by the conserved motifs RWP-RK and BP1-NLP, respectively (Figure 5). The structural and functional attributes of these two motifs have been previously elucidated [46,47,48,49]. The apparent absence of these conserved motifs within isoform STRG.10463.14 (Figure S47) underscores its incapacity to fulfill the anticipated functional role of the gene. Conversely, our investigation into the speculated exon 3 skipping within the three previously annotated isoforms—AT2G43500.9, AT2G43500.10, and AT2G43500.11—revealed no occurrence of premature stop codons or frameshift mutations. This observation holds true for both the aforementioned isoforms lacking exon 3 and the novel isoform STRG.10463.9, which retains it. Hence, it is deduced that exon 3 indeed encodes 15 in-frame amino acids (as depicted in Figure S46), substantiating the authenticity of the latter novel isoform. Intriguingly, the disparate functionalities exhibited by the five distinct isoforms within locus XLOC_008527 align with findings from prior investigations [16,17,18]. This overarching observation has relevance across myriad DAS genes analyzed within the present study, as elucidated in Tables S3 and S4. Therefore, careful attention is warranted when surveying novel isoforms of any given gene.

### 4.3. Functional Analysis of Concordantly Expressed DAS/SF Genes under Stress

A crucial aspect of this study is ensuring the conformity between the expression patterns and the documented functions of concordantly expressed DAS/SF gene pairs. Consequently, selection was carried out for the concordantly expressed DAS/SF gene pairs across the six clusters previously established as experimentally stress-related. DAS genes lacking prior information on their response to any stress combinations in this study were excluded from further analysis (Table S2). Notably, the DAS/SF pair in cluster 929 was not analyzed due to the absence of available information on the putative splicing factor (Table S2). The findings of this study revealed that SFs concordantly expressed with DAS genes predominantly belong to the highly conserved, multi-domain, non-snRNP spliceosome-related large family of RNA-binding proteins known as serine/arginine-rich (SR) splicing factors [50] (Table S2). Members of the SR protein family typically feature two RNA binding domains (RBDs), an arginine/serine-rich (RS) domain, and multiple RS dipeptide repeats at the C terminus [51].

In this study, six SR splicing factors across five clusters (i.e., 101, 102, 223, 279, and 569) were implicated in the alternative splicing of 34 out of the 35 DAS genes under different intensive light stress combinations (Figure 4). The SF in the seventh cluster (i.e., 929) is putative, thus its respective DAS gene was not investigated further. Among the other identified SR proteins, CACTIN, SR1-like, and SR30 were observed to undergo alternative splicing of their own pre-mRNAs under stress conditions, while SC35-like exhibited no such tendency, as evidenced by the presence of only one isoform encoding this protein in our RNA-Seq datasets (Table S1). Furthermore, the two *SR45a* genes residing in loci XLOC_002473 and XLOC_007097 were not found to generate isoforms of their own pre-mRNA (Table S1).

In Cluster 101, the concordant expression of SR-like *cactin* and a DAS gene encoding a 26.5 kDa heat shock protein was observed, exhibiting upregulation under intensive light stress and its combination with salt stress. Although the function of *cactin* remains elusive, it is speculated to play a role in alternative splicing due to the presence of a serine/arginine-rich (SR) domain at the N terminal [52,53]. As a small heat shock protein (sHSP), 26.5 kDa, acting as a molecular chaperone, aids in protecting proteins from stress-induced damage [54]. Despite its documented involvement in abiotic stress responses, its specific association with light stress remains unexplored (https://www.uniprot.org/uniprotkb/Q9SSQ8/entry?version=*, accessed on 1 March 2024). The observed splicing types for the DAS gene encoding the 26.5 kDa heat shock protein involve an alternate 3′ acceptor site for the isoforms of the *sHSP* gene (AT1G52560, locus XLOC_005378) and an alternate 5′ donor site for those of *cactin* gene (AT1G32870, locus XLOC_001540) (Figures S13 and S6, respectively). Given the limited information on *cactin* gene, its potential response to intensive light stress and its involvement in alternative splicing of the DAS gene remain speculative.

Cluster 102 denotes the concordant expression of two genetic loci, namely *SR45a* and a DAS gene isoform encoding heat shock 70 kDa protein 16, as delineated in Figure 4 and Table S2. The expression profile of this cluster showcases a diminution under the combined influence of intensive light and heat stresses or an increase under salt stress conditions. The SR45a protein, an integral constituent of the spliceosome machinery, was previously implicated in modulating responses to salt stress. Furthermore, it serves as a pivotal mediator in salt stress signal transduction pathways, functioning as a splicing factor for genes associated with salt stress in Arabidopsis. Its role encompasses facilitating the bridging between the 5′ and 3′ splice sites during spliceosome assembly [55,56,57,58,59]. Recent literature highlights the induction of the two co-expressed genes of *SR45a* under the influence of salt stress [58,59]. Notably, gene ontology (GO) annotation results suggest its responsiveness to light signaling (https://www.uniprot.org/uniprotkb/Q84TH4/entry#Q84TH4-2, accessed on 1 March 2024). The DAS gene encoding the heat shock 70 kDa protein 16, a cytosolic chaperone, aids in protein folding, degradation, and translocation, conferring tolerance against heat and osmotic stresses [60,61]. GO annotation further indicates its positive response to light signaling (https://www.uniprot.org/uniprotkb/Q9SAB1/entry, accessed on 1 March 2024). However, the documented functions of this DAS/SF pair do not align with those observed under the multifactorial stress combinations.

Cluster 223 features the concordant expression of an isoform of SR-like 1 and isoforms of two DAS genes encoding the chaperone protein DnaJ (or HSP40) and the shaker-type potassium channel GORK. This cluster exhibits downregulation under salt stress and upregulation under all other stress combinations. *SRL1* gene appears to exhibit no alternative splicing under the stress combinations in this study (Table S1). It was reported to participate in heat stress tolerance [62], with GO annotation suggesting its response to light stimulus (https://www.uniprot.org/uniprotkb/Q94L34/entry, accessed on 1 March 2024). The DAS gene encoding the chaperone protein DnaJ promotes protein homeostasis and positively responds to heat shock [63,64]. Although GORK’s documented function differs substantially from the concordantly expressed SF, its expression pattern aligns with this cluster’s stress response profile. GORK enhances sensitivity to ABA and negatively responds to salt and osmotic stresses via phosphatase 2A- or PP2CA-mediated signals [65]. Regarding the splicing modalities exhibited by the gene encoding DnaJ (STRG.871.4), it is noteworthy that two previously documented isoforms of an alternate stress-responsive gene coexist within the same locus (i.e., XLOC_003799), as illustrated in Figure S9. Consequently, our analysis is confined solely to the two novel isoforms of the DAS gene encoding DnaJ, which demonstrate concordant expression with the SR-like 1 gene. Notably, the splice type observed in these two novel isoforms (i.e., STRG.871.1/STRG.871.4) manifests as alternate 3′ acceptor site utilization. Turning to the splicing patterns exhibited by isoforms encoding GORK, our findings unveil instances of exon skipping and intron retention within the stress-responsive novel isoforms (i.e., STRG.14621, XLOC_015088), as delineated in Figure S29. Note that the analysis did not encompass exons/introns in the new isoforms of the *GORK* gene, as they appear to belong to another gene.

Cluster 279 epitomizes the coherent co-expression of an isoform pertaining to the gene encoding SR30 alongside isoforms of three DAS genes encoding mitogen-activated protein kinase kinase kinase 5 (MAPKKK5), calcium-binding EF hand family protein, and DEAD-box RNA helicase, as depicted in Figure 4 and Table S2. The expression profile characteristic of this cluster is the upregulation in response to the combined stresses of intensive light and heat. SR30’s established involvement in spliceosome assembly and the modulation of specific plant gene splicing further underscores its functional significance within this context [66]. Alternative splicing mediated by this splicing factor exhibits tissue- and developmental stage-specificity, primarily exerting its functional influence during early seedling development and root differentiation. Recent investigations have shed light on the observation that the protein product encoded by this splicing factor accumulates in response to both cold and heat stresses [58,59], with GO annotation suggesting its responsiveness to light stimulation and participation in stress tolerance mechanisms (https://www.uniprot.org/uniprotkb/Q9XFR5/entry#Q9XFR5-2, accessed on 1 March 2024). As an intricate cascade of phosphorylation and signal transduction processes, MAPKKK initiates the activation of MAP kinase kinase (MAPKK), subsequently facilitating the activation of MAP kinase (MAPK) [67]. The gene encoding MAPKKK5 (MAP3K5), a serine/threonine kinase protein, is implicated in a myriad of cellular processes triggered by oxidative stresses, cellular differentiation, and survival mechanisms, as well as in orchestrating the mitochondria-dependent apoptosis signal transduction cascade [67,68,69,70]. Notably, in rice, MAP3K5 has been implicated in the regulation of cell size through modulation of endogenous gibberellin levels [71]. However, extant literature lacks a precedent for elucidating the response of the *MAP3K5* gene to heat or light stress. Hence, we must regard its reaction to light and heat stresses as an indeterminate phenomenon.

The DAS gene encoding the calcium-binding EF-hand family protein plays a pivotal role in enhancing plant resilience to abiotic stresses. Upon exposure to external stimuli, plant cells undergo a differential response, culminating in an elevation of cytoplasmic calcium levels. This surge in calcium concentration is perceived by specific cellular proteins, such as Ca^2+^-binding proteins or Ca^2+^ sensors, which undergo conformational changes to facilitate interactions with signal transduction molecules necessary for their activation. Among these sensors, a subclass known as Ca^2+^-dependent protein kinases (CDPKs) assumes a crucial role in modulating the expression of light- and heat-stress-responsive genes. Notably, the EF-hand motif within calcium-binding proteins comprises a structural arrangement of two α-helices, thereby fortifying the plant’s response to abiotic stresses [72]. In the context of splicing modalities pertaining to the DAS gene (AT3G10300) harbored within locus XLOC_011288, the data presented in Figure S24 elucidates the presence of three instances of exon skipping, alongside a solitary event involving alternate 3′ acceptor site utilization across the isoforms of this gene.

The DAS gene encoding DEAD-box RNA helicase has been documented to play a pivotal role in facilitating plant adaptation to intensive light conditions [73]. This function is intricately mediated through the induction of ribosome biogenesis, a process crucial for increasing gene transcription and translation [74]. Consequently, exposure to intensive light serves as a mechanism to enhance the photosynthetic capacity of plant cells by supporting plastid ribosome abundance, thereby enabling the overexpression of a suite of light-responsive genes [73,74]. Photosynthetic organisms intricately orchestrate a complex regulatory network to finely modulate the capture and conversion of light energy, aiming to mitigate the risk of photodamage arising from imbalances between light energy conversion and utilization processes [75,76,77]. Alterations in light intensity precipitate shifts in energy conversion rates, serving as a mechanism to optimize cellular metabolic demands amidst fluctuating environmental conditions [73]. The regulatory influence of the DAS gene extends to overseeing light-dependent ribosomal RNA precursor maturation in accordance with the exigencies of plant cellular physiology. This regulatory framework necessitates diverse forms of the DAS gene to dynamically respond to variances in light intensity. The examination of splicing modalities pertaining to the DAS gene (AT1G20920) housed within locus XLOC_000999, as depicted in Figure S5, reveals the occurrence of alternate 5′ donor site utilization within the solitary exon of this gene.

Cluster 569 delineates the coordinated expression of the gene encoding SC35-like splicing factor 33 (SCL33) alongside the DAS gene encoding 1-aminocyclopropane-1-carboxylate synthase 6 (ACC synthase 6 or ACS6), as depicted in Figure 4 and Table S2. The expression profile of this cluster demonstrates upregulation under conditions of combined intensive light and heat stresses. The GO annotation highlights the light-responsive nature of the splicing factor encoding gene (https://www.uniprot.org/uniprotkb/Q9SEU4/entry, accessed on 1 March 2024). Notably, a motif termed GAA, present in numerous proteins, is implicated in bolstering exonic splicing enhancer activity by facilitating the recruitment of appropriate splicing factors for alternative splicing events [78]. The identified motif has been validated to govern intron splicing in red light-responsive genes through the recruitment of the SR protein SCL33 [79]. While the original splicing pattern facilitated by this splicing factor entails intron retention, our investigation did not detect this particular splicing event in the light/heat responsive isoforms of the DAS gene (AT3G53940), as illustrated in Figure S30. Instead, our findings revealed the occurrence of two alternative splicing modalities, namely exon skipping and alternative 5′ donor site utilization. Gene ontology annotation results further corroborated the regulatory influence of light stress on the gene encoding this enzyme (https://www.uniprot.org/uniprotkb/Q9XFI3/entry, accessed on 1 March 2024). The ACC generated by the ACS6 enzyme, encoded by this DAS gene, has recently been noted to serve as an intermediary metabolite in ethylene biosynthesis [80]. Ethylene, a stress-responsive phytohormone [81], plays a pivotal role in modulating plant growth in adverse environmental conditions [82,83]. The orchestrated activity of the regulated ACS6 and ACC oxidase 10 (ACO10, encoded by AT5G40910) enzymes constitutes the primary biosynthetic pathway responsible for ethylene synthesis [83]. In the present study, two alternatively spliced isoforms of the gene encoding ACO10 were found to be under regulatory influence during stress conditions (Figure S49). Within plants, the biosynthesis of ethylene necessitates the utilization of the sulfur-containing amino acid methionine as the principal substrate for the enzymatic reaction (Figure 6) [84]. Initially, the enzyme S-adenosyl-methionine (SAM) synthetase 2, encoded by AT4G01850, catalyzes the conversion of methionine to SAM within the Yang cycle [84,85,86]. One isoform of this gene undergoes regulation in response to stress conditions, as depicted in Figure S50. Subsequently, SAM undergoes conversion into 1-aminocyclopropane-1-carboxylate (ACC), catalyzed by the enzyme 1-aminocyclopropane-1-carboxylate synthase 6 (ACS6) [87]. Concurrently, the production of 5′-methylthioadenosine from SAM occurs, serving as a precursor for methionine regeneration via the methionine cycle, thereby replenishing the methyl group for subsequent rounds of ethylene biosynthesis [88]. ACC, serving as the direct precursor to ethylene, is enzymatically converted by 1-aminocyclopropane-1-carboxylate oxidase 10 (ACO10) to generate ethylene (Figure 6). The latter compound is synthesized in response to abiotic stressors such as drought, salt, and heat stresses, thereby instigating a series of adaptive responses [80]. These responses include the maintenance of the photosynthetic rate, the production of the osmolyte glycine betaine (GB), and other antioxidant compounds (Figure 6), collectively empowering plants with the capacity to withstand challenging environmental conditions [89,90,91].

## 5. Conclusions

In conclusion, the corroborated data pertaining to the six DAS genes within clusters 101, 223, 279, and 569, exhibiting concordant expression with four SFs, substantiates the findings obtained in this study. However, careful attention is warranted when examining new isoforms of any gene prior to exploring alternative splicing events. Consequently, we posit that the associations observed among these DAS/SF genes across distinct clusters warrant further experimental elucidation. Systematically cataloging and leveraging such associations hold promise for unveiling novel genetic-based avenues toward bolstering climate resilience, enhancing plant productivity, and augmenting the nutritional profile of cultivated crops.

## Figures and Tables

**Table 1:** Information available for RNA-Seq datasets of 10-day-old *A. thaliana* (wild-type Col-0) seedling samples exposed to different multifactorial stress combinations. C = control (0 mM NaCl, 21 °C, 50 µmol m^−2^ s^−1^), S = salt stress (50 mM NaCl, 21 °C, 50 µmol m^−2^ s^−1^), H = heat stress (0 mM NaCl, 33 °C, 50 µmol m^−2^ s^−1^), L = intensive light stress (0 mM NaCl, 21 °C, 700 µmol m^−2^ s^−1^). Further information is available in Table S1, and further growth and abiotic stress conditions were recently reported [26].

Sample	Accession	Sample	Accession	Sample	Accession	Sample	Accession
C1	SRX8045189	S3	SRX8045194	SHL1	SRX8045228	HL3	SRX8045227
C2	SRX8045190	SH2	SRX8045217	SHL2	SRX8045229	L1	SRX8045201
C3	SRX8045191	SH3	SRX8045218	SHL3	SRX8045230	L2	SRX8045202
S1	SRX8045192	SL1	SRX8045204	HL1	SRX8045225	H2	SRX8045214
S2	SRX8045193	SL2	SRX8045205	HL2	SRX8045226	H3	SRX8045215

## Data Availability

Data are contained within the article and Supplementary Materials at: https://www.mdpi.com/article/10.3390/genes15060675/s1.

## Supplementary Materials

The following supporting information can be downloaded at: https://www.mdpi.com/article/10.3390/genes15060675/s1. Table S1. Cluster analysis of transcriptome datasets of 10-d-old *A. thaliana* (wild-type Col-0) seedlings exposed to different multifactorial stress combinations. C = control (0 mM NaCl, 21 °C, 50 µmol m^−2^ s^−1^), S = salt stress (50 mM NaCl, 21 °C, 50 µmol m^−2^ s^−1^), H = heat stress (0 mM NaCl, 33 °C, 50 µmol m^−2^ s^−1^), L = high light stress (0 mM NaCl, 21 °C, 700 µmol m^−2^ s^−1^). Further growth and abiotic stress conditions were recently reported [26]. Red text refers to the most consistent expression patterns analyzed further. Yellow box refers to splicing factors (SFs), while bright green box refers to concordantly expressed genes with 2 or more regulated isoforms and bright blue box refers to concordantly expressed genes with no regulated isoforms; Table S2. Detailed description of DAS gene isoforms concordantly expressed with one or more splicing factors (SFs) within the transcriptome datasets of 10-d-old *A. thaliana* (wild-type Col-0) seedlings exposed to different multifactorial stress combinations. C = control (0 mM NaCl, 21 °C, 50 µmol m^−2^ s^−1^), S = salt stress (50 mM NaCl, 21 °C, 50 µmol m^−2^ s^−1^), H = heat stress (0 mM NaCl, 33 °C, 50 µmol m^−2^ s^−1^), L = high light stress (0 mM NaCl, 21 °C, 700 µmol m^−2^ s^−1^). Red text refers to gene isoforms that are not stress-related, thus not analyed further. Blue text refers to splicing factors. Yellow box refers to splicing factors (SFs), while bright green box refers to concordantly expressed genes with 2 or more regulated isoforms; Table S3. Detailed description of DAS gene isoforms concordantly expressed with one or more splicing factors (SFs) within the transcriptome datasets of 10-d-old *A. thaliana* (wild-type Col-0) seedlings exposed to different multifactorial stress combinations. C = control (0 mM NaCl, 21 °C, 50 µmol m^−2^ s^−1^), S = salt stress (50 mM NaCl, 21 °C, 50 µmol m^−2^ s^−1^), H = heat stress (0 mM NaCl, 33 °C, 50 µmol m^−2^ s^−1^), L = high light stress (0 mM NaCl, 21 °C, 700 µmol m^−2^ s^−1^). Red text refers to gene isoforms that are not stress-related, thus not analyed further. Blue text refers to a stress-related gene with no distinctive isoform. Bright green box refers to concordantly expressed genes with 2 or more regulated isoforms; Table S4. Detailed description of splicing factors with isoforms among those concordantly expressed with one or more DAS gene isoforms within the transcriptome datasets of 10-d-old *A. thaliana* (wild-type Col-0) seedlings exposed to different multifactorial stress combinations. C = control (0 mM NaCl, 21 °C, 50 µmol m^−2^ s^−1^), S = salt stress (50 mM NaCl, 21 °C, 50 µmol m^−2^ s^−1^), H = heat stress (0 mM NaCl, 33 °C, 50 µmol m^−2^ s^−1^), L = high light stress (0 mM NaCl, 21 °C, 700 µmol m^−2^ s^−1^). Red text refers to gene isoforms that are stress-related, while blue text refers to the original SF isoforms that are concordantly expressed with stress-related gene isoform(s); Table S5. Description of loci with regulated DAS or SF gene isoforms in *A. thaliana* (Col-0) in terms of chromosome number and locus location as well as annotated and new isoforms; Table S6. Description of all loci in *A. thaliana* (Col-0) in terms of chromosome number and locus location as well as annotated and new isoforms; Figure S1. Heatmap referring to 2-D hierarchical clusters of gene expression generated from transcriptome datasets of 10-d-old *A. thaliana* (wild-type Col-0) seedlings exposed to different multifactorial stress combinations. C = control (0 mM NaCl, 21 °C, 50 µmol m^−2^ s^−1^), S = salt stress (50 mM NaCl, 21 °C, 50 µmol m^−2^ s^−1^), H = heat stress (0 mM NaCl, 33 °C, 50 µmol m^−2^ s^−1^), L = high light stress (0 mM NaCl, 21 °C, 700 µmol m^−2^ s^−1^). Further information is available in Table S1; Figure S2. Expression profiling of the most consistent expression patterns generated from transcriptome datasets of 10-d-old *A. thaliana* (wild-type Col-0) seedlings exposed to different multifactorial stress combinations. C = control (0 mM NaCl, 21 °C, 50 µmol m^−2^ s^−1^), S = salt stress (50 mM NaCl, 21 °C, 50 µmol m^−2^ s^−1^), H = heat stress (0 mM NaCl, 33 °C, 50 µmol m^−2^ s^−1^), L = high light stress (0 mM NaCl, 21 °C, 700 µmol m^−2^ s^−1^). Further growth and abiotic stress conditions were recently reported [26]. Sequences can be found in Bioproject PRJNA622644. Detailed information of all gene clusters are shown in Table S1; Figure S3. Expression profiling of loci involving DAS genes concordantly expressed with splicing factors (SFs) within the most consistent expression patterns generated from transcriptome datasets of 10-d-old *A. thaliana* (wild-type Col-0) seedlings exposed to different multifactorial stress combinations. C = control (0 mM NaCl, 21 °C, 50 µmol m^−2^ s^−1^), S = salt stress (50 mM NaCl, 21 °C, 50 µmol m^−2^ s^−1^), H = heat stress (0 mM NaCl, 33 °C, 50 µmol m^−2^ s^−1^), L = high light stress (0 mM NaCl, 21 °C, 700 µmol m^−2^ s^−1^). Selected genes should have one or more annotated or new stress-regulated isoforms existing on the same chromosome locus. Isoforms in light gray are not stress-regulated. Detailed information of gene-SF concordant expression is available in Table S3, while that of gene loci are shown in Table S6. Isoforms in light gray are not stress-regulated; Figure S4. Expression profiling of loci with selected splicing factors that are concordantly expressed with DAS genes within the most consistent expression patterns generated from transcriptome datasets of 10-d-old *A. thaliana* (wild-type Col-0) seedlings exposed to different multifactorial stress combinations. C = control (0 mM NaCl, 21 °C, 50 µmol m^−2^ s^−1^), S = salt stress (50 mM NaCl, 21 °C, 50 µmol m^−2^ s^−1^), H = heat stress (0 mM NaCl, 33 °C, 50 µmol m^−2^ s^−1^), L = high light stress (0 mM NaCl, 21 °C, 700 µmol m^−2^ s^−1^). Selected splicing factors should have one or more Annotated or new stress-regulated isoforms existing on the same chromosome locus. Concordantly expressed splicing factors and their isoforms are shown in Table S4, while that of gene loci are shown in Table S6. Isoforms in light gray are not stress-regulated; Figure S5. Structure of previously annotated and new DAS isoforms on XLOC_000999 (Gene 1, AT1G20920) generated due to different multifactorial stress combinations in the transcriptome datasets of 10-d-old A. thaliana (wild-type Col-0) seedlings. Red arrow refers to the gene isoform concordantly expressed with a given splicing factor, while green arrow(s) refer to other regulated isoforms of this gene. Other isoforms are not consistently regulated under the stress. Further information is available in Tables S1, S3 and S6; Figure S6. Structure of previously annotated and new SF isoforms on XLOC_001540 (SF1, AT1G32870) generated due to different multifactorial stress combinations in the transcriptome datasets of 10-d-old A. thaliana (wild-type Col-0) seedlings. Red arrow refers to the SF isoform concordantly expressed with a given gene, while green arrow(s) refer to other regulated isoforms of this gene. Other isoforms are not consistently regulated under the stress. Further information is available in Tables S1, S4 and S6; Figure S7. Structure of previously annotated and new DAS isoforms on XLOC_001851 (Gene 2, STRG.3781.2) generated due to different multifactorial stress combinations in the transcriptome datasets of 10-d-old A. thaliana (wild-type Col-0) seedlings. Red arrow refers to the gene isoform concordantly expressed with a given splicing factor, while green arrow(s) refer to other regulated isoforms of this gene. Other isoforms are not consistently regulated under the stress. Further information is available in Tables S1, S3 and S6. Regulated new isoforms of this DAS gene have no annotated gene to compare with, thus, was not analyzed further; Figure S8. Structure of previously annotated and new DAS isoforms on XLOC_003623 (Gene 3, AT1G05850) generated due to different multifactorial stress combinations in the transcriptome datasets of 10-d-old *A. thaliana* (wild-type Col-0) seedlings. Red arrow refers to the gene isoform concordantly expressed with a given splicing factor, while green arrow(s) refer to other regulated isoforms of this gene. Other isoforms are not consistently regulated under the stress. One of the three isoforms (e.g., AT1G05870.12) belongs to another gene in this locus. The other two isoforms of the gene AT1G05850 show no clear case of alternative splicing, thus isoforms of this locus were not considered for further analysis. Further information is available in Tables S1, S3 and S6; Figure S9. Structure of previously annotated and new DAS isoforms on XLOC_003799 (Gene 4, STRG.871) generated due to different multifactorial stress combinations in the transcriptome datasets of 10-d-old *A. thaliana* (wild-type Col-0) seedlings. Red arrow refers to the gene isoform concordantly expressed with a given splicing factor, while green arrow(s) refer to other regulated isoforms of this gene. Other isoforms are not consistently regulated under the stress. Further information is available in Tables S1, S3 and S6; Figure S10. Structure of previously annotated and new DAS isoforms on XLOC_003907 (Gene 5, STRG.1063) generated due to different multifactorial stress combinations in the transcriptome datasets of 10-d-old *A. thaliana* (wild-type Col-0) seedlings. Red arrow refers to the gene isoform concordantly expressed with a given splicing factor, while green arrow(s) refer to other regulated isoforms of this gene. Other isoforms are not consistently regulated under the stress. Further information is available in Tables S1, S3 and S6; Figure S11. Structure of previously annotated and new DAS isoforms on XLOC_005026 (Gene 6, AT1G35730) generated due to different multifactorial stress combinations in the transcriptome datasets of 10-d-old *A. thaliana* (wild-type Col-0) seedlings. Red arrow refers to the gene isoform concordantly expressed with a given splicing factor, while green arrow(s) refer to other regulated isoforms of this gene. Other isoforms are not consistently regulated under the stress. Further information is available in Tables S1, S3 and S6; Figure S12. Structure of previously annotated and new DAS isoforms on XLOC_005205 (Gene 7, AT1G48700) generated due to different multifactorial stress combinations in the transcriptome datasets of 10-d-old *A. thaliana* (wild-type Col-0) seedlings. Red arrow refers to the gene isoform concordantly expressed with a given splicing factor, while green arrow(s) refer to other regulated isoforms of this gene. Other isoforms are not consistently regulated under the stress. Further information is available in Tables S1, S3 and S6; Figure S13. Structure of previously annotated and new DAS isoforms on XLOC_005378 (Gene 8, AT1G52560) generated due to different multifactorial stress combinations in the transcriptome datasets of 10-d-old *A. thaliana* (wild-type Col-0) seedlings. Red arrow refers to the gene isoform concordantly expressed with a given splicing factor, while green arrow(s) refer to other regulated isoforms of this gene. Other isoforms are not consistently regulated under the stress. Further information is available in Tables S1, S3 and S6; Figure S14. Structure of previously annotated and new DAS isoforms on XLOC_005837 (Gene 9, AT1G63010) generated due to different multifactorial stress combinations in the transcriptome datasets of 10-d-old *A. thaliana* (wild-type Col-0) seedlings. Red arrow refers to the gene isoform concordantly expressed with a given splicing factor, while green arrow(s) refer to other regulated isoforms of this gene. Other isoforms are not consistently regulated under the stress. Further information is available in Tables S1, S3 and S6; Figure S15. Structure of previously annotated and new DAS isoforms on XLOC_006004 (Gene 10, AT1G66410) generated due to different multifactorial stress combinations in the transcriptome datasets of 10-d-old *A. thaliana* (wild-type Col-0) seedlings. Red arrow refers to the gene isoform concordantly expressed with a given splicing factor, while green arrow(s) refer to other regulated isoforms of this gene. Other isoforms are not consistently regulated under the stress. Further information is available in Tables S1, S3 and S6; Figure S16. Structure of previously annotated and new DAS isoforms on XLOC_006475 (Gene 11, STRG.6385) generated due to different multifactorial stress combinations in the transcriptome datasets of 10-d-old *A. thaliana* (wild-type Col-0) seedlings. Red arrow refers to the gene isoform concordantly expressed with a given splicing factor, while green arrow(s) refer to other regulated isoforms of this gene. Other isoforms are not consistently regulated under the stress. Further information is available in Tables S1, S3 and S6; Figure S17. Structure of previously annotated and new SF isoforms on XLOC_007097 (SF3, AT2G14080) generated due to different multifactorial stress combinations in the transcriptome datasets of 10-d-old *A. thaliana* (wild-type Col-0) seedlings. Red arrow refers to the SF isoform concordantly expressed with a given gene, while green arrow(s) refer to other regulated isoforms of this gene. Other isoforms are not consistently regulated under the stress. Further information is available in Tables S1, S4 and S6; Figure S18. Structure of previously annotated and new DAS isoforms on XLOC_007392 (Gene 12, AT2G21070) generated due to different multifactorial stress combinations in the transcriptome datasets of 10-d-old *A. thaliana* (wild-type Col-0) seedlings. Red arrow refers to the gene isoform concordantly expressed with a given splicing factor, while green arrow(s) refer to other regulated isoforms of this gene. Other isoforms are not consistently regulated under the stress. Further information is available in Tables S1, S3 and S6; Figure S19. Structure of previously annotated and new DAS isoforms on XLOC_008122 (Gene 13, AT2G35840) generated due to different multifactorial stress combinations in the transcriptome datasets of 10-d-old *A. thaliana* (wild-type Col-0) seedlings. Red arrow refers to the gene isoform concordantly expressed with a given splicing factor, while green arrow(s) refer to other regulated isoforms of this gene. Red arrow refers to the gene isoform concordantly expressed with a given splicing factor, while green arrow(s) refer to other regulated isoforms of this gene. Other isoforms are not consistently regulated under the stress. Further information is available in Tables S1, S3 and S6; Figure S20. Structure of previously annotated and new DAS isoforms on XLOC_008527 (Gene 14, AT2G43500) generated due to different multifactorial stress combinations in the transcriptome datasets of 10-d-old *A. thaliana* (wild-type Col-0) seedlings. Red arrow refers to the gene isoform concordantly expressed with a given splicing factor, while green arrow(s) refer to other regulated isoforms of this gene. Other isoforms are not consistently regulated under the stress. Further information is available in Tables S1, S3 and S6. The two events of alternative splicing, e.g., exon skipping and intron retention, were deeply investigated. Active conserved domains in the generated protein were detected based on recent information [48]; Figure S21. Structure of previously annotated and new DAS isoforms on XLOC_009386 (Gene 15, STRG.8147) generated due to different multifactorial stress combinations in the transcriptome datasets of 10-d-old *A. thaliana* (wild-type Col-0) seedlings. Red arrow refers to the gene isoform concordantly expressed with a given splicing factor, while green arrow(s) refer to other regulated isoforms of this gene. Other isoforms are not consistently regulated under the stress. Further information is available in Tables S1, S3 and S6; Figure S22. Structure of previously annotated and new DAS isoforms on XLOC_009607 (Gene 16, AT2G24680) generated due to different multifactorial stress combinations in the transcriptome datasets of 10-d-old *A. thaliana* (wild-type Col-0) seedlings. Red arrow refers to the gene isoform concordantly expressed with a given splicing factor, while green arrow(s) refer to other regulated isoforms of this gene. Other isoforms are not consistently regulated under the stress. Further information is available in Tables S1, S3 and S6; Figure S23. Structure of previously annotated and new DAS isoforms on XLOC_010435 (Gene 17, AT2G40340) generated due to different multifactorial stress combinations in the transcriptome datasets of 10-d-old *A. thaliana* (wild-type Col-0) seedlings. Red arrow refers to the gene isoform concordantly expressed with a given splicing factor, while green arrow(s) refer to other regulated isoforms of this gene. Other isoforms are not consistently regulated under the stress. Further information is available in Tables S1, S3 and S6; Figure S24. Structure of previously annotated and new DAS isoforms on XLOC_011288 (Gene 18, AT3G10300) generated due to different multifactorial stress combinations in the transcriptome datasets of 10-d-old *A. thaliana* (wild-type Col-0) seedlings. Red arrow refers to the gene isoform concordantly expressed with a given splicing factor, while green arrow(s) refer to other regulated isoforms of this gene. Other isoforms are not consistently regulated under the stress. Further information is available in Tables S1, S3 and S6; Figure S25. Structure of previously annotated and new DAS isoforms on XLOC_012141 (Gene 19, AT3G26700) generated due to different multifactorial stress combinations in the transcriptome datasets of 10-d-old *A. thaliana* (wild-type Col-0) seedlings. Red arrow refers to the gene isoform concordantly expressed with a given splicing factor, while green arrow(s) refer to other regulated isoforms of this gene. Other isoforms are not consistently regulated under the stress. Further information is available in Tables S1, S3 and S6; Figure S26. Structure of previously annotated and new SF isoforms on XLOC_012492 (SF5, AT3G46490) generated due to different multifactorial stress combinations in the transcriptome datasets of 10-d-old *A. thaliana* (wild-type Col-0) seedlings. Red arrow refers to the SF isoform concordantly expressed with a given gene, while green arrow(s) refer to other regulated isoforms of this gene. Other isoforms are not consistently regulated under the stress. Further information is available in Tables S1, S4 and S6; Figure S27. Structure of previously annotated and new DAS isoforms on XLOC_014308 (Gene 20, AT3G19830) generated due to different multifactorial stress combinations in the transcriptome datasets of 10-d-old *A. thaliana* (wild-type Col-0) seedlings. Red arrow refers to the gene isoform concordantly expressed with a given splicing factor, while green arrow(s) refer to other regulated isoforms of this gene. Other isoforms are not consistently regulated under the stress. Further information is available in Tables S1, S3 and S6; Figure S28. Structure of previously annotated and new DAS isoforms on XLOC_014905 (Gene 21, STRG.14271) generated due to different multifactorial stress combinations in the transcriptome datasets of 10-d-old *A. thaliana* (wild-type Col-0) seedlings. Red arrow refers to the gene isoform concordantly expressed with a given splicing factor, while green arrow(s) refer to other regulated isoforms of this gene. Other isoforms are not consistently regulated under the stress. Further information is available in Tables S1, S3 and S6; Figure S29. Structure of previously annotated and new DAS isoforms on XLOC_015088 (Gene 22, STRG.14621) generated due to different multifactorial stress combinations in the transcriptome datasets of 10-d-old *A. thaliana* (wild-type Col-0) seedlings. Red arrow refers to the gene isoform concordantly expressed with a given splicing factor, while green arrow(s) refer to other regulated isoforms of this gene. Other isoforms are not consistently regulated under the stress. Further information is available in Tables S1, S3 and S6; Figure S30. Structure of previously annotated and new DAS isoforms on XLOC_015398 (Gene 23, AT3G53940) generated due to different multifactorial stress combinations in the transcriptome datasets of 10-d-old *A. thaliana* (wild-type Col-0) seedlings. Red arrow refers to the gene isoform concordantly expressed with a given splicing factor, while green arrow(s) refer to other regulated isoforms of this gene. Other isoforms are not consistently regulated under the stress. Further information is available in Tables S1, S3 and S6; Figure S31. Structure of previously annotated and new DAS isoforms on XLOC_015679 (Gene 24, AT3G59430) generated due to different multifactorial stress combinations in the transcriptome datasets of 10-d-old *A. thaliana* (wild-type Col-0) seedlings. Red arrow refers to the gene isoform concordantly expressed with a given splicing factor, while green arrow(s) refer to other regulated isoforms of this gene. Other isoforms are not consistently regulated under the stress. Further information is available in Tables S1, S3 and S6; Figure S32. Structure of previously annotated and new DAS isoforms on XLOC_017325 (Gene 25, AT4G29340/ STRG.19084) generated due to different multifactorial stress combinations in the transcriptome datasets of 10-d-old *A. thaliana* (wild-type Col-0) seedlings. Red arrow refers to the gene isoform concordantly expressed with a given splicing factor, while green arrow(s) refer to other regulated isoforms of this gene. Other isoforms are not consistently regulated under the stress. Further information is available in Tables S1, S3 and S6; Figure S33. Structure of previously annotated and new DAS isoforms on XLOC_018080 (Gene 26, STRG.16675) generated due to different multifactorial stress combinations in the transcriptome datasets of 10-d-old *A. thaliana* (wild-type Col-0) seedlings. Red arrow refers to the gene isoform concordantly expressed with a given splicing factor, while green arrow(s) refer to other regulated isoforms of this gene. Other isoforms are not consistently regulated under the stress. Further information is available in Tables S1, S3 and S6; Figure S34. Structure of previously annotated and new DAS isoforms on XLOC_019807 (Gene 27, AT5G01490/AT5G01500/AT5G01520) generated due to different multifactorial stress combinations in the transcriptome datasets of 10-d-old *A. thaliana* (wild-type Col-0) seedlings. Red arrow refers to the gene isoform concordantly expressed with a given splicing factor, while green arrow(s) refer to other regulated isoforms of this gene. Other isoforms are not consistently regulated under the stress. Further information is available in Tables S1, S3 and S6. Note that the three isoforms belong to different genes, thus, the concordantly expressed isoform (e.g., AT5G01490.2) with SF5 (e.g., AT3G46490.2) was not analyzed further; Figure S35. Structure of previously annotated and new DAS isoforms on XLOC_019990 (Gene 28, STRG.20609) generated due to different multifactorial stress combinations in the transcriptome datasets of 10-d-old *A. thaliana* (wild-type Col-0) seedlings. Red arrow refers to the gene isoform concordantly expressed with a given splicing factor, while green arrow(s) refer to other regulated isoforms of this gene. Other isoforms are not consistently regulated under the stress. Further information is available in Tables S1, S3 and S6; Figure S36. Structure of previously annotated and new DAS isoforms on XLOC_020544 (Gene 29, STRG.21767) generated due to different multifactorial stress combinations in the transcriptome datasets of 10-d-old *A. thaliana* (wild-type Col-0) seedlings. Red arrow refers to the gene isoform concordantly expressed with a given splicing factor, while green arrow(s) refer to other regulated isoforms of this gene. Other isoforms are not consistently regulated under the stress. Further information is available in Tables S1, S3 and S6; Figure S37. Structure of previously annotated and new DAS isoforms on XLOC_021961 (Gene 30, AT5G53120) generated due to different multifactorial stress combinations in the transcriptome datasets of 10-d-old *A. thaliana* (wild-type Col-0) seedlings. Red arrow refers to the gene isoform concordantly expressed with a given splicing factor, while green arrow(s) refer to other regulated isoforms of this gene. Other isoforms are not consistently regulated under the stress. Further information is available in Tables S1, S3 and S6; Figure S38. Structure of previously annotated and new DAS isoforms on XLOC_022121 (Gene 31, STRG.24929) generated due to different multifactorial stress combinations in the transcriptome datasets of 10-d-old *A. thaliana* (wild-type Col-0) seedlings. Red arrow refers to the gene isoform concordantly expressed with a given splicing factor, while green arrow(s) refer to other regulated isoforms of this gene. Other isoforms are not consistently regulated under the stress. Further information is available in Tables S1, S3 and S6; Figure S39. Structure of previously annotated and new DAS isoforms on XLOC_022555 (Gene 32, STRG.25829) generated due to different multifactorial stress combinations in the transcriptome datasets of 10-d-old *A. thaliana* (wild-type Col-0) seedlings. Red arrow refers to the gene isoform concordantly expressed with a given splicing factor, while green arrow(s) refer to other regulated isoforms of this gene. Other isoforms are not consistently regulated under the stress. Further information is available in Tables S1, S3 and S6; Figure S40. Structure of previously annotated and new DAS isoforms on XLOC_023309 (Gene 33, AT5G14020) generated due to different multifactorial stress combinations in the transcriptome datasets of 10-d-old *A. thaliana* (wild-type Col-0) seedlings. Red arrow refers to the gene isoform concordantly expressed with a given splicing factor, while green arrow(s) refer to other regulated isoforms of this gene. Other isoforms are not consistently regulated under the stress. Further information is available in Tables S1, S3 and S6; Figure S41. Structure of previously annotated and new DAS isoforms on XLOC_025093 (Gene 34, STRG.25124) generated due to different multifactorial stress combinations in the transcriptome datasets of 10-d-old *A. thaliana* (wild-type Col-0) seedlings. Red arrow refers to the gene isoform concordantly expressed with a given splicing factor, while green arrow(s) refer to other regulated isoforms of this gene. Other isoforms are not consistently regulated under the stress. Further information is available in Tables S1, S3 and S6; Figure S42. Structure of previously annotated and new DAS isoforms on XLOC_022555 (Gene 35, STRG.26050) generated due to different multifactorial stress combinations in the transcriptome datasets of 10-d-old *A. thaliana* (wild-type Col-0) seedlings. Red arrow refers to the gene isoform concordantly expressed with a given splicing factor, while green arrow(s) refer to other regulated isoforms of this gene. Other isoforms are not consistently regulated under the stress. Further information is available in Tables S1, S3 and S6; Figure S43. Multiple sequence alignment at the DNA level for annotated and new isoforms of *A. thaliana* locus XLOC_008527 generated under different multifactorial stress combinations where isoforms AT2G43500.11 and STRG.10463.9 showed expression pattern HL↑, isoform STRG. 10463.14 showed expression pattern all stress combinations↑, while expression of isoforms AT2G43500.9 and AT2G43500.10 was arbitrary. H = heat stress, L = high light stress; Figure S44. Multiple sequence alignment at the amino acid level for annotated and new isoforms of *A. thaliana* locus XLOC_008527 generated under different multifactorial stress combinations where isoforms AT2G43500.11 and STRG.10463.9 showed expression pattern HL↑, isoform STRG. 10463.14 showed expression pattern all stress combinations↑, while expression of isoforms AT2G43500.9 and AT2G43500.10 was arbitrary. H = heat stress, L = high light stress. The figure emphasizes Exon 1 alignment as referred to in Figure S20; Figure S45. Multiple sequence alignment at the amino acid level for annotated and new isoforms of *A. thaliana* locus XLOC_008527 generated under different multifactorial stress combinations where isoforms AT2G43500.11 and STRG.10463.9 showed expression pattern HL↑, isoform STRG.10463.14 showed expression pattern all stress combinations↑, while expression of isoforms AT2G43500.9 and AT2G43500.10 was arbitrary. H = heat stress, L = high light stress. The figure emphasizes Exon 2 alignment as referred to in Figure S20; Figure S46. Multiple sequence alignment at the amino acid level for annotated and new isoforms of *A. thaliana* locus XLOC_008527 generated under different multifactorial stress combinations where isoforms AT2G43500.11 and STRG.10463.9 showed expression pattern HL↑, isoform STRG.10463.14 showed expression pattern all stress combinations↑, while expression of isoforms AT2G43500.9 and AT2G43500.10 was arbitrary. H = heat stress, L = high light stress. The figure emphasizes Exons 3 and 4 alignment as referred to in Figure S20; Figure S47. Multiple sequence alignment at the amino acid level for annotated and new isoforms of *A. thaliana* locus XLOC_008527 generated under different multifactorial stress combinations where isoforms AT2G43500.11 and STRG.10463.9 showed expression pattern HL↑, isoform STRG.10463.14 showed expression pattern all stress combinations↑, while expression of isoforms AT2G43500.9 and AT2G43500.10 was arbitrary. H = heat stress, L = high light stress. The figure emphasizes Exons 5/6/7/8 alignment as referred to in Figure S20; Figure S48. Position of the active conserved motifs RWP-RK (pfam02042) and PB1-NLP (cd06407) of the ABC transporter B family member 11 protein encoded by the *A. thaliana* gene isoform AT2G43500.11 located on locus XLOC_008527. Structure and function of these two motifs were previously described [46,47,48,49]. Further information on the structure of this gene isoform is available in Figure 5 and Figure S20; Figure S49. Expression profiling of isoforms on the two loci XLOC_021339 and XLOC_017971 involving the genes encoding 1-aminocyclopropane-1-carboxylate oxidase 10 (ACO10) (AT5G40910) of cluster 967 and S-adenosyl-methionine (SAM) synthetase 2 (SAM synthetase 2) (AT4G01850) of cluster 1414, respectively, generated from transcriptome datasets of 10-d-old *A. thaliana* (wild-type Col-0) seedlings exposed to different multifactorial stress combinations. C = control (0 mM NaCl, 21 °C, 50 µmol m^−2^ s^−1^), S = salt stress (50 mM NaCl, 21 °C, 50 µmol m^−2^ s^−1^), H = heat stress (0 mM NaCl, 33 °C, 50 µmol m^−2^ s^−1^), L = high light stress (0 mM NaCl, 21 °C, 700 µmol m^−2^ s^−1^); Figure S50. Splicing patterns of the two isoforms of locus XLOC_021339 with the gene (AT5G40910) encoding 1-aminocyclopropane-1-carboxylate oxidase 10 (ACO10). These two isoforms were alternatively spliced with intron retention as the sole splicing type. Splicing patterns of the two splicing isoforms were detected from the following ensemble links: https://ensembl.gramene.org/Arabidopsis_thaliana/Transcript/Summary?db=core;g=AT5G40910;r=5:16394502-16399129;t=AT5G40910.4, https://ensembl.gramene.org/Arabidopsis_thaliana/Transcript/Summary?db=core;g=AT5G40910;r=5:16394502-16399129;t=AT5G40910.6 (accessed on 1 April 2024).
