# Supplementary material for: Concordant Gene Expression and Alternative Splicing Regulation under Abiotic Stresses in Arabidopsis
Source: Genes (Basel). 2024 May 23;15(6):675. doi: 10.3390/genes15060675 (PMC11202685; doi:10.3390/genes15060675)
Supplement: Supplementary file 1 [file genes-15-00675-s001.zip › genes-2990598-Supplementary Figures-new.pdf]

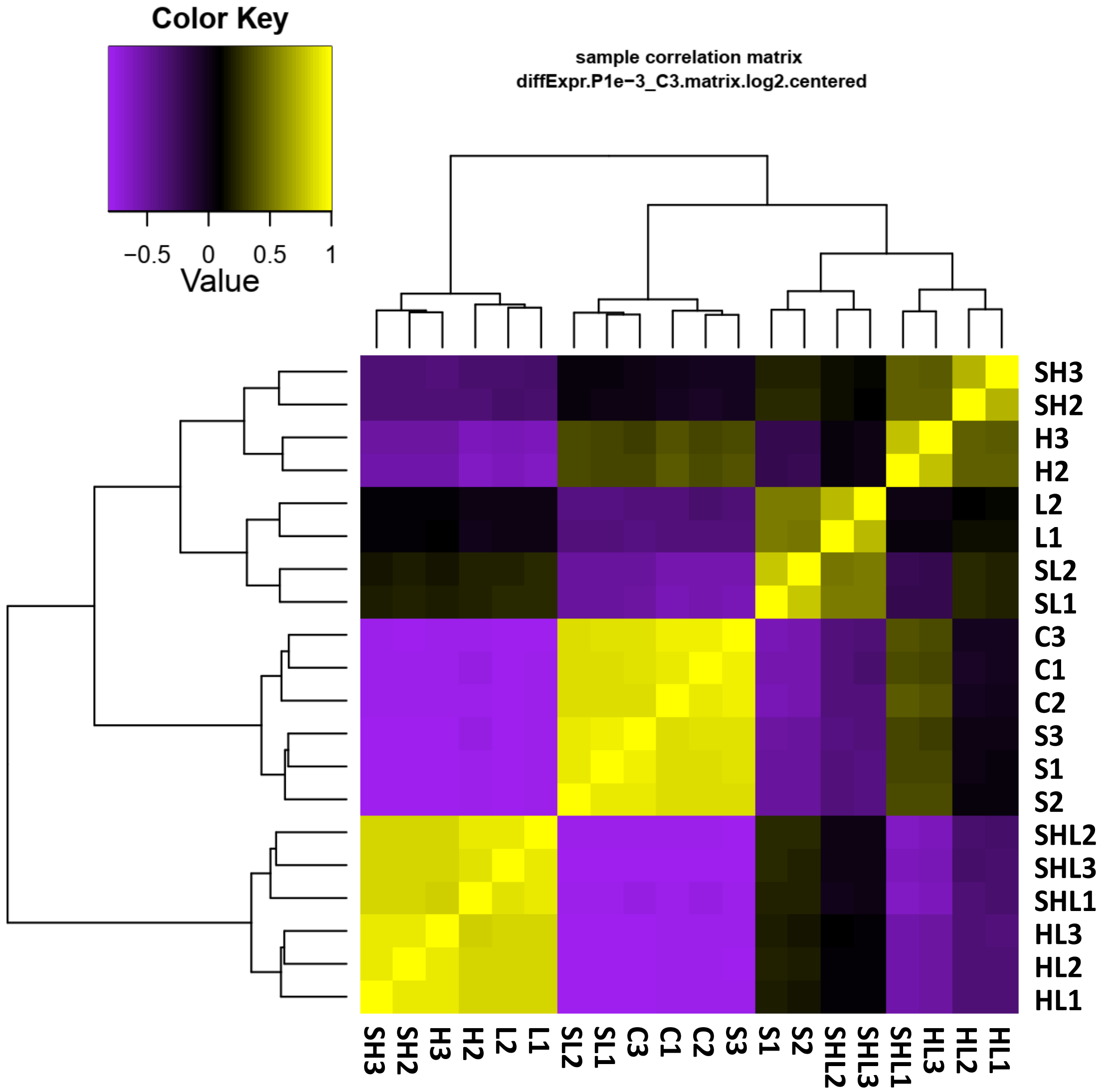

Figure S1. Heatmap referring to 2-D hierarchical clusters of gene expression generated from transcriptome datasets of 10-d-old *Arabidopsis thaliana* (wild-type Col-0) seedlings exposed to different multifactorial stress combinations. C = control (0 mM NaCl, 21°C, 50 µmol m^{-2} s^{-1}), S = salt stress (50 mM NaCl, 21°C, 50 µmol m^{-2} s^{-1}), H = heat stress (0 mM NaCl, 33°C, 50 µmol m^{-2} s^{-1}), L = high light stress (0 mM NaCl, 21°C, 700 µmol m^{-2} s^{-1}). Further information is available in Table S1.

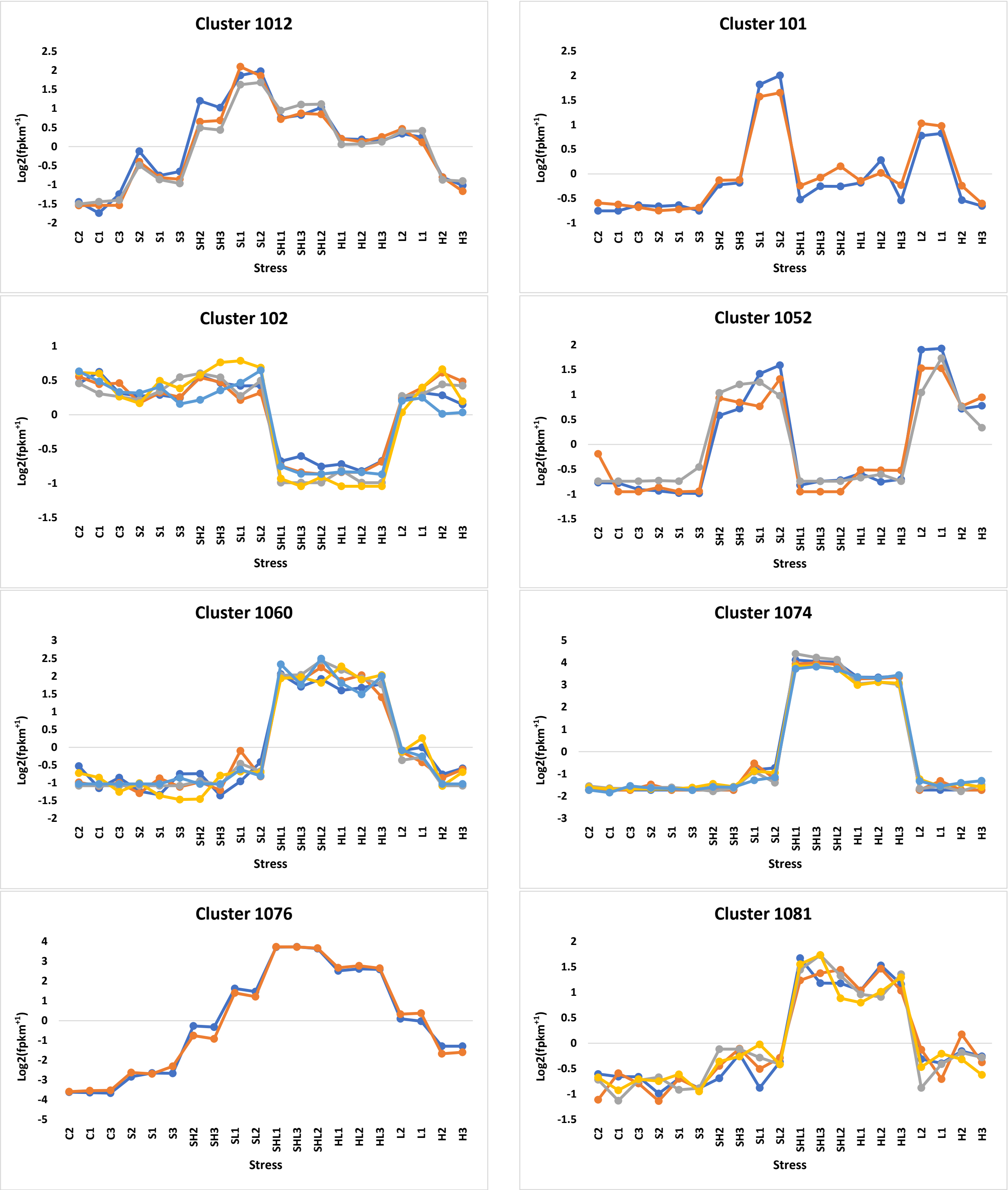

Figure S2. Expression profiling of the most consistent expression patterns generated from transcriptome datasets of 10-d-old *A. thaliana* (wild-type Col-0) seedlings exposed to different multifactorial stress combinations. C = control (0 mM NaCl, 21°C, 50 µmol m^{-2} s^{-1}), S = salt stress (50 mM NaCl, 21°C, 50 µmol m^{-2} s^{-1}), H = heat stress (0 mM NaCl, 33°C, 50 µmol m^{-2} s^{-1}), L = high light stress (0 mM NaCl, 21°C, 700 µmol m^{-2} s^{-1}). Further growth and abiotic stress conditions were recently reported (Zandalinas et al., 2021). Sequences can be found in Bioproject PRJNA622644. Detailed information of all gene clusters are shown in Table S1.

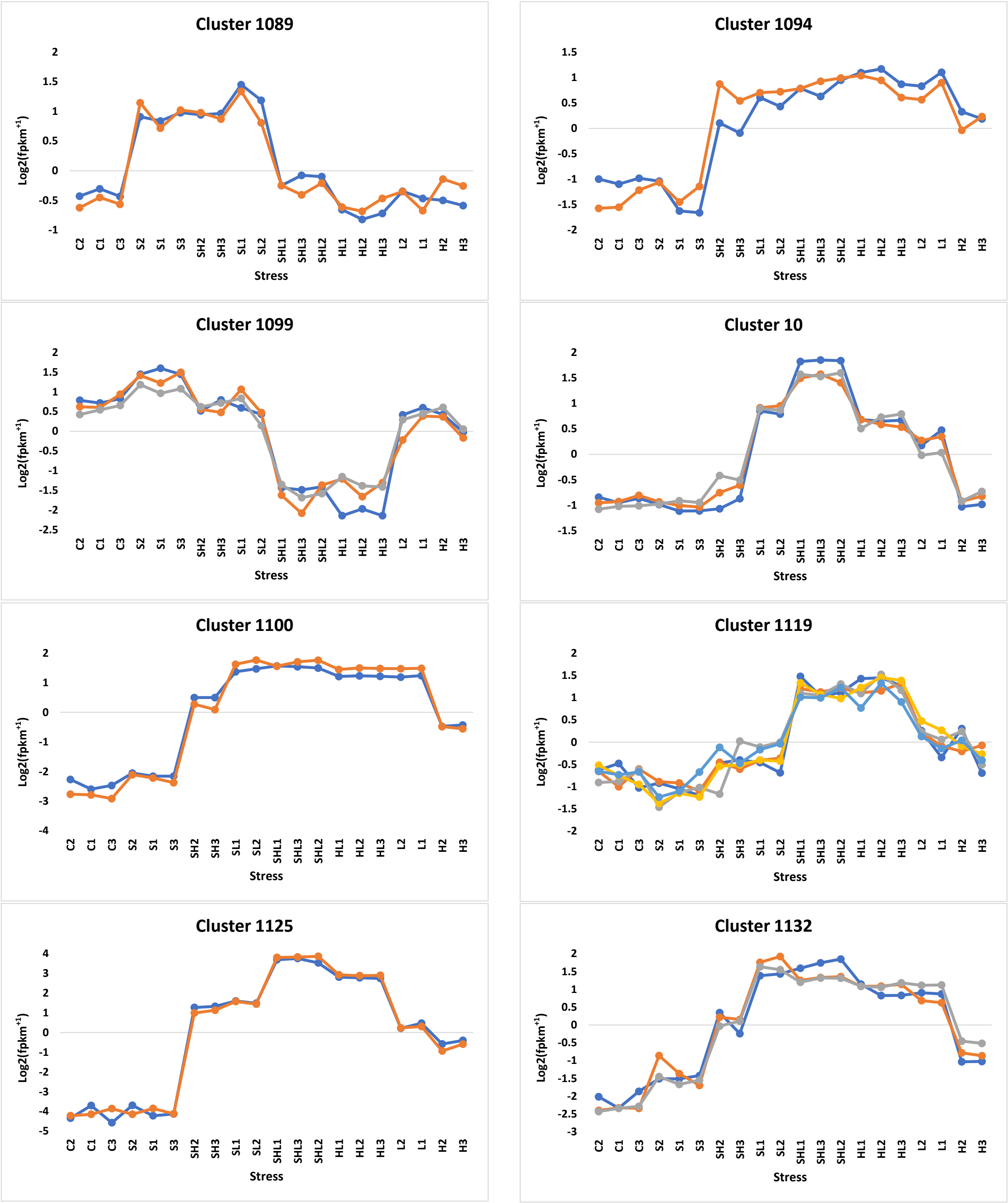

Figure S2. Continued

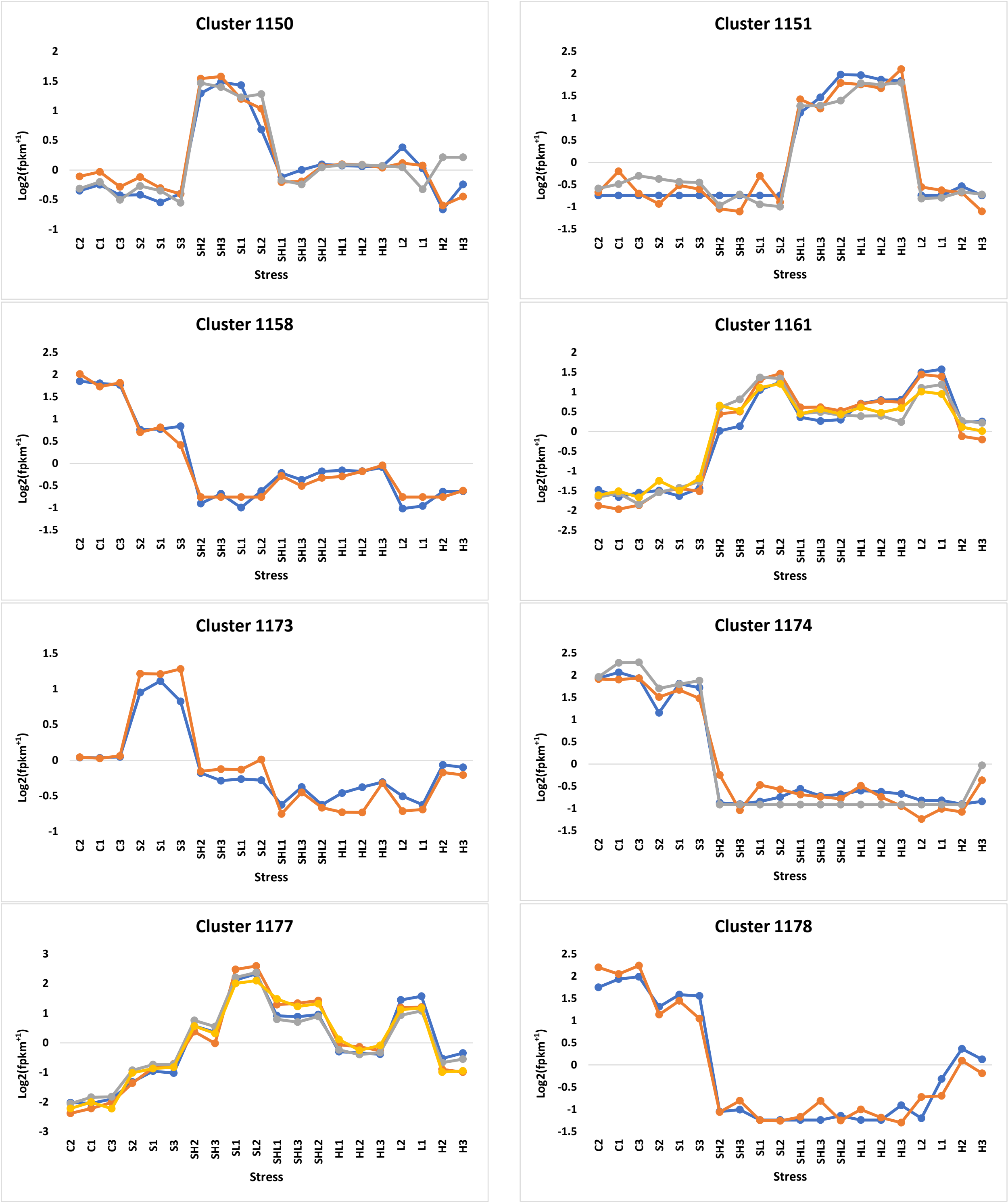

Figure S2. Continued

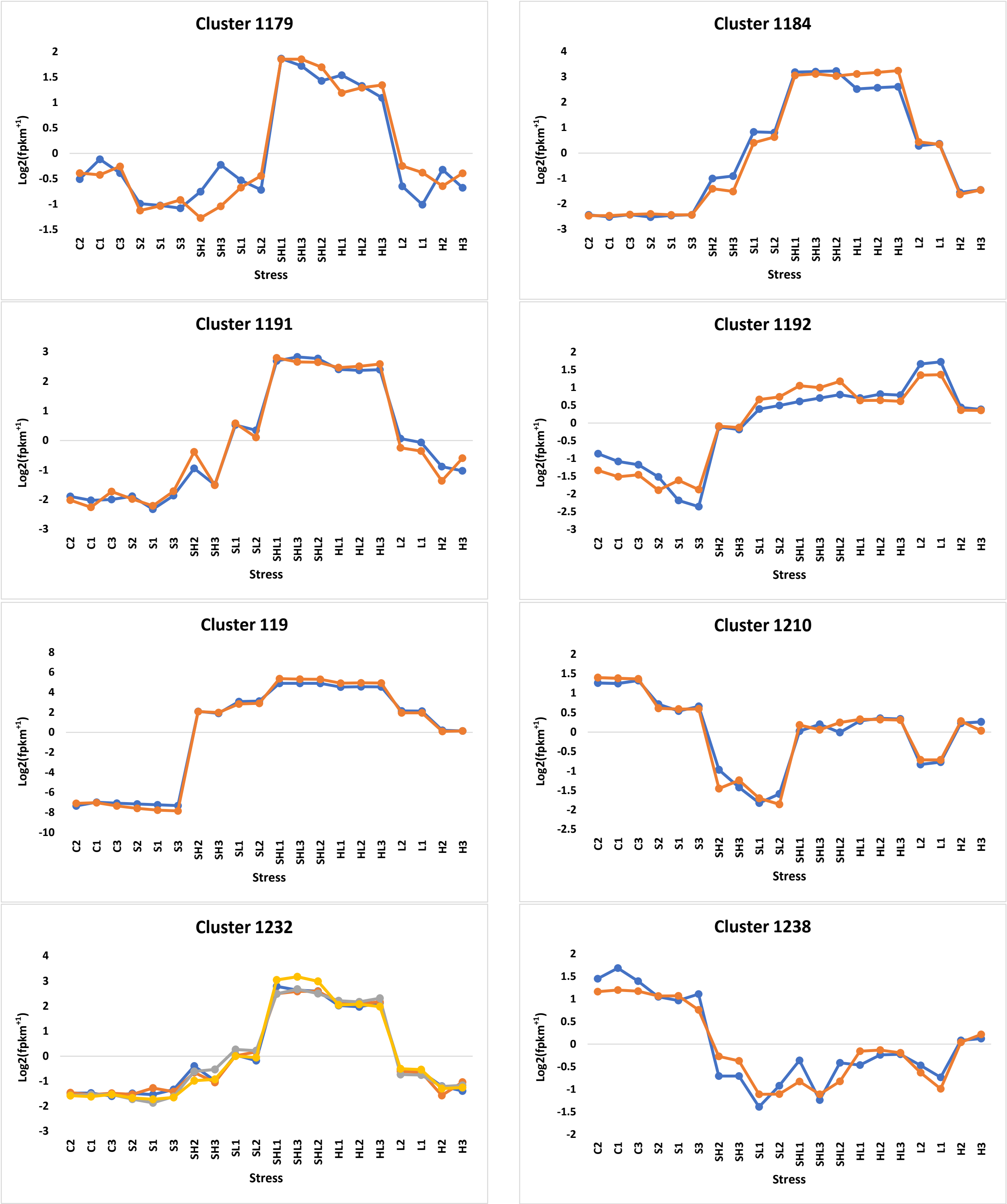

Figure S2. Continued

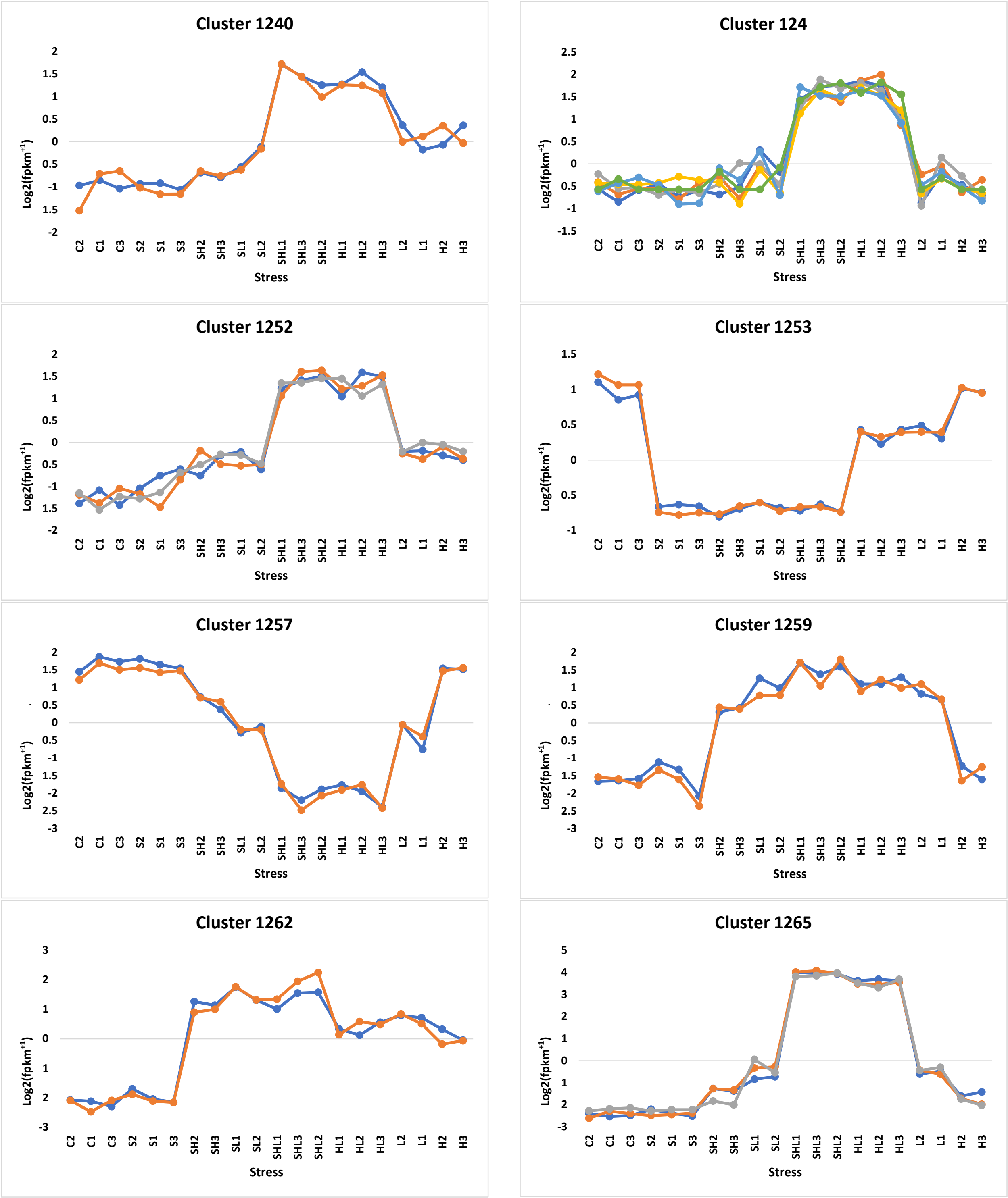

Figure S2. Continued

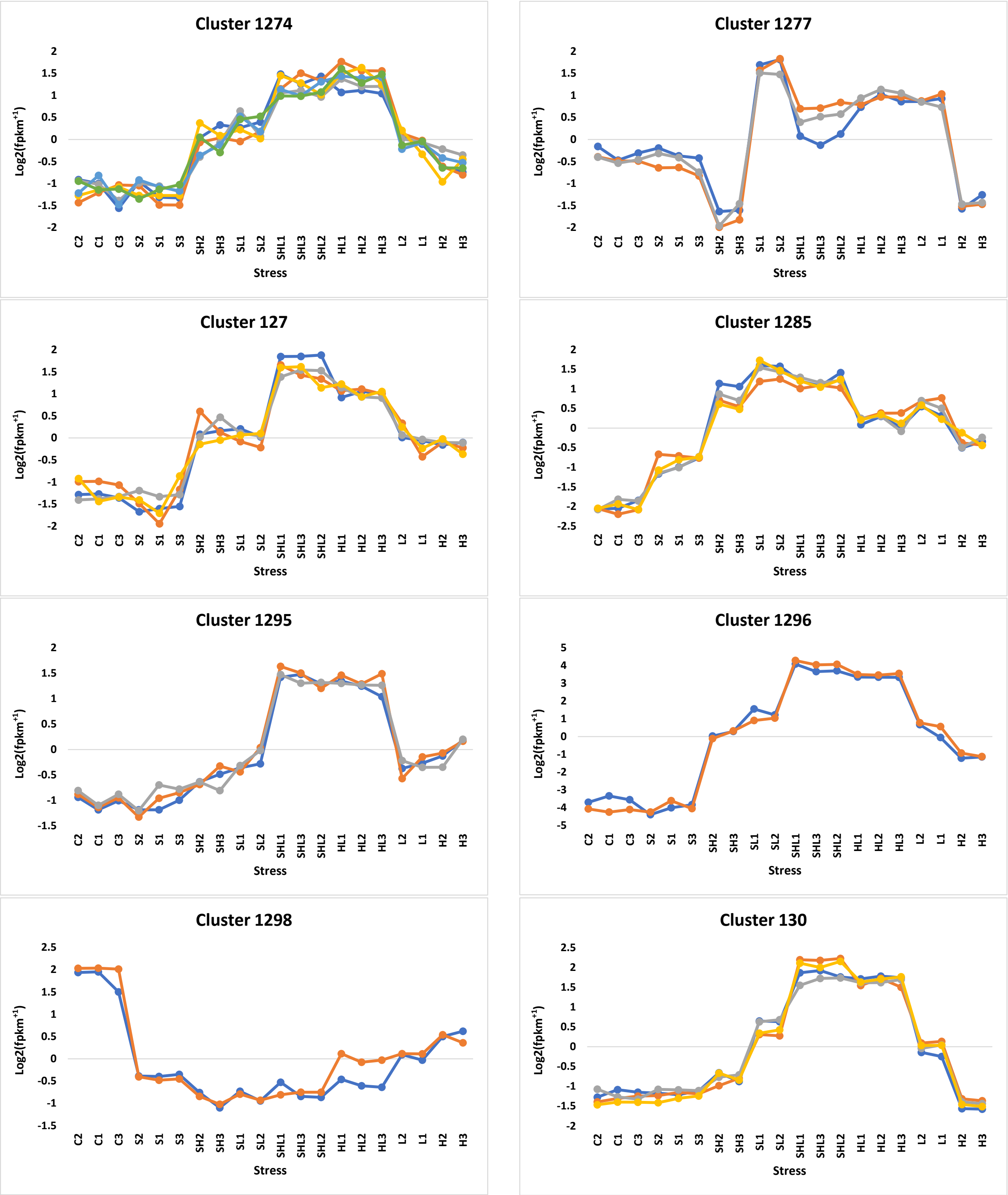

Figure S2. Continued

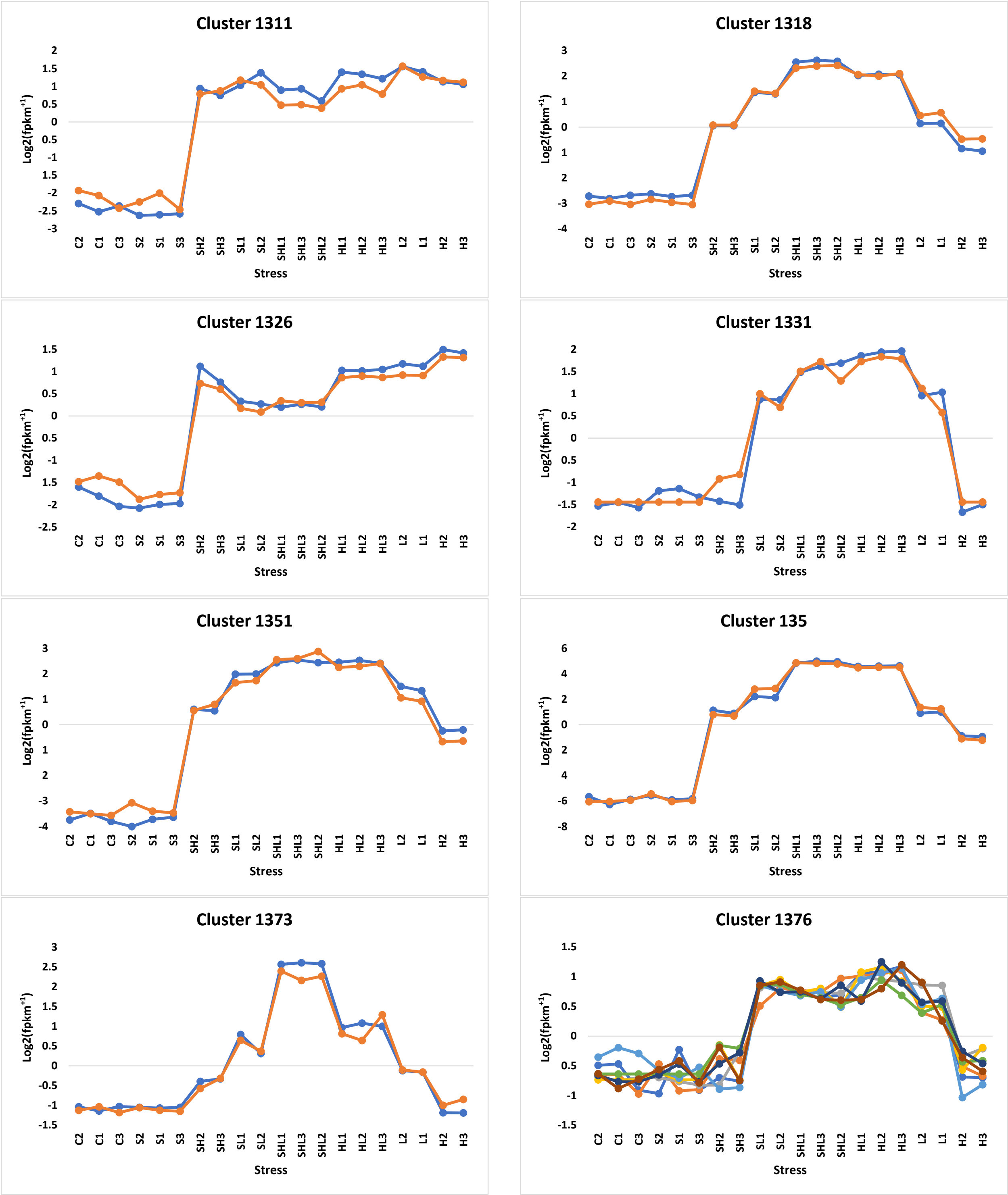

Figure S2. Continued

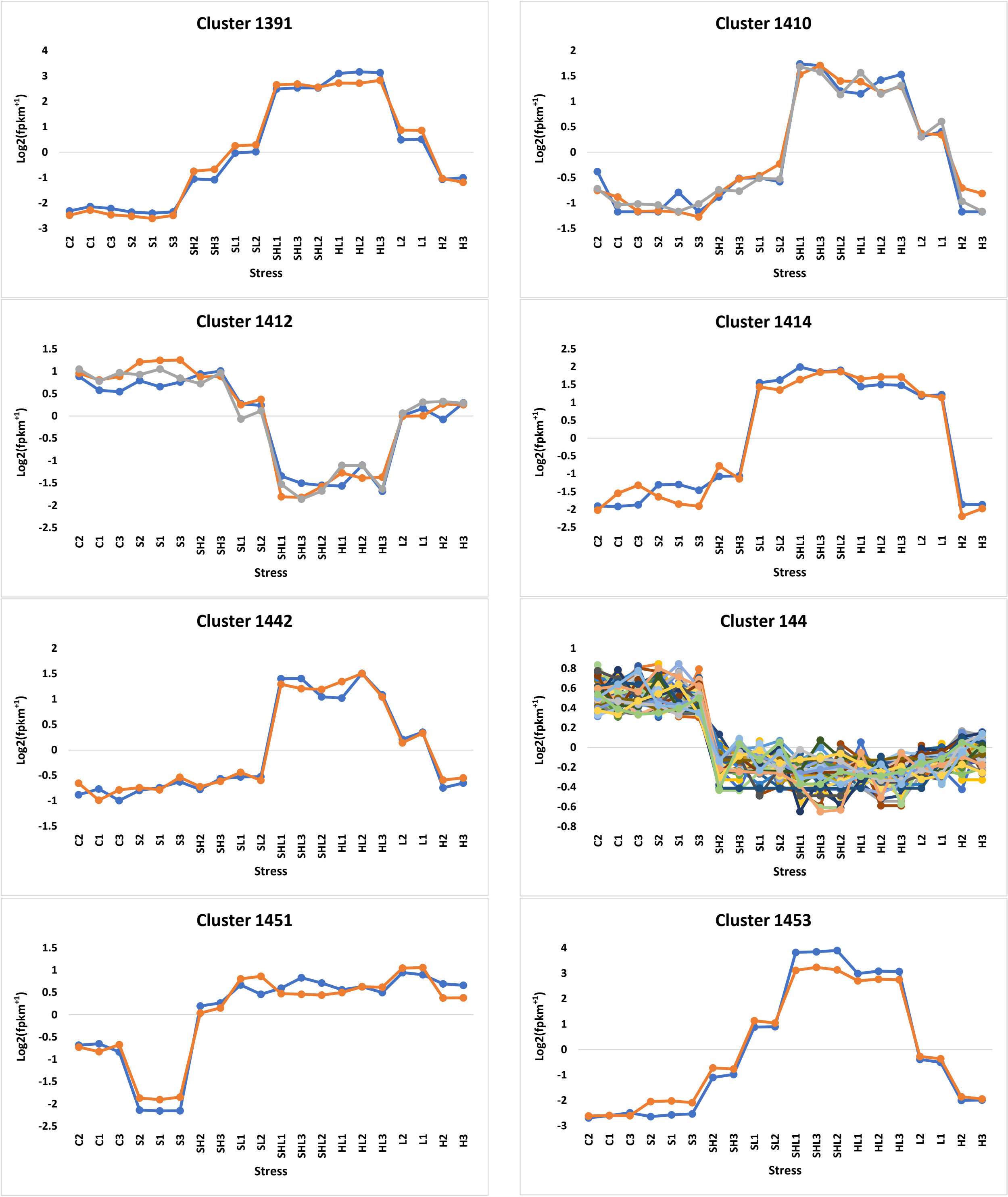

Figure S2. Continued

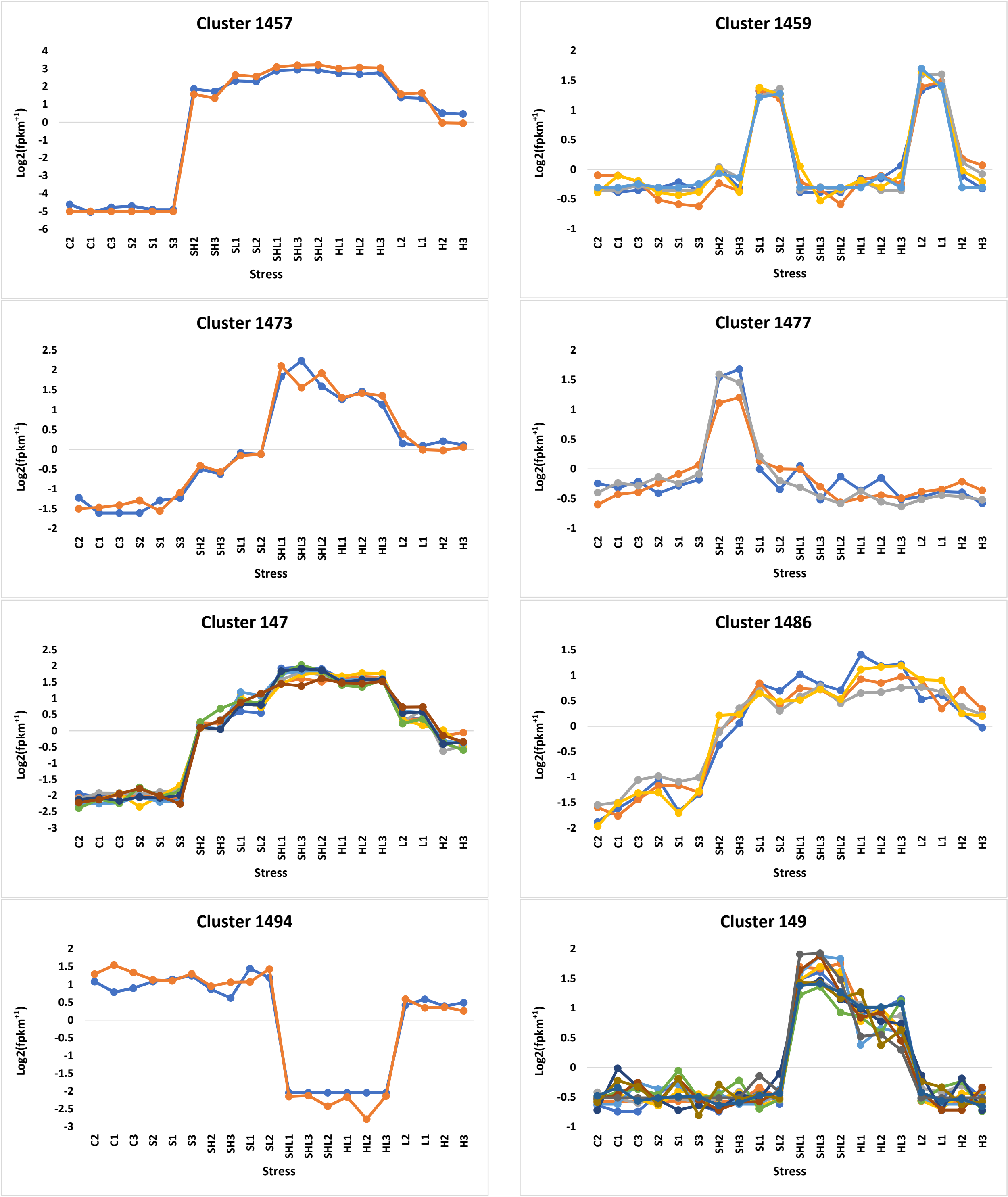

Figure S2. Continued

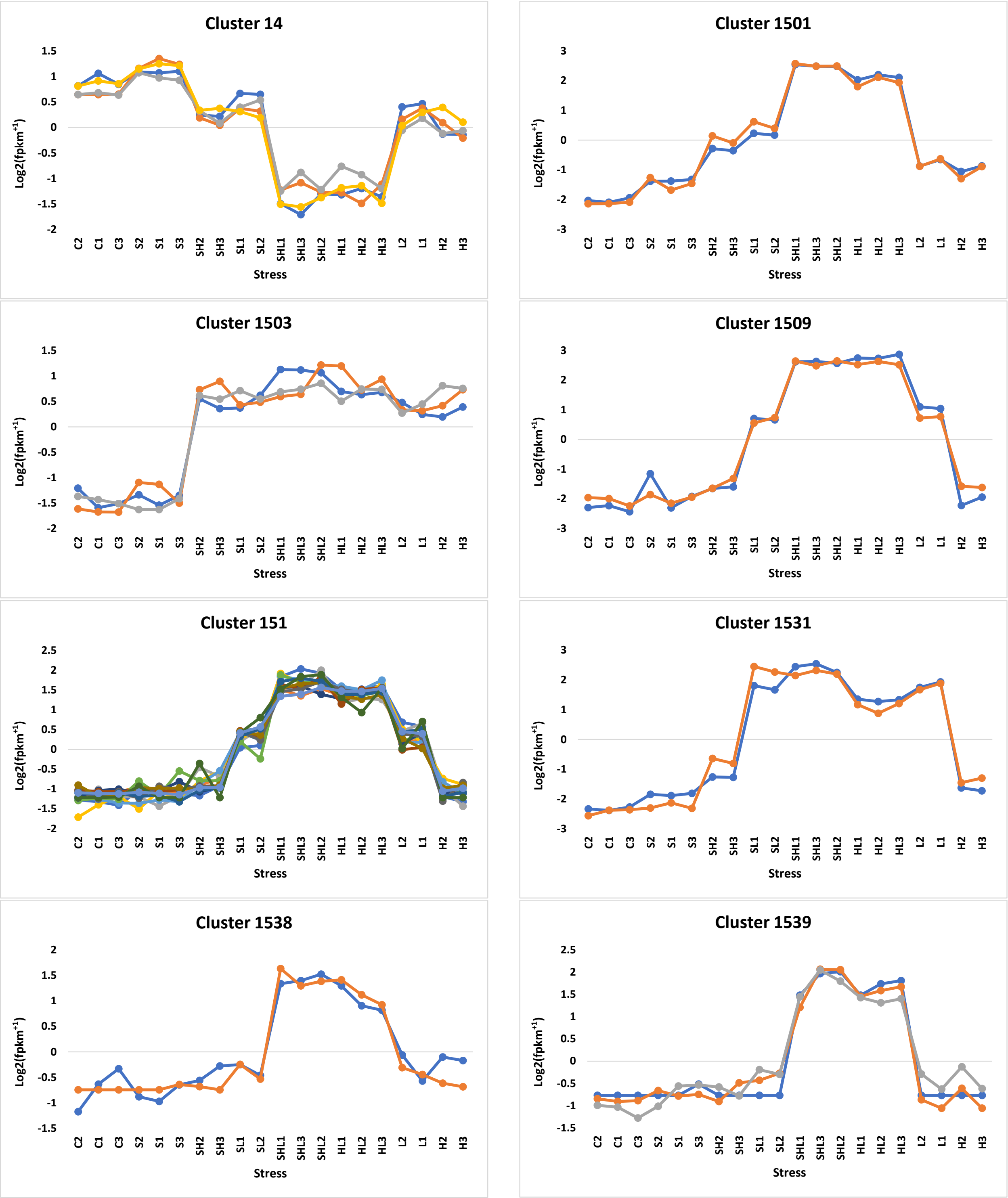

Figure S2. Continued

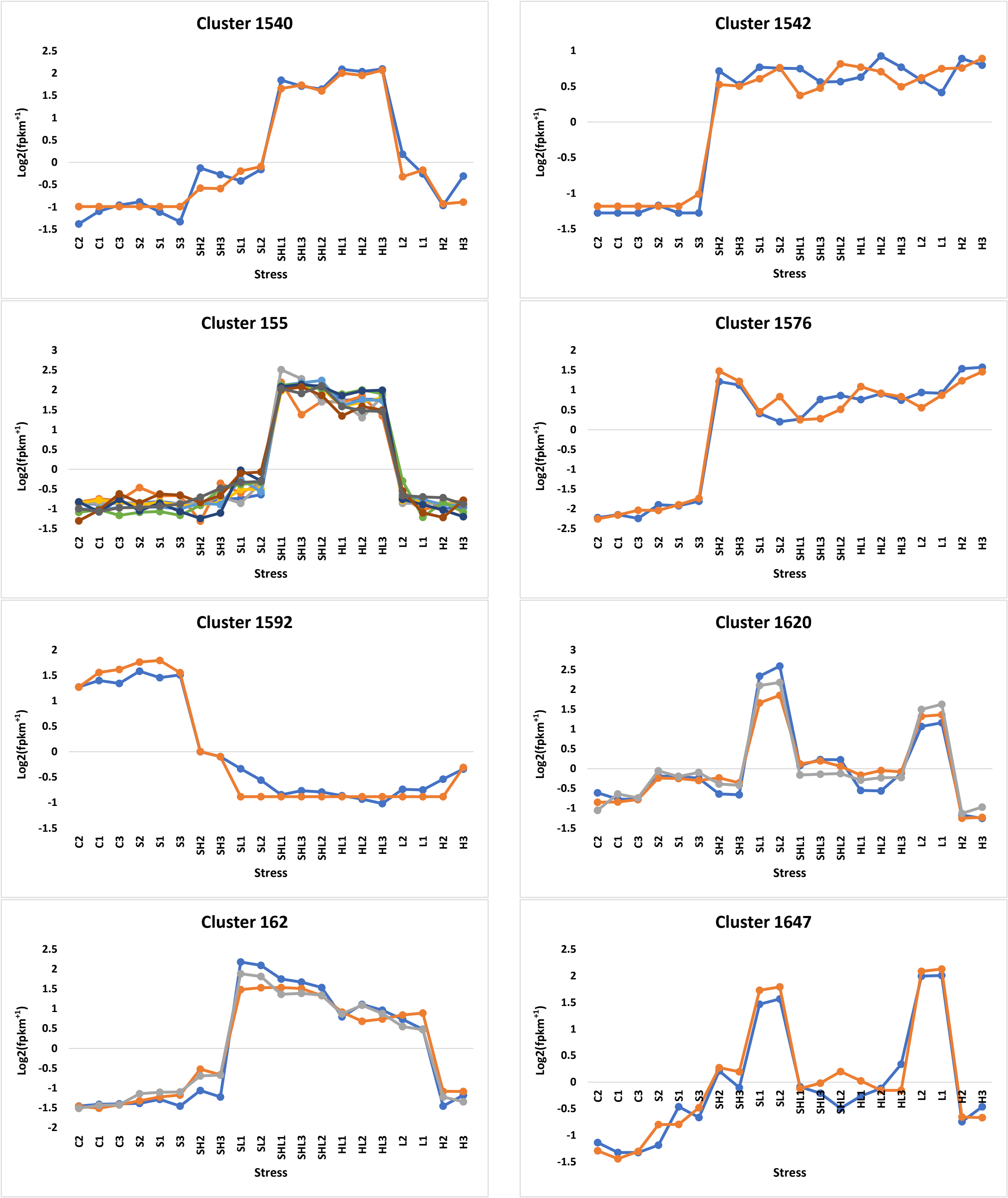

Figure S2. Continued

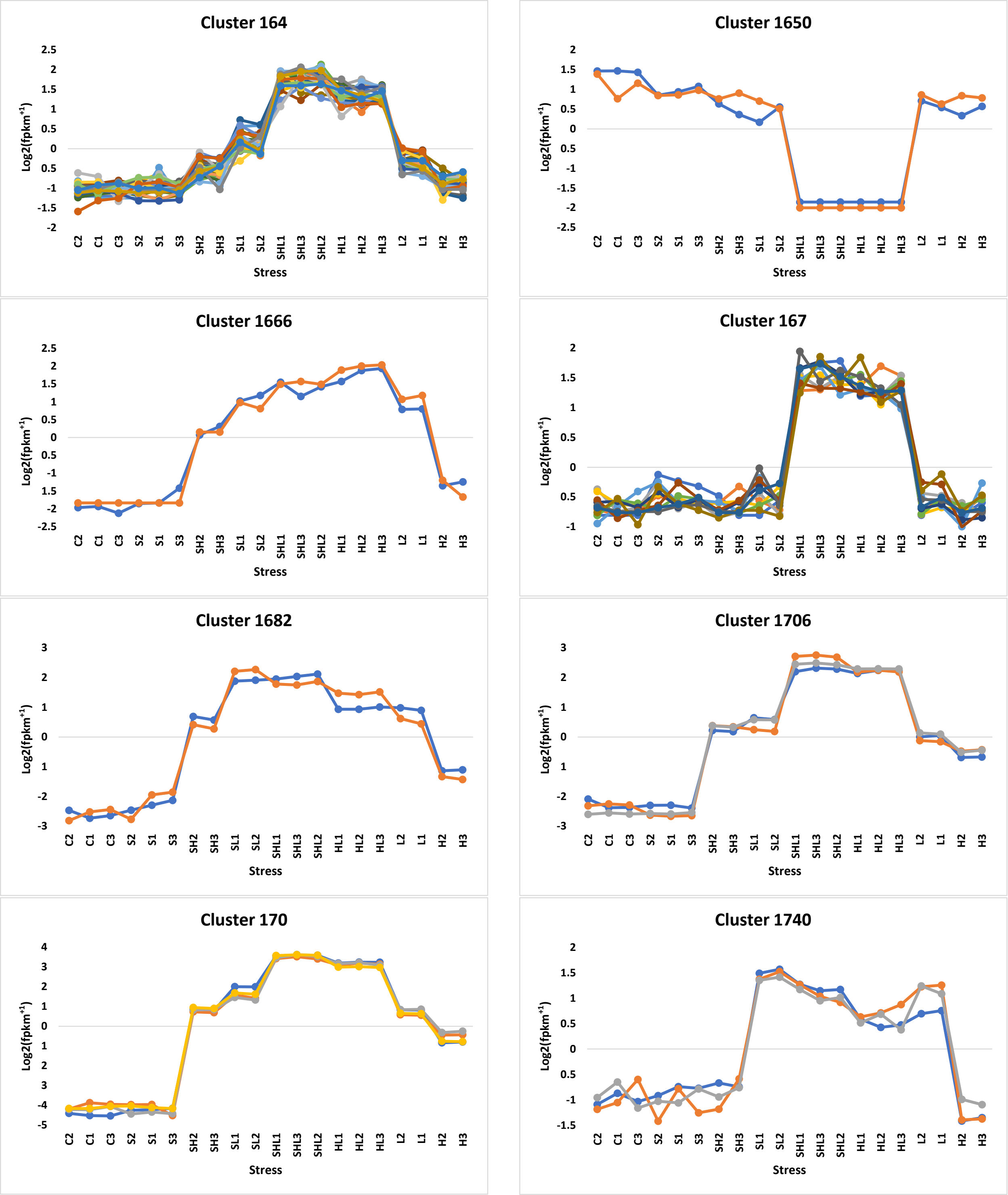

Figure S2. Continued

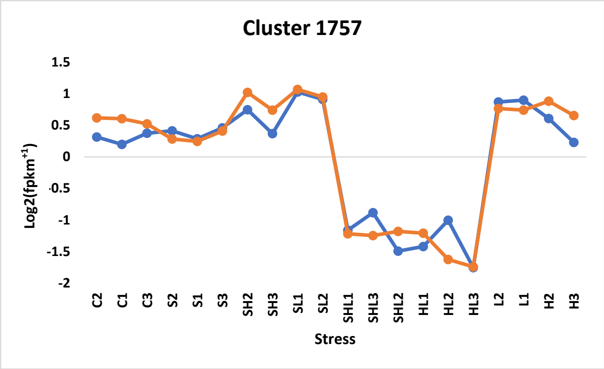
Cluster 1757
1.5 1 0.5 0 -0.5 -1 -1.5 -2
Log2(fpkm+1)
C2 C1 C3 S2 S1 S3 SH2 SH3 SL1 SL2 SHL1 SHL3 SHL2 HL1 HL2 HL3 L2 L1 H2 H3
Stress

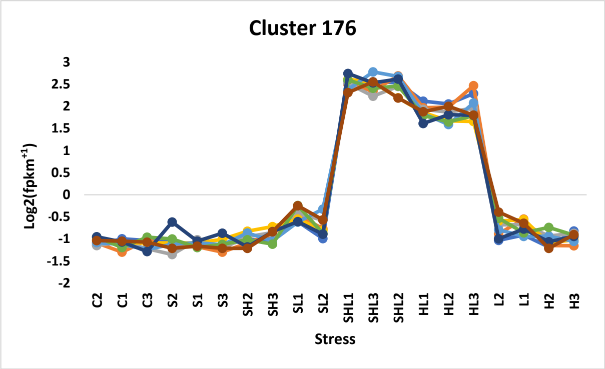
Cluster 176
3 2.5 2 1.5 1 0.5 0 -0.5 -1 -1.5 -2
Log2(fpkm+1)
C2 C1 C3 S2 S1 S3 SH2 SH3 SL1 SL2 SHL1 SHL3 SHL2 HL1 HL2 HL3 L2 L1 H2 H3
Stress

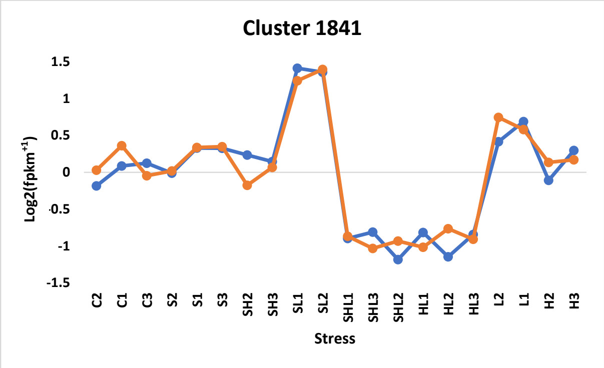
Cluster 1841
1.5 1 0.5 0 -0.5 -1 -1.5
Log2(fpkm+1)
C2 C1 C3 S2 S1 S3 SH2 SH3 SL1 SL2 SHL1 SHL3 SHL2 HL1 HL2 HL3 L2 L1 H2 H3
Stress

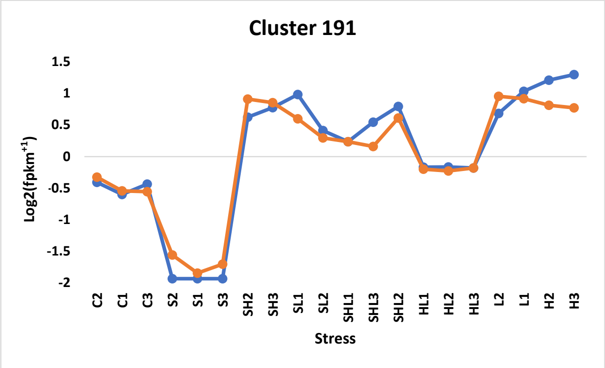
Cluster 191
1.5 1 0.5 0 -0.5 -1 -1.5 -2
Log2(fpkm+1)
C2 C1 C3 S2 S1 S3 SH2 SH3 SL1 SL2 SHL1 SHL3 SHL2 HL1 HL2 HL3 L2 L1 H2 H3
Stress

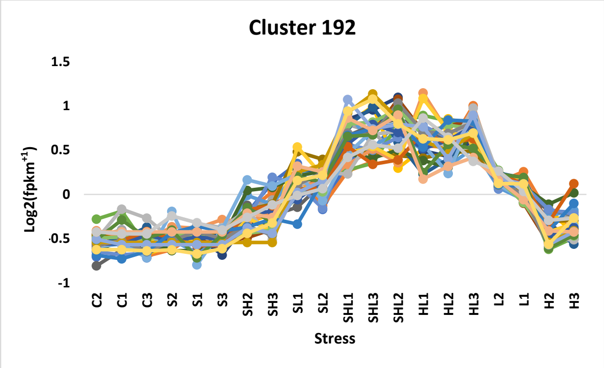
Cluster 192
1.5 1 0.5 0 -0.5 -1
Log2(fpkm+1)
C2 C1 C3 S2 S1 S3 SH2 SH3 SL1 SL2 SHL1 SHL3 SHL2 HL1 HL2 HL3 L2 L1 H2 H3
Stress

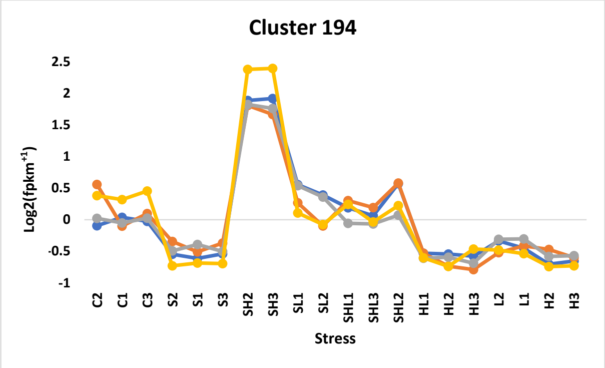
Cluster 194
2.5 2 1.5 1 0.5 0 -0.5 -1
Log2(fpkm+1)
C2 C1 C3 S2 S1 S3 SH2 SH3 SL1 SL2 SHL1 SHL3 SHL2 HL1 HL2 HL3 L2 L1 H2 H3
Stress

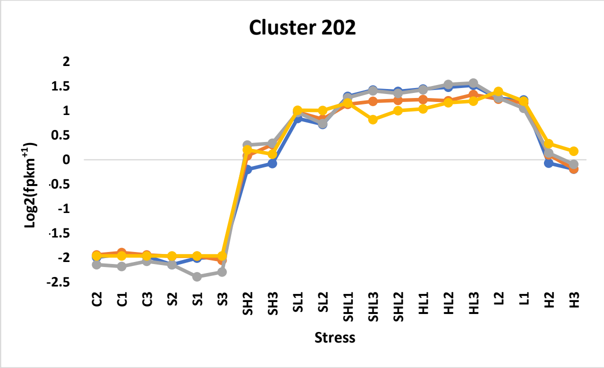
Cluster 202
2 1.5 1 0.5 0 -0.5 -1 -1.5 -2 -2.5
Log2(fpkm+1)
C2 C1 C3 S2 S1 S3 SH2 SH3 SL1 SL2 SHL1 SHL3 SHL2 HL1 HL2 HL3 L2 L1 H2 H3
Stress

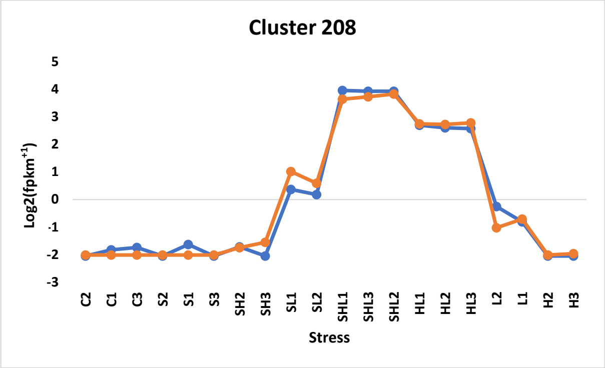
Cluster 208
5 4 3 2 1 0 -1 -2 -3
Log2(fpkm+1)
C2 C1 C3 S2 S1 S3 SH2 SH3 SL1 SL2 SHL1 SHL3 SHL2 HL1 HL2 HL3 L2 L1 H2 H3
Stress

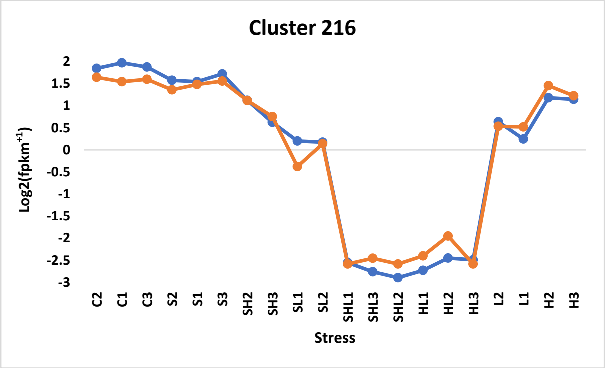
Cluster 216
Log2(fpkm+1)
2 1.5 1 0.5 0 -0.5 -1 -1.5 -2 -2.5 -3
C2 C1 C3 S2 S1 S3 SH2 SH3 SL1 SL2 SHL1 SHL3 SHL2 HL1 HL2 HL3 L2 L1 H2 H3
Stress

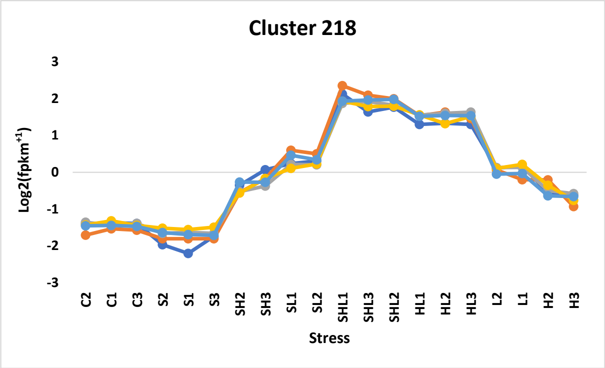
Cluster 218
Log2(fpkm+1)
3 2 1 0 -1 -2 -3
C2 C1 C3 S2 S1 S3 SH2 SH3 SL1 SL2 SHL1 SHL3 SHL2 HL1 HL2 HL3 L2 L1 H2 H3
Stress

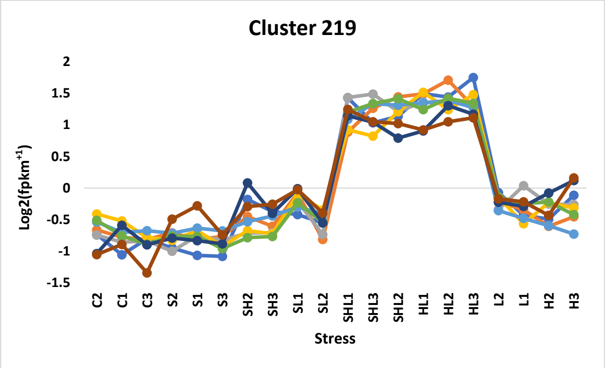
Cluster 219
Log2(fpkm+1)
2 1.5 1 0.5 0 -0.5 -1 -1.5
C2 C1 C3 S2 S1 S3 SH2 SH3 SL1 SL2 SHL1 SHL3 SHL2 HL1 HL2 HL3 L2 L1 H2 H3
Stress

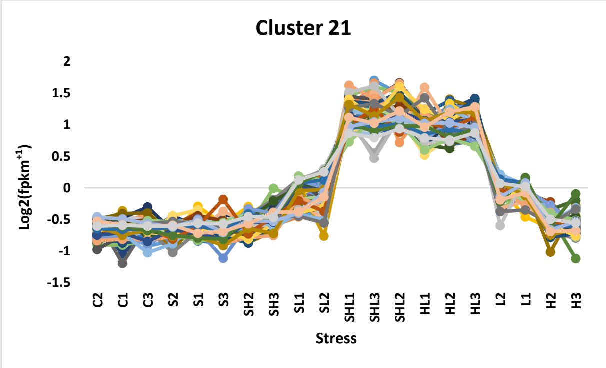
Cluster 21
Log2(fpkm+1)
2 1.5 1 0.5 0 -0.5 -1 -1.5
C2 C1 C3 S2 S1 S3 SH2 SH3 SL1 SL2 SHL1 SHL3 SHL2 HL1 HL2 HL3 L2 L1 H2 H3
Stress

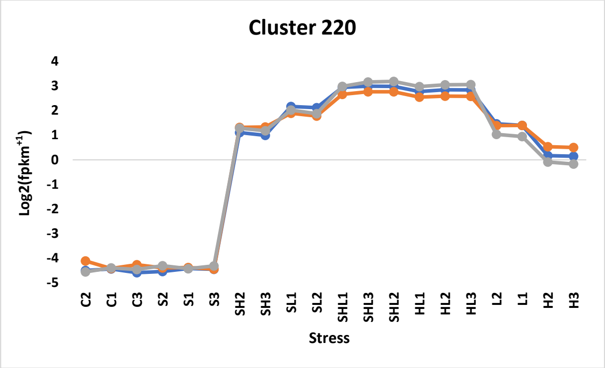
Cluster 220
Log2(fpkm+1)
4 3 2 1 0 -1 -2 -3 -4 -5
C2 C1 C3 S2 S1 S3 SH2 SH3 SL1 SL2 SHL1 SHL3 SHL2 HL1 HL2 HL3 L2 L1 H2 H3
Stress

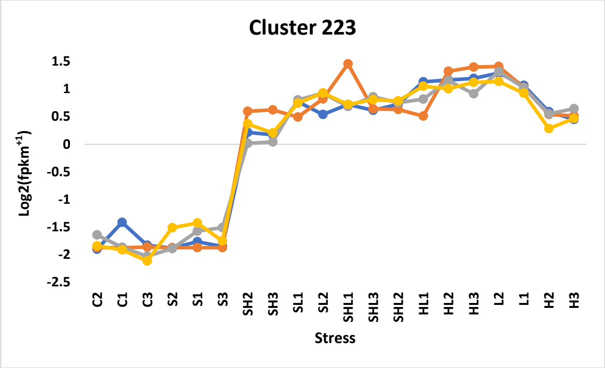
Cluster 223
Log2(fpkm+1)
1.5 1 0.5 0 -0.5 -1 -1.5 -2 -2.5
C2 C1 C3 S2 S1 S3 SH2 SH3 SL1 SL2 SHL1 SHL3 SHL2 HL1 HL2 HL3 L2 L1 H2 H3
Stress

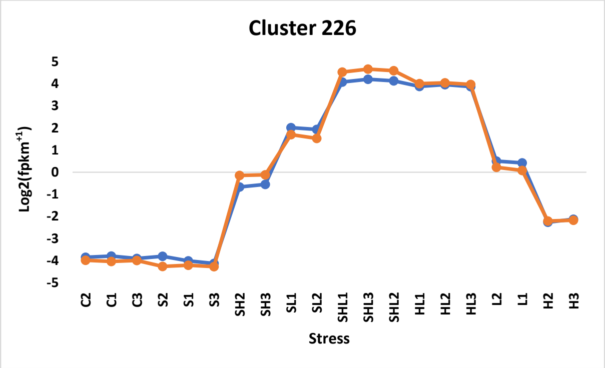
Cluster 226
Log2(fpkm+1)
5 4 3 2 1 0 -1 -2 -3 -4 -5
C2 C1 C3 S2 S1 S3 SH2 SH3 SL1 SL2 SHL1 SHL3 SHL2 HL1 HL2 HL3 L2 L1 H2 H3
Stress

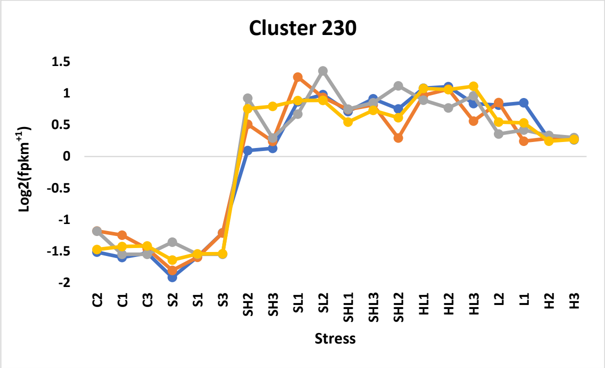
Cluster 230
Log2(fpkm+1)
1.5 1 0.5 0 -0.5 -1 -1.5 -2
C2 C1 C3 S2 S1 S3 SH2 SH3 SL1 SL2 SHL1 SHL3 SHL2 HL1 HL2 HL3 L2 L1 H2 H3
Stress

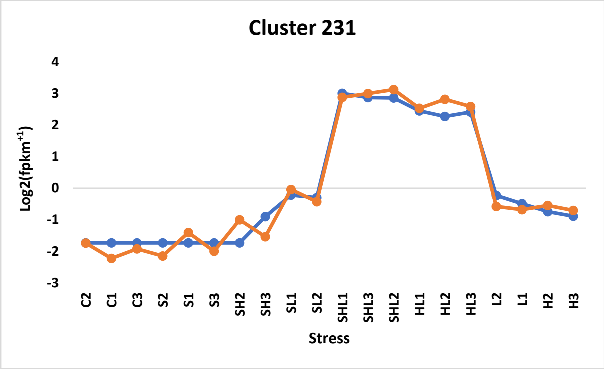
Cluster 231
Log2(fpkm+1)
Stress
C2 C1 C3 S2 S1 S3 SH2 SH3 SL1 SL2 SHL1 SHL3 SHL2 HL1 HL2 HL3 L2 L1 H2 H3

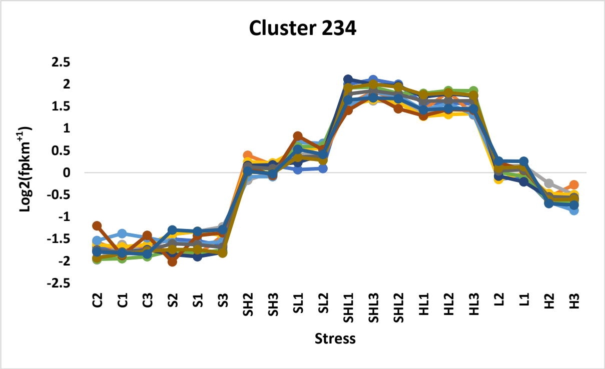
Cluster 234
Log2(fpkm+1)
Stress
C2 C1 C3 S2 S1 S3 SH2 SH3 SL1 SL2 SHL1 SHL3 SHL2 HL1 HL2 HL3 L2 L1 H2 H3

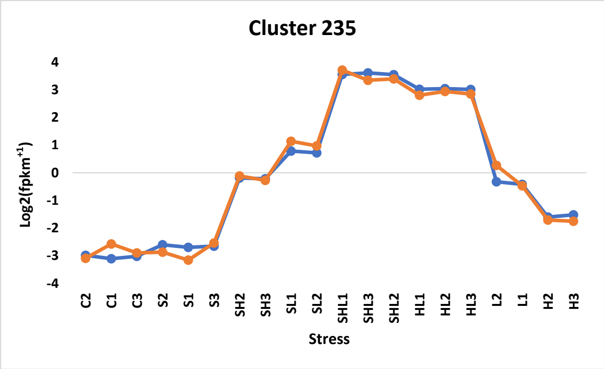
Cluster 235
Log2(fpkm+1)
Stress
C2 C1 C3 S2 S1 S3 SH2 SH3 SL1 SL2 SHL1 SHL3 SHL2 HL1 HL2 HL3 L2 L1 H2 H3

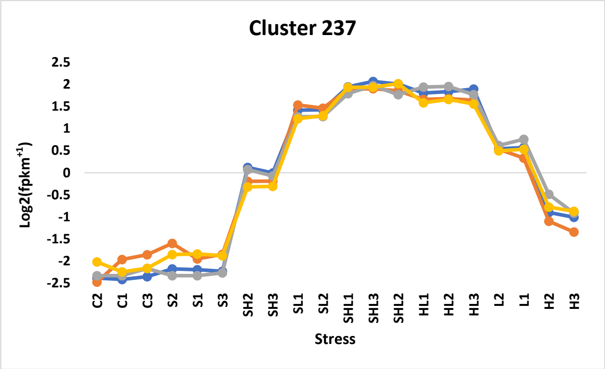
Cluster 237
Log2(fpkm+1)
Stress
C2 C1 C3 S2 S1 S3 SH2 SH3 SL1 SL2 SHL1 SHL3 SHL2 HL1 HL2 HL3 L2 L1 H2 H3

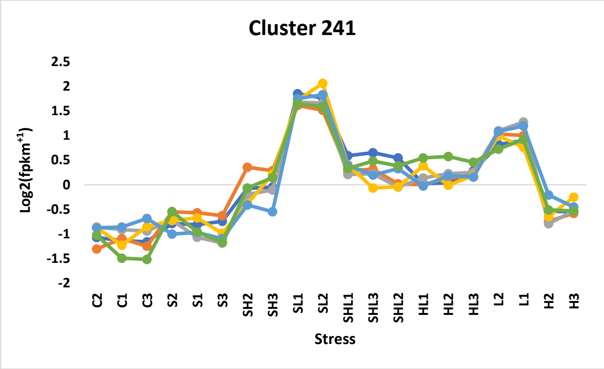
Cluster 241
Log2(fpkm+1)
Stress
C2 C1 C3 S2 S1 S3 SH2 SH3 SL1 SL2 SHL1 SHL3 SHL2 HL1 HL2 HL3 L2 L1 H2 H3

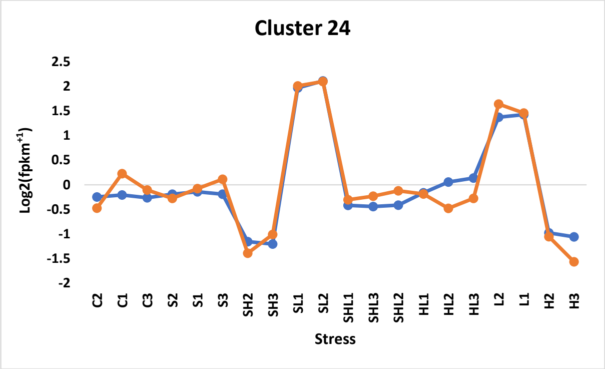
Cluster 24
Log2(fpkm+1)
Stress
C2 C1 C3 S2 S1 S3 SH2 SH3 SL1 SL2 SHL1 SHL3 SHL2 HL1 HL2 HL3 L2 L1 H2 H3

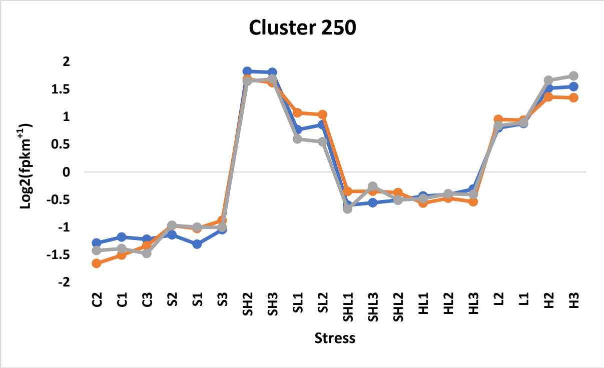
Cluster 250
Log2(fpkm+1)
Stress
C2 C1 C3 S2 S1 S3 SH2 SH3 SL1 SL2 SHL1 SHL3 SHL2 HL1 HL2 HL3 L2 L1 H2 H3

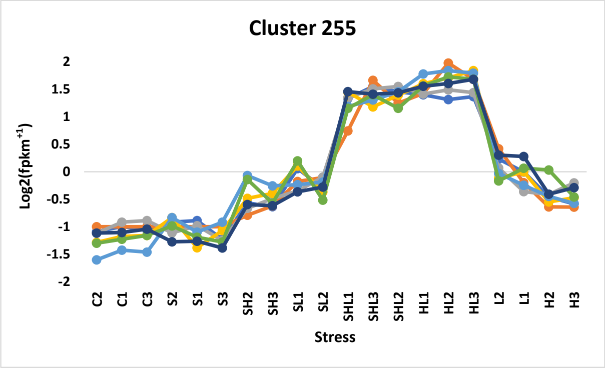
Cluster 255
Log2(fpkm+1)
Stress
C2 C1 C3 S2 S1 S3 SH2 SH3 SL1 SL2 SHL1 SHL3 SHL2 HL1 HL2 HL3 L2 L1 H2 H3

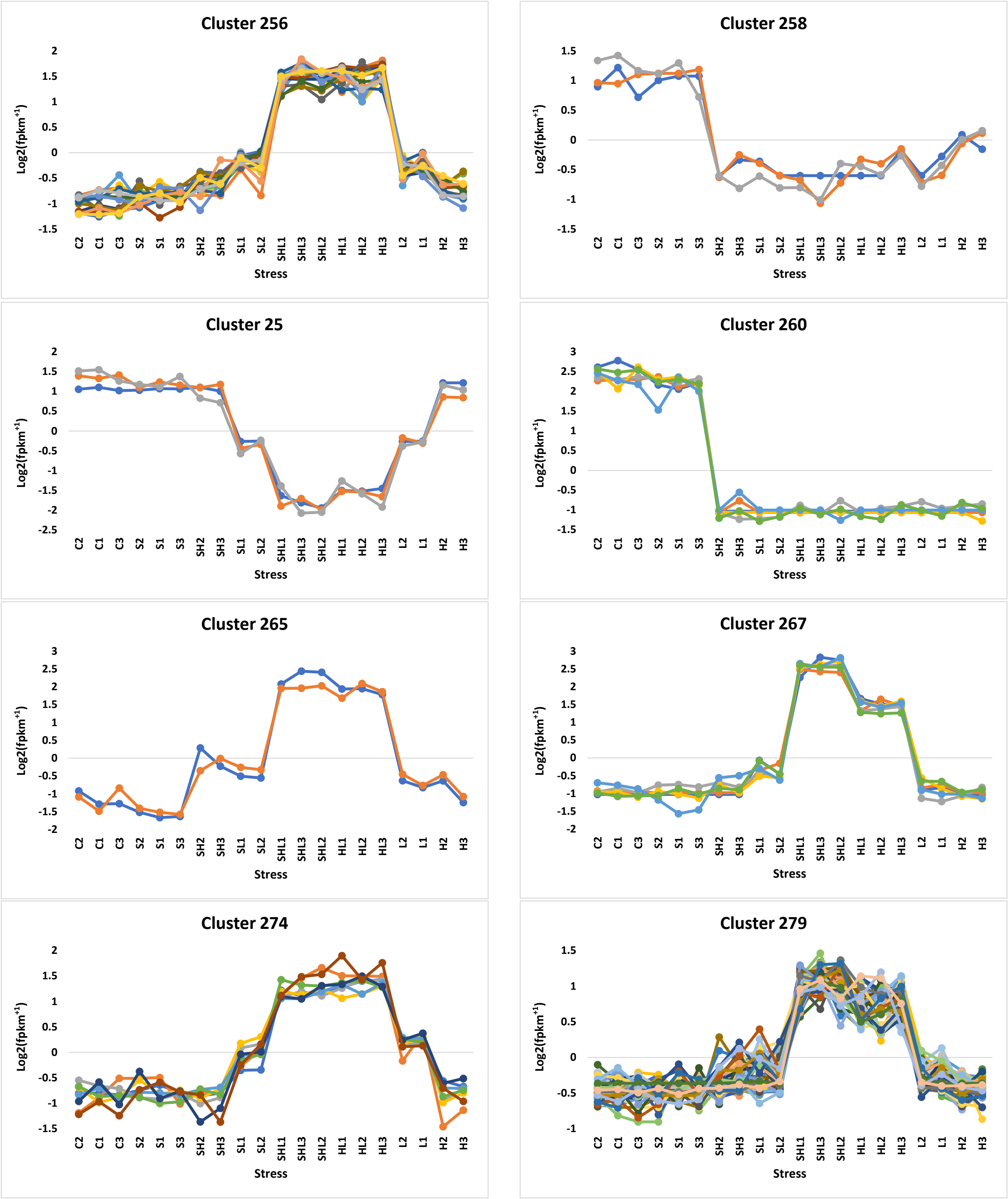

Figure S2. Continued

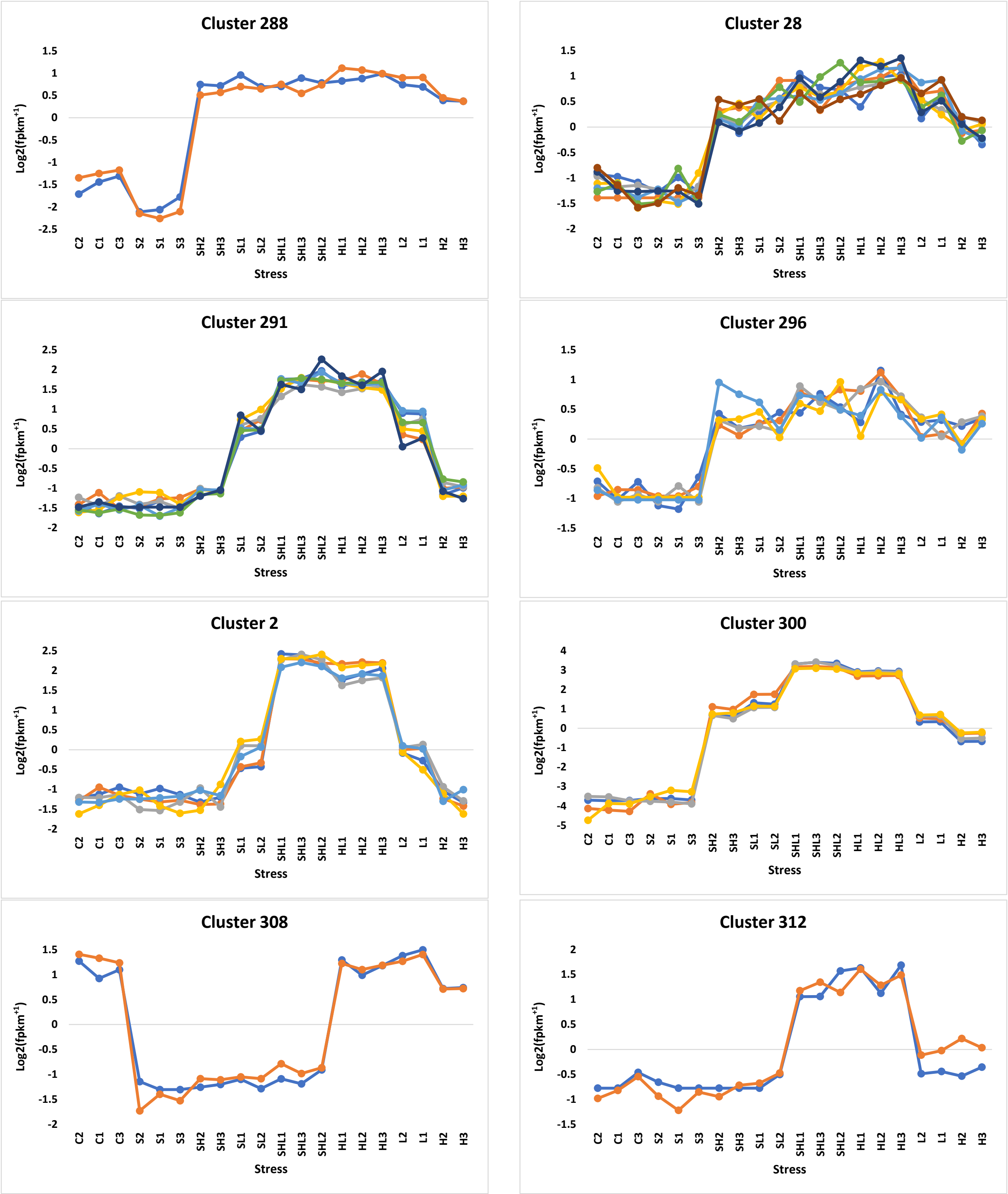

Figure S2. Continued

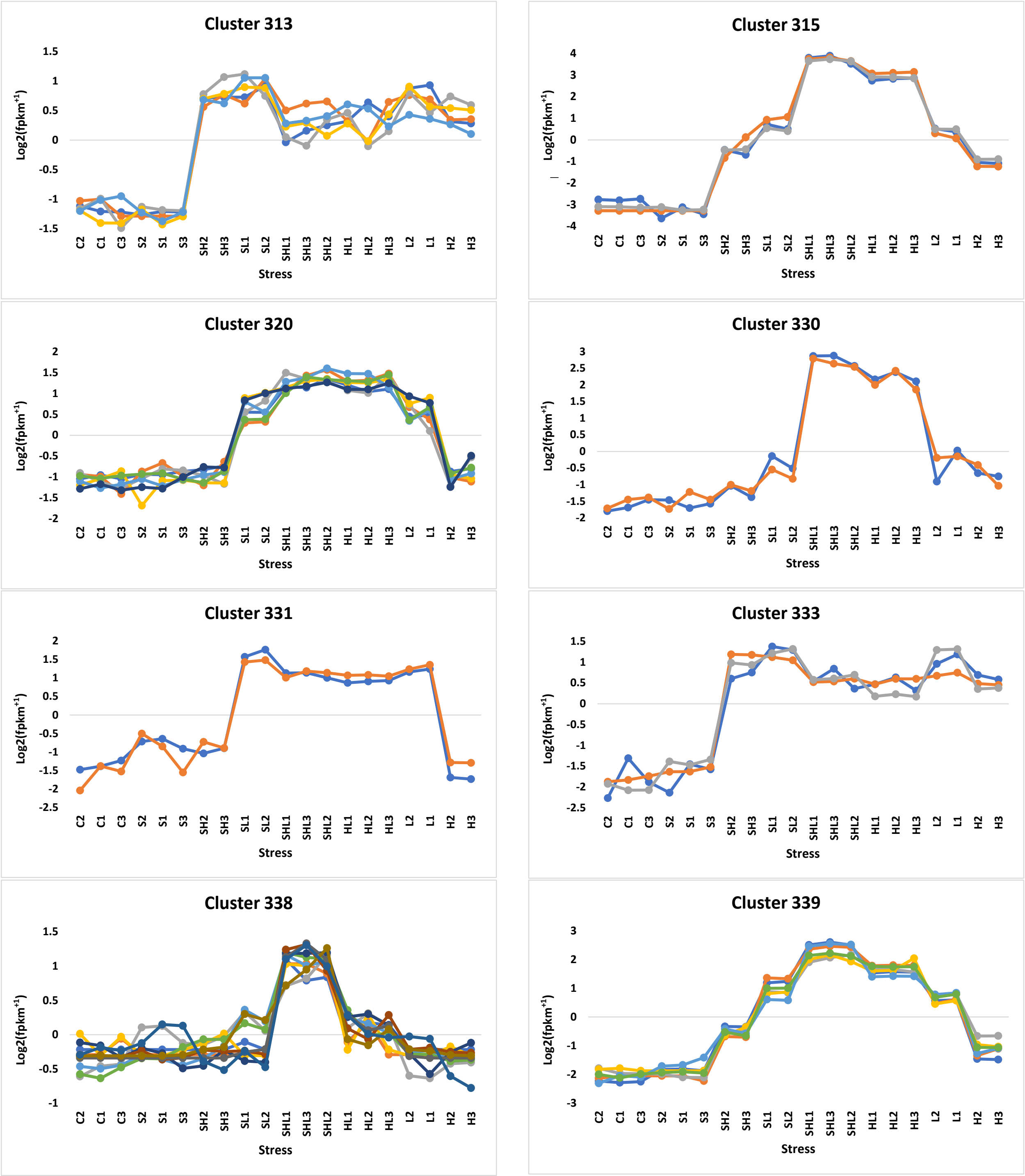

Figure S2. Continued

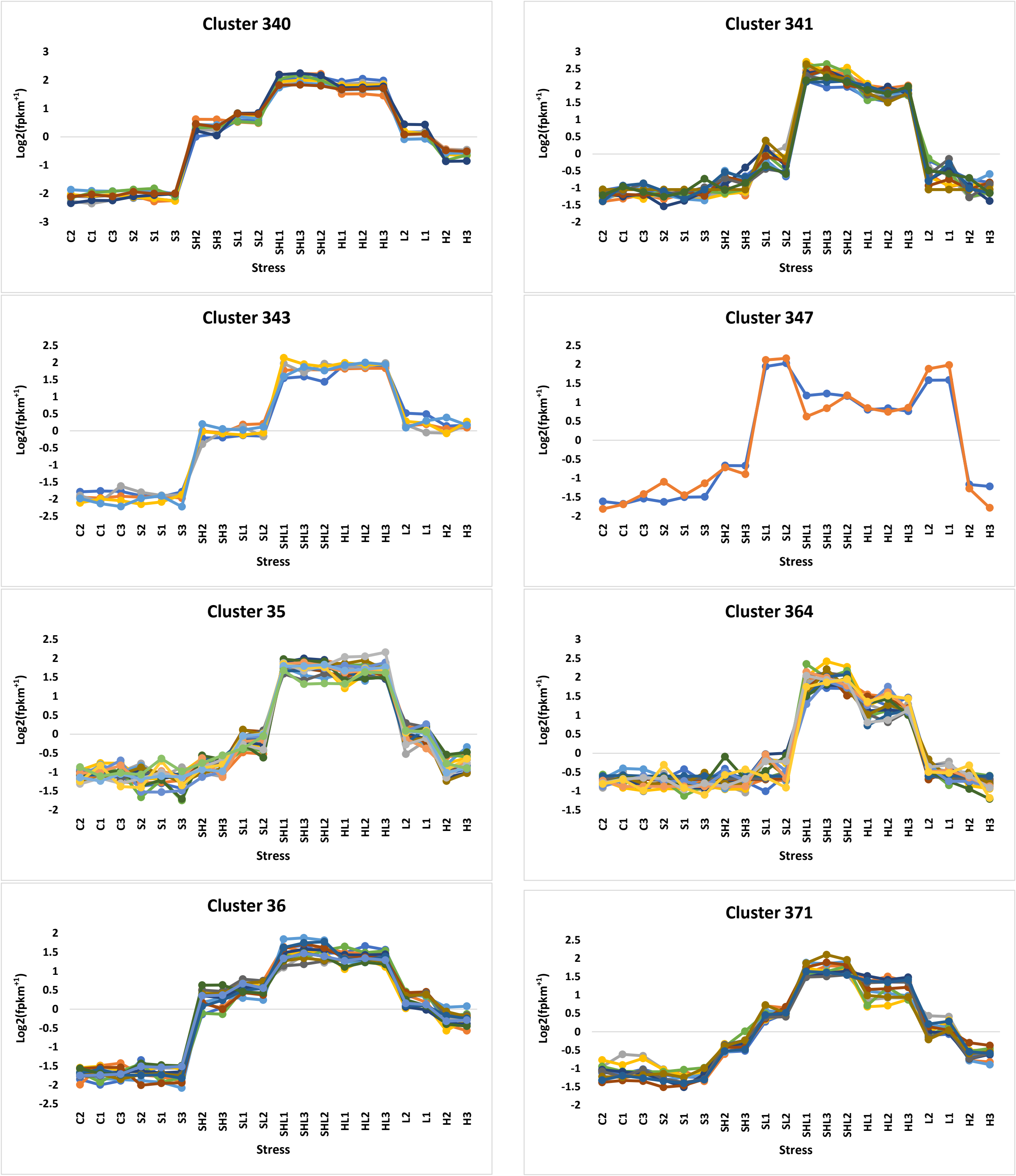

Figure S2. Continued

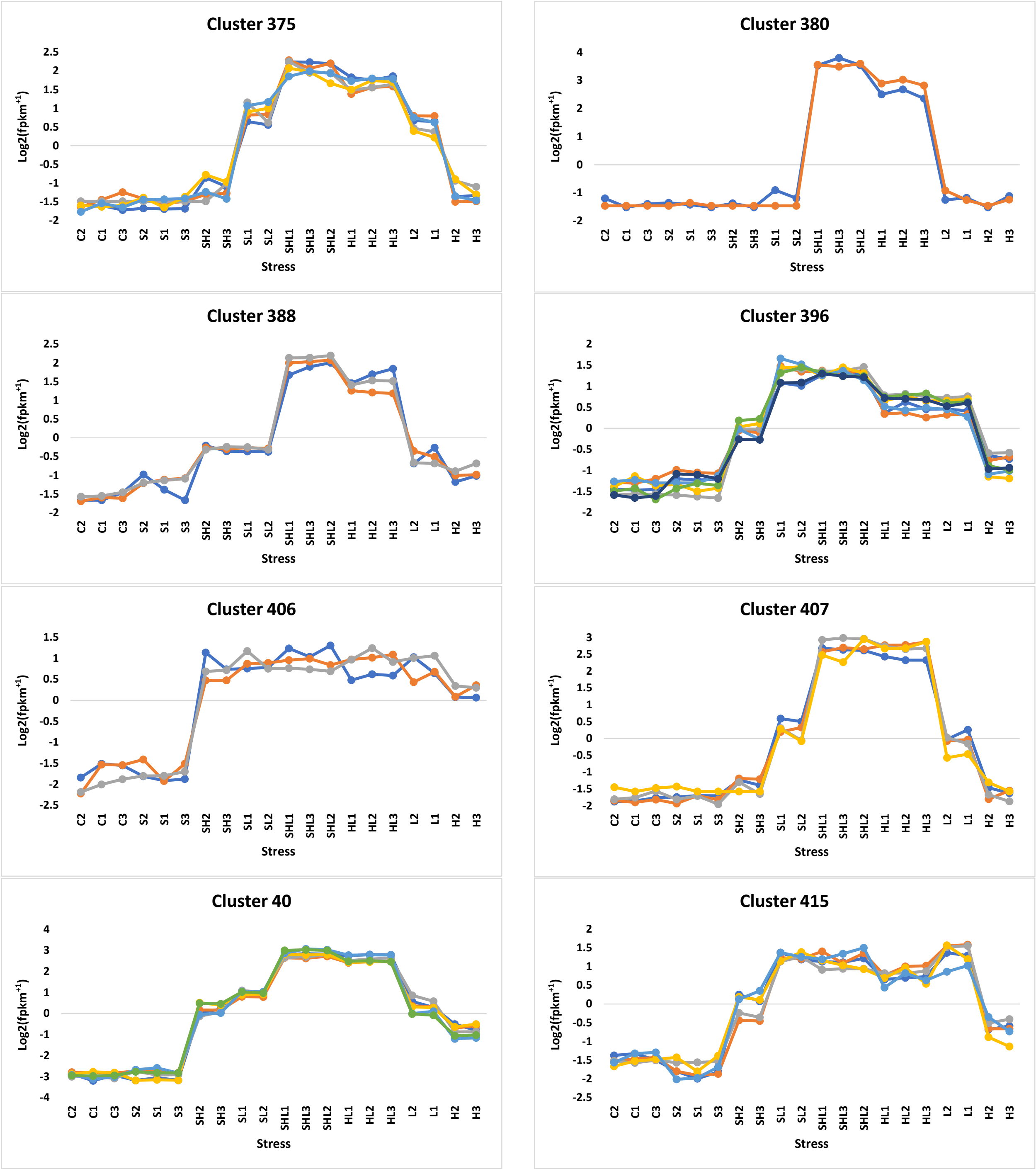

Figure S2. Continued

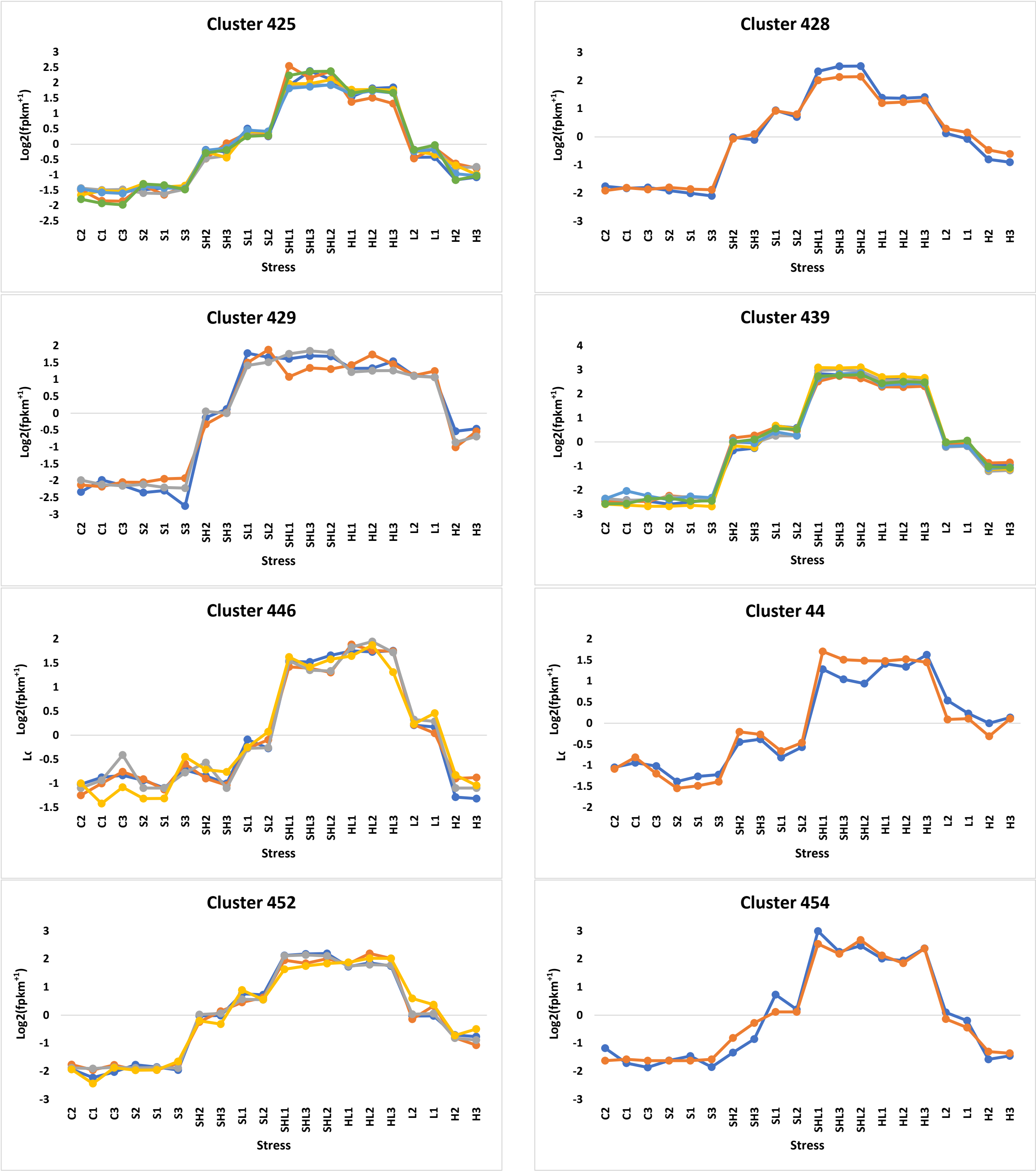

Figure S2. Continued

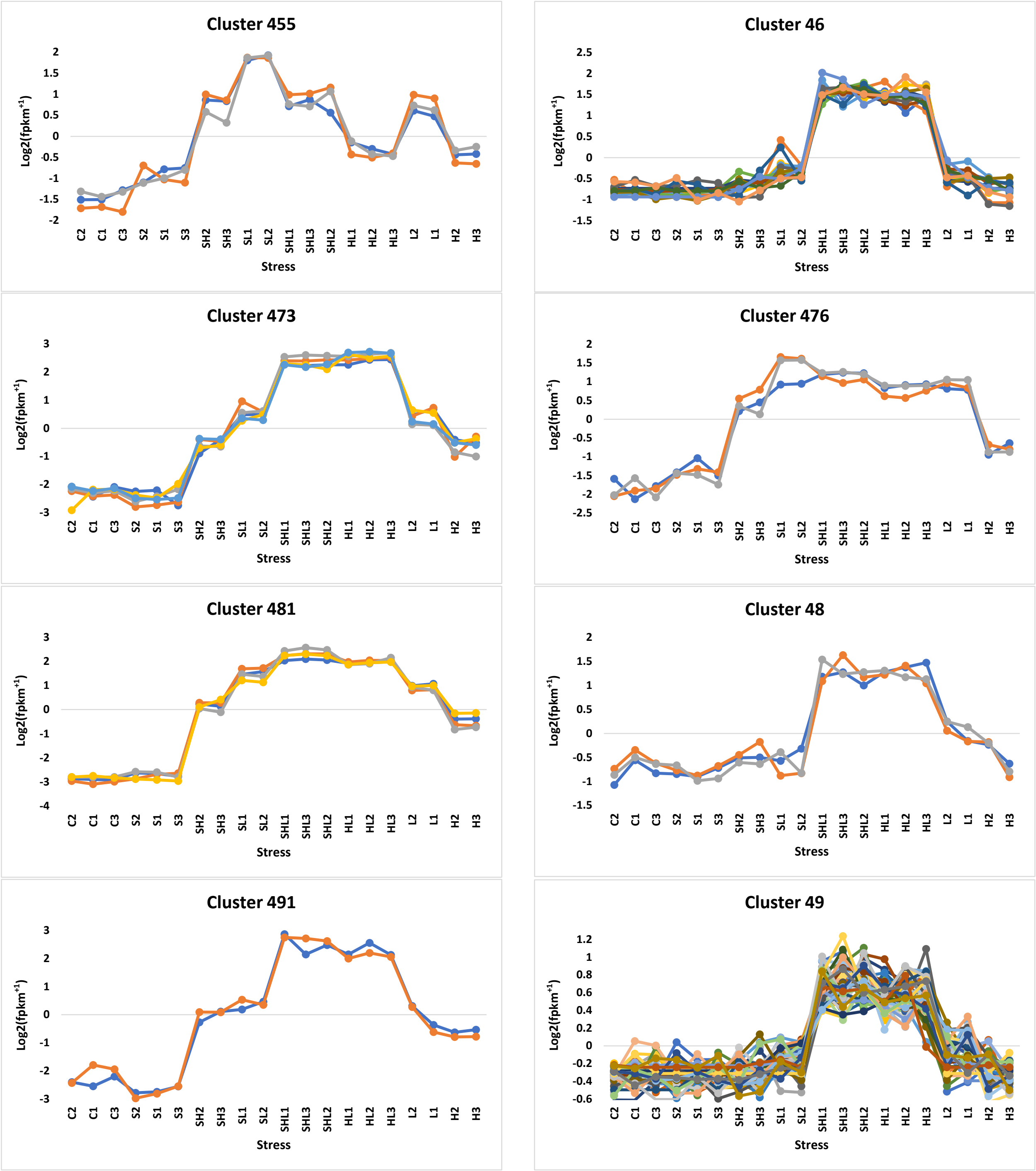

Figure S2. Continued

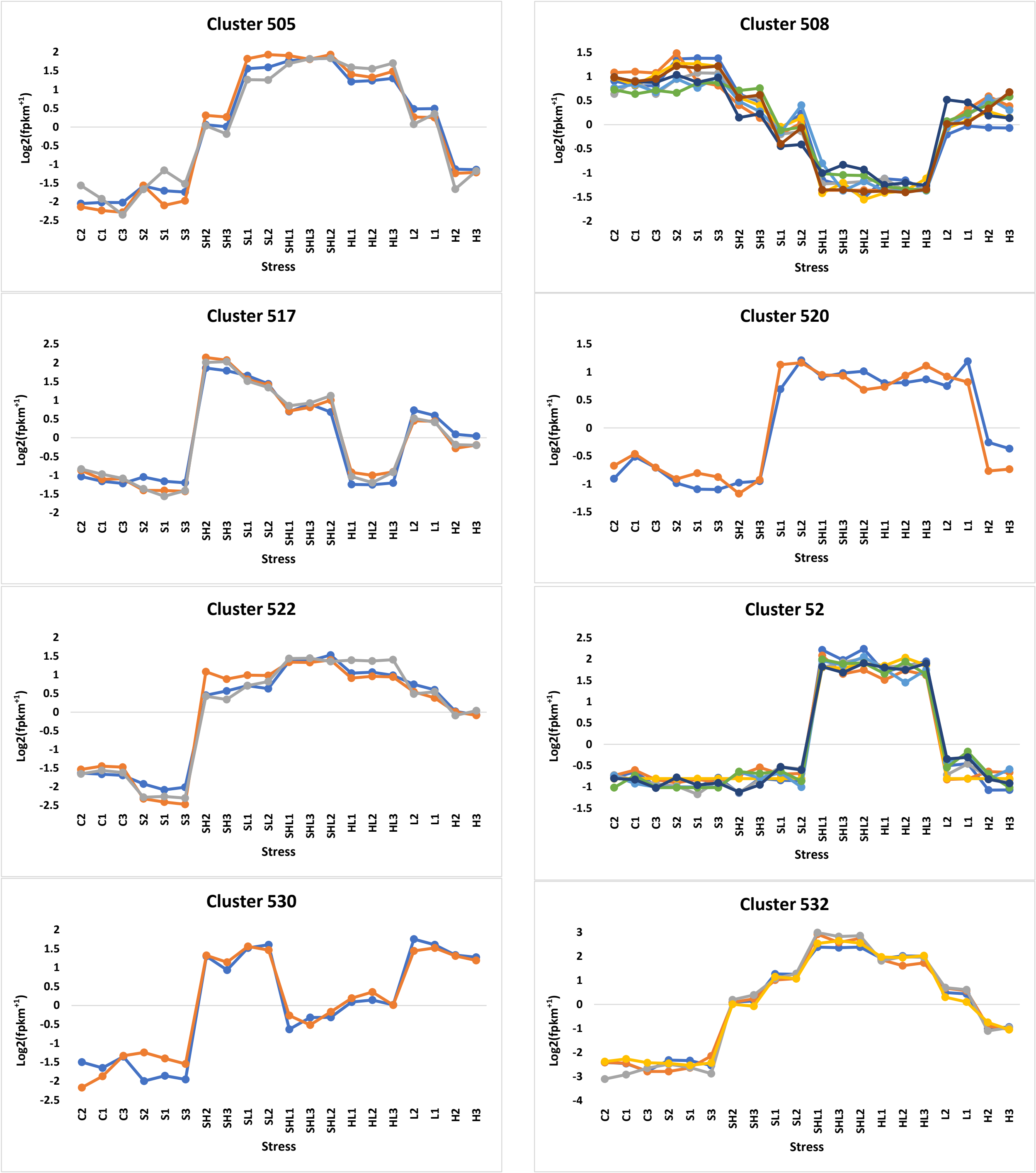

Figure S2. Continued

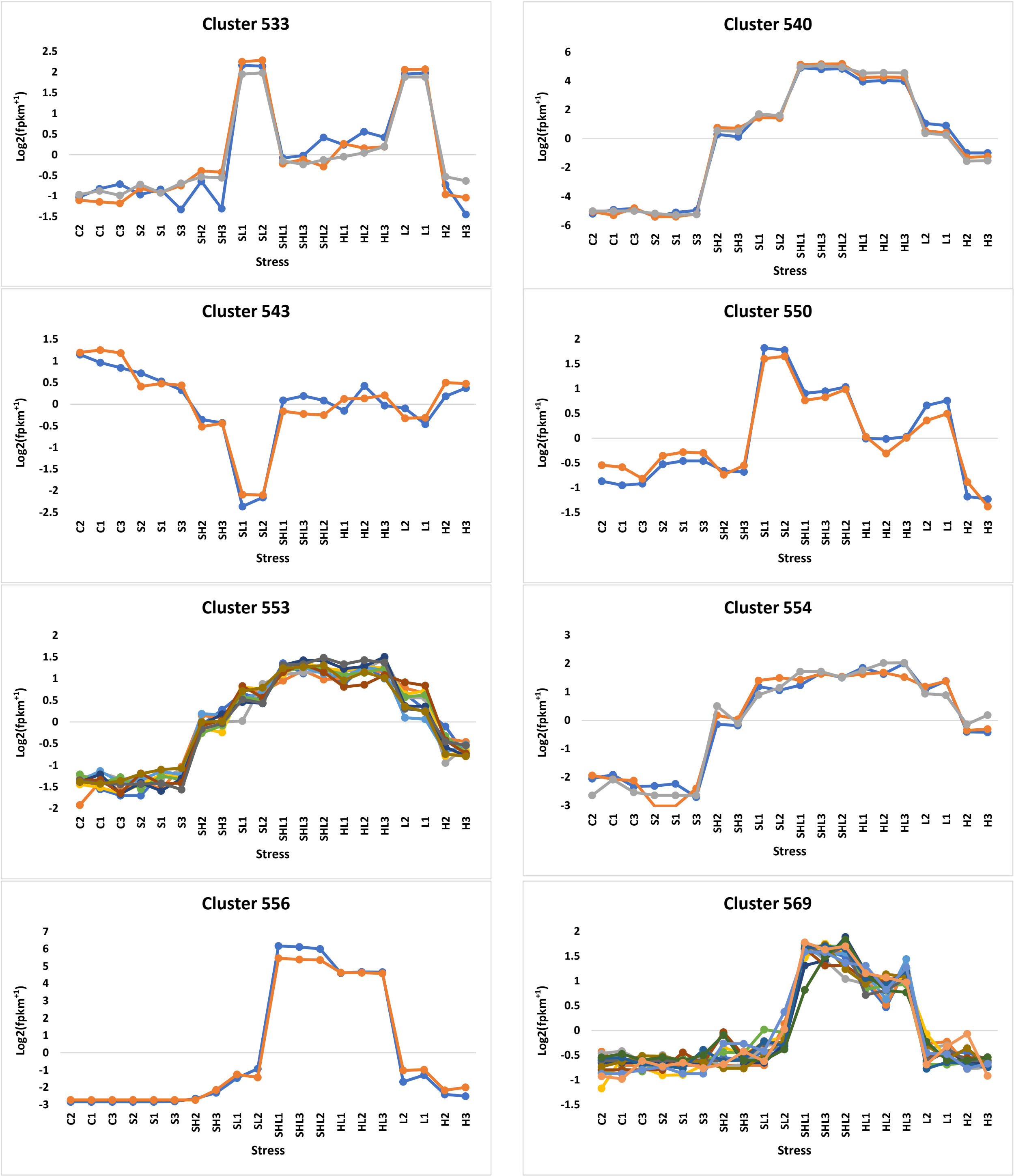

Figure S2. Continued

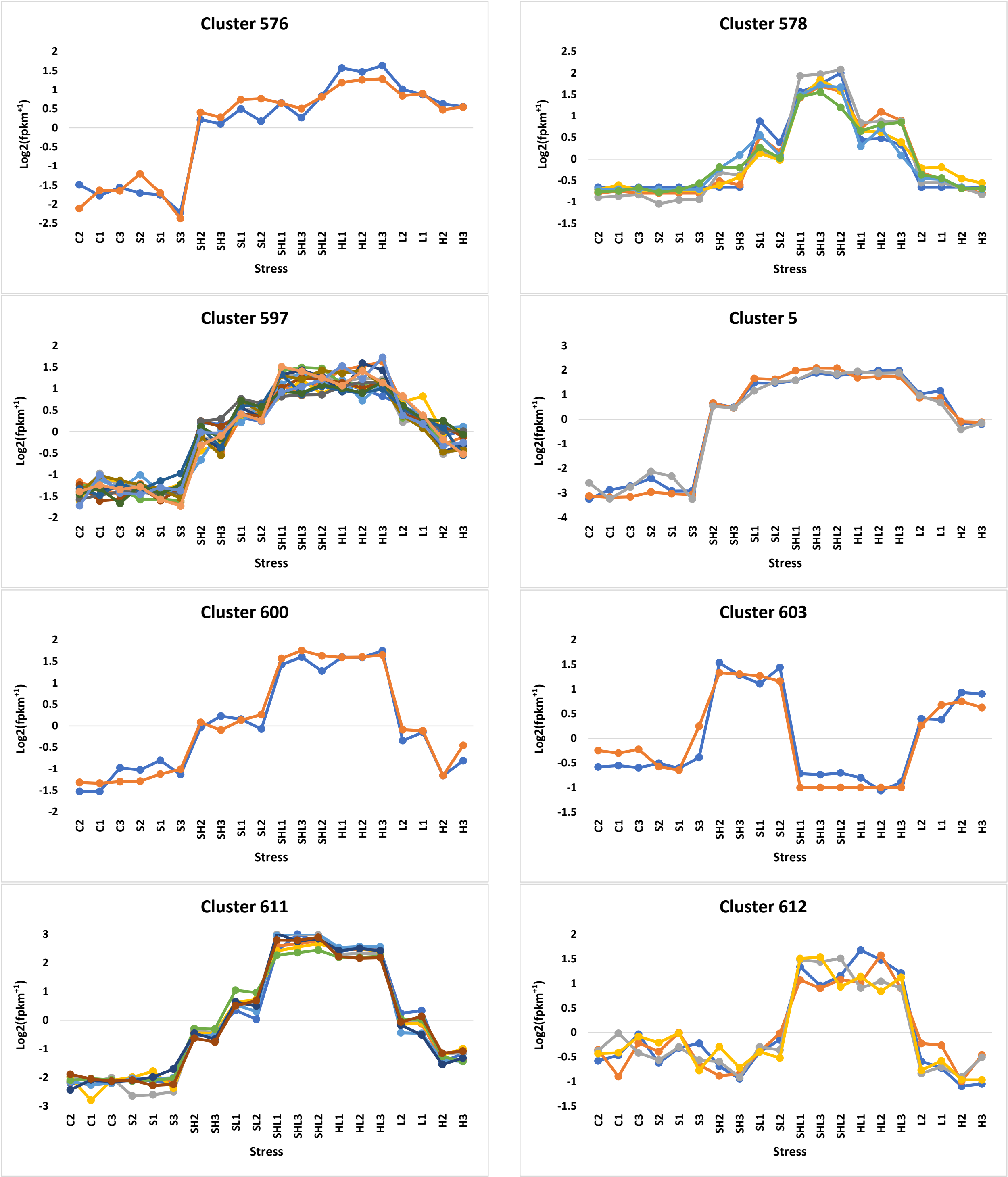

Figure S2. Continued

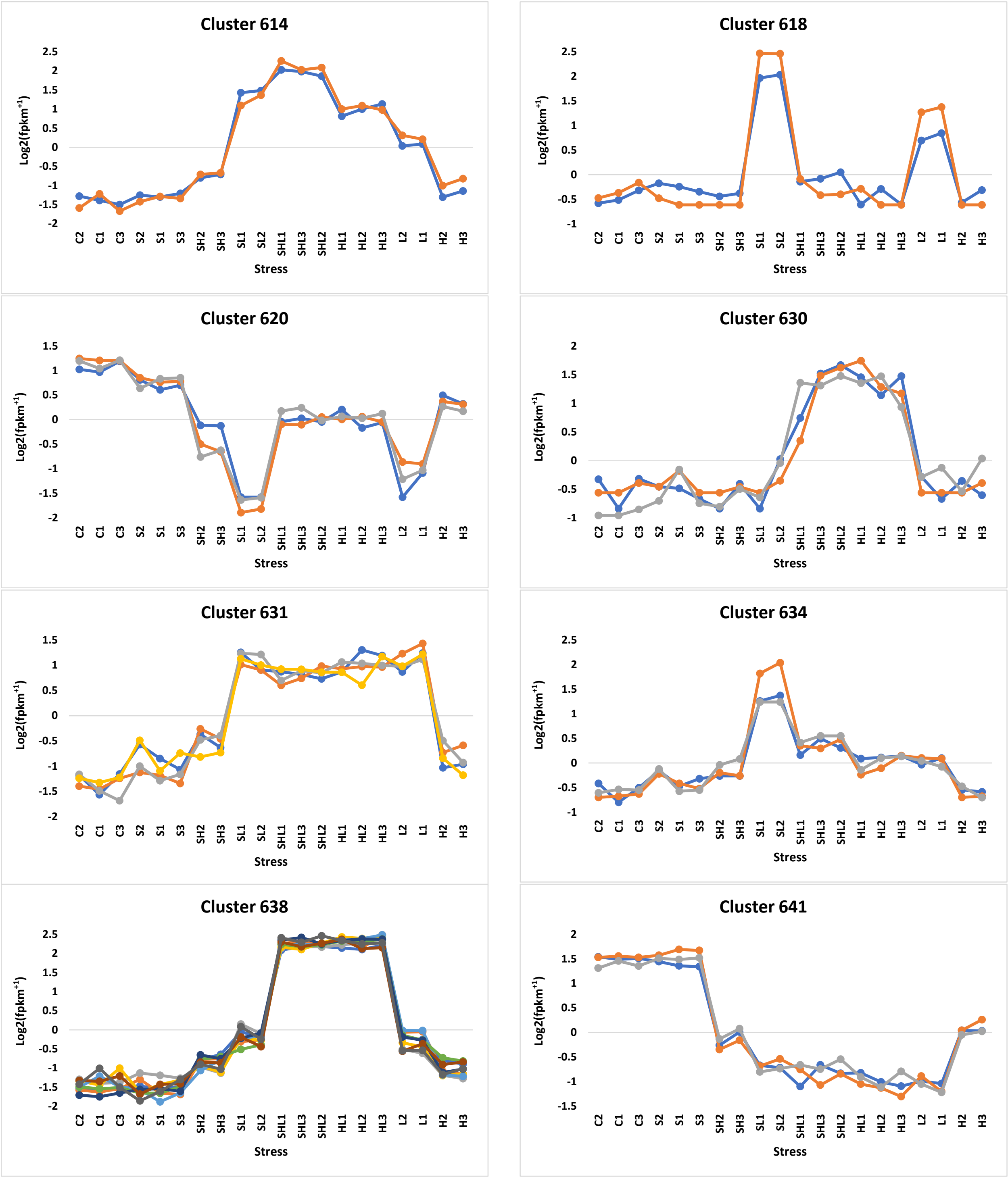

Figure S2. Continued

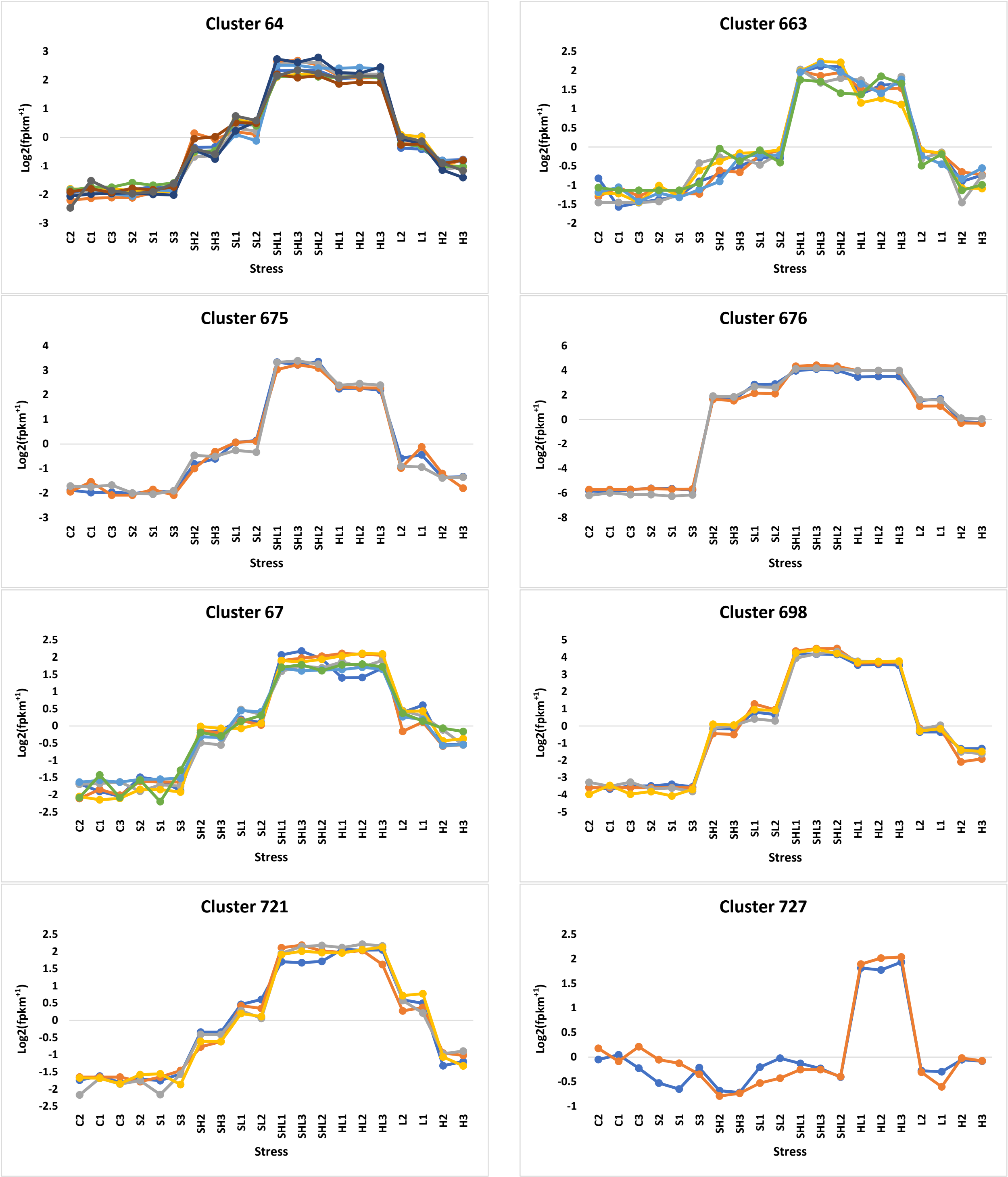

Figure S2. Continued

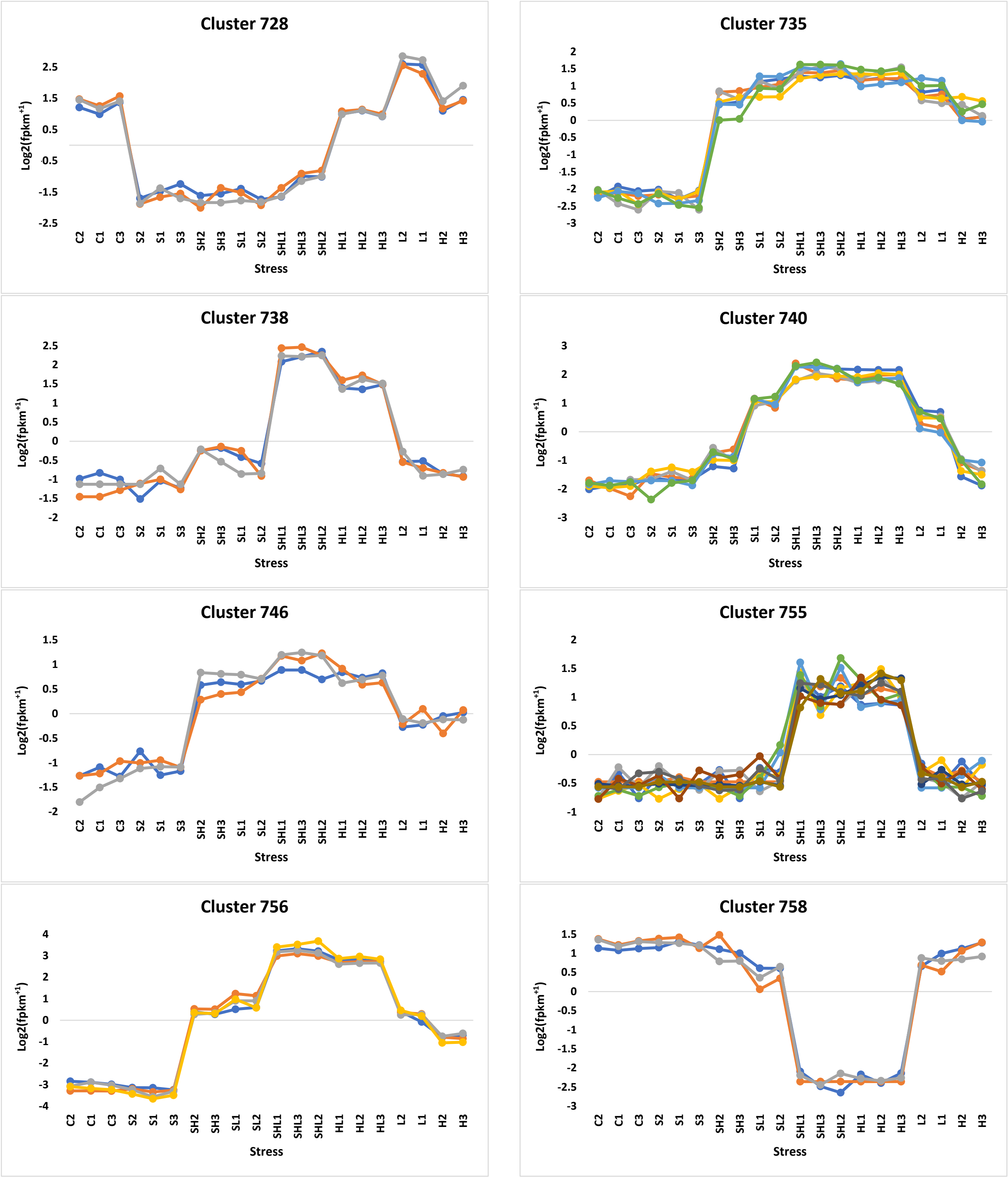

Figure S2. Continued

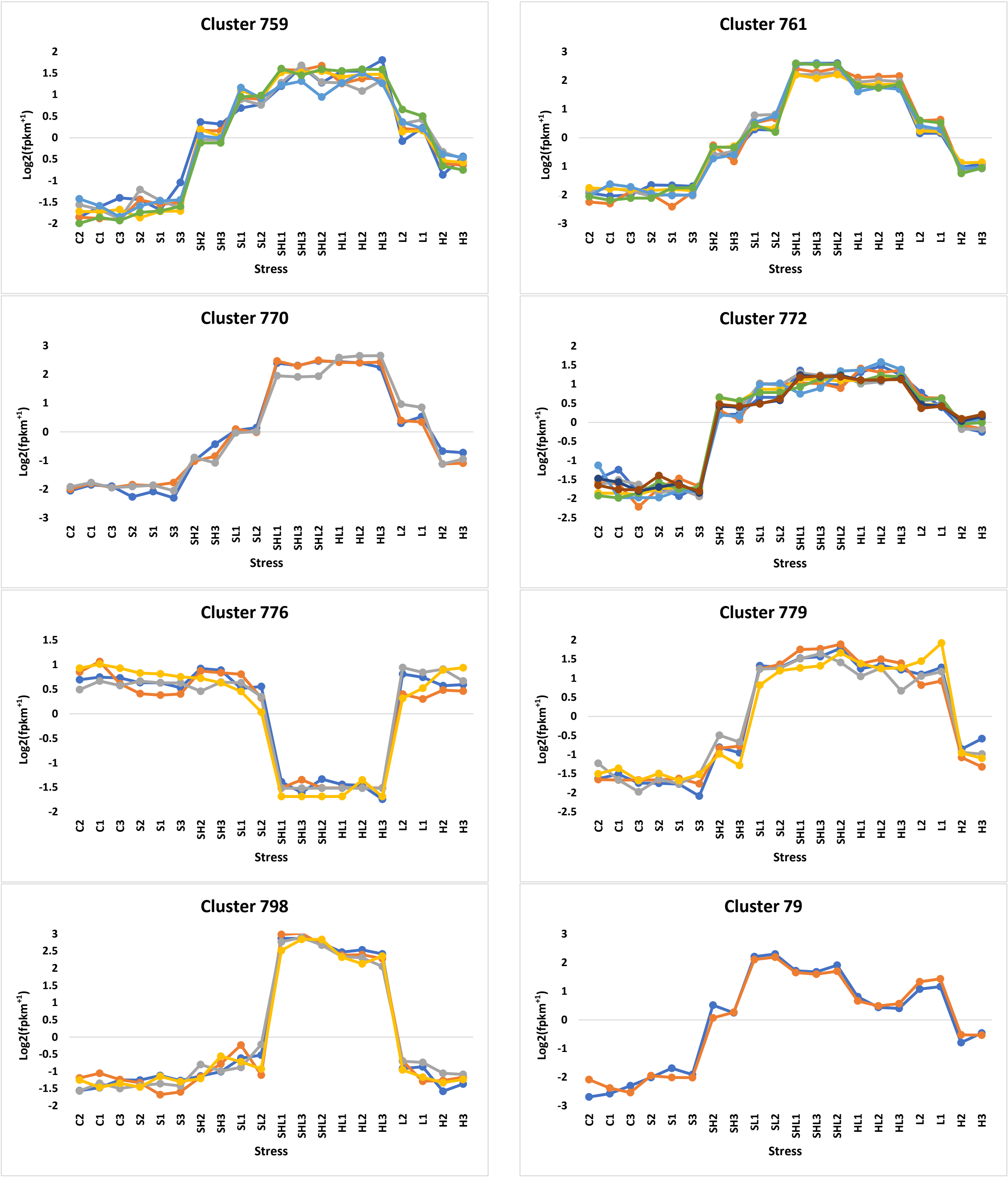

Figure S2. Continued

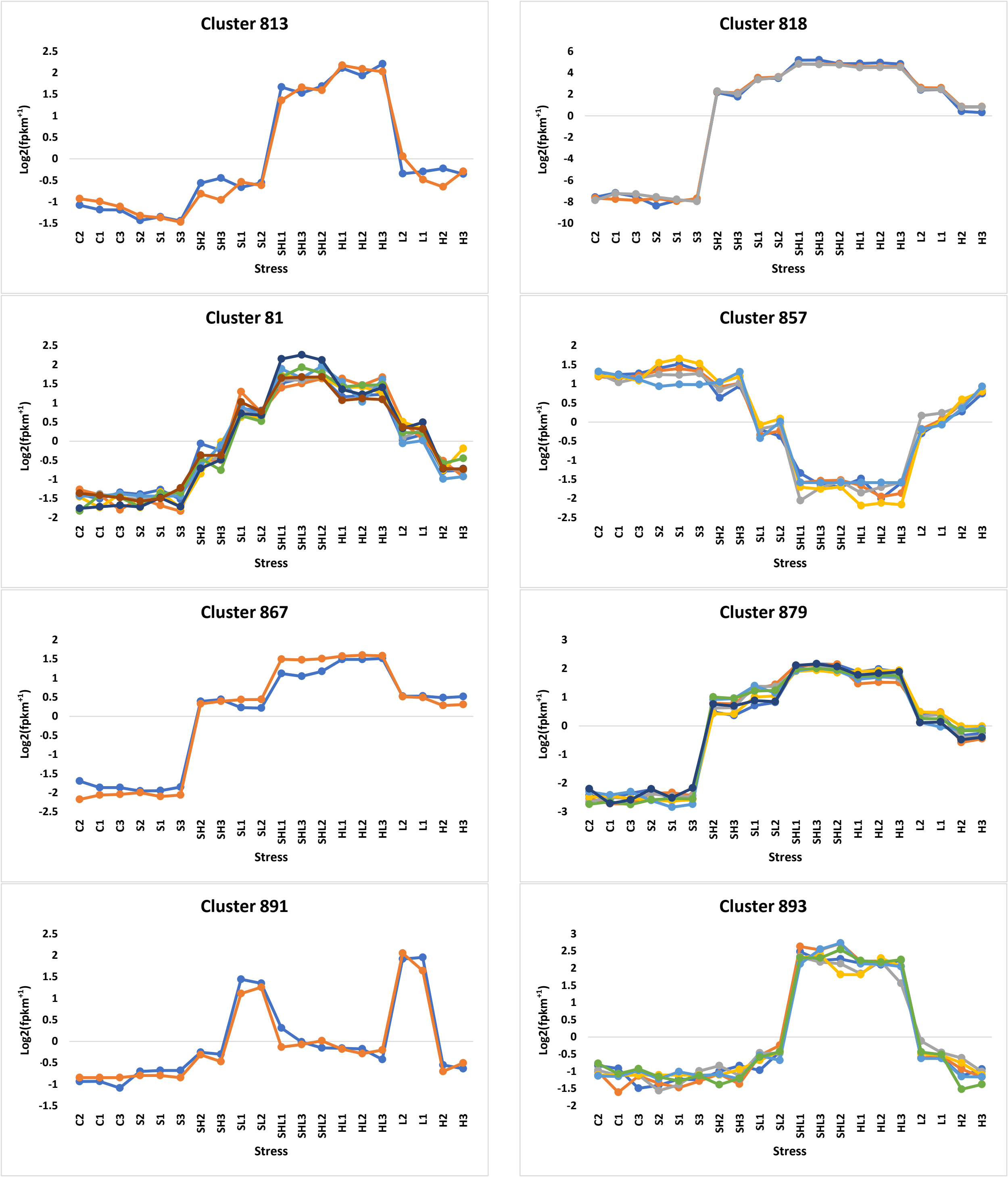

Figure S2. Continued

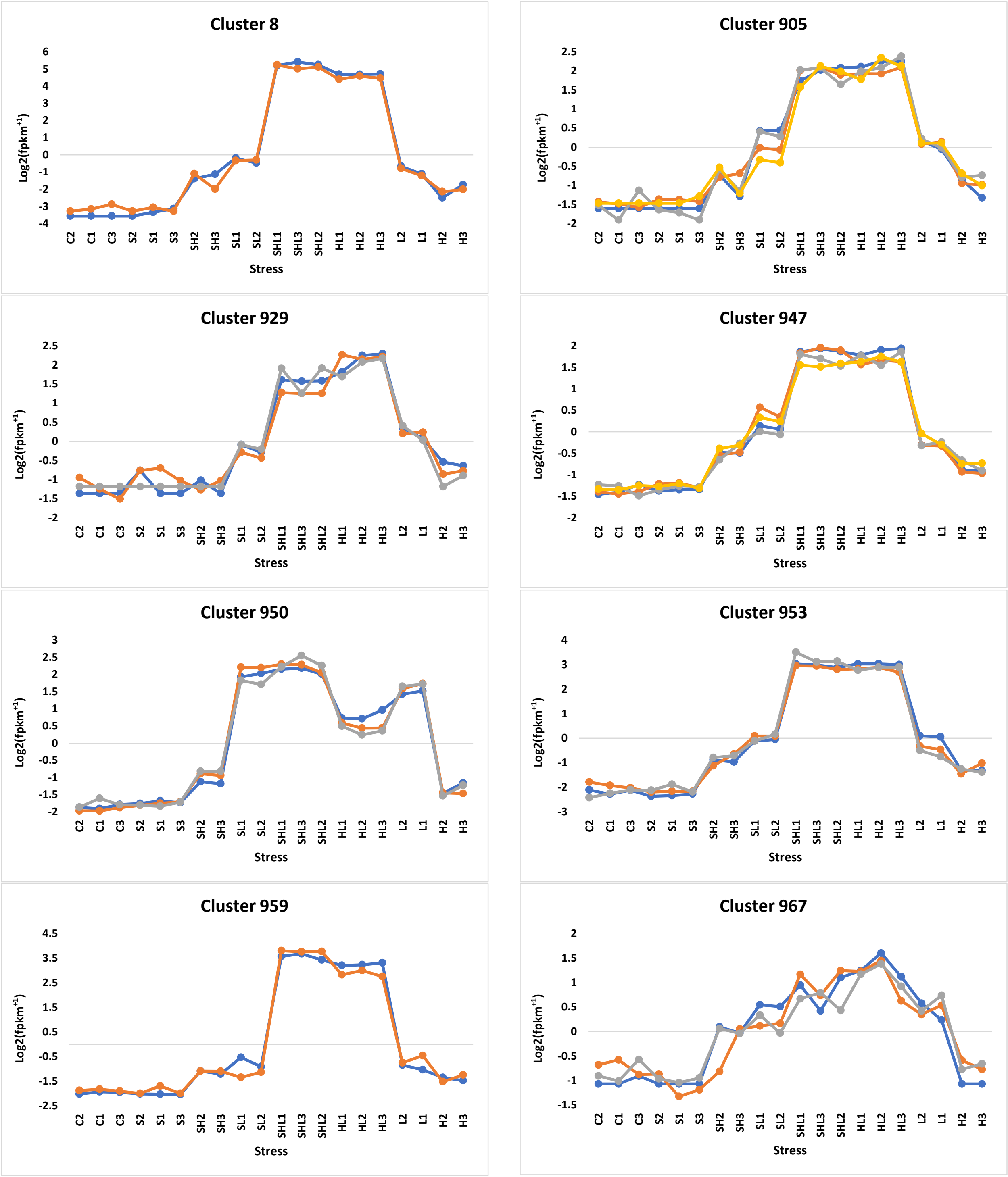

Figure S2. Continued

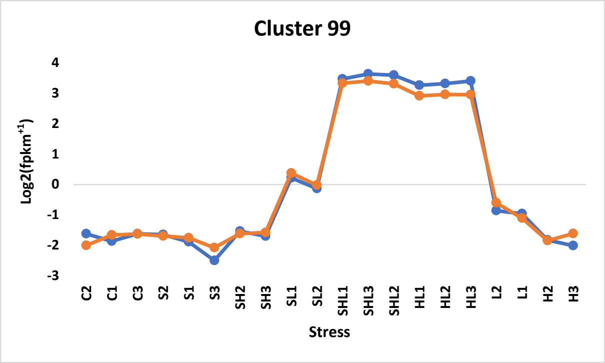

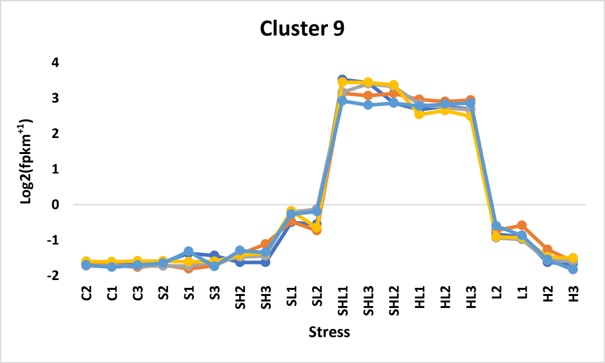

Figure S2. Continued

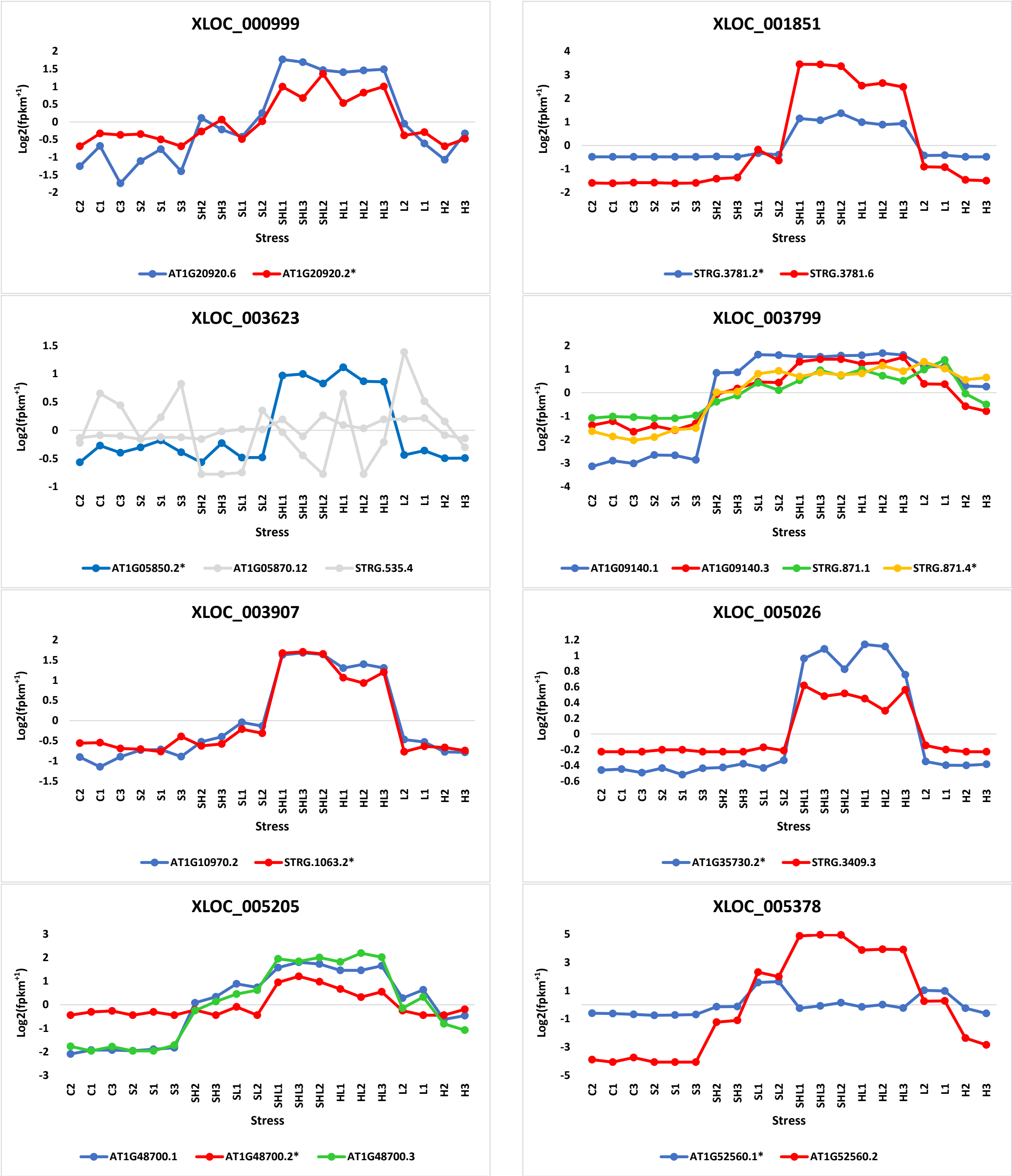

Figure S3. Expression profiling of loci involving DAS genes concordantly expressed with splicing factors (SFs) within the most consistent expression patterns generated from transcriptome datasets of 10-d-old *A. thaliana* (wild-type Col-0) seedlings exposed to different multifactorial stress combinations. C = control (0 mM NaCl, 21°C, 50 µmol m^{-2} s^{-1}), S = salt stress (50 mM NaCl, 21°C, 50 µmol m^{-2} s^{-1}), H = heat stress (0 mM NaCl, 33°C, 50 µmol m^{-2} s^{-1}), L = high light stress (0 mM NaCl, 21°C, 700 µmol m^{-2} s^{-1}). Selected genes should have one or more annotated or new stress-regulated isoforms existing on the same chromosome locus. Isoforms in light gray are not stress-regulated. Detailed information of gene-SF concordant expression is available in Table S3, while that of gene loci are shown in Table S6. Isoforms in light gray are not stress-regulated.

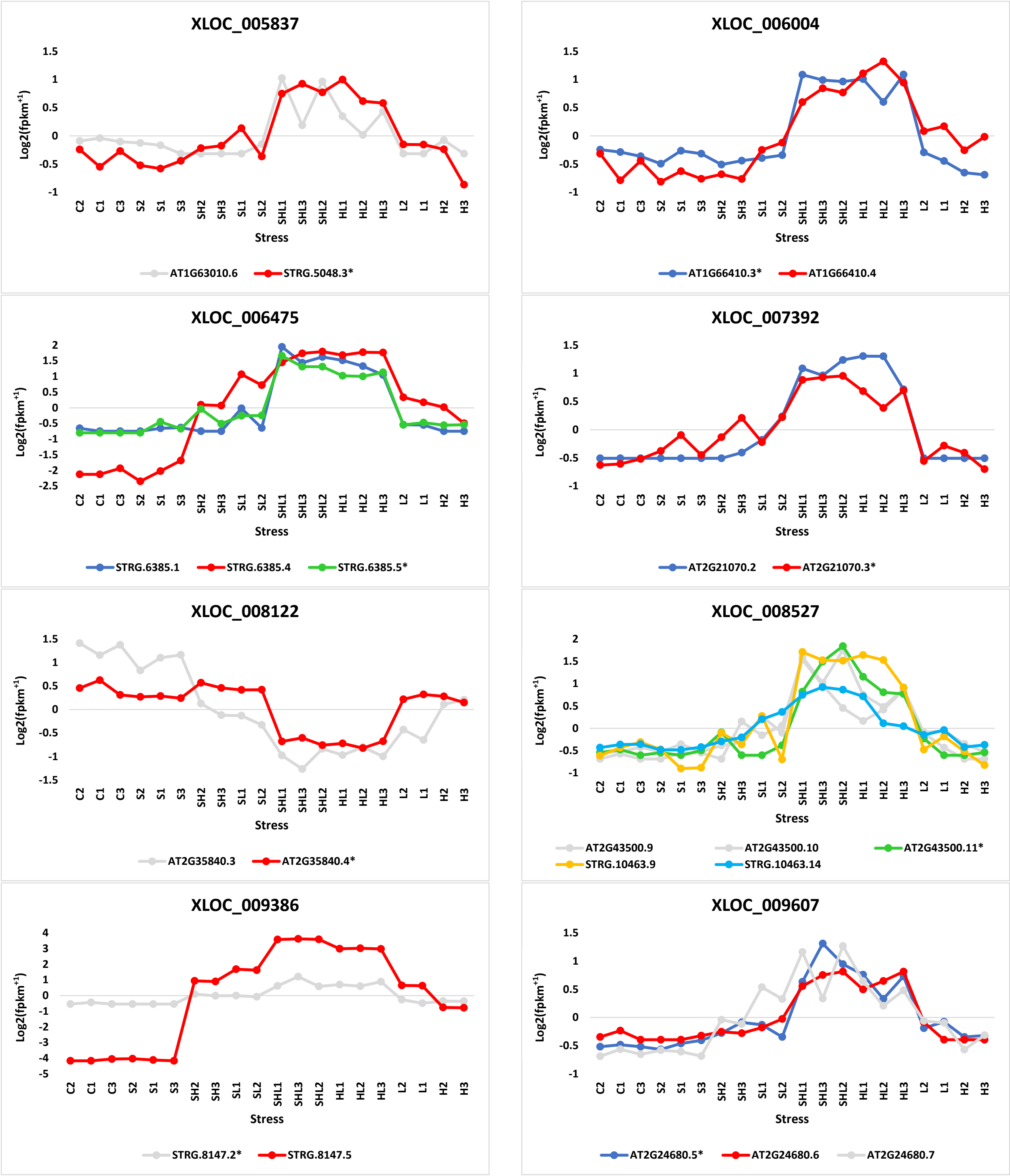

Figure S3. Continued

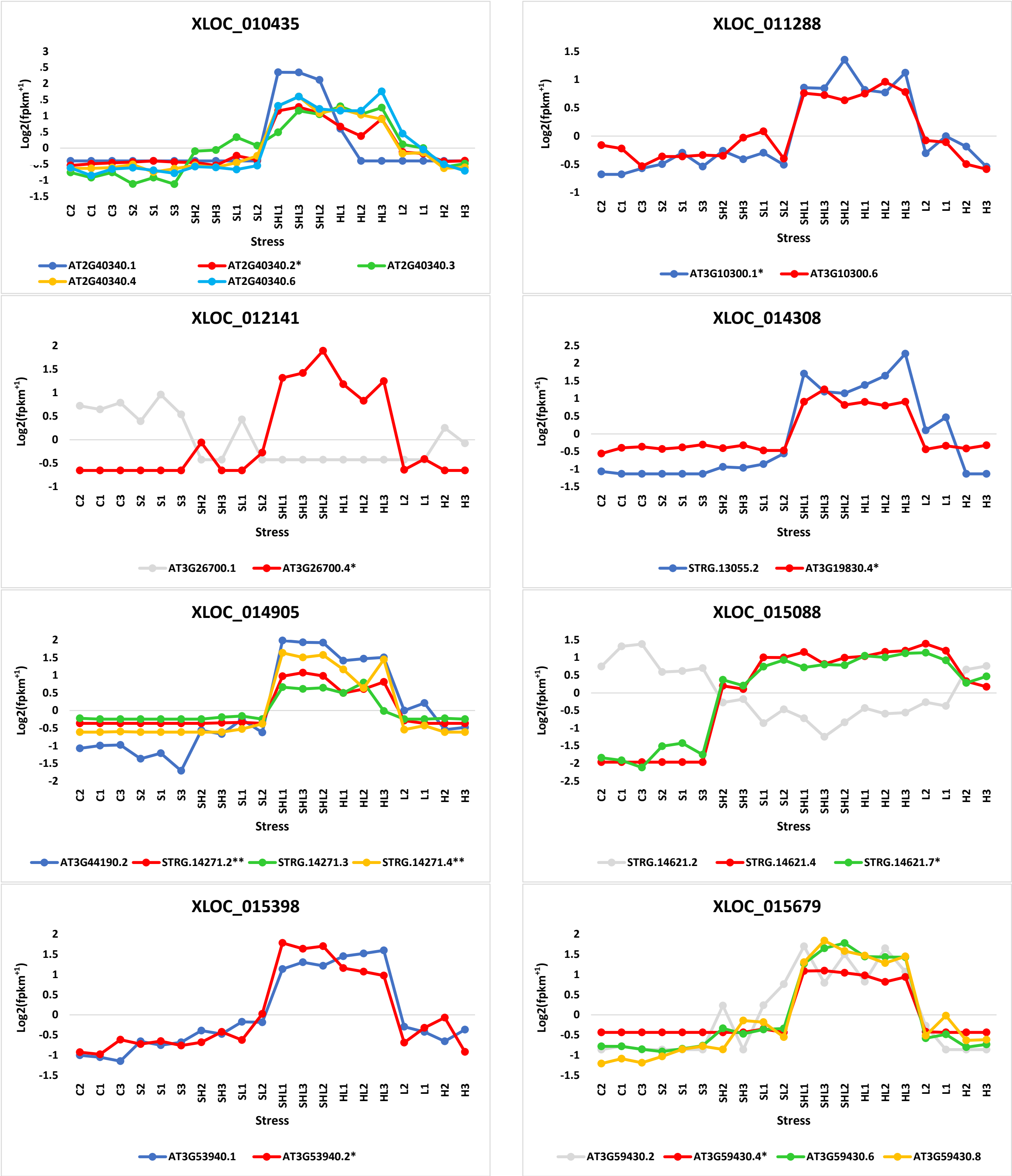

Figure S3. Continued

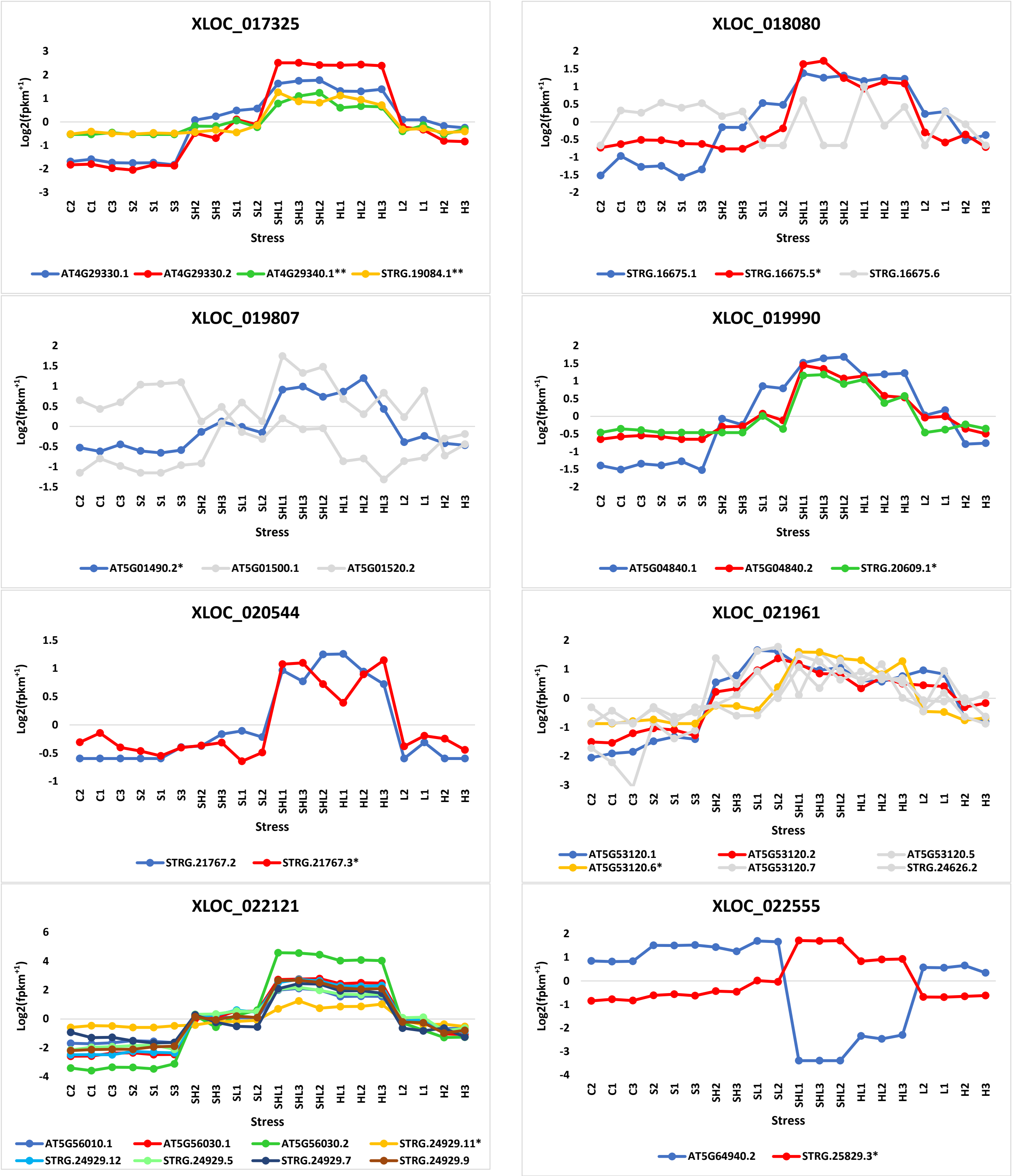

Figure S3. Continued

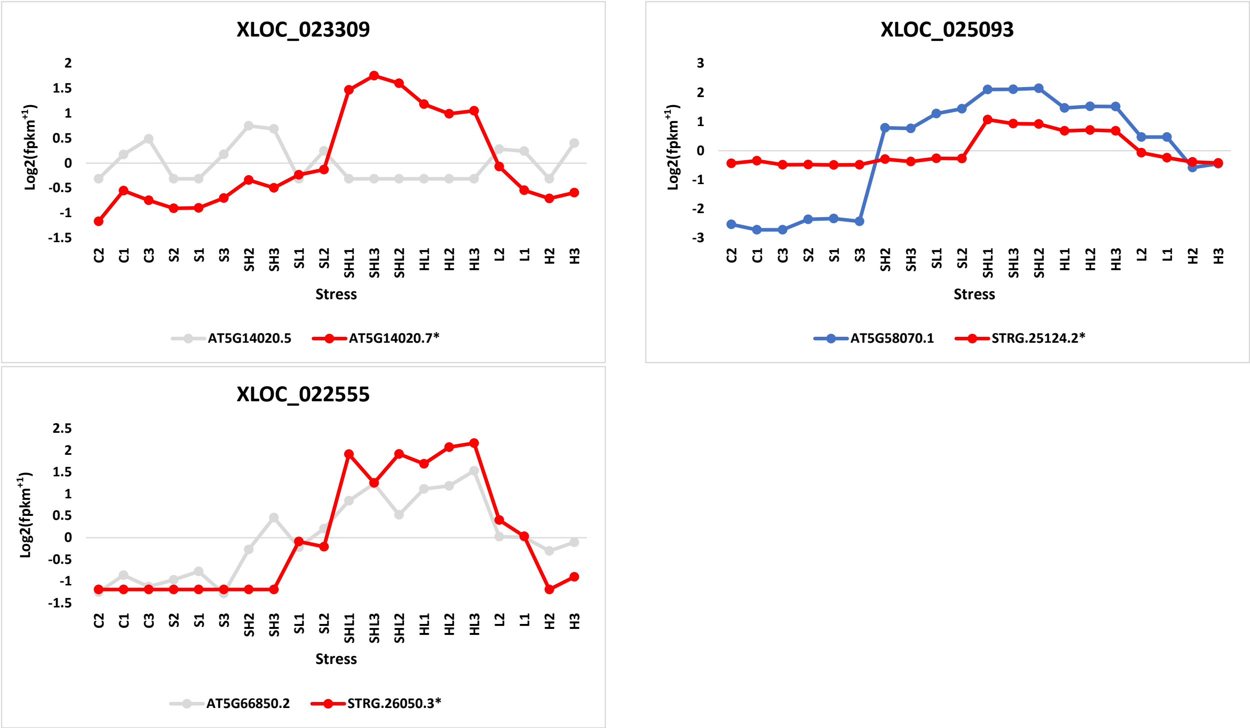

Figure S3. Continued

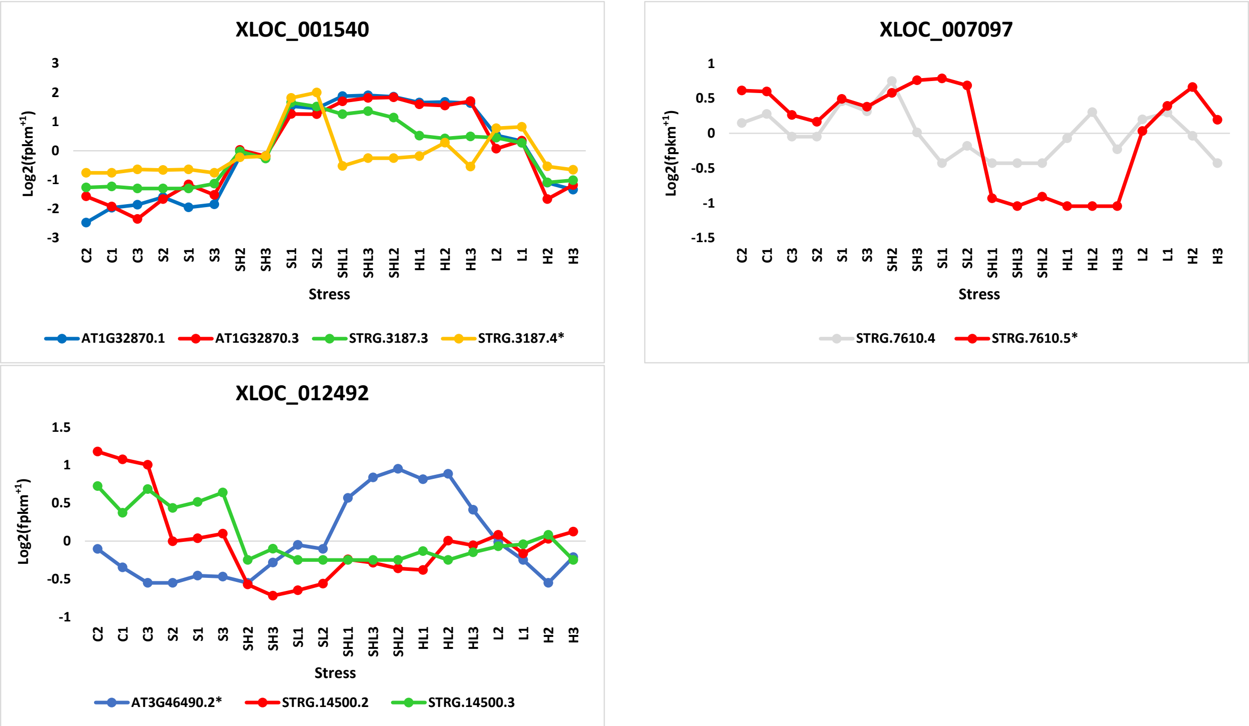

Figure S4. Expression profiling of loci with selected splicing factors that are concordantly expressed with DAS genes within the most consistent expression patterns generated from transcriptome datasets of 10-d-old *A. thaliana* (wild-type Col-0) seedlings exposed to different multifactorial stress combinations. C = control (0 mM NaCl, 21°C, 50 µmol m^{-2} s^{-1}), S = salt stress (50 mM NaCl, 21°C, 50 µmol m^{-2} s^{-1}), H = heat stress (0 mM NaCl, 33°C, 50 µmol m^{-2} s^{-1}), L = high light stress (0 mM NaCl, 21°C, 700 µmol m^{-2} s^{-1}). Selected splicing factors should have one or more Annotated or new stress-regulated isoforms existing on the same chromosome locus. Concordantly expressed splicing factors and their isoforms are shown in Table S4, while that of gene loci are shown in Table S6. Isoforms in light gray are not stress-regulated.

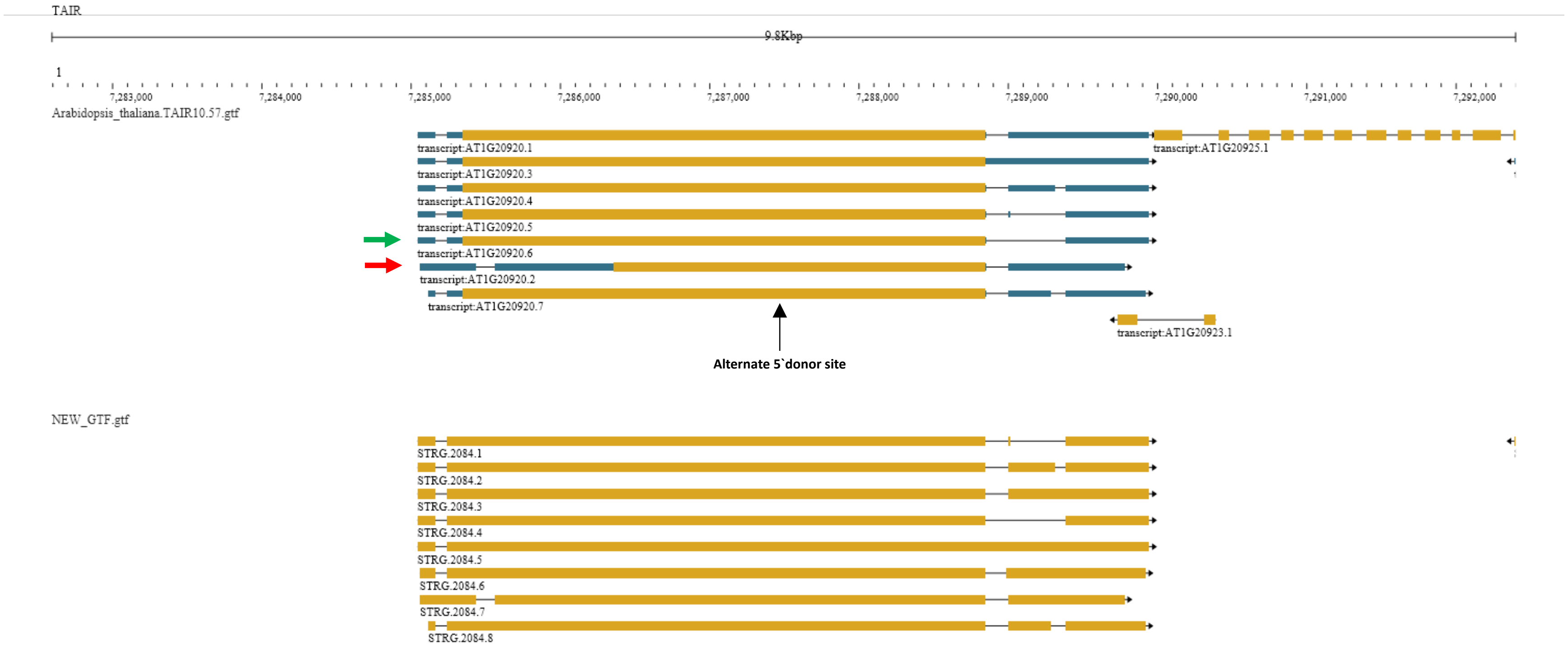

Figure S5. Structure of previously annotated and new DAS isoforms on XLOC_000999 (Gene 1, AT1G20920) generated due to different multifactorial stress combinations in the transcriptome datasets of 10-d-old *A. thaliana* (wild-type Col-0) seedlings. Red arrow refers to the gene isoform concordantly expressed with a given splicing factor, while green arrow(s) refer to other regulated isoforms of this gene. Other isoforms are not consistently regulated under the stress. Further information is available in Tables S1, S3 and S6.

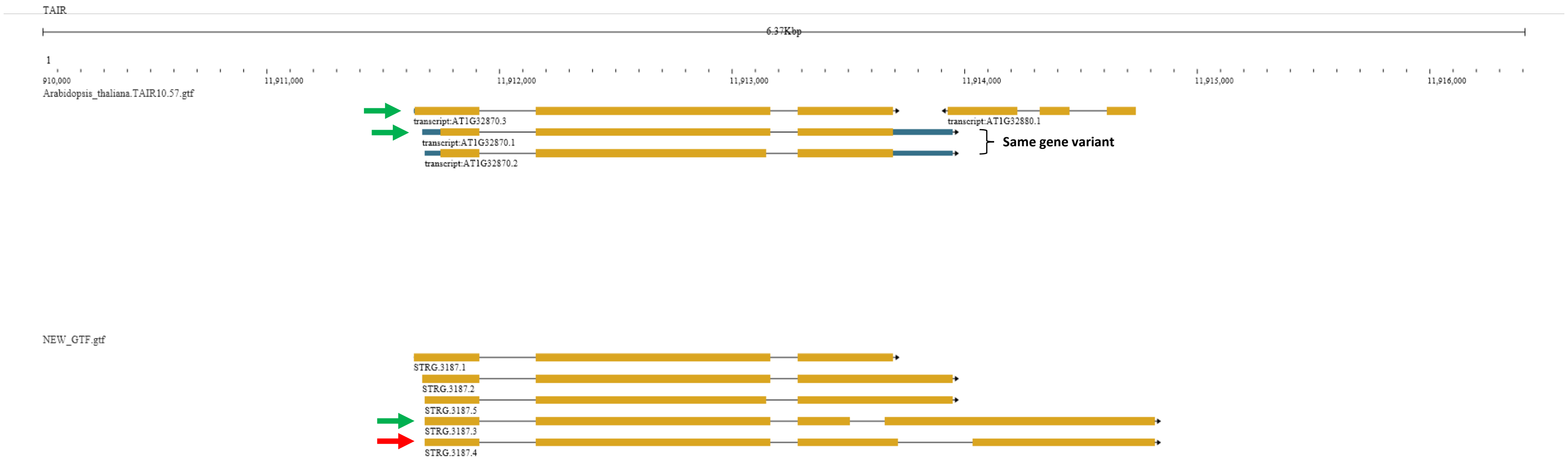

Figure S6. Structure of previously annotated and new SF isoforms on XLOC_001540 (SF1, AT1G32870) generated due to different multifactorial stress combinations in the transcriptome datasets of 10-d-old *A. thaliana* (wild-type Col-0) seedlings. Red arrow refers to the SF isoform concordantly expressed with a given gene, while green arrow(s) refer to other regulated isoforms of this gene. Other isoforms are not consistently regulated under the stress. Further information is available in Tables S1, S4 and S6.

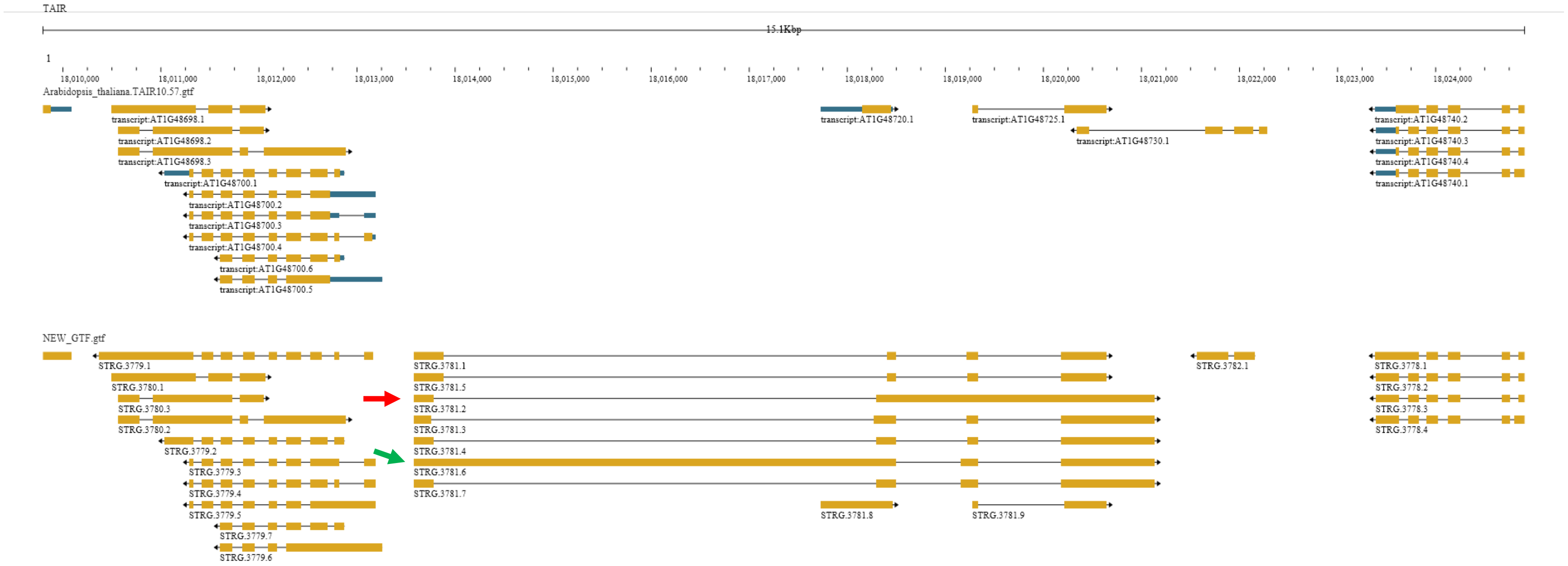

Figure S7. Structure of previously annotated and new DAS isoforms on XLOC_001851 (Gene 2, STRG.3781.2) generated due to different multifactorial stress combinations in the transcriptome datasets of 10-d-old *A. thaliana* (wild-type Col-0) seedlings. Red arrow refers to the gene isoform concordantly expressed with a given splicing factor, while green arrow(s) refer to other regulated isoforms of this gene. Other isoforms are not consistently regulated under the stress. Further information is available in Tables S1, S3 and S6. Regulated new isoforms of this DAS gene have no annotated gene to compare with, thus, was not analyzed further.

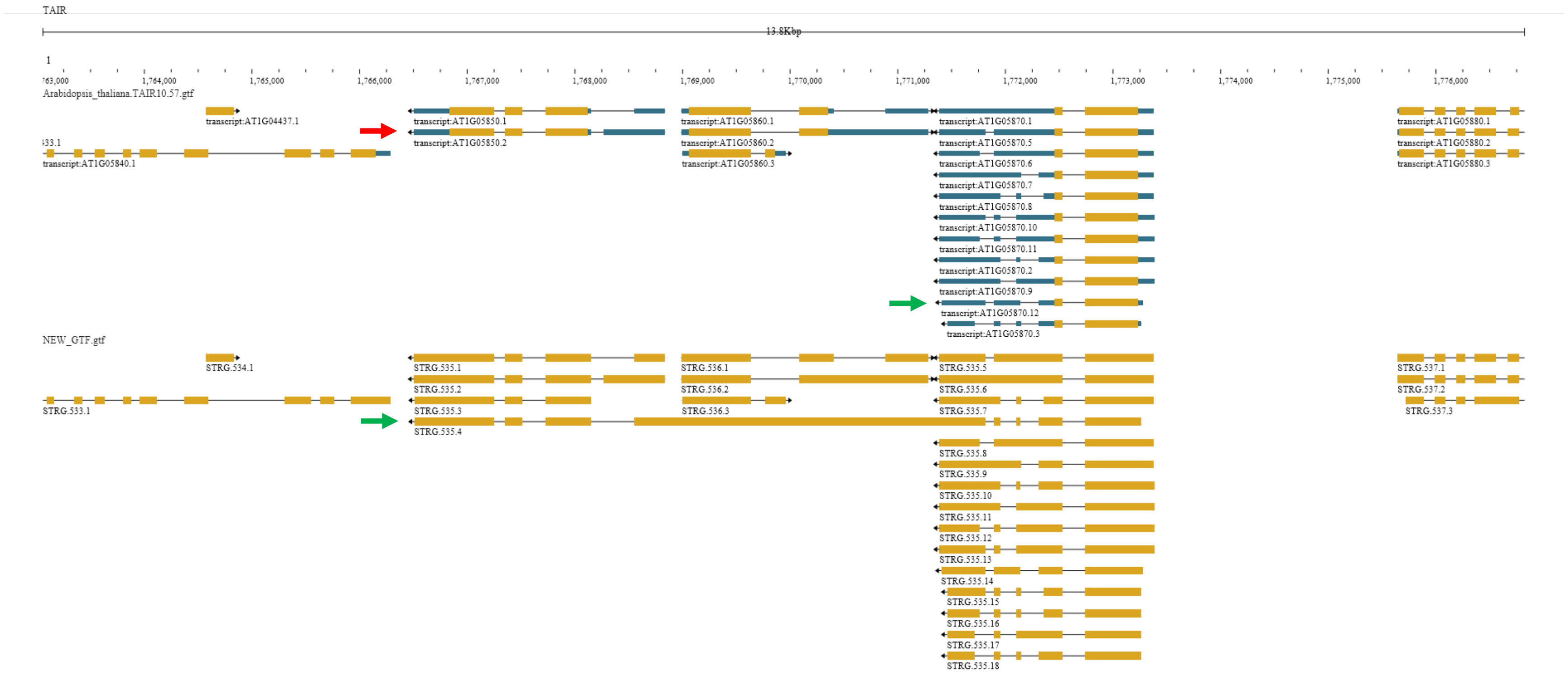

Figure S8. Structure of previously annotated and new DAS isoforms on XLOC_003623 (Gene 3, AT1G05850) generated due to different multifactorial stress combinations in the transcriptome datasets of 10-d-old *A. thaliana* (wild-type Col-0) seedlings. Red arrow refers to the gene isoform concordantly expressed with a given splicing factor, while green arrow(s) refer to other regulated isoforms of this gene. Other isoforms are not consistently regulated under the stress. One of the three isoforms (e.g., AT1G05870.12) belongs to another gene in this locus. The other two isoforms of the gene AT1G05850 show no clear case of alternative splicing, thus isoforms of this locus were not considered for further analysis. Further information is available in Tables S1, S3 and S6.

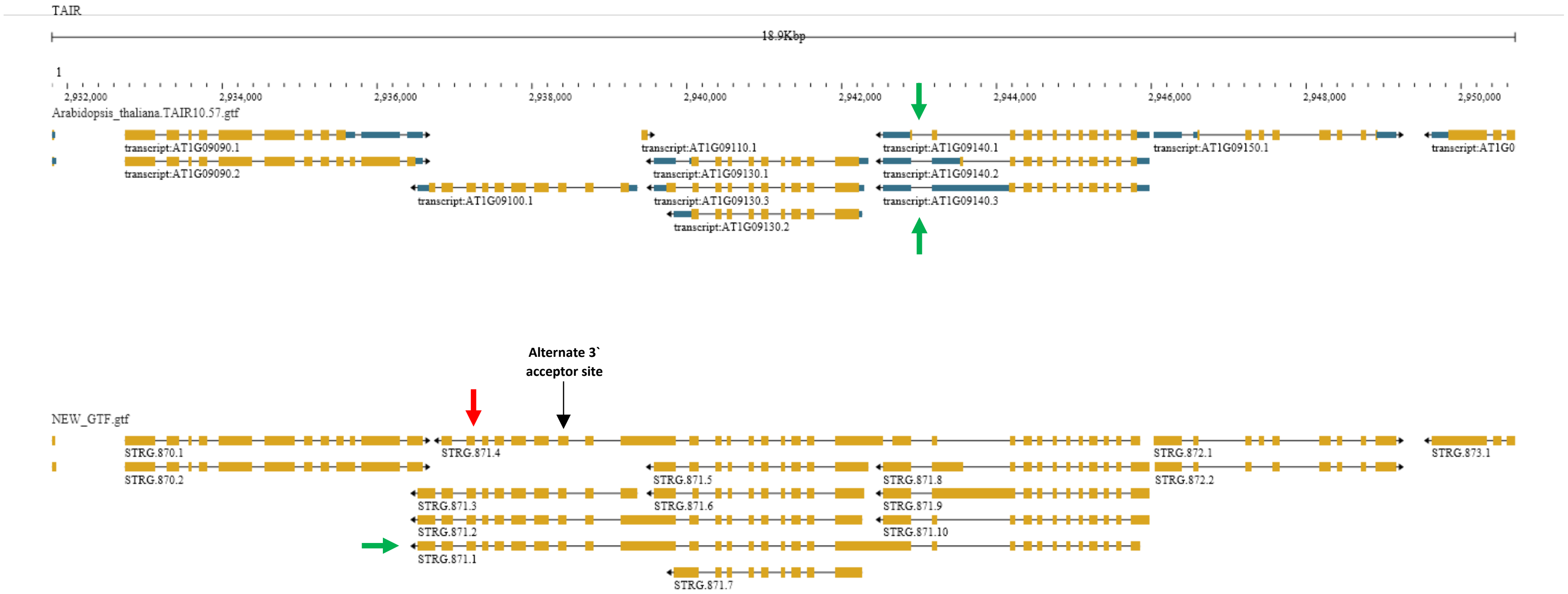

Figure S9. Structure of previously annotated and new DAS isoforms on XLOC_003799 (Gene 4, STRG.871) generated due to different multifactorial stress combinations in the transcriptome datasets of 10-d-old *A. thaliana* (wild-type Col-0) seedlings. Red arrow refers to the gene isoform concordantly expressed with a given splicing factor, while green arrow(s) refer to other regulated isoforms of this gene. Other isoforms are not consistently regulated under the stress. Further information is available in Tables S1, S3 and S6.

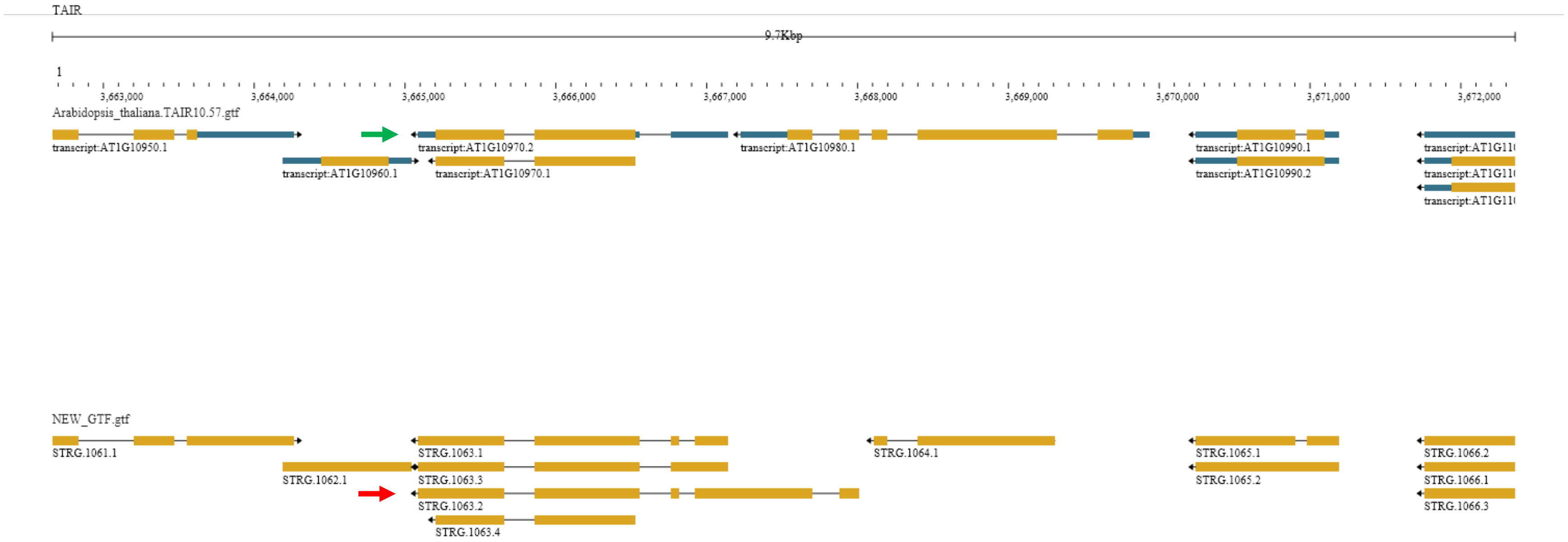

Figure S10. Structure of previously annotated and new DAS isoforms on XLOC_003907 (Gene 5, STRG.1063) generated due to different multifactorial stress combinations in the transcriptome datasets of 10-d-old *A. thaliana* (wild-type Col-0) seedlings. Red arrow refers to the gene isoform concordantly expressed with a given splicing factor, while green arrow(s) refer to other regulated isoforms of this gene. Other isoforms are not consistently regulated under the stress. Further information is available in Tables S1, S3 and S6.

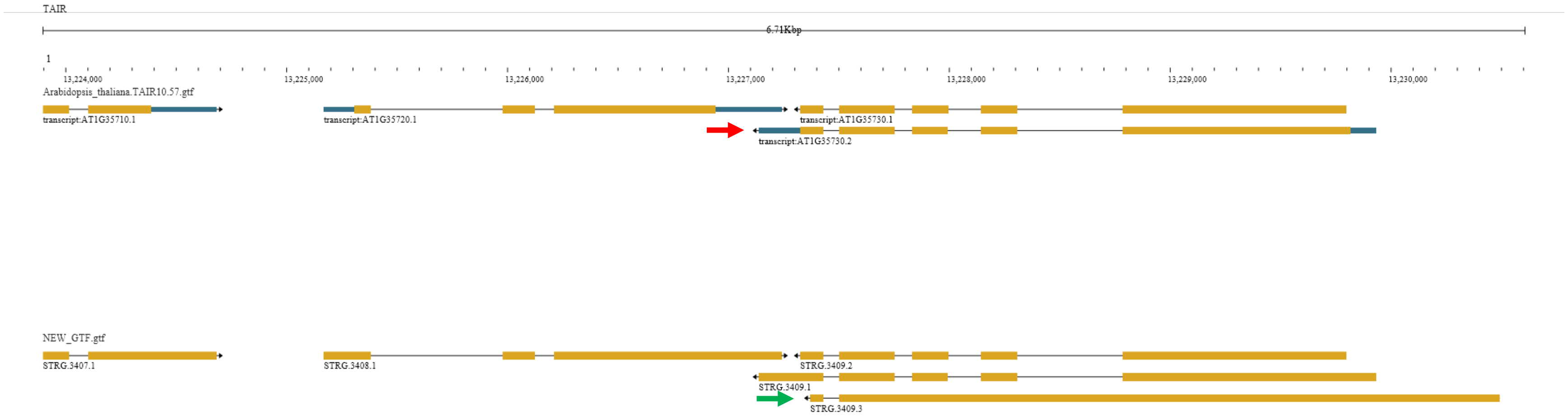

Figure S11. Structure of previously annotated and new DAS isoforms on XLOC_005026 (Gene 6, AT1G35730) generated due to different multifactorial stress combinations in the transcriptome datasets of 10-d-old *A. thaliana* (wild-type Col-0) seedlings. Red arrow refers to the gene isoform concordantly expressed with a given splicing factor, while green arrow(s) refer to other regulated isoforms of this gene. Other isoforms are not consistently regulated under the stress. Further information is available in Tables S1, S3 and S6.

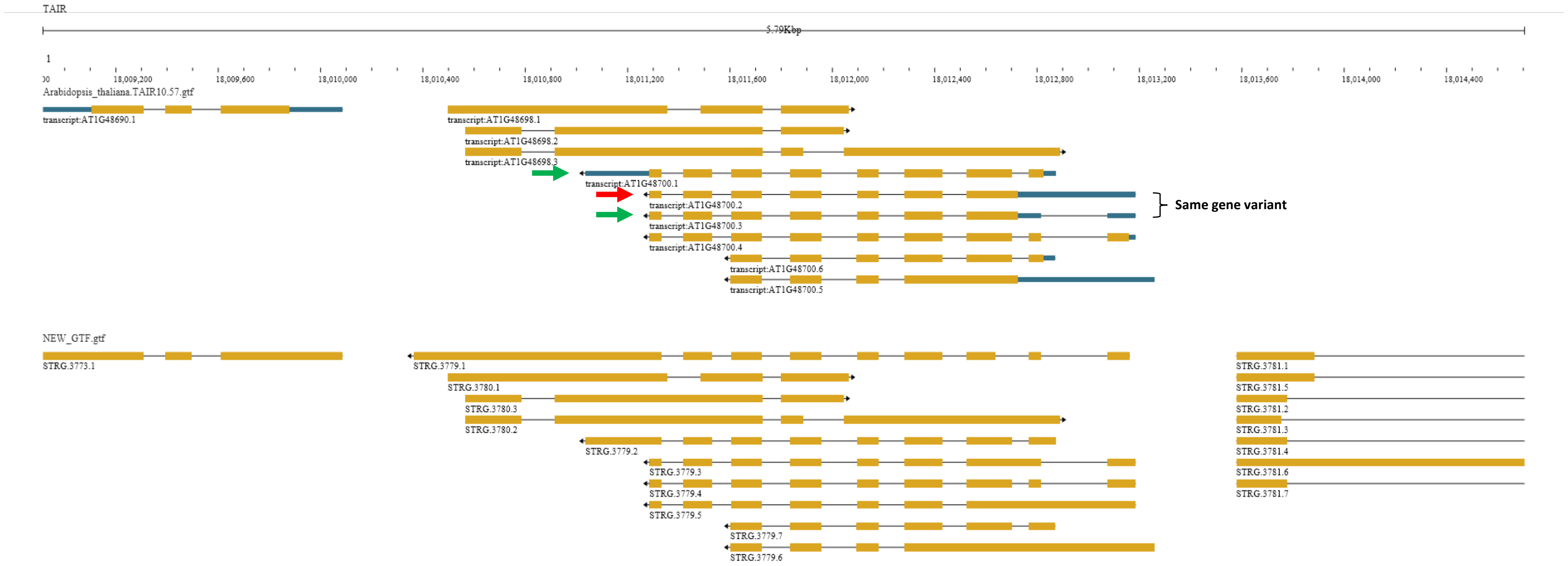

Figure S12. Structure of previously annotated and new DAS isoforms on XLOC_005205 (Gene 7, AT1G48700) generated due to different multifactorial stress combinations in the transcriptome datasets of 10-d-old *A. thaliana* (wild-type Col-0) seedlings. Red arrow refers to the gene isoform concordantly expressed with a given splicing factor, while green arrow(s) refer to other regulated isoforms of this gene. Other isoforms are not consistently regulated under the stress. Further information is available in Tables S1, S3 and S6.

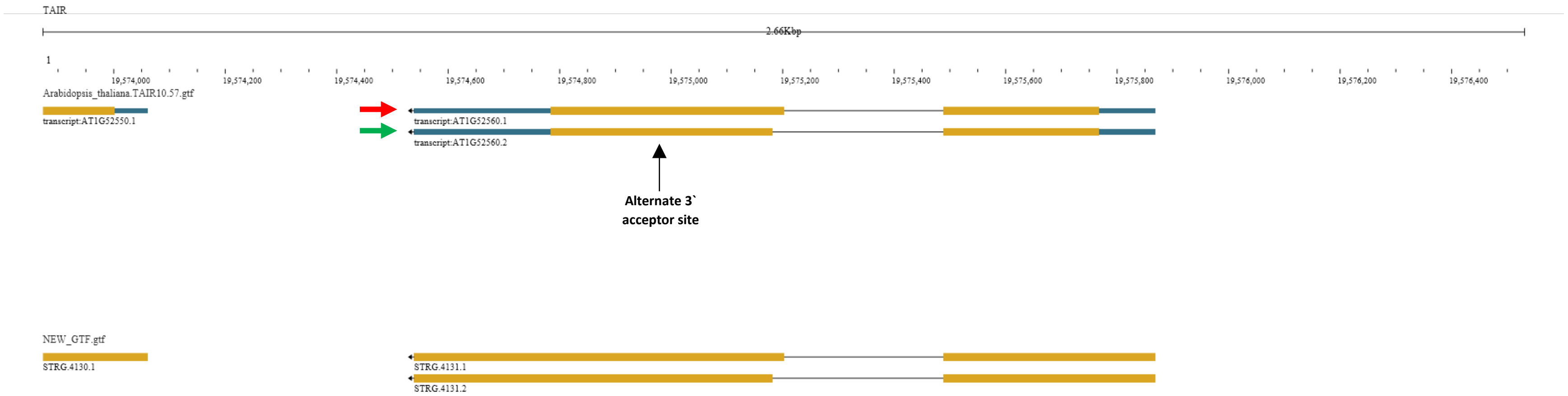

Figure S13. Structure of previously annotated and new DAS isoforms on XLOC_005378 (Gene 8, AT1G52560) generated due to different multifactorial stress combinations in the transcriptome datasets of 10-d-old *A. thaliana* (wild-type Col-0) seedlings. Red arrow refers to the gene isoform concordantly expressed with a given splicing factor, while green arrow(s) refer to other regulated isoforms of this gene. Other isoforms are not consistently regulated under the stress. Further information is available in Tables S1, S3 and S6.

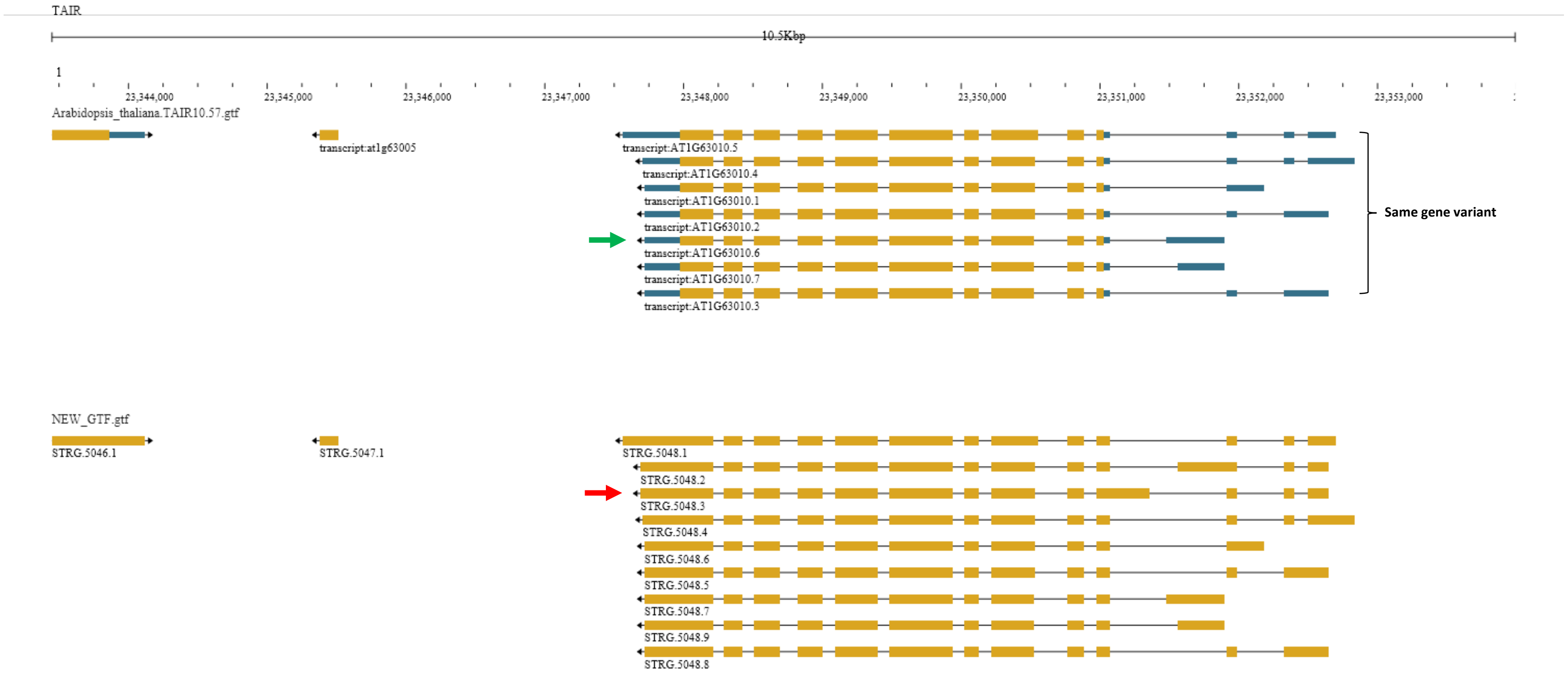

Figure S14. Structure of previously annotated and new DAS isoforms on XLOC_005837 (Gene 9, AT1G63010) generated due to different multifactorial stress combinations in the transcriptome datasets of 10-d-old *A. thaliana* (wild-type Col-0) seedlings. Red arrow refers to the gene isoform concordantly expressed with a given splicing factor, while green arrow(s) refer to other regulated isoforms of this gene. Other isoforms are not consistently regulated under the stress. Further information is available in Tables S1, S3 and S6.

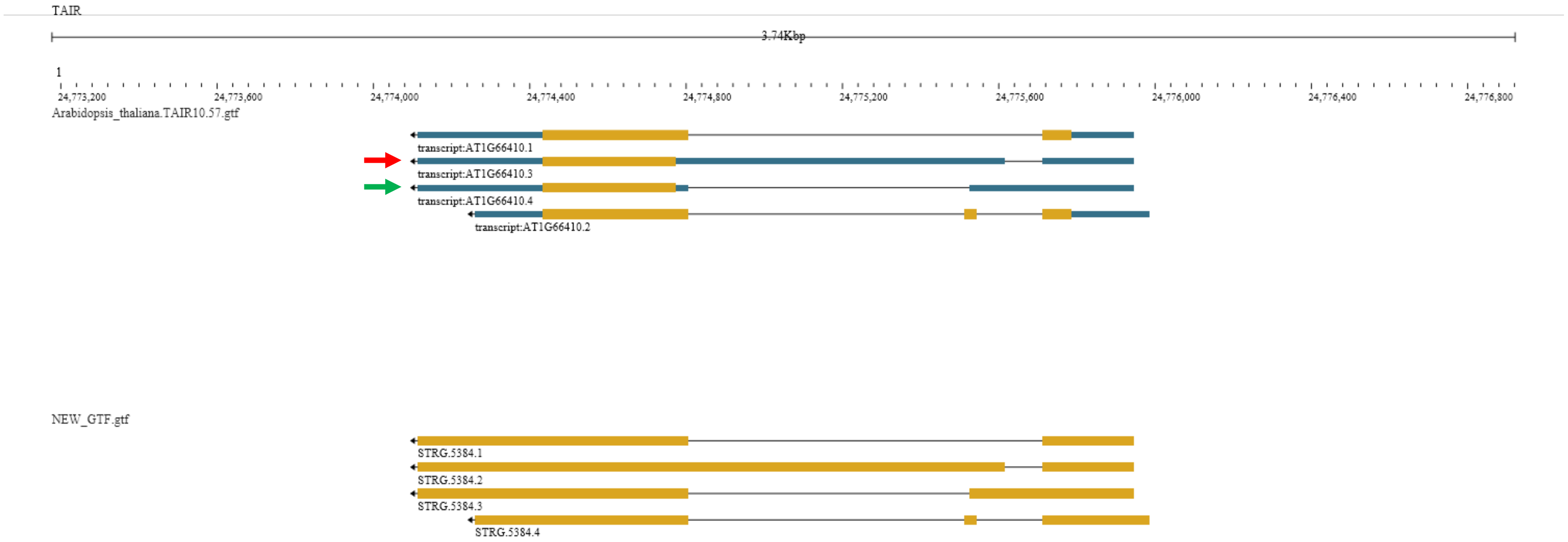

Figure S15. Structure of previously annotated and new DAS isoforms on XLOC_006004 (Gene 10, AT1G66410) generated due to different multifactorial stress combinations in the transcriptome datasets of 10-d-old *A. thaliana* (wild-type Col-0) seedlings. Red arrow refers to the gene isoform concordantly expressed with a given splicing factor, while green arrow(s) refer to other regulated isoforms of this gene. Other isoforms are not consistently regulated under the stress. Further information is available in Tables S1, S3 and S6.

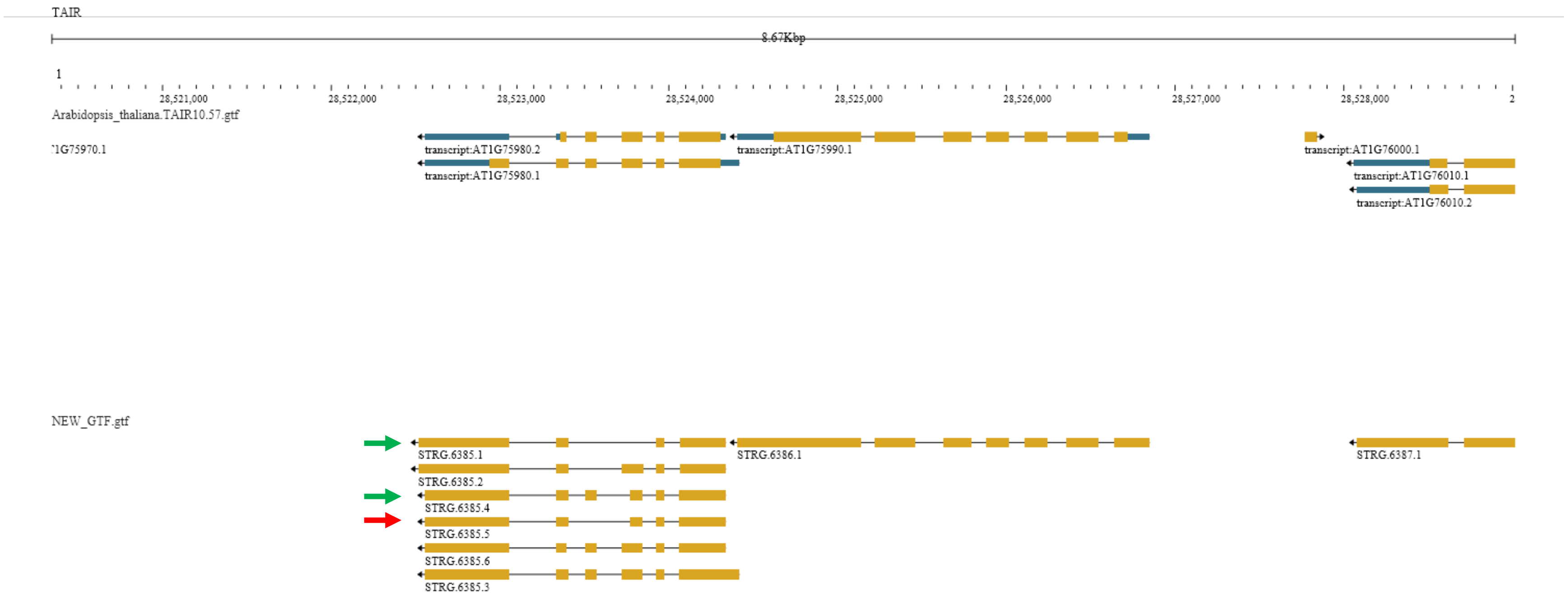

Figure S16. Structure of previously annotated and new DAS isoforms on XLOC_006475 (Gene 11, STRG.6385) generated due to different multifactorial stress combinations in the transcriptome datasets of 10-d-old *A. thaliana* (wild-type Col-0) seedlings. Red arrow refers to the gene isoform concordantly expressed with a given splicing factor, while green arrow(s) refer to other regulated isoforms of this gene. Other isoforms are not consistently regulated under the stress. Further information is available in Tables S1, S3 and S6.

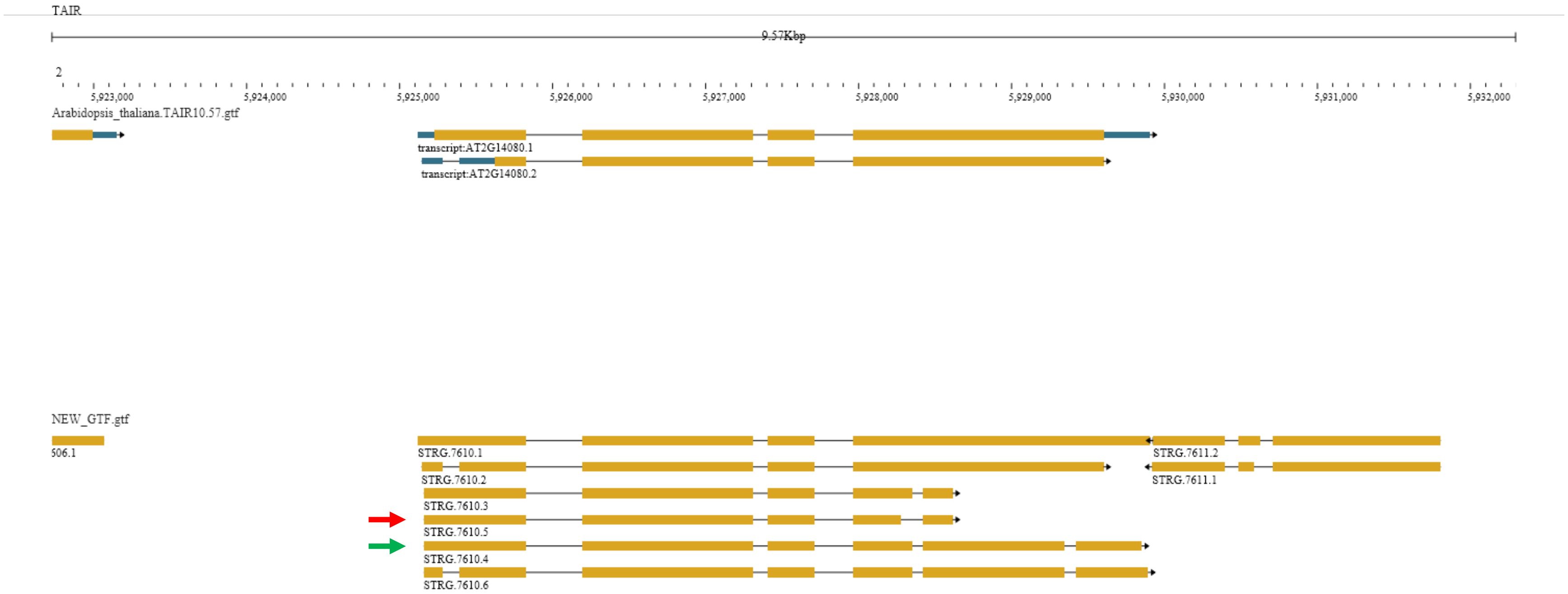

Figure S17. Structure of previously annotated and new SF isoforms on XLOC_007097 (SF3, AT2G14080) generated due to different multifactorial stress combinations in the transcriptome datasets of 10-d-old *A. thaliana* (wild-type Col-0) seedlings. Red arrow refers to the SF isoform concordantly expressed with a given gene, while green arrow(s) refer to other regulated isoforms of this gene. Other isoforms are not consistently regulated under the stress. Further information is available in Tables S1, S4 and S6.

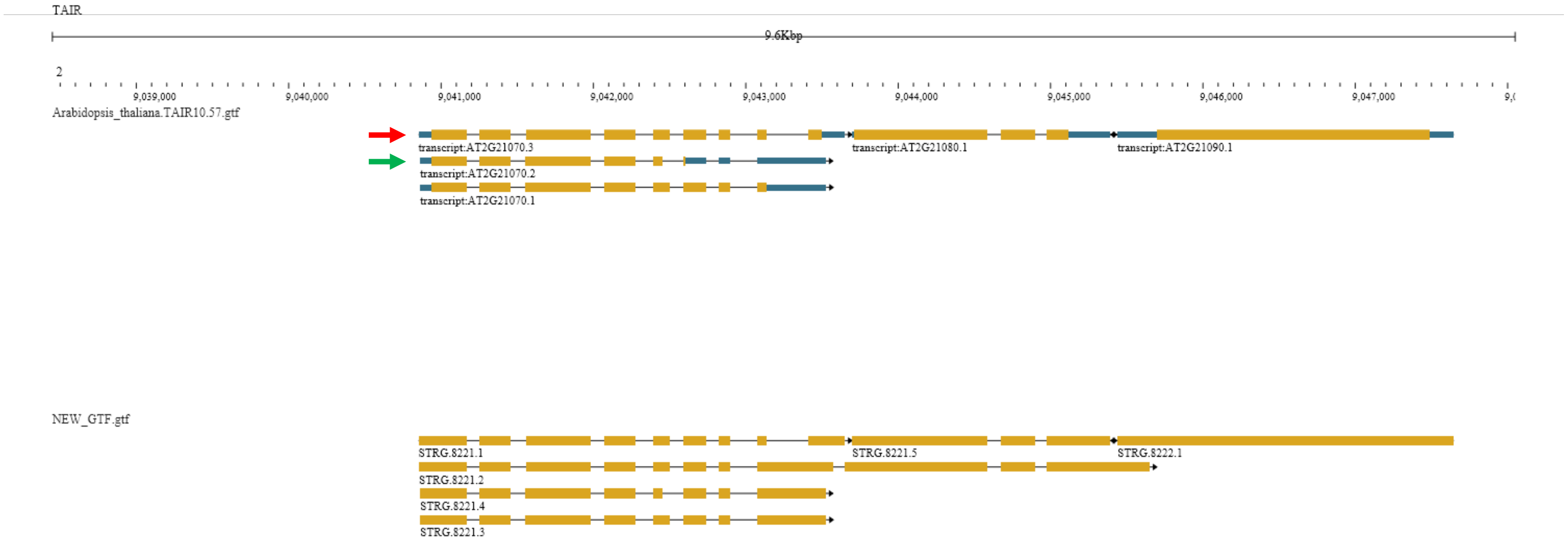

Figure S18. Structure of previously annotated and new DAS isoforms on XLOC_007392 (Gene 12, AT2G21070) generated due to different multifactorial stress combinations in the transcriptome datasets of 10-d-old *A. thaliana* (wild-type Col-0) seedlings. Red arrow refers to the gene isoform concordantly expressed with a given splicing factor, while green arrow(s) refer to other regulated isoforms of this gene. Other isoforms are not consistently regulated under the stress. Further information is available in Tables S1, S3 and S6.

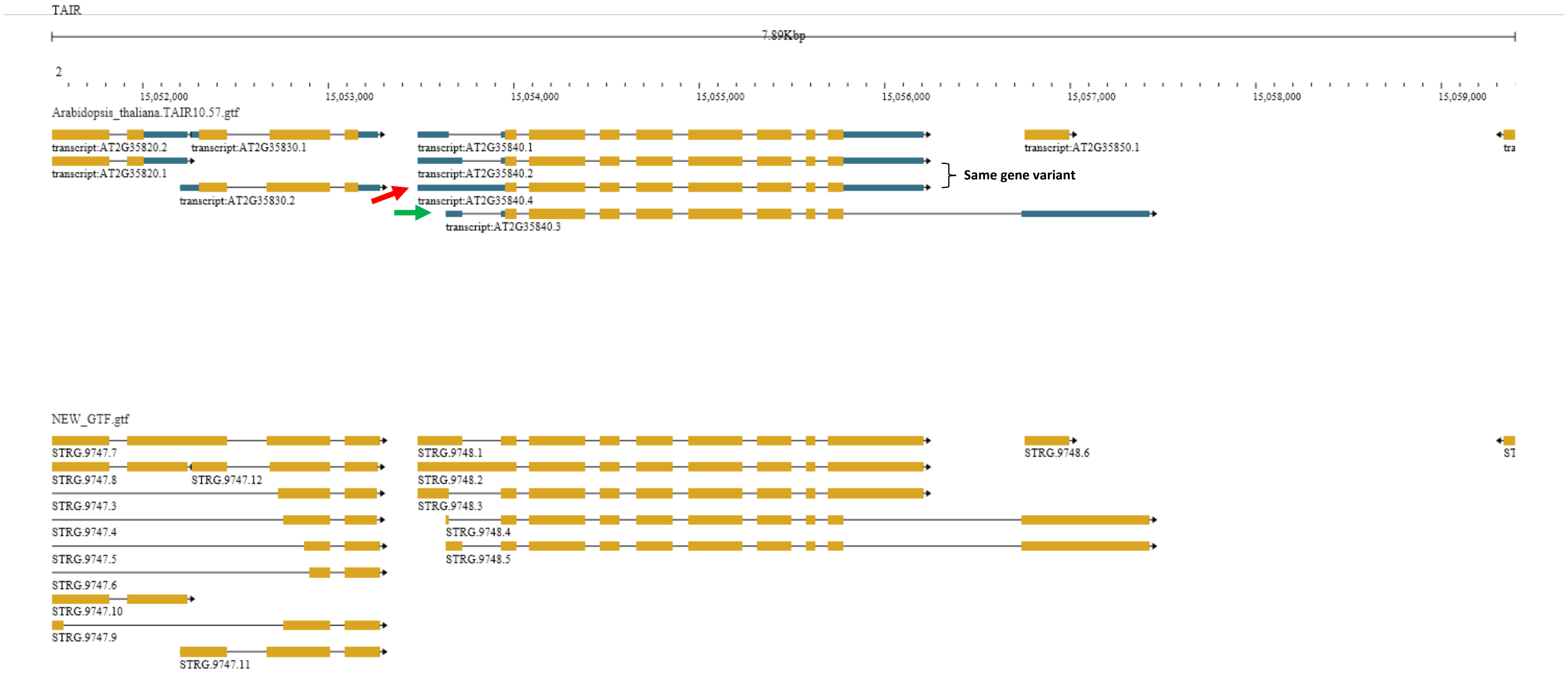

Figure S19. Structure of previously annotated and new DAS isoforms on XLOC_008122 (Gene 13, AT2G35840) generated due to different multifactorial stress combinations in the transcriptome datasets of 10-d-old *A. thaliana* (wild-type Col-0) seedlings. Red arrow refers to the gene isoform concordantly expressed with a given splicing factor, while green arrow(s) refer to other regulated isoforms of this gene. Red arrow refers to the gene isoform concordantly expressed with a given splicing factor, while green arrow(s) refer to other regulated isoforms of this gene. Other isoforms are not consistently regulated under the stress. Further information is available in Tables S1, S3 and S6.

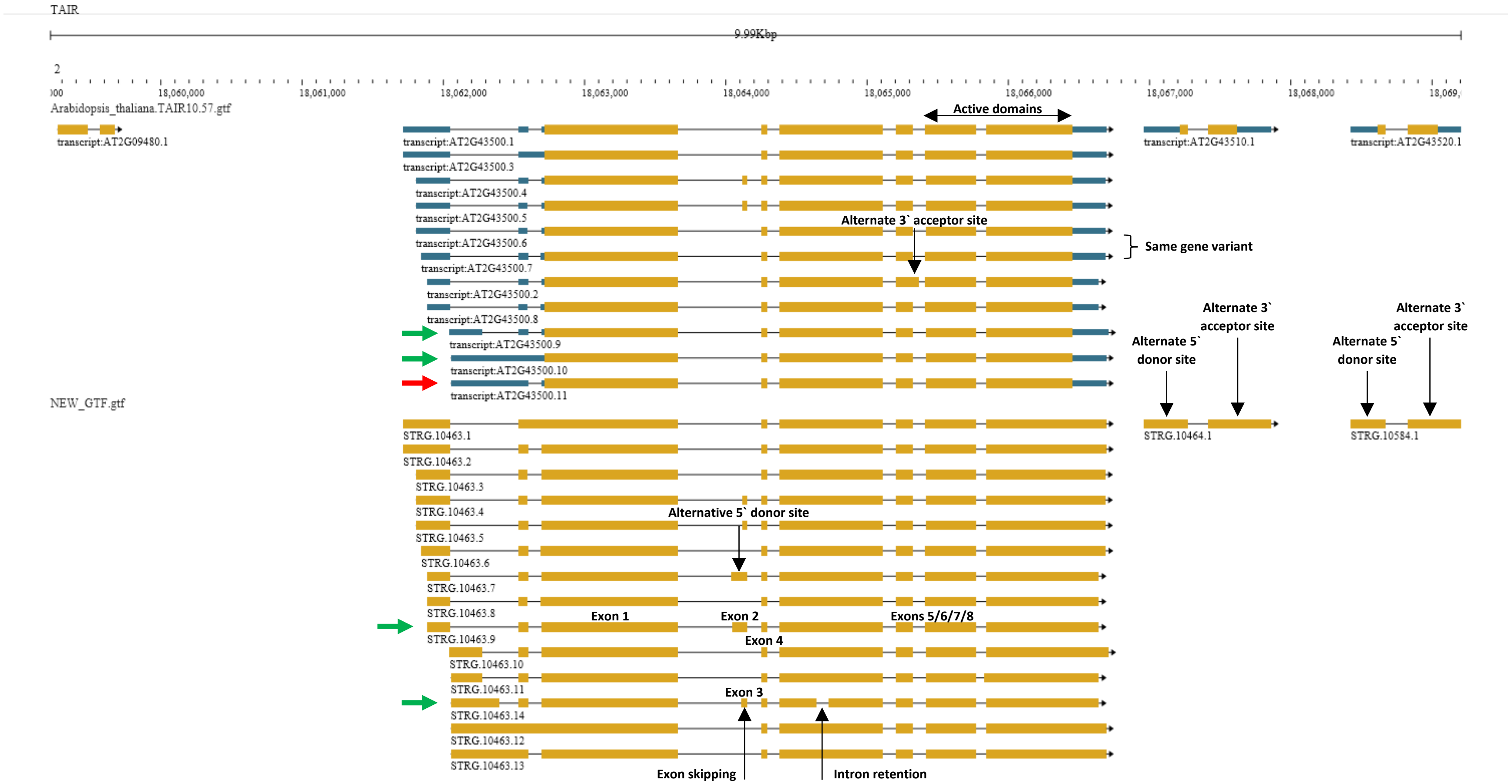

Figure S20. Structure of previously annotated and new DAS isoforms on XLOC_008527 (Gene 14, AT2G43500) generated due to different multifactorial stress combinations in the transcriptome datasets of 10-d-old *A. thaliana* (wild-type Col-0) seedlings. Red arrow refers to the gene isoform concordantly expressed with a given splicing factor, while green arrow(s) refer to other regulated isoforms of this gene. Other isoforms are not consistently regulated under the stress. Further information is available in Tables S1, S3 and S6. The two events of alternative splicing, e.g., exon skipping and intron retention, were deeply investigated. Active conserved domains in the generated protein were detected based on recent information (Ge et al., 2018).

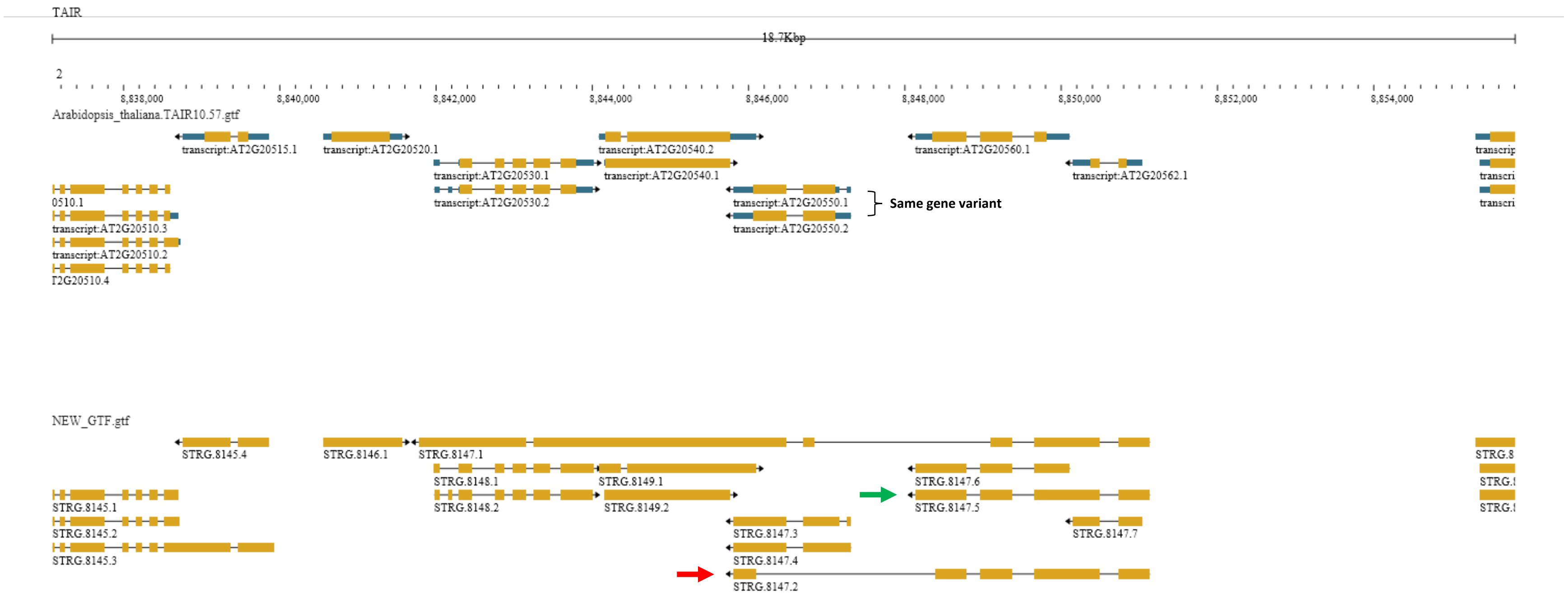

Figure S21. Structure of previously annotated and new DAS isoforms on XLOC_009386 (Gene 15, STRG.8147) generated due to different multifactorial stress combinations in the transcriptome datasets of 10-d-old *A. thaliana* (wild-type Col-0) seedlings. Red arrow refers to the gene isoform concordantly expressed with a given splicing factor, while green arrow(s) refer to other regulated isoforms of this gene. Other isoforms are not consistently regulated under the stress. Further information is available in Tables S1, S3 and S6.

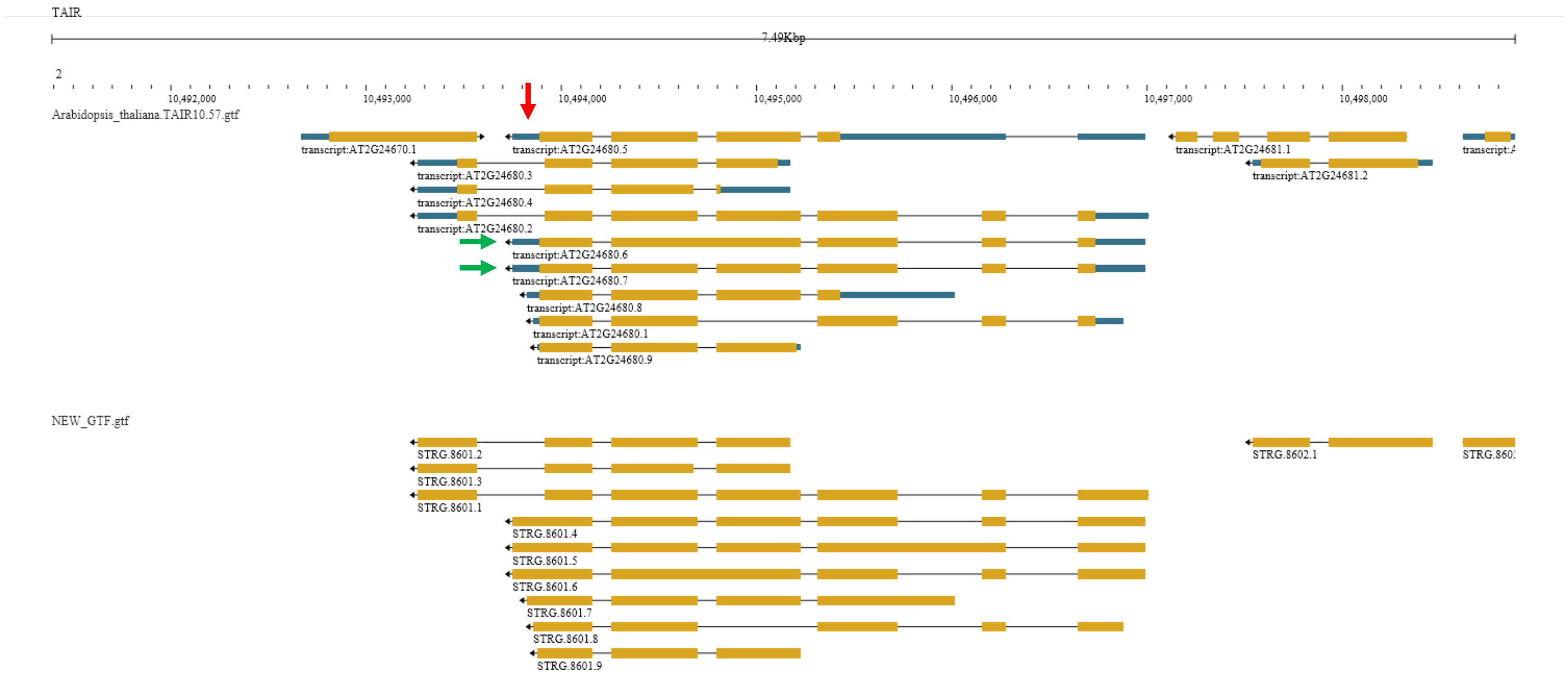

Figure S22. Structure of previously annotated and new DAS isoforms on XLOC_009607 (Gene 16, AT2G24680) generated due to different multifactorial stress combinations in the transcriptome datasets of 10-d-old *A. thaliana* (wild-type Col-0) seedlings. Red arrow refers to the gene isoform concordantly expressed with a given splicing factor, while green arrow(s) refer to other regulated isoforms of this gene. Other isoforms are not consistently regulated under the stress. Further information is available in Tables S1, S3 and S6.

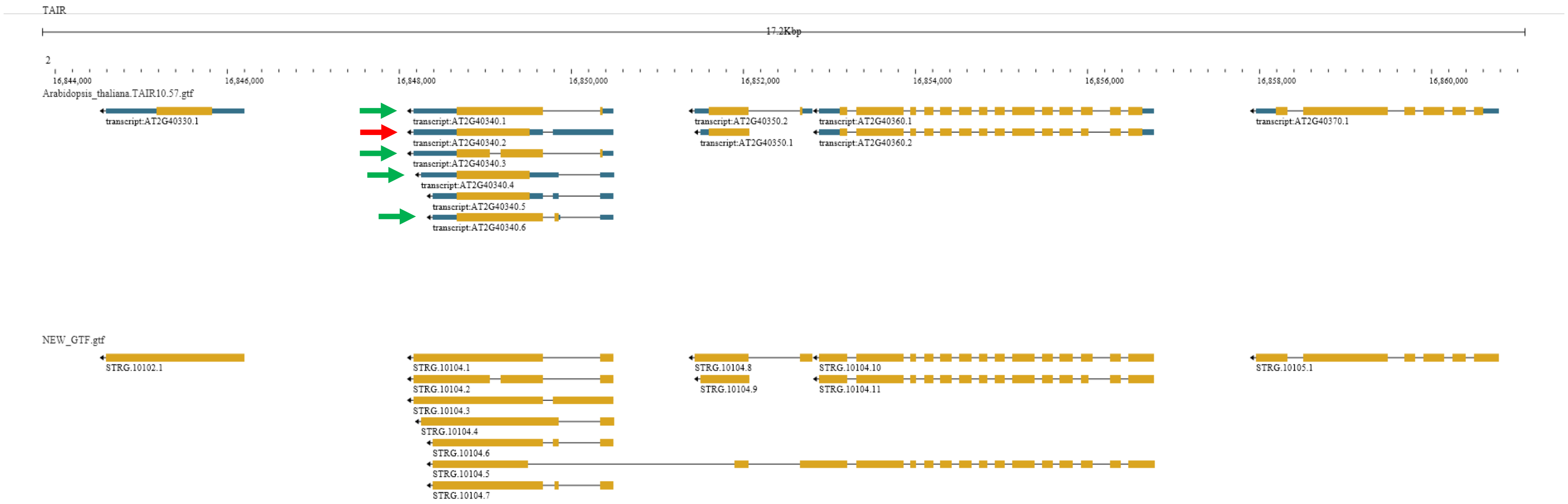

Figure S23. Structure of previously annotated and new DAS isoforms on XLOC_010435 (Gene 17, AT2G40340) generated due to different multifactorial stress combinations in the transcriptome datasets of 10-d-old *A. thaliana* (wild-type Col-0) seedlings. Red arrow refers to the gene isoform concordantly expressed with a given splicing factor, while green arrow(s) refer to other regulated isoforms of this gene. Other isoforms are not consistently regulated under the stress. Further information is available in Tables S1, S3 and S6.

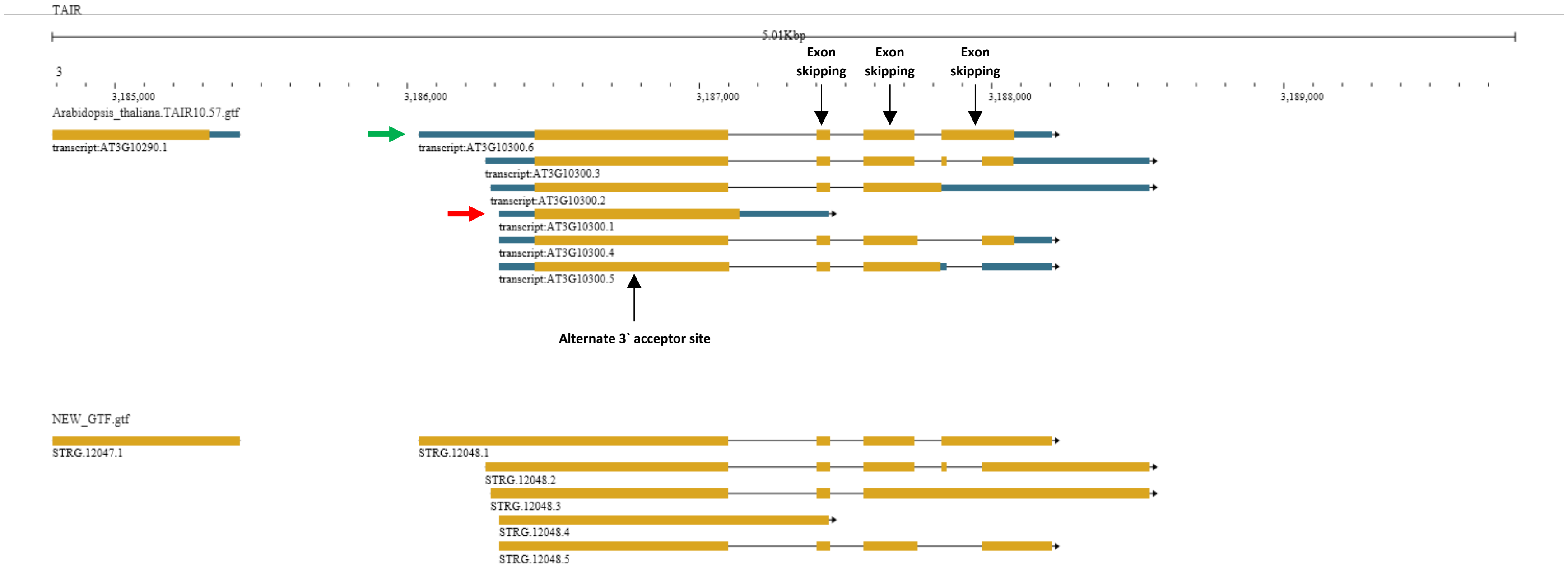

Figure S24. Structure of previously annotated and new DAS isoforms on XLOC_011288 (Gene 18, AT3G10300) generated due to different multifactorial stress combinations in the transcriptome datasets of 10-d-old *A. thaliana* (wild-type Col-0) seedlings. Red arrow refers to the gene isoform concordantly expressed with a given splicing factor, while green arrow(s) refer to other regulated isoforms of this gene. Other isoforms are not consistently regulated under the stress. Further information is available in Tables S1, S3 and S6.

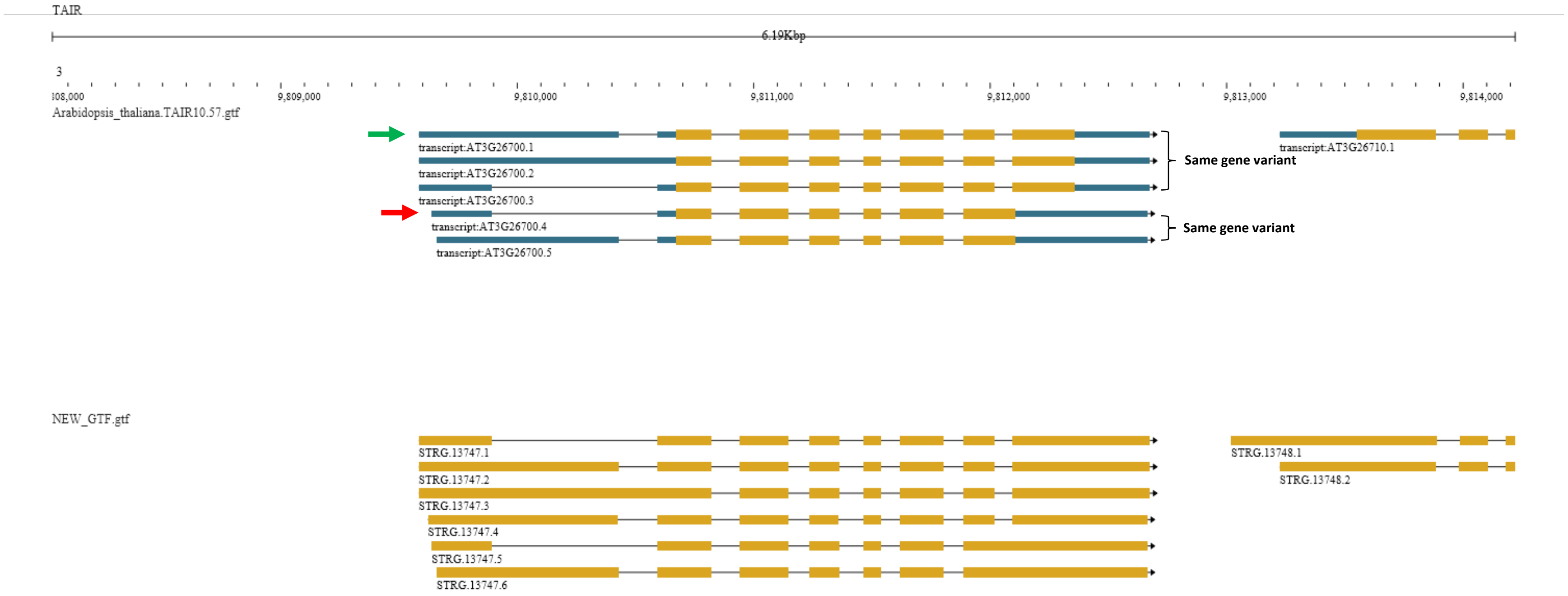

Figure S25. Structure of previously annotated and new DAS isoforms on XLOC_012141 (Gene 19, AT3G26700) generated due to different multifactorial stress combinations in the transcriptome datasets of 10-d-old *A. thaliana* (wild-type Col-0) seedlings. Red arrow refers to the gene isoform concordantly expressed with a given splicing factor, while green arrow(s) refer to other regulated isoforms of this gene. Other isoforms are not consistently regulated under the stress. Further information is available in Tables S1, S3 and S6.

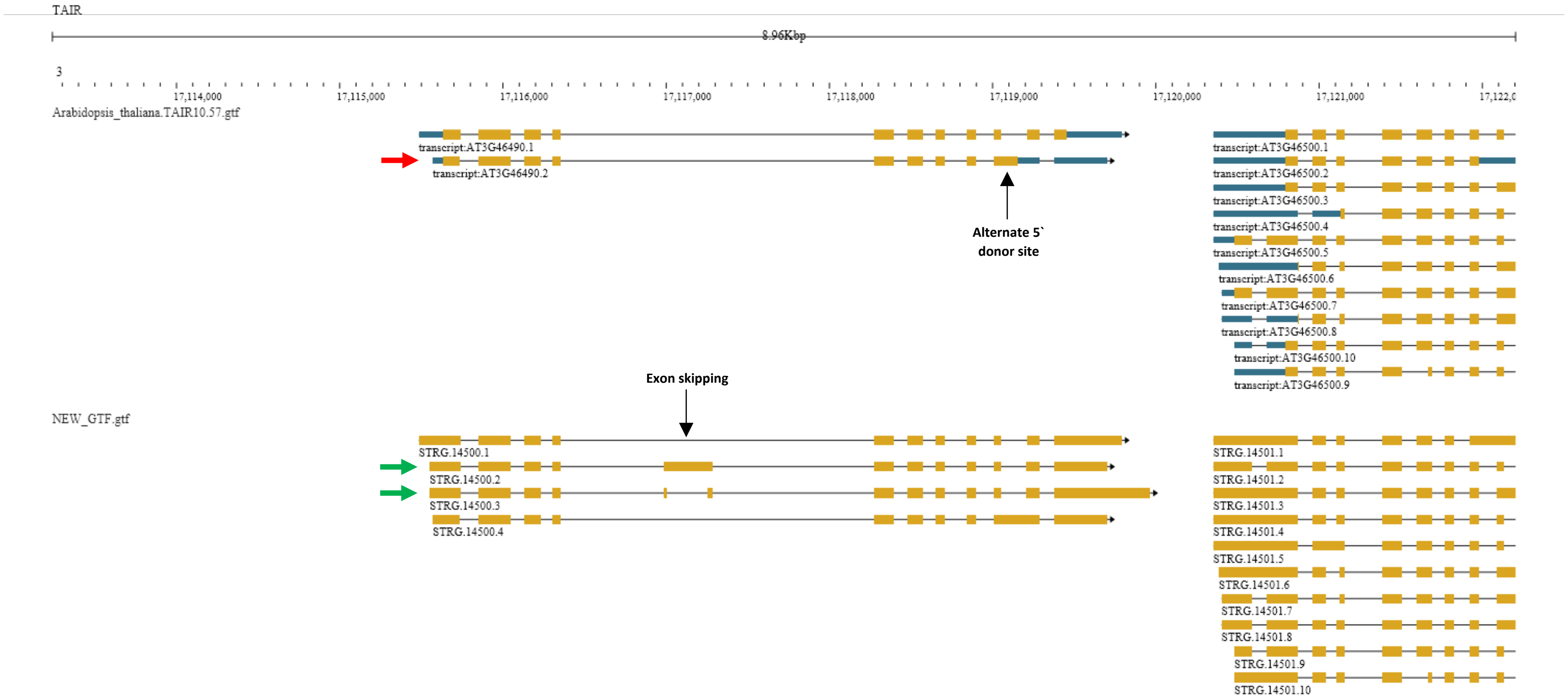

Figure S26. Structure of previously annotated and new SF isoforms on XLOC_012492 (SF5, AT3G46490) generated due to different multifactorial stress combinations in the transcriptome datasets of 10-d-old *A. thaliana* (wild-type Col-0) seedlings. Red arrow refers to the SF isoform concordantly expressed with a given gene, while green arrow(s) refer to other regulated isoforms of this gene. Other isoforms are not consistently regulated under the stress. Further information is available in Tables S1, S4 and S6.

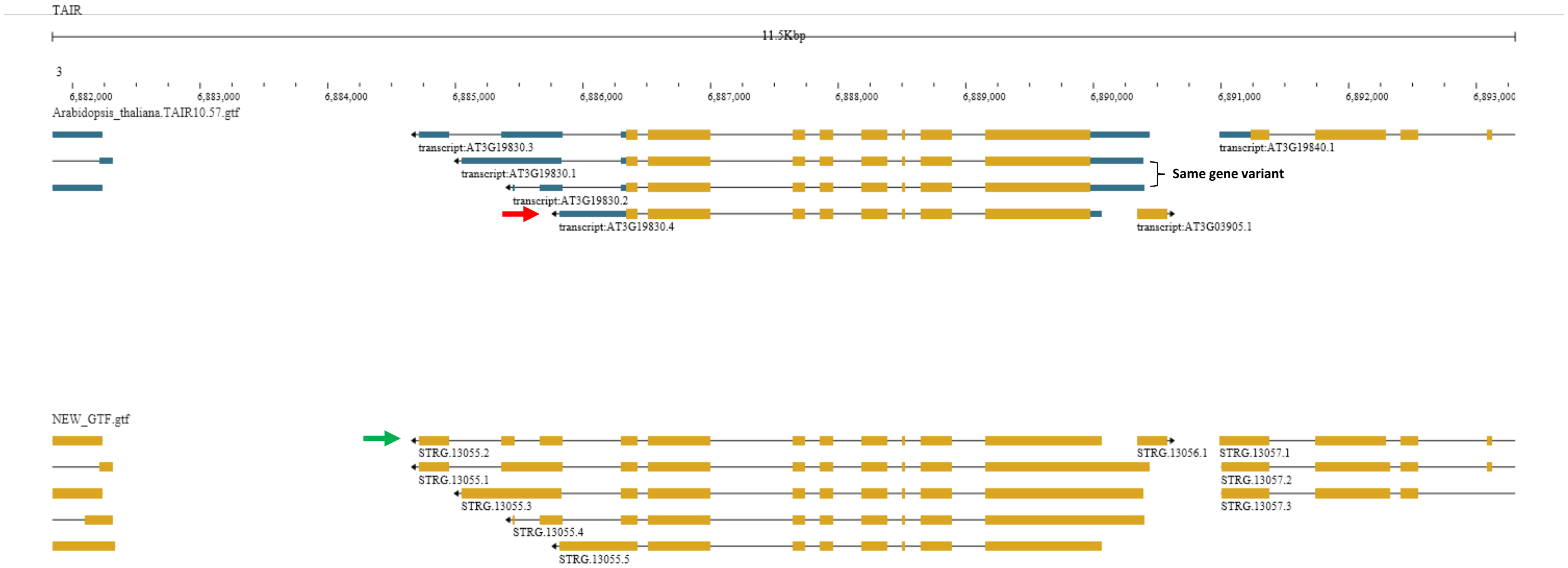

Figure S27. Structure of previously annotated and new DAS isoforms on XLOC_014308 (Gene 20, AT3G19830) generated due to different multifactorial stress combinations in the transcriptome datasets of 10-d-old *A. thaliana* (wild-type Col-0) seedlings. Red arrow refers to the gene isoform concordantly expressed with a given splicing factor, while green arrow(s) refer to other regulated isoforms of this gene. Other isoforms are not consistently regulated under the stress. Further information is available in Tables S1, S3 and S6.

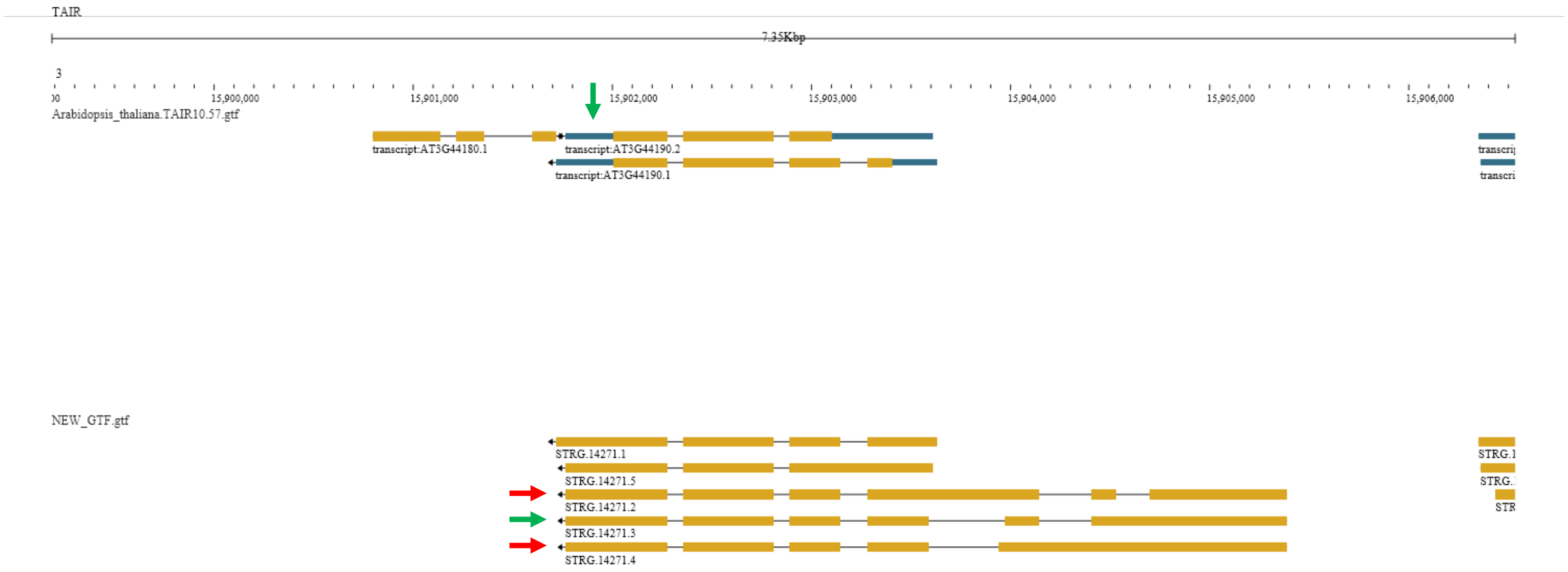

Figure S28. Structure of previously annotated and new DAS isoforms on XLOC_014905 (Gene 21, STRG.14271) generated due to different multifactorial stress combinations in the transcriptome datasets of 10-d-old *A. thaliana* (wild-type Col-0) seedlings. Red arrow refers to the gene isoform concordantly expressed with a given splicing factor, while green arrow(s) refer to other regulated isoforms of this gene. Other isoforms are not consistently regulated under the stress. Further information is available in Tables S1, S3 and S6.

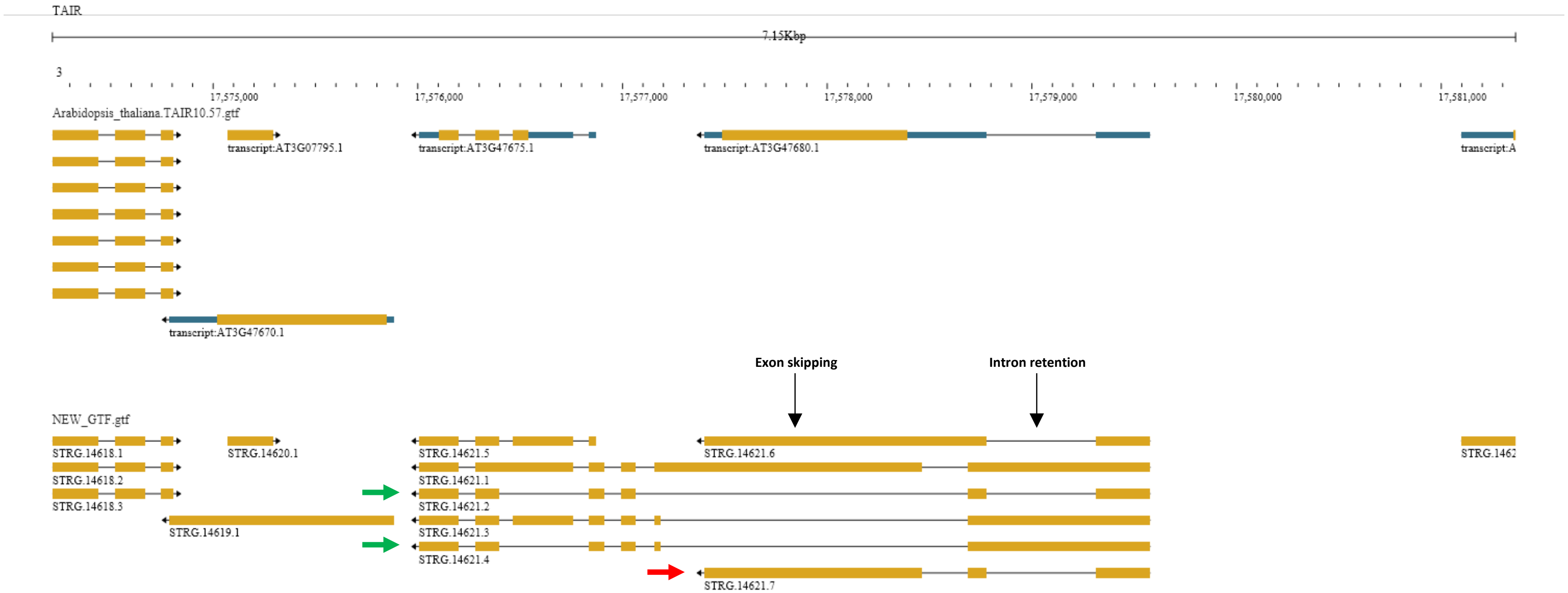

Figure S29. Structure of previously annotated and new DAS isoforms on XLOC_015088 (Gene 22, STRG.14621) generated due to different multifactorial stress combinations in the transcriptome datasets of 10-d-old *A. thaliana* (wild-type Col-0) seedlings. Red arrow refers to the gene isoform concordantly expressed with a given splicing factor, while green arrow(s) refer to other regulated isoforms of this gene. Other isoforms are not consistently regulated under the stress. Further information is available in Tables S1, S3 and S6.

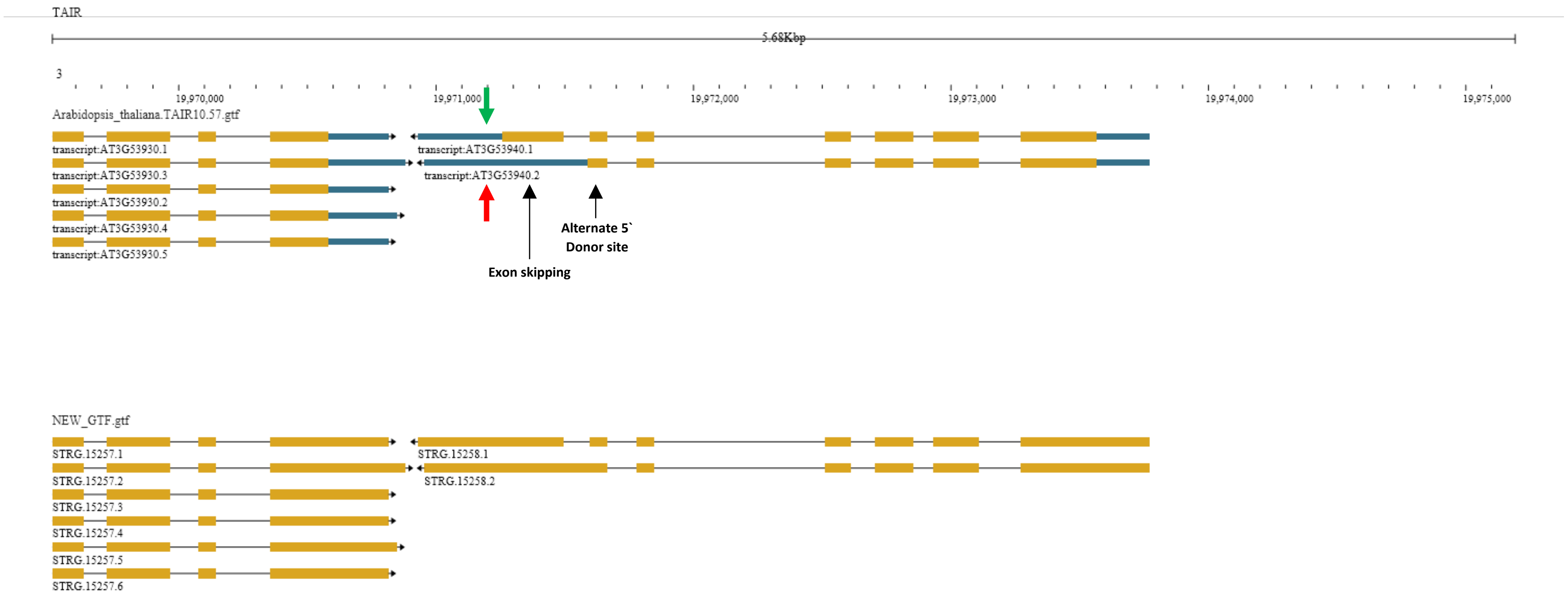

Figure S30. Structure of previously annotated and new DAS isoforms on XLOC_015398 (Gene 23, AT3G53940) generated due to different multifactorial stress combinations in the transcriptome datasets of 10-d-old *A. thaliana* (wild-type Col-0) seedlings. Red arrow refers to the gene isoform concordantly expressed with a given splicing factor, while green arrow(s) refer to other regulated isoforms of this gene. Other isoforms are not consistently regulated under the stress. Further information is available in Tables S1, S3 and S6.

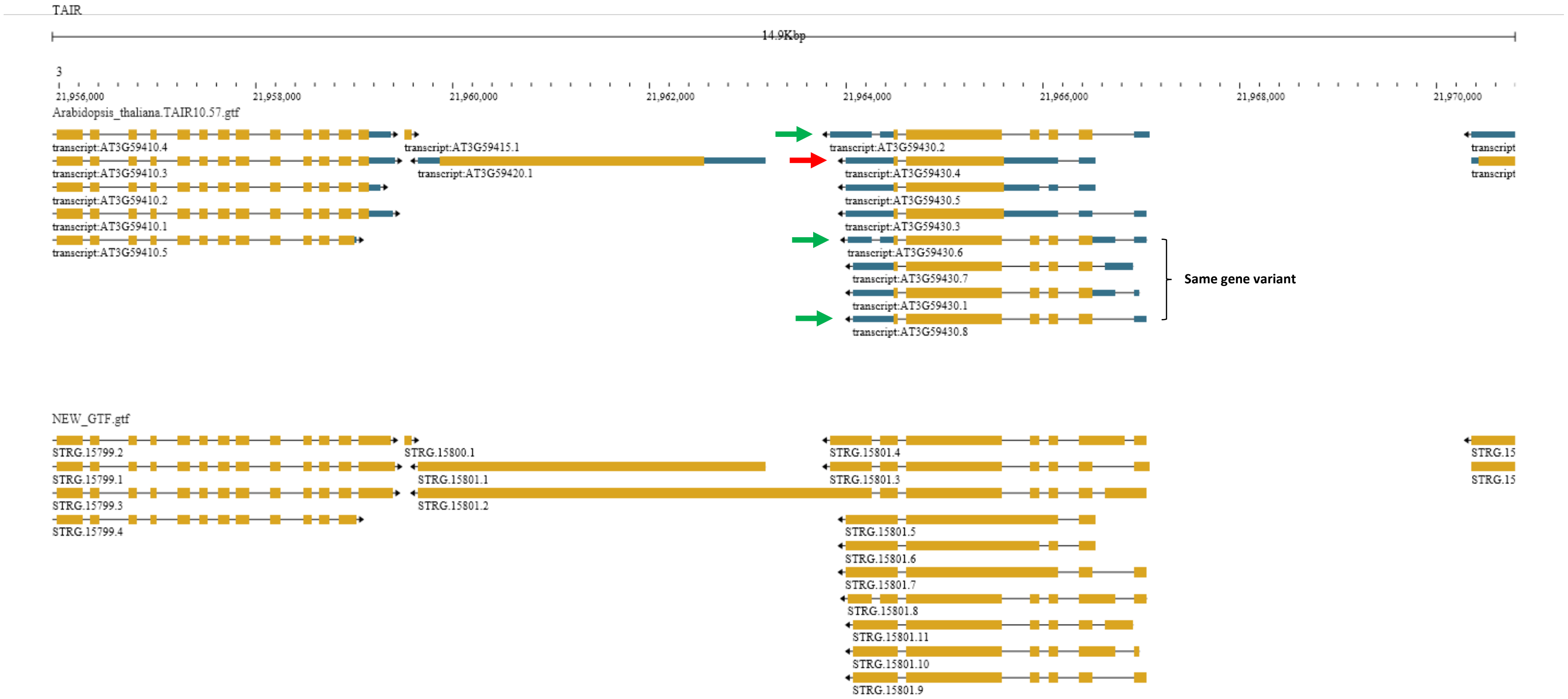

Figure S31. Structure of previously annotated and new DAS isoforms on XLOC_015679 (Gene 24, AT3G59430) generated due to different multifactorial stress combinations in the transcriptome datasets of 10-d-old *A. thaliana* (wild-type Col-0) seedlings. Red arrow refers to the gene isoform concordantly expressed with a given splicing factor, while green arrow(s) refer to other regulated isoforms of this gene. Other isoforms are not consistently regulated under the stress. Further information is available in Tables S1, S3 and S6.

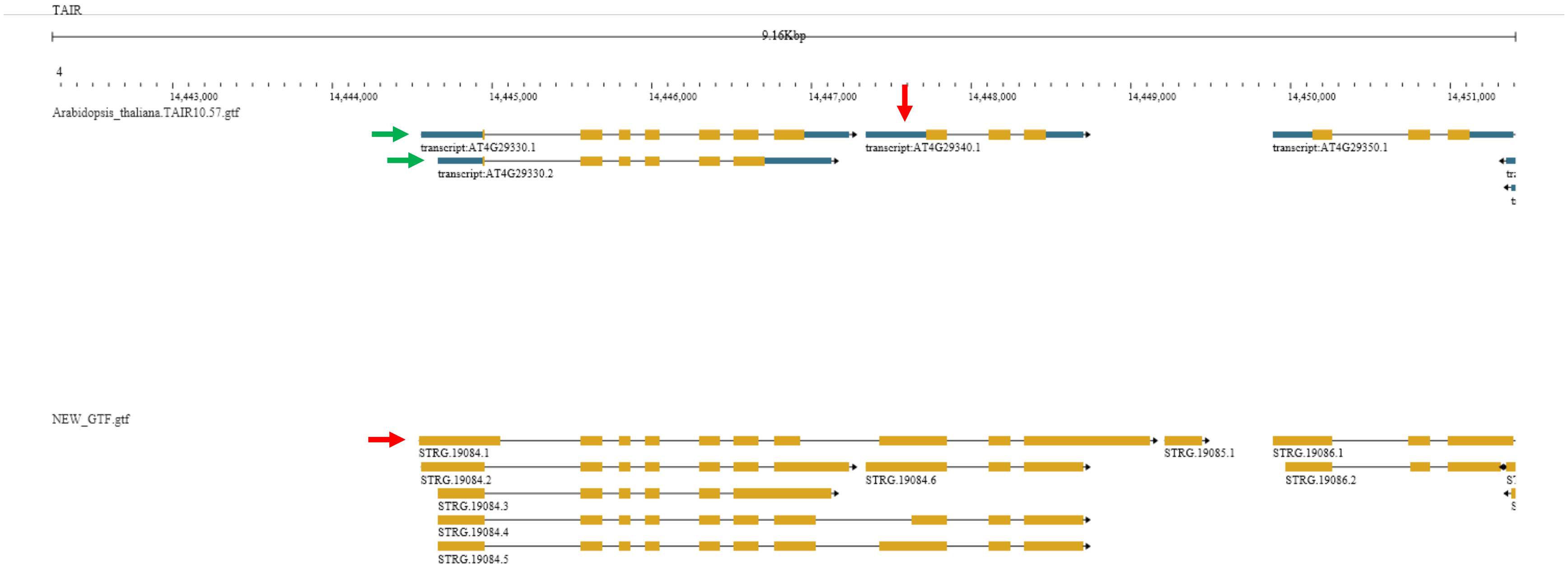

Figure S32. Structure of previously annotated and new DAS isoforms on XLOC_017325 (Gene 25, AT4G29340/ STRG.19084) generated due to different multifactorial stress combinations in the transcriptome datasets of 10-d-old *A. thaliana* (wild-type Col-0) seedlings. Red arrow refers to the gene isoform concordantly expressed with a given splicing factor, while green arrow(s) refer to other regulated isoforms of this gene. Other isoforms are not consistently regulated under the stress. Further information is available in Tables S1, S3 and S6.

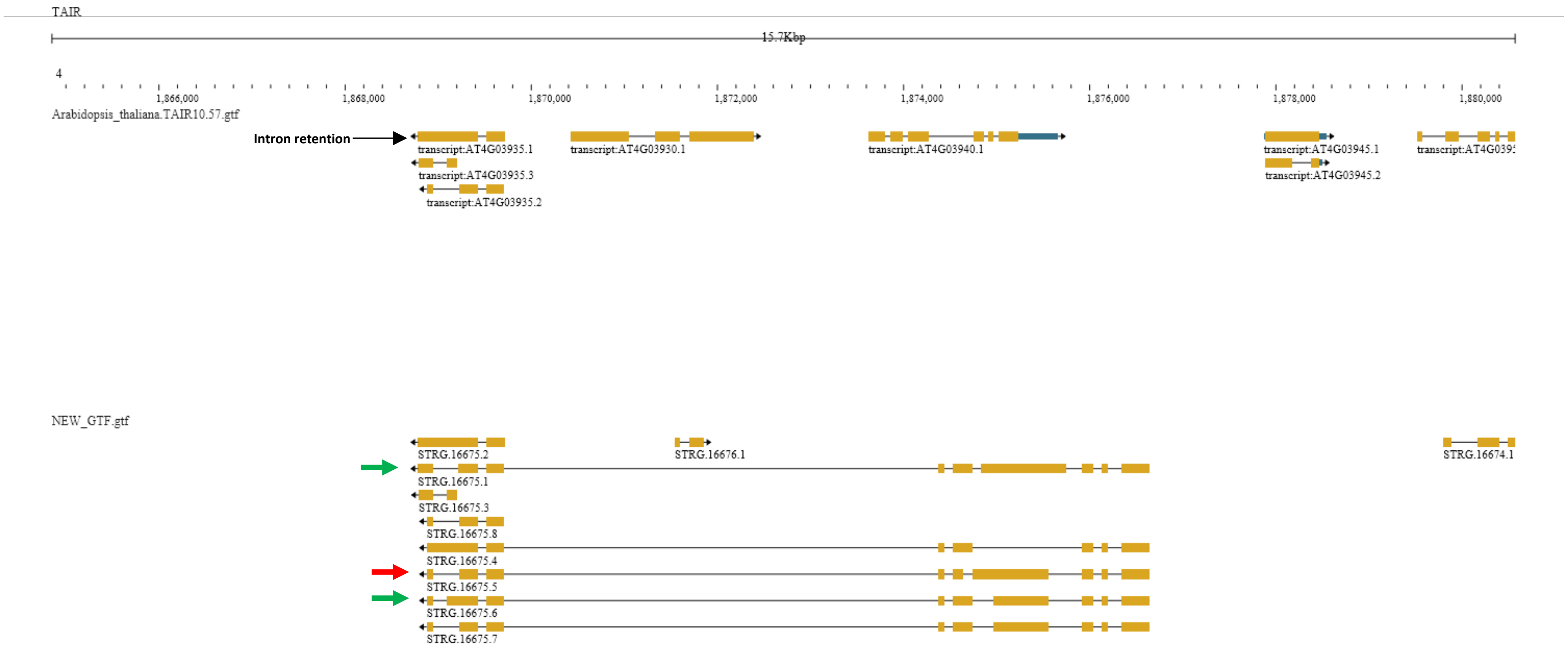

Figure S33. Structure of previously annotated and new DAS isoforms on XLOC_018080 (Gene 26, STRG.16675) generated due to different multifactorial stress combinations in the transcriptome datasets of 10-d-old *A. thaliana* (wild-type Col-0) seedlings. Red arrow refers to the gene isoform concordantly expressed with a given splicing factor, while green arrow(s) refer to other regulated isoforms of this gene. Other isoforms are not consistently regulated under the stress. Further information is available in Tables S1, S3 and S6.

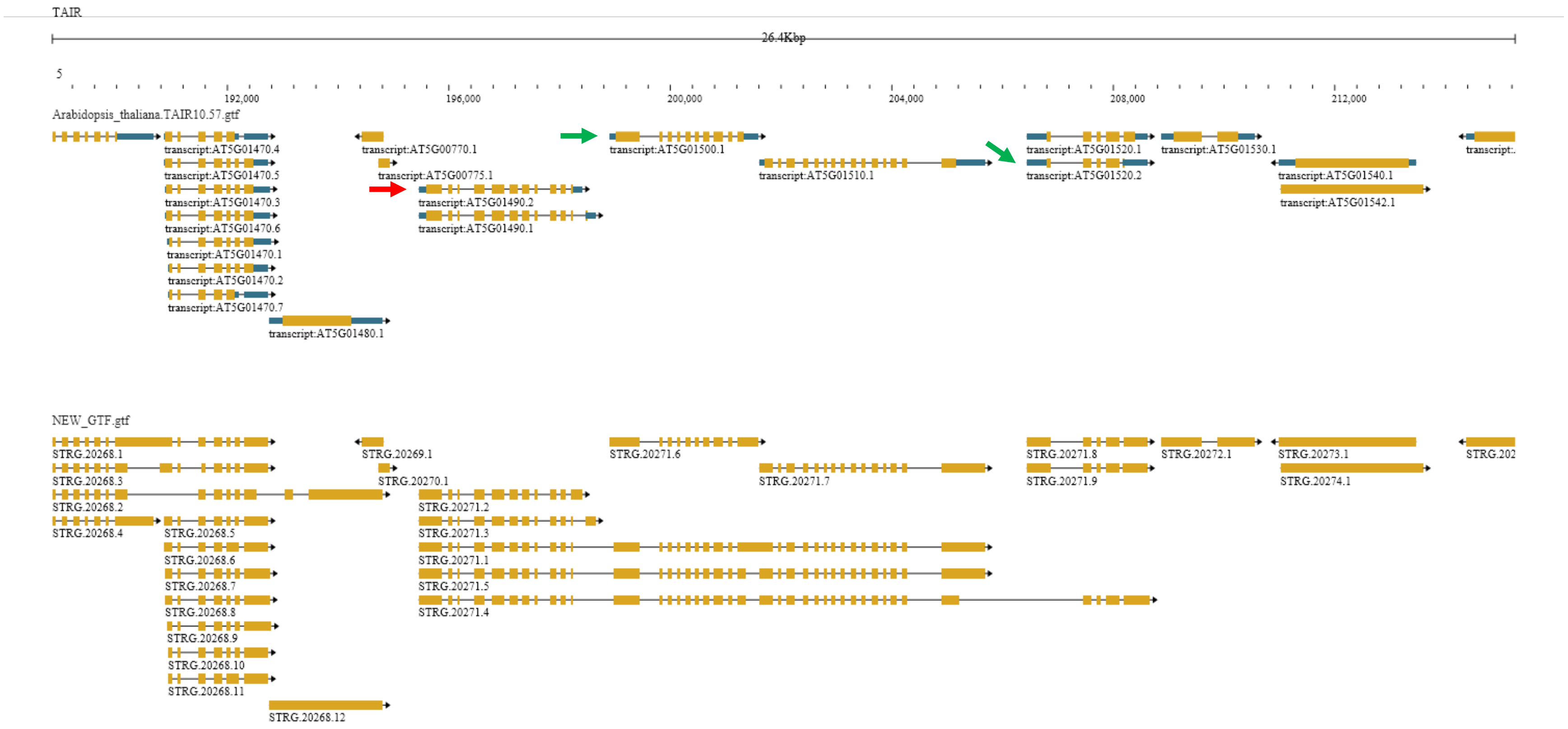

Figure S34. Structure of previously annotated and new DAS isoforms on XLOC_019807 (Gene 27, AT5G01490/AT5G01500/AT5G01520) generated due to different multifactorial stress combinations in the transcriptome datasets of 10-d-old *A. thaliana* (wild-type Col-0) seedlings. Red arrow refers to the gene isoform concordantly expressed with a given splicing factor, while green arrow(s) refer to other regulated isoforms of this gene. Other isoforms are not consistently regulated under the stress. Further information is available in Tables S1, S3 and S6. Note that the three isoforms belong to different genes, thus, the concordantly expressed isoform (e.g., AT5G01490.2) with SF5 (e.g., AT3G46490.2) was not analyzed further.

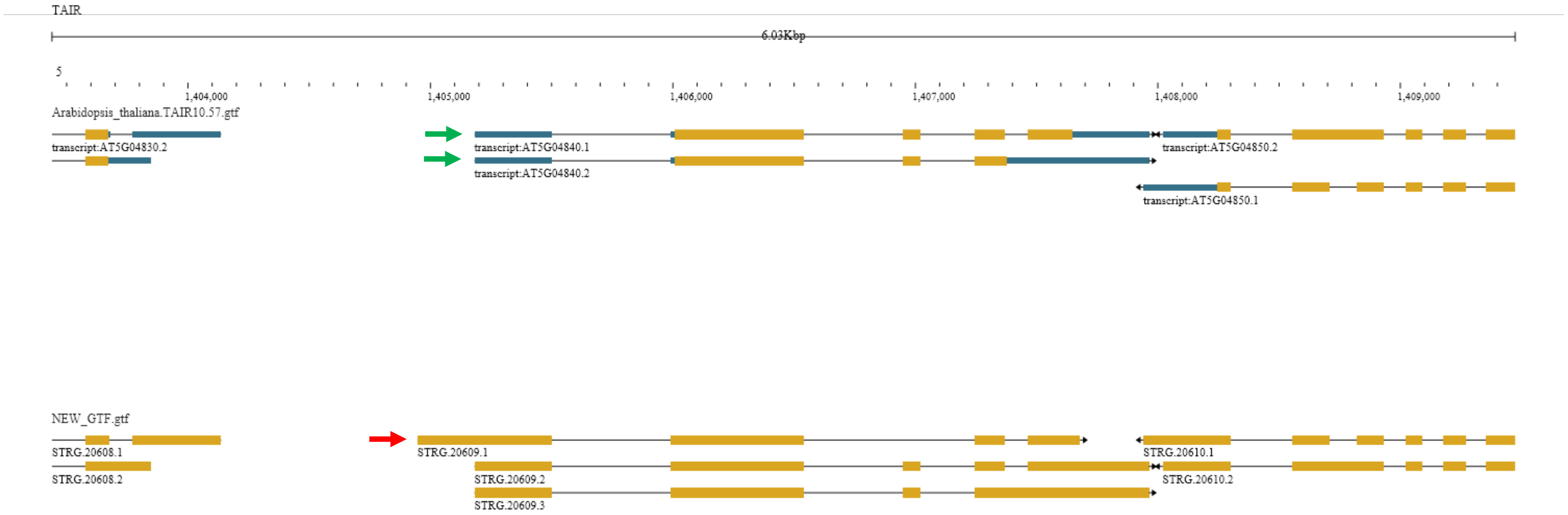

Figure S35. Structure of previously annotated and new DAS isoforms on XLOC_019990 (Gene 28, STRG.20609) generated due to different multifactorial stress combinations in the transcriptome datasets of 10-d-old *A. thaliana* (wild-type Col-0) seedlings. Red arrow refers to the gene isoform concordantly expressed with a given splicing factor, while green arrow(s) refer to other regulated isoforms of this gene. Other isoforms are not consistently regulated under the stress. Further information is available in Tables S1, S3 and S6.

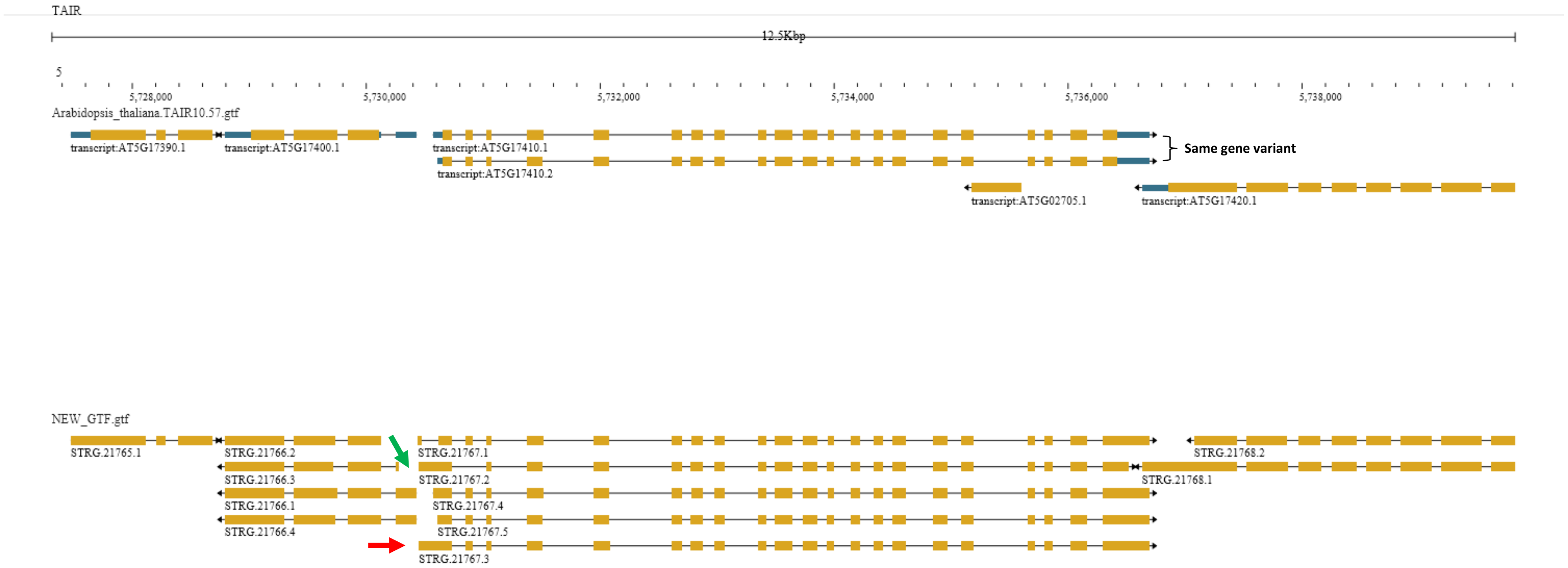

Figure S36. Structure of previously annotated and new DAS isoforms on XLOC_020544 (Gene 29, STRG.21767) generated due to different multifactorial stress combinations in the transcriptome datasets of 10-d-old *A. thaliana* (wild-type Col-0) seedlings. Red arrow refers to the gene isoform concordantly expressed with a given splicing factor, while green arrow(s) refer to other regulated isoforms of this gene. Other isoforms are not consistently regulated under the stress. Further information is available in Tables S1, S3 and S6.

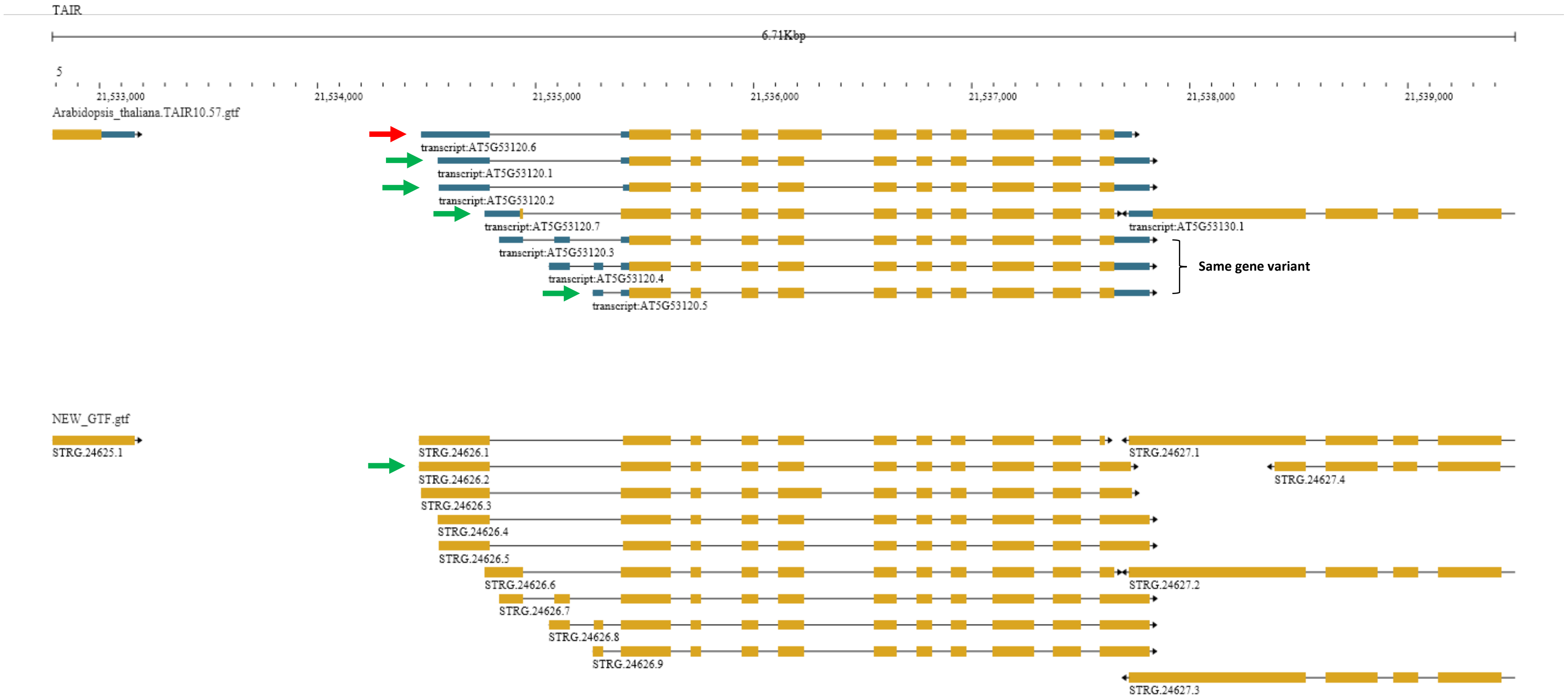

Figure S37. Structure of previously annotated and new DAS isoforms on XLOC_021961 (Gene 30, AT5G53120) generated due to different multifactorial stress combinations in the transcriptome datasets of 10-d-old *A. thaliana* (wild-type Col-0) seedlings. Red arrow refers to the gene isoform concordantly expressed with a given splicing factor, while green arrow(s) refer to other regulated isoforms of this gene. Other isoforms are not consistently regulated under the stress. Further information is available in Tables S1, S3 and S6.

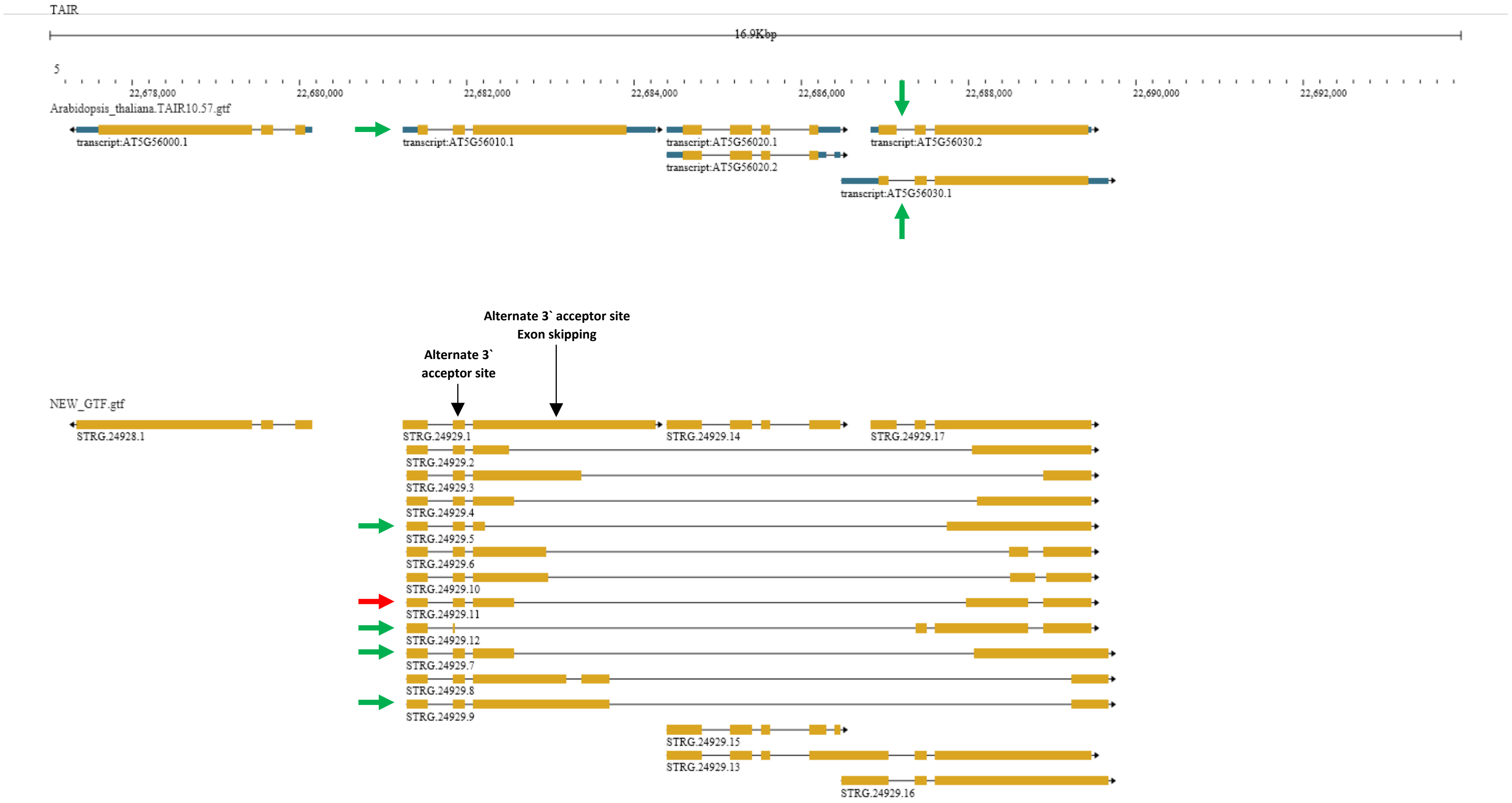

Figure S38. Structure of previously annotated and new DAS isoforms on XLOC_022121 (Gene 31, STRG.24929) generated due to different multifactorial stress combinations in the transcriptome datasets of 10-d-old *A. thaliana* (wild-type Col-0) seedlings. Red arrow refers to the gene isoform concordantly expressed with a given splicing factor, while green arrow(s) refer to other regulated isoforms of this gene. Other isoforms are not consistently regulated under the stress. Further information is available in Tables S1, S3 and S6.

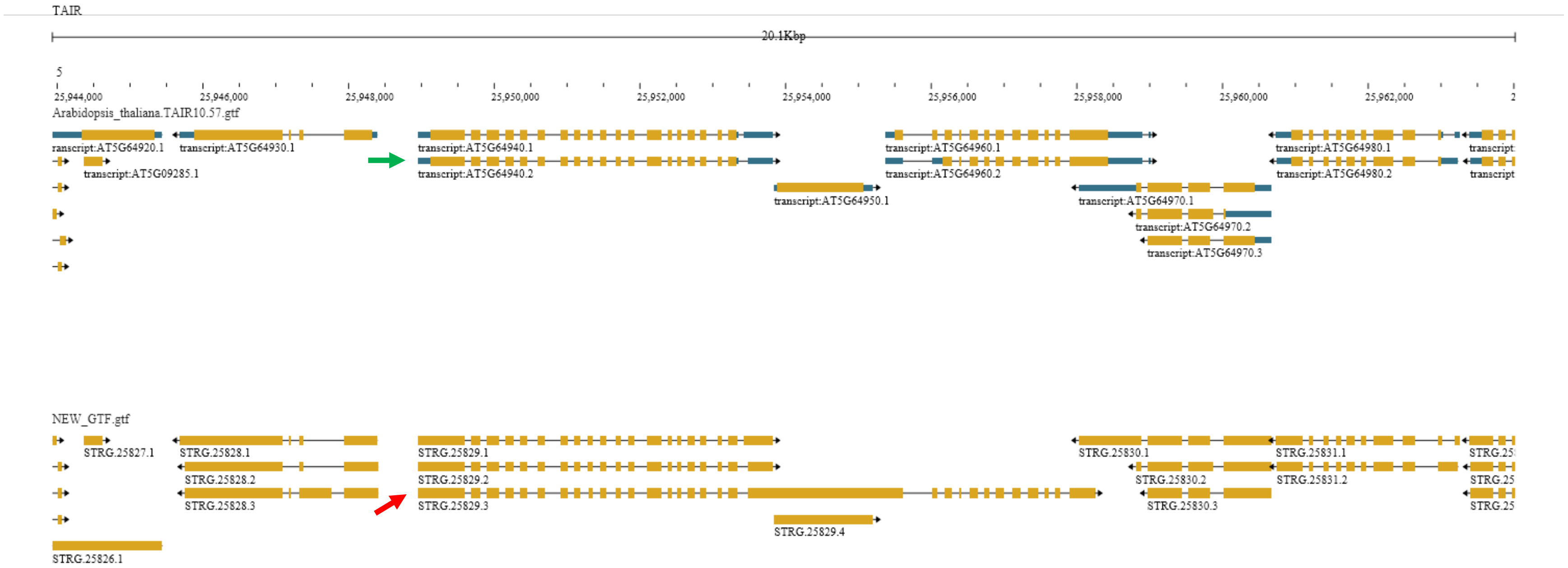

Figure S39. Structure of previously annotated and new DAS isoforms on XLOC_022555 (Gene 32, STRG.25829) generated due to different multifactorial stress combinations in the transcriptome datasets of 10-d-old *A. thaliana* (wild-type Col-0) seedlings. Red arrow refers to the gene isoform concordantly expressed with a given splicing factor, while green arrow(s) refer to other regulated isoforms of this gene. Other isoforms are not consistently regulated under the stress. Further information is available in Tables S1, S3 and S6.

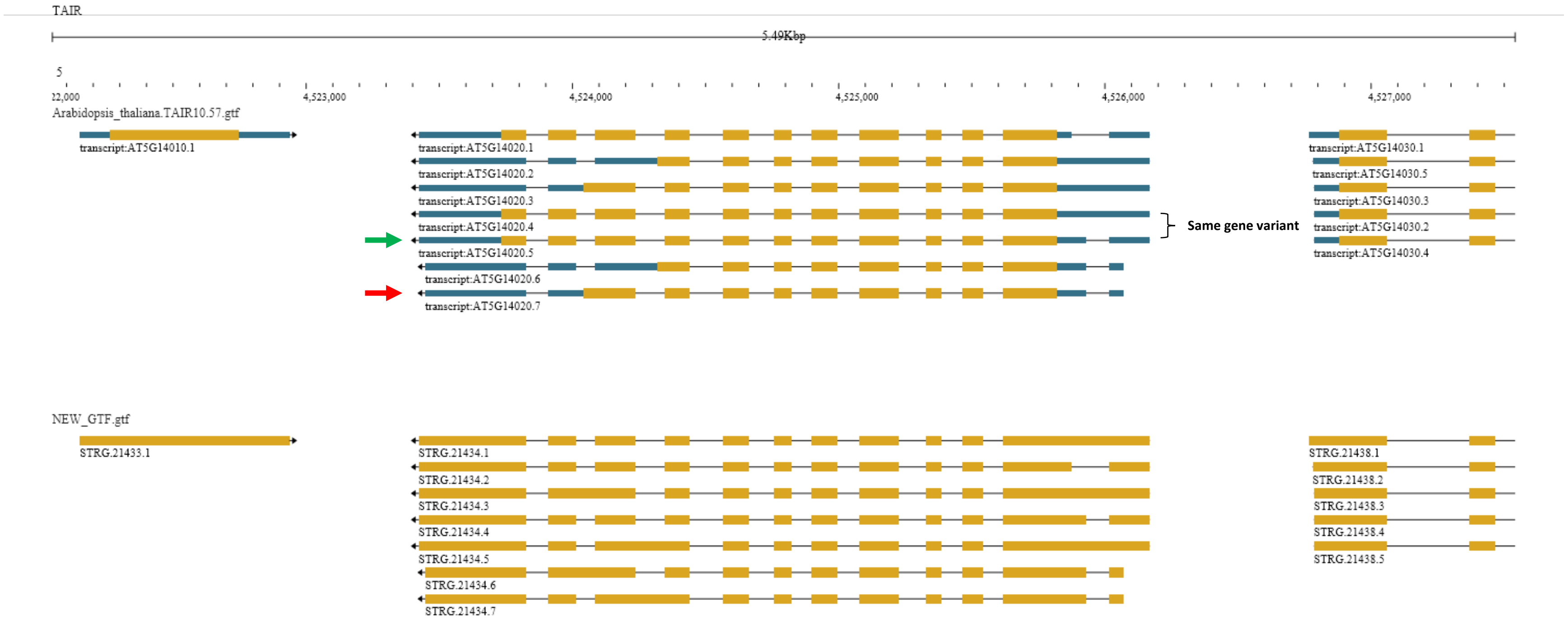

Figure S40. Structure of previously annotated and new DAS isoforms on XLOC_023309 (Gene 33, AT5G14020) generated due to different multifactorial stress combinations in the transcriptome datasets of 10-d-old *A. thaliana* (wild-type Col-0) seedlings. Red arrow refers to the gene isoform concordantly expressed with a given splicing factor, while green arrow(s) refer to other regulated isoforms of this gene. Other isoforms are not consistently regulated under the stress. Further information is available in Tables S1, S3 and S6.

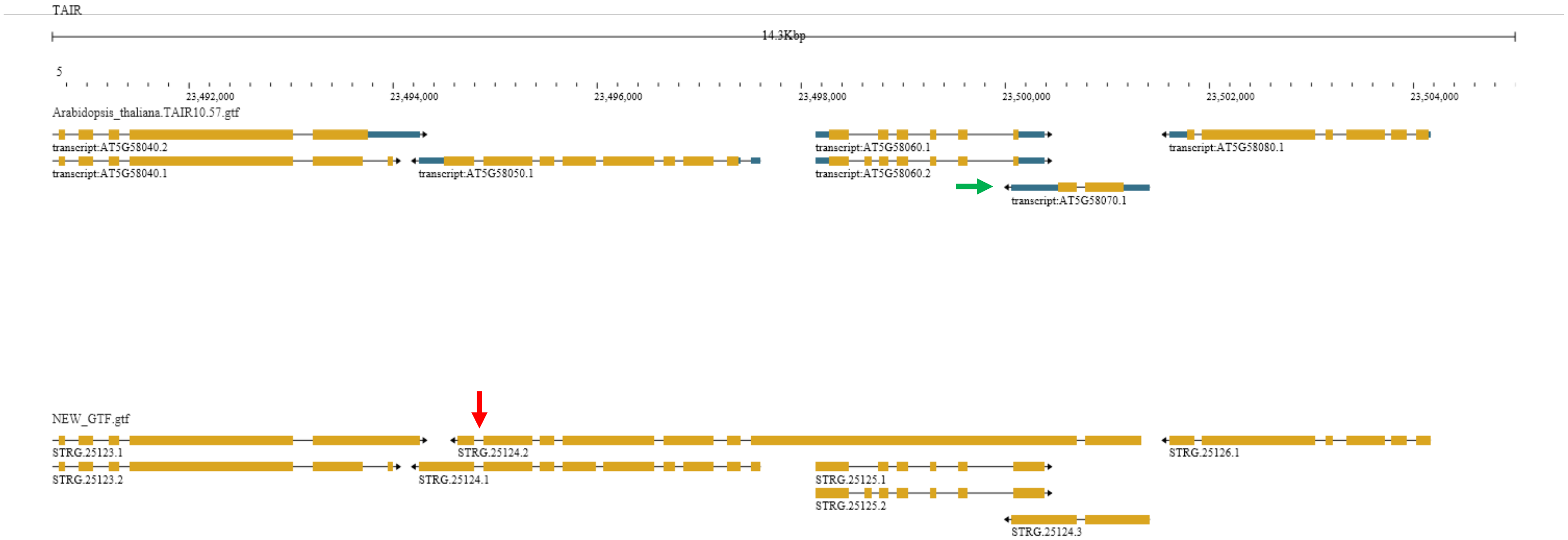

Figure S41. Structure of previously annotated and new DAS isoforms on XLOC_025093 (Gene 34, STRG.25124) generated due to different multifactorial stress combinations in the transcriptome datasets of 10-d-old *A. thaliana* (wild-type Col-0) seedlings. Red arrow refers to the gene isoform concordantly expressed with a given splicing factor, while green arrow(s) refer to other regulated isoforms of this gene. Other isoforms are not consistently regulated under the stress. Further information is available in Tables S1, S3 and S6.

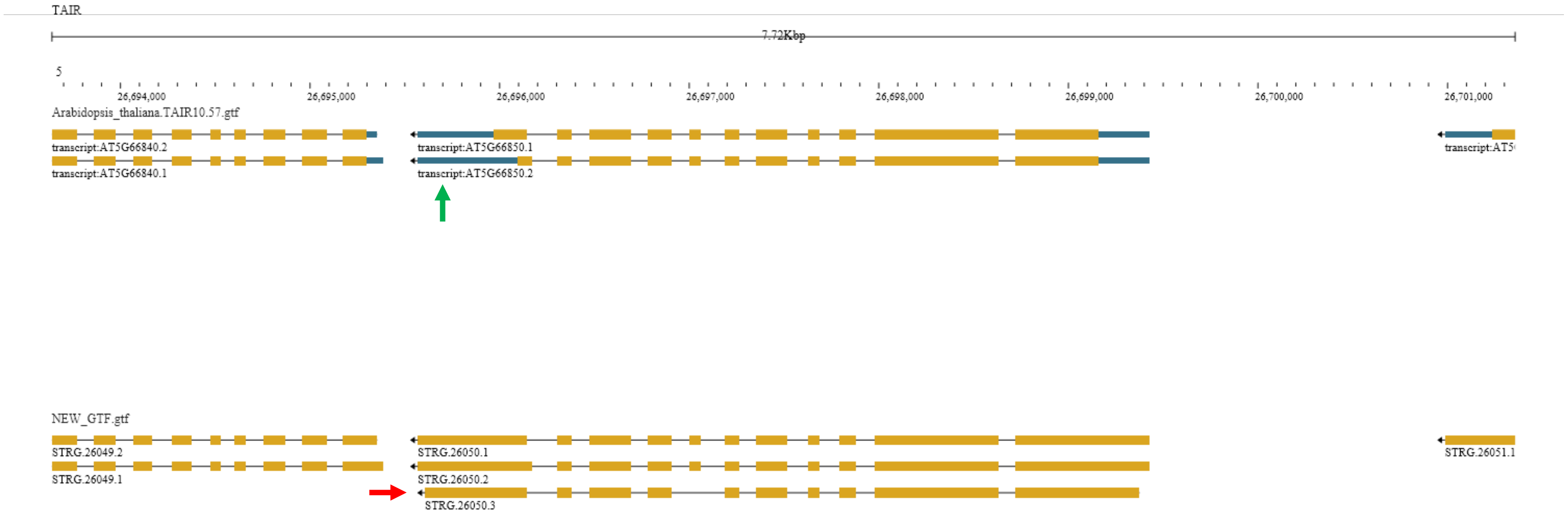

Figure S42. Structure of previously annotated and new DAS isoforms on XLOC_022555 (Gene 35, STRG.26050) generated due to different multifactorial stress combinations in the transcriptome datasets of 10-d-old *A. thaliana* (wild-type Col-0) seedlings. Red arrow refers to the gene isoform concordantly expressed with a given splicing factor, while green arrow(s) refer to other regulated isoforms of this gene. Other isoforms are not consistently regulated under the stress. Further information is available in Tables S1, S3 and S6.

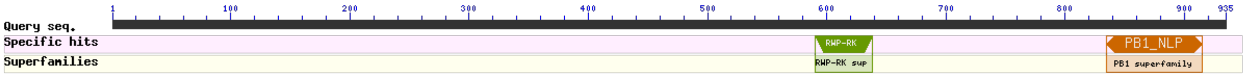

Figure S48. Position of the active conserved motifs RWP-RK (pfam02042) and PB1-NLP (cd06407) of the ABC transporter B family member 11 protein encoded by the *A. thaliana* gene isoform AT2G43500.11 located on locus XLOC_008527. Structure and function of these two motifs were previously describes (English et al., 1995, Schauser et al., 1999, Ge et al., 2018, Wang et al., 2023). Further information on the structure of this gene isoform is available in Figures 5 and S20.

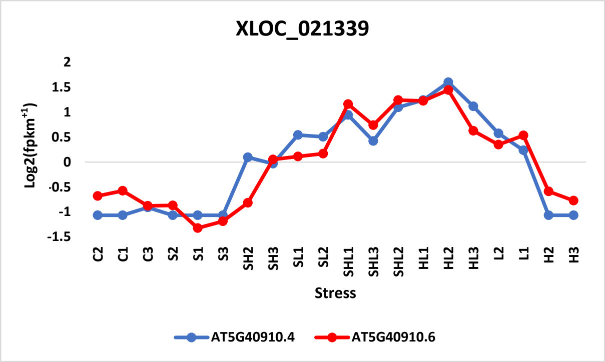

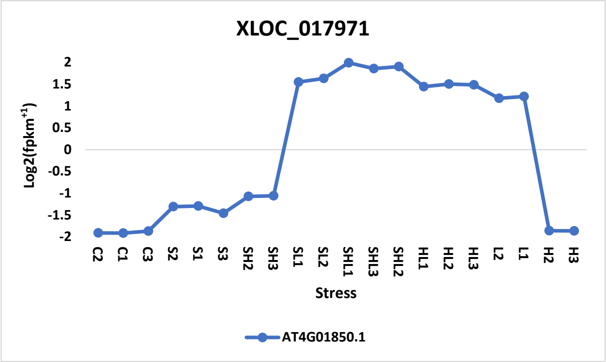

Figure S49. Expression profiling of isoforms on the two loci XLOC_021339 and XLOC_017971 involving the genes encoding 1-aminocyclopropane-1-carboxylate oxidase 10 (ACO10) (AT5G40910) of cluster 967 and S-adenosyl-methionine (SAM) synthetase 2 (SAM synthetase 2) (AT4G01850) of cluster 1414, respectively, generated from transcriptome datasets of 10-d-old *A. thaliana* (wild-type Col-0) seedlings exposed to different multifactorial stress combinations. C = control (0 mM NaCl, 21°C, 50 µmol m^{-2} s^{-1}), S = salt stress (50 mM NaCl, 21°C, 50 µmol m^{-2} s^{-1}), H = heat stress (0 mM NaCl, 33°C, 50 µmol m^{-2} s^{-1}), L = high light stress (0 mM NaCl, 21°C, 700 µmol m^{-2} s^{-1}).

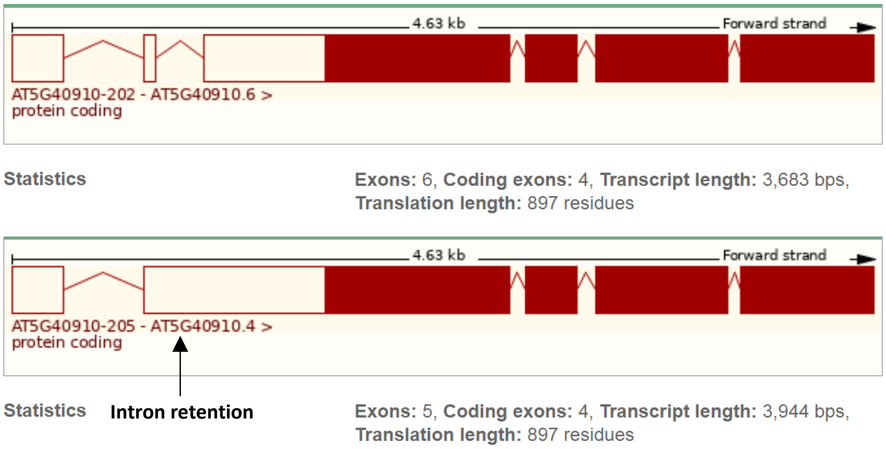

Figure S50. Splicing patterns of the two isoforms of locus XLOC_021339 with the gene (AT5G40910) encoding 1-aminocyclopropane-1-carboxylate oxidase 10 (ACO10). These two isoforms were alternatively spliced with intron retention as the sole splicing type. Splicing patterns of the two splicing isoforms were detected from the following ensemble links:

https://ensembl.gramene.org/A._thaliana/Transcript/Summary?db=core;g=AT5G40910;r=5:16394502-16399129;t=AT5G40910.4

https://ensembl.gramene.org/A._thaliana/Transcript/Summary?db=core;g=AT5G40910;r=5:16394502-16399129;t=AT5G40910.6

References

English, J.M., Vanderbilt, C.A., Xu, S., Marcus, S., Cobb, M.H., 1995. Isolation of mek5 and differential expression of alternatively spliced forms. J Biol Chem. https://doi.org/10.1074/jbc.270.48.28897

Ge, M., Liu, Y., Jiang, L., Wang, Y., Lv, Y., Zhou, L., Liang, S., Bao, H., Zhao, H., 2018. Genome-wide analysis of maize nlp transcription factor family revealed the roles in nitrogen response. Plant Growth Regulation.

Schauser, L., Roussis, A., Stiller, J., Stougaard, J., 1999. A plant regulator controlling development of symbiotic root nodules. Nature. https://doi.org/10.1038/46058

Wang, J., Chitsaz, F., Derbyshire, M.K., Gonzales, N.R., Gwadz, M., Lu, S., Marchler, G.H., Song, J.S., Thanki, N., Yamashita, R.A., Yang, M., Zhang, D., Zheng, C., Lanczycki, C.J., Marchler-Bauer, A., 2023. The conserved domain database in 2023. Nucleic Acids Res. https://doi.org/10.1093/nar/gkac1096

Zandalinas, S.I., Sengupta, S., Fritschi, F.B., Azad, R.K., Nechushtai, R., Mittler, R., 2021. The impact of multifactorial stress combination on plant growth and survival. New Phytol. https://doi.org/10.1111/nph.17232
