# Supplementary material for: Concordant Gene Expression and Alternative Splicing Regulation under Abiotic Stresses in Arabidopsis
Source: Genes (Basel). 2024 May 23;15(6):675. doi: 10.3390/genes15060675 (PMC11202685; doi:10.3390/genes15060675)
Supplement: Supplementary file 1 [file genes-15-00675-s001.zip › Figure S43.pdf]

Figure S43. Multiple sequence alignment at the DNA level for annotated and new isoforms of *A. thaliana* locus XLOC_008527 generated under different multifactorial stress combinations where isoforms AT2G43500.11 and STRG.10463.9 showed expression pattern HL↑, isoform STRG. 10463.14 showed expression pattern all stress combinations↑, while expression of isoforms AT2G43500.9 and AT2G43500.10 was arbitrary. H = heat stress, L = high light stress.

Alignment Name: Untitled4
Length: 3102

	10 / 20 / 30
AT2G43500.9	A T G G A A A A C C C T T T T G C T T C T A G A G A A A A G G G
AT2G43500.10	A T G G A A A A C C C T T T T G C T T C T A G A G A A A A G G G
AT2G43500.11	A T G G A A A A C C C T T T T G C T T C T A G A G A A A A G G G
STRG.10463.14	A T G G A A A A C C C T T T T G C T T C T A G A G A A A A G G G
STRG.10463.9	A T G G A A A A C C C T T T T G C T T C T A G A G A A A A G G G
	A T G G A A A A C C C T T T T G C T T C T A G A G A A A A G G G

	40 / 50 / 60
AT2G43500.9	G T T T G G T A A T T A T T C A G A T T T T C C A A C A G A A C
AT2G43500.10	G T T T G G T A A T T A T T C A G A T T T T C C A A C A G A A C
AT2G43500.11	G T T T G G T A A T T A T T C A G A T T T T C C A A C A G A A C
STRG.10463.14	G T T T G G T A A T T A T T C A G A T T T T C C A A C A G A A C
STRG.10463.9	G T T T G G T A A T T A T T C A G A T T T T C C A A C A G A A C
	G T T T G G T A A T T A T T C A G A T T T T C C A A C A G A A C

	70 / 80 / 90
AT2G43500.9	A A A T G G A T G G T T T A A G C T C A A A C T T T G G T T C T
AT2G43500.10	A A A T G G A T G G T T T A A G C T C A A A C T T T G G T T C T
AT2G43500.11	A A A T G G A T G G T T T A A G C T C A A A C T T T G G T T C T
STRG.10463.14	A A A T G G A T G G T T T A A G C T C A A A C T T T G G T T C T
STRG.10463.9	A A A T G G A T G G T T T A A G C T C A A A C T T T G G T T C T
	A A A T G G A T G G T T T A A G C T C A A A C T T T G G T T C T

	100 / 110 / 120
AT2G43500.9	G G C G T T A G G A A T T T G A T C T C T G A T G A T A T G T T
AT2G43500.10	G G C G T T A G G A A T T T G A T C T C T G A T G A T A T G T T
AT2G43500.11	G G C G T T A G G A A T T T G A T C T C T G A T G A T A T G T T
STRG.10463.14	G G C G T T A G G A A T T T G A T C T C T G A T G A T A T G T T
STRG.10463.9	G G C G T T A G G A A T T T G A T C T C T G A T G A T A T G T T
	G G C G T T A G G A A T T T G A T C T C T G A T G A T A T G T T

	130 / 140 / 150 / 160
AT2G43500.9	C A A C C C T T C C T C C G A G C T T A T G A A T T T C G A C T
AT2G43500.10	C A A C C C T T C C T C C G A G C T T A T G A A T T T C G A C T
AT2G43500.11	C A A C C C T T C C T C C G A G C T T A T G A A T T T C G A C T
STRG.10463.14	C A A C C C T T C C T C C G A G C T T A T G A A T T T C G A C T
STRG.10463.9	C A A C C C T T C C T C C G A G C T T A T G A A T T T C G A C T
	C A A C C C T T C C T C C G A G C T T A T G A A T T T C G A C T

	170 / 180 / 190
AT2G43500.9	C T T T G G C C G C A T G G T G T A A C A G C C C T T C C G C T
AT2G43500.10	C T T T G G C C G C A T G G T G T A A C A G C C C T T C C G C T
AT2G43500.11	C T T T G G C C G C A T G G T G T A A C A G C C C T T C C G C T
STRG.10463.14	C T T T G G C C G C A T G G T G T A A C A G C C C T T C C G C T
STRG.10463.9	C T T T G G C C G C A T G G T G T A A C A G C C C T T C C G C T
	C T T T G G C C G C A T G G T G T A A C A G C C C T T C C G C T

Alignment Name: Untitled4
Length: 3102

Position markers: 200, 210, 220

AT2G43500.9	ACTGATATATTGTTCGCCCAATATGGTCTGTC
AT2G43500.10	ACTGATATATTGTTCGCCCAATATGGTCTGTC
AT2G43500.11	ACTGATATATTGTTCGCCCAATATGGTCTGTC
STRG.10463.14	ACTGATATATTGTTCGCCCAATATGGTCTGTC
STRG.10463.9	ACTGATATATTGTTCGCCCAATATGGTCTGTC
	ACTGATATATTGTTCGCCCAATATGGTCTGTC

Position markers: 230, 240, 250

AT2G43500.9	GAACTCCCAACCTATGCCTTTTGGAGCTTTCA
AT2G43500.10	GAACTCCCAACCTATGCCTTTTGGAGCTTTCA
AT2G43500.11	GAACTCCCAACCTATGCCTTTTGGAGCTTTCA
STRG.10463.14	GAACTCCCAACCTATGCCTTTTGGAGCTTTCA
STRG.10463.9	GAACTCCCAACCTATGCCTTTTGGAGCTTTCA
	GAACTCCCAACCTATGCCTTTTGGAGCTTTCA

Position markers: 260, 270, 280

AT2G43500.9	CTTCATTTCATGTAGCTGACCCGAAGGCTACC
AT2G43500.10	CTTCATTTCATGTAGCTGACCCGAAGGCTACC
AT2G43500.11	CTTCATTTCATGTAGCTGACCCGAAGGCTACC
STRG.10463.14	CTTCATTTCATGTAGCTGACCCGAAGGCTACC
STRG.10463.9	CTTCATTTCATGTAGCTGACCCGAAGGCTACC
	CTTCATTTCATGTAGCTGACCCGAAGGCTACC

Position markers: 290, 300, 310, 320

AT2G43500.9	AGTCTCACTCGTTCGTTCTATGATTTGGAAAG
AT2G43500.10	AGTCTCACTCGTTCGTTCTATGATTTGGAAAG
AT2G43500.11	AGTCTCACTCGTTCGTTCTATGATTTGGAAAG
STRG.10463.14	AGTCTCACTCGTTCGTTCTATGATTTGGAAAG
STRG.10463.9	AGTCTCACTCGTTCGTTCTATGATTTGGAAAG
	AGTCTCACTCGTTCGTTCTATGATTTGGAAAG

Position markers: 330, 340, 350

AT2G43500.9	CTCTTACTATGGTGAAGAAAGATCGTCAGCAC
AT2G43500.10	CTCTTACTATGGTGAAGAAAGATCGTCAGCAC
AT2G43500.11	CTCTTACTATGGTGAAGAAAGATCGTCAGCAC
STRG.10463.14	CTCTTACTATGGTGAAGAAAGATCGTCAGCAC
STRG.10463.9	CTCTTACTATGGTGAAGAAAGATCGTCAGCAC
	CTCTTACTATGGTGAAGAAAGATCGTCAGCAC

Position markers: 360, 370, 380

AT2G43500.9	AGGAAATGAATTCTCAGTTTCATCGCTCTTCA
AT2G43500.10	AGGAAATGAATTCTCAGTTTCATCGCTCTTCA
AT2G43500.11	AGGAAATGAATTCTCAGTTTCATCGCTCTTCA
STRG.10463.14	AGGAAATGAATTCTCAGTTTCATCGCTCTTCA
STRG.10463.9	AGGAAATGAATTCTCAGTTTCATCGCTCTTCA
	AGGAAATGAATTCTCAGTTTCATCGCTCTTCA

Alignment Name: Untitled4
Length: 3102

						390										400										410						
AT2G43500.9	G	A	T	A	G	C	G	A	T	G	A	G	T	T	A	A	G	T	G	G	T	A	A	A	C	G	A	C	G	C	A	A
AT2G43500.10	G	A	T	A	G	C	G	A	T	G	A	G	T	T	A	A	G	T	G	G	T	A	A	A	C	G	A	C	G	C	A	A
AT2G43500.11	G	A	T	A	G	C	G	A	T	G	A	G	T	T	A	A	G	T	G	G	T	A	A	A	C	G	A	C	G	C	A	A
STRG.10463.14	G	A	T	A	G	C	G	A	T	G	A	G	T	T	A	A	G	T	G	G	T	A	A	A	C	G	A	C	G	C	A	A
STRG.10463.9	G	A	T	A	G	C	G	A	T	G	A	G	T	T	A	A	G	T	G	G	T	A	A	A	C	G	A	C	G	C	A	A
	G	A	T	A	G	C	G	A	T	G	A	G	T	T	A	A	G	T	G	G	T	A	A	A	C	G	A	C	G	C	A	A

				420										430										440								
AT2G43500.9	G	G	T	T	G	T	T	A	A	T	C	A	G	A	A	G	A	T	T	G	G	T	T	T	T	C	C	T	A	A	C	G
AT2G43500.10	G	G	T	T	G	T	T	A	A	T	C	A	G	A	A	G	A	T	T	G	G	T	T	T	T	C	C	T	A	A	C	G
AT2G43500.11	G	G	T	T	G	T	T	A	A	T	C	A	G	A	A	G	A	T	T	G	G	T	T	T	T	C	C	T	A	A	C	G
STRG.10463.14	G	G	T	T	G	T	T	A	A	T	C	A	G	A	A	G	A	T	T	G	G	T	T	T	T	C	C	T	A	A	C	G
STRG.10463.9	G	G	T	T	G	T	T	A	A	T	C	A	G	A	A	G	A	T	T	G	G	T	T	T	T	C	C	T	A	A	C	G
	G	G	T	T	G	T	T	A	A	T	C	A	G	A	A	G	A	T	T	G	G	T	T	T	T	C	C	T	A	A	C	G

		450										460										470										480
AT2G43500.9	T	A	C	T	C	A	A	C	T	G	T	A	C	T	A	T	T	C	C	C	A	G	G	T	C	T	T	T	G	A	G	C
AT2G43500.10	T	A	C	T	C	A	A	C	T	G	T	A	C	T	A	T	T	C	C	C	A	G	G	T	C	T	T	T	G	A	G	C
AT2G43500.11	T	A	C	T	C	A	A	C	T	G	T	A	C	T	A	T	T	C	C	C	A	G	G	T	C	T	T	T	G	A	G	C
STRG.10463.14	T	A	C	T	C	A	A	C	T	G	T	A	C	T	A	T	T	C	C	C	A	G	G	T	C	T	T	T	G	A	G	C
STRG.10463.9	T	A	C	T	C	A	A	C	T	G	T	A	C	T	A	T	T	C	C	C	A	G	G	T	C	T	T	T	G	A	G	C
	T	A	C	T	C	A	A	C	T	G	T	A	C	T	A	T	T	C	C	C	A	G	G	T	C	T	T	T	G	A	G	C

										490										500										510		
AT2G43500.9	C	A	C	T	C	A	T	T	A	G	A	T	G	A	G	A	A	G	A	T	G	C	T	T	A	A	G	G	C	A	T	T
AT2G43500.10	C	A	C	T	C	A	T	T	A	G	A	T	G	A	G	A	A	G	A	T	G	C	T	T	A	A	G	G	C	A	T	T
AT2G43500.11	C	A	C	T	C	A	T	T	A	G	A	T	G	A	G	A	A	G	A	T	G	C	T	T	A	A	G	G	C	A	T	T
STRG.10463.14	C	A	C	T	C	A	T	T	A	G	A	T	G	A	G	A	A	G	A	T	G	C	T	T	A	A	G	G	C	A	T	T
STRG.10463.9	C	A	C	T	C	A	T	T	A	G	A	T	G	A	G	A	A	G	A	T	G	C	T	T	A	A	G	G	C	A	T	T
	C	A	C	T	C	A	T	T	A	G	A	T	G	A	G	A	A	G	A	T	G	C	T	T	A	A	G	G	C	A	T	T

								520										530										540				
AT2G43500.9	A	A	G	C	T	T	A	T	T	T	A	T	G	G	A	G	T	C	C	T	C	A	G	G	T	T	C	A	G	G	T	G
AT2G43500.10	A	A	G	C	T	T	A	T	T	T	A	T	G	G	A	G	T	C	C	T	C	A	G	G	T	T	C	A	G	G	T	G
AT2G43500.11	A	A	G	C	T	T	A	T	T	T	A	T	G	G	A	G	T	C	C	T	C	A	G	G	T	T	C	A	G	G	T	G
STRG.10463.14	A	A	G	C	T	T	A	T	T	T	A	T	G	G	A	G	T	C	C	T	C	A	G	G	T	T	C	A	G	G	T	G
STRG.10463.9	A	A	G	C	T	T	A	T	T	T	A	T	G	G	A	G	T	C	C	T	C	A	G	G	T	T	C	A	G	G	T	G
	A	A	G	C	T	T	A	T	T	T	A	T	G	G	A	G	T	C	C	T	C	A	G	G	T	T	C	A	G	G	T	G

						550										560										570						
AT2G43500.9	A	G	G	G	C	A	T	T	T	T	A	G	C	G	C	A	A	G	T	T	T	G	G	A	C	C	C	C	T	A	T	C
AT2G43500.10	A	G	G	G	C	A	T	T	T	T	A	G	C	G	C	A	A	G	T	T	T	G	G	A	C	C	C	C	T	A	T	C
AT2G43500.11	A	G	G	G	C	A	T	T	T	T	A	G	C	G	C	A	A	G	T	T	T	G	G	A	C	C	C	C	T	A	T	C
STRG.10463.14	A	G	G	G	C	A	T	T	T	T	A	G	C	G	C	A	A	G	T	T	T	G	G	A	C	C	C	C	T	A	T	C
STRG.10463.9	A	G	G	G	C	A	T	T	T	T	A	G	C	G	C	A	A	G	T	T	T	G	G	A	C	C	C	C	T	A	T	C
	A	G	G	G	C	A	T	T	T	T	A	G	C	G	C	A	A	G	T	T	T	G	G	A	C	C	C	C	T	A	T	C

Alignment Name: Untitled4
Length: 3102

580 590 600

AT2G43500.9 AAGACAGGAGATCAGTACTTGCTCAGTACTTG
AT2G43500.10 AAGACAGGAGATCAGTACTTGCTCAGTACTTG
AT2G43500.11 AAGACAGGAGATCAGTACTTGCTCAGTACTTG
STRG.10463.14 AAGACAGGAGATCAGTACTTGCTCAGTACTTG
STRG.10463.9 AAGACAGGAGATCAGTACTTGCTCAGTACTTG

AAGACAGGAGATCAGTACTTGCTCAGTACTTG

610 620 630 640

AT2G43500.9 TGATCAGGCCTATTTGCTTGATCCGAGGTTTT
AT2G43500.10 TGATCAGGCCTATTTGCTTGATCCGAGGTTTT
AT2G43500.11 TGATCAGGCCTATTTGCTTGATCCGAGGTTTT
STRG.10463.14 TGATCAGGCCTATTTGCTTGATCCGAGGTTTT
STRG.10463.9 TGATCAGGCCTATTTGCTTGATCCGAGGTTTT

TGATCAGGCCTATTTGCTTGATCCGAGGTTTT

650 660 670

AT2G43500.9 CTCAGTACCGTGAAGTGTCAAGGAGATTTACG
AT2G43500.10 CTCAGTACCGTGAAGTGTCAAGGAGATTTACG
AT2G43500.11 CTCAGTACCGTGAAGTGTCAAGGAGATTTACG
STRG.10463.14 CTCAGTACCGTGAAGTGTCAAGGAGATTTACG
STRG.10463.9 CTCAGTACCGTGAAGTGTCAAGGAGATTTACG

CTCAGTACCGTGAAGTGTCAAGGAGATTTACG

680 690 700

AT2G43500.9 TTTGCTGCTGAAGCAAATCAATGTTCTTTTCC
AT2G43500.10 TTTGCTGCTGAAGCAAATCAATGTTCTTTTCC
AT2G43500.11 TTTGCTGCTGAAGCAAATCAATGTTCTTTTCC
STRG.10463.14 TTTGCTGCTGAAGCAAATCAATGTTCTTTTCC
STRG.10463.9 TTTGCTGCTGAAGCAAATCAATGTTCTTTTCC

TTTGCTGCTGAAGCAAATCAATGTTCTTTTCC

710 720 730

AT2G43500.9 GGGTCTTCCTGGCCGAGTCTTTATCTCGGGAG
AT2G43500.10 GGGTCTTCCTGGCCGAGTCTTTATCTCGGGAG
AT2G43500.11 GGGTCTTCCTGGCCGAGTCTTTATCTCGGGAG
STRG.10463.14 GGGTCTTCCTGGCCGAGTCTTTATCTCGGGAG
STRG.10463.9 GGGTCTTCCTGGCCGAGTCTTTATCTCGGGAG

GGGTCTTCCTGGCCGAGTCTTTATCTCGGGAG

740 750 760

AT2G43500.9 TACCTGAATGGACATCAAACGTTATGTATTAC
AT2G43500.10 TACCTGAATGGACATCAAACGTTATGTATTAC
AT2G43500.11 TACCTGAATGGACATCAAACGTTATGTATTAC
STRG.10463.14 TACCTGAATGGACATCAAACGTTATGTATTAC
STRG.10463.9 TACCTGAATGGACATCAAACGTTATGTATTAC

TACCTGAATGGACATCAAACGTTATGTATTAC

770 780 790 800

AT2G43500.9	AAGACCGATGAGTATTTGCGCATGAAGCATGC
AT2G43500.10	AAGACCGATGAGTATTTGCGCATGAAGCATGC
AT2G43500.11	AAGACCGATGAGTATTTGCGCATGAAGCATGC
STRG.10463.14	AAGACCGATGAGTATTTGCGCATGAAGCATGC
STRG.10463.9	AAGACCGATGAGTATTTGCGCATGAAGCATGC
	AAGACCGATGAGTATTTGCGCATGAAGCATGC

810 820 830

AT2G43500.9	AATTGACAATGAAGTCCGTGGTTCCATTGCAA
AT2G43500.10	AATTGACAATGAAGTCCGTGGTTCCATTGCAA
AT2G43500.11	AATTGACAATGAAGTCCGTGGTTCCATTGCAA
STRG.10463.14	AATTGACAATGAAGTCCGTGGTTCCATTGCAA
STRG.10463.9	AATTGACAATGAAGTCCGTGGTTCCATTGCAA
	AATTGACAATGAAGTCCGTGGTTCCATTGCAA

840 850 860

AT2G43500.9	TACCTATCCTTGAAGCTTCTGGGACATCTTGT
AT2G43500.10	TACCTATCCTTGAAGCTTCTGGGACATCTTGT
AT2G43500.11	TACCTATCCTTGAAGCTTCTGGGACATCTTGT
STRG.10463.14	TACCTATCCTTGAAGCTTCTGGGACATCTTGT
STRG.10463.9	TACCTATCCTTGAAGCTTCTGGGACATCTTGT
	TACCTATCCTTGAAGCTTCTGGGACATCTTGT

870 880 890

AT2G43500.9	TGTGCTGTCATGGAACTCGTTACATCCAAGGA
AT2G43500.10	TGTGCTGTCATGGAACTCGTTACATCCAAGGA
AT2G43500.11	TGTGCTGTCATGGAACTCGTTACATCCAAGGA
STRG.10463.14	TGTGCTGTCATGGAACTCGTTACATCCAAGGA
STRG.10463.9	TGTGCTGTCATGGAACTCGTTACATCCAAGGA
	TGTGCTGTCATGGAACTCGTTACATCCAAGGA

900 910 920

AT2G43500.9	AAAACCTAATTTTGATATGGAGATGGACTCTG
AT2G43500.10	AAAACCTAATTTTGATATGGAGATGGACTCTG
AT2G43500.11	AAAACCTAATTTTGATATGGAGATGGACTCTG
STRG.10463.14	AAAACCTAATTTTGATATGGAGATGGACTCTG
STRG.10463.9	AAAACCTAATTTTGATATGGAGATGGACTCTG
	AAAACCTAATTTTGATATGGAGATGGACTCTG

930 940 950 960

AT2G43500.9	TTTGCCGTGCTCTCCAGG--------------
AT2G43500.10	TTTGCCGTGCTCTCCAGG--------------
AT2G43500.11	TTTGCCGTGCTCTCCAGG--------------
STRG.10463.14	TTTGCCGTGCTCTCCAGC--------------
STRG.10463.9	TTTGCCGTGCTCTCCAGGGTATGAGAATCTTT
	TTTGCCGTGCTCTCCAGgGTATGAGAATCTTT

Alignment Name: Untitled4
Length: 3102

	970 · 980 · 990
AT2G43500.9	--------------------------------
AT2G43500.10	--------------------------------
AT2G43500.11	--------------------------------
STRG.10463.14	--------------------------------
STRG.10463.9	GTTGGCTTAAGACCTGCCTGAGTCCTCTGCCT
	GTTGGCTTAAGACCTGCCTGAGTCCTCTGCCT

	1000 · 1010 · 1020
AT2G43500.9	--------------------------------
AT2G43500.10	--------------------------------
AT2G43500.11	--------------------------------
STRG.10463.14	--------------TTCCAGGGCAGCTCTGGA
STRG.10463.9	TTGTCAATGAAAGCTTCCAGGGCAGCTCTGGA
	TTGTCAATGAAAGCTTCCAGGGCAGCTCTGGA

	1030 · 1040 · 1050
AT2G43500.9	----------------------------CTGT
AT2G43500.10	----------------------------CTGT
AT2G43500.11	----------------------------CTGT
STRG.10463.14	GACAGTTTGTTGTGGTTGCAACCTGAGGCTGT
STRG.10463.9	GACAGTTTGTTGTGGTTGCAACCTGAGGCTGT
	GACAGTTTGTTGTGGTTGCAACCTGAGGCTGT

	1060 · 1070 · 1080
AT2G43500.9	AAACTTACGGACAGCAGCCATTCCTCGTCCTC
AT2G43500.10	AAACTTACGGACAGCAGCCATTCCTCGTCCTC
AT2G43500.11	AAACTTACGGACAGCAGCCATTCCTCGTCCTC
STRG.10463.14	AAACTTACGGACAGCAGCCATTCCTCGTCCTC
STRG.10463.9	AAACTTACGGACAGCAGCCATTCCTCGTCCTC
	AAACTTACGGACAGCAGCCATTCCTCGTCCTC

	1090 · 1100 · 1110 · 1120
AT2G43500.9	AGTATCTTTCAAGTAGTCAAAGAGATGCCTTA
AT2G43500.10	AGTATCTTTCAAGTAGTCAAAGAGATGCCTTA
AT2G43500.11	AGTATCTTTCAAGTAGTCAAAGAGATGCCTTA
STRG.10463.14	AGTATCTTTCAAGTAGTCAAAGAGATGCCTTA
STRG.10463.9	AGTATCTTTCAAGTAGTCAAAGAGATGCCTTA
	AGTATCTTTCAAGTAGTCAAAGAGATGCCTTA

	1130 · 1140 · 1150
AT2G43500.9	GCTGAAATACAAGATGTTCTTCGAACAGTATG
AT2G43500.10	GCTGAAATACAAGATGTTCTTCGAACAGTATG
AT2G43500.11	GCTGAAATACAAGATGTTCTTCGAACAGTATG
STRG.10463.14	GCTGAAATACAAGATGTTCTTCGAACAGTATG
STRG.10463.9	GCTGAAATACAAGATGTTCTTCGAACAGTATG
	GCTGAAATACAAGATGTTCTTCGAACAGTATG

Alignment Name: Untitled4
Length: 3102

```
                    1160                1170                1180
AT2G43500.9     TCATGCACACAAGTTGCCCTTAGCTCTTGCCT
AT2G43500.10    TCATGCACACAAGTTGCCCTTAGCTCTTGCCT
AT2G43500.11    TCATGCACACAAGTTGCCCTTAGCTCTTGCCT
STRG.10463.14   TCATGCACACAAGTTGCCCTTAGCTCTTGCCT
STRG.10463.9    TCATGCACACAAGTTGCCCTTAGCTCTTGCCT

                TCATGCACACAAGTTGCCCTTAGCTCTTGCCT
```

```
                1190                1200                1210
AT2G43500.9     GGATTCCTTGTAGAAAGGATCAATCTATAAGG
AT2G43500.10    GGATTCCTTGTAGAAAGGATCAATCTATAAGG
AT2G43500.11    GGATTCCTTGTAGAAAGGATCAATCTATAAGG
STRG.10463.14   GGATTCCTTGTAGAAAGGATCAATCTATAAGG
STRG.10463.9    GGATTCCTTGTAGAAAGGATCAATCTATAAGG

                GGATTCCTTGTAGAAAGGATCAATCTATAAGG
```

```
                1220                1230                1240
AT2G43500.9     GTTTCTGGACAAAAATCTGGTGAAAATTGTAT
AT2G43500.10    GTTTCTGGACAAAAATCTGGTGAAAATTGTAT
AT2G43500.11    GTTTCTGGACAAAAATCTGGTGAAAATTGTAT
STRG.10463.14   GTTTCTGGACAAAAATCTGGTGAAAATTGTAT
STRG.10463.9    GTTTCTGGACAAAAATCTGGTGAAAATTGTAT

                GTTTCTGGACAAAAATCTGGTGAAAATTGTAT
```

```
                1250                1260                1270                1280
AT2G43500.9     ACTTTGCATAGAGGAGACAGCTTGTTATGTGA
AT2G43500.10    ACTTTGCATAGAGGAGACAGCTTGTTATGTGA
AT2G43500.11    ACTTTGCATAGAGGAGACAGCTTGTTATGTGA
STRG.10463.14   ACTTTGCATAGAGGAGACAGCTTGTTATGTGA
STRG.10463.9    ACTTTGCATAGAGGAGACAGCTTGTTATGTGA

                ACTTTGCATAGAGGAGACAGCTTGTTATGTGA
```

```
                1290                1300                1310
AT2G43500.9     ACGATATGGAGATGGAAGGCTTTGTGCACGCA
AT2G43500.10    ACGATATGGAGATGGAAGGCTTTGTGCACGCA
AT2G43500.11    ACGATATGGAGATGGAAGGCTTTGTGCACGCA
STRG.10463.14   ACGATATGGAGATGGAAGGCTTTGTGCACGCA
STRG.10463.9    ACGATATGGAGATGGAAGGCTTTGTGCACGCA

                ACGATATGGAGATGGAAGGCTTTGTGCACGCA
```

```
                1320                1330                1340
AT2G43500.9     TGTTTGGAGCATTGTCTAAGAGAAAAAGAAGG
AT2G43500.10    TGTTTGGAGCATTGTCTAAGAGAAAAAGAAGG
AT2G43500.11    TGTTTGGAGCATTGTCTAAGAGAAAAAGAAGG
STRG.10463.14   TGTTTGGAGCATTGTCTAAGAGAAAAAGAAGG
STRG.10463.9    TGTTTGGAGCATTGTCTAAGAGAAAAAGAAGG

                TGTTTGGAGCATTGTCTAAGAGAAAAAGAAGG
```

					1350										1360										1370							
AT2G43500.9	A	A	T	T	G	T	T	G	G	T	A	A	A	G	C	T	T	T	C	A	T	A	T	C	C	A	A	C	C	A	G	C
AT2G43500.10	A	A	T	T	G	T	T	G	G	T	A	A	A	G	C	T	T	T	C	A	T	A	T	C	C	A	A	C	C	A	G	C
AT2G43500.11	A	A	T	T	G	T	T	G	G	T	A	A	A	G	C	T	T	T	C	A	T	A	T	C	C	A	A	C	C	A	G	C
STRG.10463.14	A	A	T	T	G	T	T	G	-	-	-	-	-	-	-	-	-	-	-	-	-	-	-	-	-	-	-	-	-	-	-	-
STRG.10463.9	A	A	T	T	G	T	T	G	G	T	A	A	A	G	C	T	T	T	C	A	T	A	T	C	C	A	A	C	C	A	G	C
	A	A	T	T	G	T	T	G	G	T	A	A	A	G	C	T	T	T	C	A	T	A	T	C	C	A	A	C	C	A	G	C

			1380										1390										1400									
AT2G43500.9	C	G	T	T	C	T	T	T	T	C	T	T	C	T	G	A	T	G	T	G	A	A	G	G	C	A	T	A	T	G	A	C
AT2G43500.10	C	G	T	T	C	T	T	T	T	C	T	T	C	T	G	A	T	G	T	G	A	A	G	G	C	A	T	A	T	G	A	C
AT2G43500.11	C	G	T	T	C	T	T	T	T	C	T	T	C	T	G	A	T	G	T	G	A	A	G	G	C	A	T	A	T	G	A	C
STRG.10463.14	-	-	-	-	-	-	-	-	-	-	-	-	-	-	-	-	-	-	-	-	-	-	-	-	-	-	-	-	-	-	-	-
STRG.10463.9	C	G	T	T	C	T	T	T	T	C	T	T	C	T	G	A	T	G	T	G	A	A	G	G	C	A	T	A	T	G	A	C
	C	G	T	T	C	T	T	T	T	C	T	T	C	T	G	A	T	G	T	G	A	A	G	G	C	A	T	A	T	G	A	C

		1410										1420										1430										1440
AT2G43500.9	A	T	C	A	G	T	G	A	A	T	A	C	C	C	T	A	T	T	G	T	T	C	A	G	C	A	T	G	C	T	C	G
AT2G43500.10	A	T	C	A	G	T	G	A	A	T	A	C	C	C	T	A	T	T	G	T	T	C	A	G	C	A	T	G	C	T	C	G
AT2G43500.11	A	T	C	A	G	T	G	A	A	T	A	C	C	C	T	A	T	T	G	T	T	C	A	G	C	A	T	G	C	T	C	G
STRG.10463.14	-	-	-	-	-	-	-	-	-	-	-	-	-	-	-	-	-	-	-	-	-	-	-	-	C	A	T	G	C	T	C	G
STRG.10463.9	A	T	C	A	G	T	G	A	A	T	A	C	C	C	T	A	T	T	G	T	T	C	A	G	C	A	T	G	C	T	C	G
	A	T	C	A	G	T	G	A	A	T	A	C	C	C	T	A	T	T	G	T	T	C	A	G	C	A	T	G	C	T	C	G

									1450										1460										1470			
AT2G43500.9	A	A	A	G	T	A	C	G	G	T	C	T	G	A	A	T	G	C	T	G	C	T	G	T	C	G	C	T	A	T	A	A
AT2G43500.10	A	A	A	G	T	A	C	G	G	T	C	T	G	A	A	T	G	C	T	G	C	T	G	T	C	G	C	T	A	T	A	A
AT2G43500.11	A	A	A	G	T	A	C	G	G	T	C	T	G	A	A	T	G	C	T	G	C	T	G	T	C	G	C	T	A	T	A	A
STRG.10463.14	A	A	A	G	T	A	C	G	G	T	C	T	G	A	A	T	G	C	T	G	C	T	G	T	C	G	C	T	A	T	A	A
STRG.10463.9	A	A	A	G	T	A	C	G	G	T	C	T	G	A	A	T	G	C	T	G	C	T	G	T	C	G	C	T	A	T	A	A
	A	A	A	G	T	A	C	G	G	T	C	T	G	A	A	T	G	C	T	G	C	T	G	T	C	G	C	T	A	T	A	A

								1480										1490										1500				
AT2G43500.9	A	A	C	T	G	A	G	G	A	G	C	A	C	T	T	A	C	A	C	T	G	G	T	G	A	A	G	A	T	G	A	T
AT2G43500.10	A	A	C	T	G	A	G	G	A	G	C	A	C	T	T	A	C	A	C	T	G	G	T	G	A	A	G	A	T	G	A	T
AT2G43500.11	A	A	C	T	G	A	G	G	A	G	C	A	C	T	T	A	C	A	C	T	G	G	T	G	A	A	G	A	T	G	A	T
STRG.10463.14	A	A	C	T	G	A	G	G	A	G	C	A	C	T	T	A	C	A	C	T	G	G	T	G	A	A	G	A	T	G	A	T
STRG.10463.9	A	A	C	T	G	A	G	G	A	G	C	A	C	T	T	A	C	A	C	T	G	G	T	G	A	A	G	A	T	G	A	T
	A	A	C	T	G	A	G	G	A	G	C	A	C	T	T	A	C	A	C	T	G	G	T	G	A	A	G	A	T	G	A	T

					1510										1520										1530							
AT2G43500.9	T	A	C	A	T	A	C	T	T	G	A	A	C	T	G	T	T	C	T	T	G	C	C	T	G	T	A	A	G	T	A	T
AT2G43500.10	T	A	C	A	T	A	C	T	T	G	A	A	C	T	G	T	T	C	T	T	G	C	C	T	G	T	A	A	G	T	A	T
AT2G43500.11	T	A	C	A	T	A	C	T	T	G	A	A	C	T	G	T	T	C	T	T	G	C	C	T	G	T	A	A	G	T	A	T
STRG.10463.14	T	A	C	A	T	A	C	T	T	G	A	A	C	T	G	T	T	C	T	T	G	C	C	T	G	T	A	A	G	T	A	T
STRG.10463.9	T	A	C	A	T	A	C	T	T	G	A	A	C	T	G	T	T	C	T	T	G	C	C	T	G	T	A	A	G	T	A	T
	T	A	C	A	T	A	C	T	T	G	A	A	C	T	G	T	T	C	T	T	G	C	C	T	G	T	A	A	G	T	A	T

Alignment Name: Untitled4
Length: 3102

1540 · 1550 · 1560

AT2G43500.9	G A A A G G A A G C T T G G A A C A A C A A C T T C T A T T A G
AT2G43500.10	G A A A G G A A G C T T G G A A C A A C A A C T T C T A T T A G
AT2G43500.11	G A A A G G A A G C T T G G A A C A A C A A C T T C T A T T A G
STRG.10463.14	G A A A G G A A G C T T G G A A C A A C A A C T T C T A T T A G
STRG.10463.9	G A A A G G A A G C T T G G A A C A A C A A C T T C T A T T A G
	G A A A G G A A G C T T G G A A C A A C A A C T T C T A T T A G

1570 · 1580 · 1590 · 1600

AT2G43500.9	A C A G C C T T T C G G G T A C A A T G C A G A G A A T T T G T
AT2G43500.10	A C A G C C T T T C G G G T A C A A T G C A G A G A A T T T G T
AT2G43500.11	A C A G C C T T T C G G G T A C A A T G C A G A G A A T T T G T
STRG.10463.14	A C A G C C T T T C G G G T A C A A T G C A G A G A A T T T G T
STRG.10463.9	A C A G C C T T T C G G G T A C A A T G C A G A G A A T T T G T
	A C A G C C T T T C G G G T A C A A T G C A G A G A A T T T G T

1610 · 1620 · 1630

AT2G43500.9	C G A A C T T T G A G A A C T G T T T C A G A A G T G G G G T C
AT2G43500.10	C G A A C T T T G A G A A C T G T T T C A G A A G T G G G G T C
AT2G43500.11	C G A A C T T T G A G A A C T G T T T C A G A A G T G G G G T C
STRG.10463.14	C G A A C T T T G A G A A C T G T T T C A G A A G T G G G G T C
STRG.10463.9	C G A A C T T T G A G A A C T G T T T C A G A A G T G G G G T C
	C G A A C T T T G A G A A C T G T T T C A G A A G T G G G G T C

1640 · 1650 · 1660

AT2G43500.9	A A C T A A A A A A G A A G G G A C T A A A C C T G G A T T T C
AT2G43500.10	A A C T A A A A A A G A A G G G A C T A A A C C T G G A T T T C
AT2G43500.11	A A C T A A A A A A G A A G G G A C T A A A C C T G G A T T T C
STRG.10463.14	A A C T A A A A A A G A A G G G A C T A A A C C T G G A T T T C
STRG.10463.9	A A C T A A A A A A G A A G G G A C T A A A C C T G G A T T T C
	A A C T A A A A A A G A A G G G A C T A A A C C T G G A T T T C

1670 · 1680 · 1690

AT2G43500.9	G G A G T A G T G A T A T G T C T A A T T T C C C G C A G A C A
AT2G43500.10	G G A G T A G T G A T A T G T C T A A T T T C C C G C A G A C A
AT2G43500.11	G G A G T A G T G A T A T G T C T A A T T T C C C G C A G A C A
STRG.10463.14	G G A G T A G T G A T A T G T C T A A T T T C C C G C A G A C A
STRG.10463.9	G G A G T A G T G A T A T G T C T A A T T T C C C G C A G A C A
	G G A G T A G T G A T A T G T C T A A T T T C C C G C A G A C A

1700 · 1710 · 1720

AT2G43500.9	A C G T C T T C A G A A A A T T T T C A G A C A A T A T C A T T
AT2G43500.10	A C G T C T T C A G A A A A T T T T C A G A C A A T A T C A T T
AT2G43500.11	A C G T C T T C A G A A A A T T T T C A G A C A A T A T C A T T
STRG.10463.14	A C G T C T T C A G A A A A T T T T C A G A C A A T A T C A T T
STRG.10463.9	A C G T C T T C A G A A A A T T T T C A G A C A A T A T C A T T
	A C G T C T T C A G A A A A T T T T C A G A C A A T A T C A T T

Alignment Name: Untitled4
Length: 3102

	1730 · 1740 · 1750 · 1760
AT2G43500.9	G G A T T C C G A G T T T A A C T C T A C T A G A A G C A T G T
AT2G43500.10	G G A T T C C G A G T T T A A C T C T A C T A G A A G C A T G T
AT2G43500.11	G G A T T C C G A G T T T A A C T C T A C T A G A A G C A T G T
STRG.10463.14	G G A T T C C G A G T T T A A C T C T A C T A G A A G C A T G T
STRG.10463.9	G G A T T C C G A G T T T A A C T C T A C T A G A A G C A T G T
	G G A T T C C G A G T T T A A C T C T A C T A G A A G C A T G T

	1770 · 1780 · 1790
AT2G43500.9	T T T C G G G T A T G T C C T C T G A T A A A G A A A A C A G T
AT2G43500.10	T T T C G G G T A T G T C C T C T G A T A A A G A A A A C A G T
AT2G43500.11	T T T C G G G T A T G T C C T C T G A T A A A G A A A A C A G T
STRG.10463.14	T T T C G G G T A T G T C C T C T G A T A A A G A A A A C A G T
STRG.10463.9	T T T C G G G T A T G T C C T C T G A T A A A G A A A A C A G T
	T T T C G G G T A T G T C C T C T G A T A A A G A A A A C A G T

	1800 · 1810 · 1820
AT2G43500.9	A T C A C A G T A T C T C A A G G C A C T T T G G A G C A G G A
AT2G43500.10	A T C A C A G T A T C T C A A G G C A C T T T G G A G C A G G A
AT2G43500.11	A T C A C A G T A T C T C A A G G C A C T T T G G A G C A G G A
STRG.10463.14	A T C A C A G T A T C T C A A G G C A C T T T G G A G C A G G A
STRG.10463.9	A T C A C A G T A T C T C A A G G C A C T T T G G A G C A G G A
	A T C A C A G T A T C T C A A G G C A C T T T G G A G C A G G A

	1830 · 1840 · 1850
AT2G43500.9	T G T G A G C A A A G C A A G A A C A C C A G A G A A G A A G A
AT2G43500.10	T G T G A G C A A A G C A A G A A C A C C A G A G A A G A A G A
AT2G43500.11	T G T G A G C A A A G C A A G A A C A C C A G A G A A G A A G A
STRG.10463.14	T G T G A G C A A A G C A A G A A C A C C A G A G A A G A A G A
STRG.10463.9	T G T G A G C A A A G C A A G A A C A C C A G A G A A G A A G A
	T G T G A G C A A A G C A A G A A C A C C A G A G A A G A A G A

	1860 · 1870 · 1880
AT2G43500.9	A A A G C A C T A C A G A G A A A A A T G T G A G C T T A A G C
AT2G43500.10	A A A G C A C T A C A G A G A A A A A T G T G A G C T T A A G C
AT2G43500.11	A A A G C A C T A C A G A G A A A A A T G T G A G C T T A A G C
STRG.10463.14	A A A G C A C T A C A G A G A A A A A T G T G A G C T T A A G C
STRG.10463.9	A A A G C A C T A C A G A G A A A A A T G T G A G C T T A A G C
	A A A G C A C T A C A G A G A A A A A T G T G A G C T T A A G C

	1890 · 1900 · 1910 · 1920
AT2G43500.9	G C T C T C C A A C A A C A C T T C T C T G G G A G T C T A A A
AT2G43500.10	G C T C T C C A A C A A C A C T T C T C T G G G A G T C T A A A
AT2G43500.11	G C T C T C C A A C A A C A C T T C T C T G G G A G T C T A A A
STRG.10463.14	G C T C T C C A A C A A C A C T T C T C T G G G A G T C T A A A
STRG.10463.9	G C T C T C C A A C A A C A C T T C T C T G G G A G T C T A A A
	G C T C T C C A A C A A C A C T T C T C T G G G A G T C T A A A

Alignment Name: Untitled4
Length: 3102

	1930 · 1940 · 1950
AT2G43500.9	GGATGCTGCAAAAAGCCTTGGTGTTTGTCCAA
AT2G43500.10	GGATGCTGCAAAAAGCCTTGGTGTTTGTCCAA
AT2G43500.11	GGATGCTGCAAAAAGCCTTGGTGTTTGTCCAA
STRG.10463.14	GGATGCTGCAAAAAGCCTTGGTGTTTGTCCAA
STRG.10463.9	GGATGCTGCAAAAAGCCTTGGTGTTTGTCCAA
	GGATGCTGCAAAAAGCCTTGGTGTTTGTCCAA

	1960 · 1970 · 1980
AT2G43500.9	CTACATTGAAACGGATATGCAGGCAACATGGG
AT2G43500.10	CTACATTGAAACGGATATGCAGGCAACATGGG
AT2G43500.11	CTACATTGAAACGGATATGCAGGCAACATGGG
STRG.10463.14	CTACATTGAAACGGATATGCAGGCAACATGGG
STRG.10463.9	CTACATTGAAACGGATATGCAGGCAACATGGG
	CTACATTGAAACGGATATGCAGGCAACATGGG

	1990 · 2000 · 2010
AT2G43500.9	ATCATGAGGTGGCCATCTCGTAAGATTAACAA
AT2G43500.10	ATCATGAGGTGGCCATCTCGTAAGATTAACAA
AT2G43500.11	ATCATGAGGTGGCCATCTCGTAAGATTAACAA
STRG.10463.14	ATCATGAGGTGGCCATCTCGTAAGATTAACAA
STRG.10463.9	ATCATGAGGTGGCCATCTCGTAAGATTAACAA
	ATCATGAGGTGGCCATCTCGTAAGATTAACAA

	2020 · 2030 · 2040
AT2G43500.9	AGTGAACAGGTCACTAAGGAAAATACAGACGG
AT2G43500.10	AGTGAACAGGTCACTAAGGAAAATACAGACGG
AT2G43500.11	AGTGAACAGGTCACTAAGGAAAATACAGACGG
STRG.10463.14	AGTGAACAGGTCACTAAGGAAAATACAGACGG
STRG.10463.9	AGTGAACAGGTCACTAAGGAAAATACAGACGG
	AGTGAACAGGTCACTAAGGAAAATACAGACGG

	2050 · 2060 · 2070 · 2080
AT2G43500.9	TACTGGACTCGGTCCAAGGTGTAGAAGGAGGA
AT2G43500.10	TACTGGACTCGGTCCAAGGTGTAGAAGGAGGA
AT2G43500.11	TACTGGACTCGGTCCAAGGTGTAGAAGGAGGA
STRG.10463.14	TACTGGACTCGGTCCAAGGTGTAGAAGGAGGA
STRG.10463.9	TACTGGACTCGGTCCAAGGTGTAGAAGGAGGA
	TACTGGACTCGGTCCAAGGTGTAGAAGGAGGA

	2090 · 2100 · 2110
AT2G43500.9	CTAAAGTTCGACTCAGCAACTGGCGAATTCAT
AT2G43500.10	CTAAAGTTCGACTCAGCAACTGGCGAATTCAT
AT2G43500.11	CTAAAGTTCGACTCAGCAACTGGCGAATTCAT
STRG.10463.14	CTAAAGTTCGACTCAGCAACTGGCGAATTCAT
STRG.10463.9	CTAAAGTTCGACTCAGCAACTGGCGAATTCAT
	CTAAAGTTCGACTCAGCAACTGGCGAATTCAT

Alignment Name: Untitled4
Length: 3102

```
                  2120      2130      2140
AT2G43500.9    TGCAGTTAGACCTTTTATTCAAGAAATTGATA
AT2G43500.10   TGCAGTTAGACCTTTTATTCAAGAAATTGATA
AT2G43500.11   TGCAGTTAGACCTTTTATTCAAGAAATTGATA
STRG.10463.14  TGCAGTTAGACCTTTTATTCAAGAAATTGATA
STRG.10463.9   TGCAGTTAGACCTTTTATTCAAGAAATTGATA

               TGCAGTTAGACCTTTTATTCAAGAAATTGATA
```

```
                2150      2160      2170
AT2G43500.9    CCCAAAAGGGTCTGTCGTCTCTTGATAATGAT
AT2G43500.10   CCCAAAAGGGTCTGTCGTCTCTTGATAATGAT
AT2G43500.11   CCCAAAAGGGTCTGTCGTCTCTTGATAATGAT
STRG.10463.14  CCCAAAAGGGTCTGTCGTCTCTTGATAATGAT
STRG.10463.9   CCCAAAAGGGTCTGTCGTCTCTTGATAATGAT

               CCCAAAAGGGTCTGTCGTCTCTTGATAATGAT
```

```
                2180      2190      2200
AT2G43500.9    GCACATGCAAGAAGAAGTCAGGAGGATATGCC
AT2G43500.10   GCACATGCAAGAAGAAGTCAGGAGGATATGCC
AT2G43500.11   GCACATGCAAGAAGAAGTCAGGAGGATATGCC
STRG.10463.14  GCACATGCAAGAAGAAGTCAGGAGGATATGCC
STRG.10463.9   GCACATGCAAGAAGAAGTCAGGAGGATATGCC

               GCACATGCAAGAAGAAGTCAGGAGGATATGCC
```

```
               2210      2220      2230      2240
AT2G43500.9    TGACGATACTTCATTCAAGCTCCAGGAAGCTA
AT2G43500.10   TGACGATACTTCATTCAAGCTCCAGGAAGCTA
AT2G43500.11   TGACGATACTTCATTCAAGCTCCAGGAAGCTA
STRG.10463.14  TGACGATACTTCATTCAAGCTCCAGGAAGCTA
STRG.10463.9   TGACGATACTTCATTCAAGCTCCAGGAAGCTA

               TGACGATACTTCATTCAAGCTCCAGGAAGCTA
```

```
                   2250      2260      2270
AT2G43500.9    AATCTGTCGACAATGCCATTAAGTTAGAGGAG
AT2G43500.10   AATCTGTCGACAATGCCATTAAGTTAGAGGAG
AT2G43500.11   AATCTGTCGACAATGCCATTAAGTTAGAGGAG
STRG.10463.14  AATCTGTCGACAATGCCATTAAGTTAGAGGAG
STRG.10463.9   AATCTGTCGACAATGCCATTAAGTTAGAGGAG

               AATCTGTCGACAATGCCATTAAGTTAGAGGAG
```

```
                  2280      2290      2300
AT2G43500.9    GATACAACCATGAATCAAGCAAGACCAGGATC
AT2G43500.10   GATACAACCATGAATCAAGCAAGACCAGGATC
AT2G43500.11   GATACAACCATGAATCAAGCAAGACCAGGATC
STRG.10463.14  GATACAACCATGAATCAAGCAAGACCAGGATC
STRG.10463.9   GATACAACCATGAATCAAGCAAGACCAGGATC

               GATACAACCATGAATCAAGCAAGACCAGGATC
```

	2310 · 2320 · 2330
AT2G43500.9	ATTCATGGAGGTTAATGCTAGTGGTCAGCCAT
AT2G43500.10	ATTCATGGAGGTTAATGCTAGTGGTCAGCCAT
AT2G43500.11	ATTCATGGAGGTTAATGCTAGTGGTCAGCCAT
STRG.10463.14	ATTCATGGAGGTTAATGCTAGTGGTCAGCCAT
STRG.10463.9	ATTCATGGAGGTTAATGCTAGTGGTCAGCCAT
	ATTCATGGAGGTTAATGCTAGTGGTCAGCCAT

	2340 · 2350 · 2360
AT2G43500.9	GGGCTTGGATGGCCAAAGAGTCTGGCTTGAAT
AT2G43500.10	GGGCTTGGATGGCCAAAGAGTCTGGCTTGAAT
AT2G43500.11	GGGCTTGGATGGCCAAAGAGTCTGGCTTGAAT
STRG.10463.14	GGGCTTGGATGGCCAAAGAGTCTGGCTTGAAT
STRG.10463.9	GGGCTTGGATGGCCAAAGAGTCTGGCTTGAAT
	GGGCTTGGATGGCCAAAGAGTCTGGCTTGAAT

	2370 · 2380 · 2390 · 2400
AT2G43500.9	GGCAGTGAAGGAATAAAGAGCGTTTGCAACTT
AT2G43500.10	GGCAGTGAAGGAATAAAGAGCGTTTGCAACTT
AT2G43500.11	GGCAGTGAAGGAATAAAGAGCGTTTGCAACTT
STRG.10463.14	GGCAGTGAAGGAATAAAGAGCGTTTGCAACTT
STRG.10463.9	GGCAGTGAAGGAATAAAGAGCGTTTGCAACTT
	GGCAGTGAAGGAATAAAGAGCGTTTGCAACTT

	2410 · 2420 · 2430
AT2G43500.9	AAGCTCTGTGGAAATTTCAGATGGAATGGATC
AT2G43500.10	AAGCTCTGTGGAAATTTCAGATGGAATGGATC
AT2G43500.11	AAGCTCTGTGGAAATTTCAGATGGAATGGATC
STRG.10463.14	AAGCTCTGTGGAAATTTCAGATGGAATGGATC
STRG.10463.9	AAGCTCTGTGGAAATTTCAGATGGAATGGATC
	AAGCTCTGTGGAAATTTCAGATGGAATGGATC

	2440 · 2450 · 2460
AT2G43500.9	CAACAATCCGATGCAGTGGCAGTATTGTTGAA
AT2G43500.10	CAACAATCCGATGCAGTGGCAGTATTGTTGAA
AT2G43500.11	CAACAATCCGATGCAGTGGCAGTATTGTTGAA
STRG.10463.14	CAACAATCCGATGCAGTGGCAGTATTGTTGAA
STRG.10463.9	CAACAATCCGATGCAGTGGCAGTATTGTTGAA
	CAACAATCCGATGCAGTGGCAGTATTGTTGAA

	2470 · 2480 · 2490
AT2G43500.9	CCTAACCAATCCATGTCATGCAGCATATCAGA
AT2G43500.10	CCTAACCAATCCATGTCATGCAGCATATCAGA
AT2G43500.11	CCTAACCAATCCATGTCATGCAGCATATCAGA
STRG.10463.14	CCTAACCAATCCATGTCATGCAGCATATCAGA
STRG.10463.9	CCTAACCAATCCATGTCATGCAGCATATCAGA
	CCTAACCAATCCATGTCATGCAGCATATCAGA

	2500 · 2510 · 2520
AT2G43500.9	T T C A T C A A A T G G C T C A G G C G C A G T T C T G C G T G
AT2G43500.10	T T C A T C A A A T G G C T C A G G C G C A G T T C T G C G T G
AT2G43500.11	T T C A T C A A A T G G C T C A G G C G C A G T T C T G C G T G
STRG.10463.14	T T C A T C A A A T G G C T C A G G C G C A G T T C T G C G T G
STRG.10463.9	T T C A T C A A A T G G C T C A G G C G C A G T T C T G C G T G
	T T C A T C A A A T G G C T C A G G C G C A G T T C T G C G T G

	2530 · 2540 · 2550 · 2560
AT2G43500.9	G A A G C T C A T C T A C T T C C A T G G A A G A T T G G A A C
AT2G43500.10	G A A G C T C A T C T A C T T C C A T G G A A G A T T G G A A C
AT2G43500.11	G A A G C T C A T C T A C T T C C A T G G A A G A T T G G A A C
STRG.10463.14	G A A G C T C A T C T A C T T C C A T G G A A G A T T G G A A C
STRG.10463.9	G A A G C T C A T C T A C T T C C A T G G A A G A T T G G A A C
	G A A G C T C A T C T A C T T C C A T G G A A G A T T G G A A C

	2570 · 2580 · 2590
AT2G43500.9	C A A A T G A G A A C C C A C A A C A G T A A T A G C A G C G A
AT2G43500.10	C A A A T G A G A A C C C A C A A C A G T A A T A G C A G C G A
AT2G43500.11	C A A A T G A G A A C C C A C A A C A G T A A T A G C A G C G A
STRG.10463.14	C A A A T G A G A A C C C A C A A C A G T A A T A G C A G C G A
STRG.10463.9	C A A A T G A G A A C C C A C A A C A G T A A T A G C A G C G A
	C A A A T G A G A A C C C A C A A C A G T A A T A G C A G C G A

	2600 · 2610 · 2620
AT2G43500.9	G A G T G G A T C A A C A A C G C T G A T C G T A A A G G C C A
AT2G43500.10	G A G T G G A T C A A C A A C G C T G A T C G T A A A G G C C A
AT2G43500.11	G A G T G G A T C A A C A A C G C T G A T C G T A A A G G C C A
STRG.10463.14	G A G T G G A T C A A C A A C G C T G A T C G T A A A G G C C A
STRG.10463.9	G A G T G G A T C A A C A A C G C T G A T C G T A A A G G C C A
	G A G T G G A T C A A C A A C G C T G A T C G T A A A G G C C A

	2630 · 2640 · 2650
AT2G43500.9	G T T A T A G A G A A G A C A C T G T A C G T T T C A A G T T C
AT2G43500.10	G T T A T A G A G A A G A C A C T G T A C G T T T C A A G T T C
AT2G43500.11	G T T A T A G A G A A G A C A C T G T A C G T T T C A A G T T C
STRG.10463.14	G T T A T A G A G A A G A C A C T G T A C G T T T C A A G T T C
STRG.10463.9	G T T A T A G A G A A G A C A C T G T A C G T T T C A A G T T C
	G T T A T A G A G A A G A C A C T G T A C G T T T C A A G T T C

	2660 · 2670 · 2680
AT2G43500.9	G A G C C A T C A G T T G G G T G T C C T C A G C T C T A C A A
AT2G43500.10	G A G C C A T C A G T T G G G T G T C C T C A G C T C T A C A A
AT2G43500.11	G A G C C A T C A G T T G G G T G T C C T C A G C T C T A C A A
STRG.10463.14	G A G C C A T C A G T T G G G T G T C C T C A G C T C T A C A A
STRG.10463.9	G A G C C A T C A G T T G G G T G T C C T C A G C T C T A C A A
	G A G C C A T C A G T T G G G T G T C C T C A G C T C T A C A A

Alignment Name: Untitled4
Length: 3102

		2690									2700										2710										2720
AT2G43500.9	A	G	A	A	G	T	T	G	G	A	A	A	A	C	G	T	T	T	T	A	A	A	C	T	G	C	A	G	G	A	C	G
AT2G43500.10	A	G	A	A	G	T	T	G	G	A	A	A	A	C	G	T	T	T	T	A	A	A	C	T	G	C	A	G	G	A	C	G
AT2G43500.11	A	G	A	A	G	T	T	G	G	A	A	A	A	C	G	T	T	T	T	A	A	A	C	T	G	C	A	G	G	A	C	G
STRG.10463.14	A	G	A	A	G	T	T	G	G	A	A	A	A	C	G	T	T	T	T	A	A	A	C	T	G	C	A	G	G	A	C	G
STRG.10463.9	A	G	A	A	G	T	T	G	G	A	A	A	A	C	G	T	T	T	T	A	A	A	C	T	G	C	A	G	G	A	C	G
	A	G	A	A	G	T	T	G	G	A	A	A	A	C	G	T	T	T	T	A	A	A	C	T	G	C	A	G	G	A	C	G

										2730										2740										2750		
AT2G43500.9	G	G	T	C	G	T	T	T	C	A	G	C	T	G	A	A	G	T	A	C	T	T	G	G	A	T	G	A	T	G	A	A
AT2G43500.10	G	G	T	C	G	T	T	T	C	A	G	C	T	G	A	A	G	T	A	C	T	T	G	G	A	T	G	A	T	G	A	A
AT2G43500.11	G	G	T	C	G	T	T	T	C	A	G	C	T	G	A	A	G	T	A	C	T	T	G	G	A	T	G	A	T	G	A	A
STRG.10463.14	G	G	T	C	G	T	T	T	C	A	G	C	T	G	A	A	G	T	A	C	T	T	G	G	A	T	G	A	T	G	A	A
STRG.10463.9	G	G	T	C	G	T	T	T	C	A	G	C	T	G	A	A	G	T	A	C	T	T	G	G	A	T	G	A	T	G	A	A
	G	G	T	C	G	T	T	T	C	A	G	C	T	G	A	A	G	T	A	C	T	T	G	G	A	T	G	A	T	G	A	A

								2760										2770										2780				
AT2G43500.9	G	A	A	G	A	A	T	G	G	G	T	G	A	T	G	C	T	G	G	T	T	A	C	A	G	A	T	T	C	T	G	A
AT2G43500.10	G	A	A	G	A	A	T	G	G	G	T	G	A	T	G	C	T	G	G	T	T	A	C	A	G	A	T	T	C	T	G	A
AT2G43500.11	G	A	A	G	A	A	T	G	G	G	T	G	A	T	G	C	T	G	G	T	T	A	C	A	G	A	T	T	C	T	G	A
STRG.10463.14	G	A	A	G	A	A	T	G	G	G	T	G	A	T	G	C	T	G	G	T	T	A	C	A	G	A	T	T	C	T	G	A
STRG.10463.9	G	A	A	G	A	A	T	G	G	G	T	G	A	T	G	C	T	G	G	T	T	A	C	A	G	A	T	T	C	T	G	A
	G	A	A	G	A	A	T	G	G	G	T	G	A	T	G	C	T	G	G	T	T	A	C	A	G	A	T	T	C	T	G	A

						2790										2800										2810						
AT2G43500.9	T	C	T	C	C	A	A	G	A	A	T	G	T	T	T	G	G	A	G	A	T	A	T	T	A	C	A	T	G	G	T	A
AT2G43500.10	T	C	T	C	C	A	A	G	A	A	T	G	T	T	T	G	G	A	G	A	T	A	T	T	A	C	A	T	G	G	T	A
AT2G43500.11	T	C	T	C	C	A	A	G	A	A	T	G	T	T	T	G	G	A	G	A	T	A	T	T	A	C	A	T	G	G	T	A
STRG.10463.14	T	C	T	C	C	A	A	G	A	A	T	G	T	T	T	G	G	A	G	A	T	A	T	T	A	C	A	T	G	G	T	A
STRG.10463.9	T	C	T	C	C	A	A	G	A	A	T	G	T	T	T	G	G	A	G	A	T	A	T	T	A	C	A	T	G	G	T	A
	T	C	T	C	C	A	A	G	A	A	T	G	T	T	T	G	G	A	G	A	T	A	T	T	A	C	A	T	G	G	T	A

				2820										2830										2840								
AT2G43500.9	T	G	G	G	A	A	A	A	C	A	C	T	C	G	G	T	G	A	A	G	T	T	T	C	T	C	G	T	T	C	G	T
AT2G43500.10	T	G	G	G	A	A	A	A	C	A	C	T	C	G	G	T	G	A	A	G	T	T	T	C	T	C	G	T	T	C	G	T
AT2G43500.11	T	G	G	G	A	A	A	A	C	A	C	T	C	G	G	T	G	A	A	G	T	T	T	C	T	C	G	T	T	C	G	T
STRG.10463.14	T	G	G	G	A	A	A	A	C	A	C	T	C	G	G	T	G	A	A	G	T	T	T	C	T	C	G	T	T	C	G	T
STRG.10463.9	T	G	G	G	A	A	A	A	C	A	C	T	C	G	G	T	G	A	A	G	T	T	T	C	T	C	G	T	T	C	G	T
	T	G	G	G	A	A	A	A	C	A	C	T	C	G	G	T	G	A	A	G	T	T	T	C	T	C	G	T	T	C	G	T

		2850									2860										2870										2880
AT2G43500.9	G	A	T	T	T	G	T	C	T	G	C	C	C	C	T	C	T	A	G	G	T	A	G	T	T	C	T	G	G	T	G	G
AT2G43500.10	G	A	T	T	T	G	T	C	T	G	C	C	C	C	T	C	T	A	G	G	T	A	G	T	T	C	T	G	G	T	G	G
AT2G43500.11	G	A	T	T	T	G	T	C	T	G	C	C	C	C	T	C	T	A	G	G	T	A	G	T	T	C	T	G	G	T	G	G
STRG.10463.14	G	A	T	T	T	G	T	C	T	G	C	C	C	C	T	C	T	A	G	G	T	A	G	T	T	C	T	G	G	T	G	G
STRG.10463.9	G	A	T	T	T	G	T	C	T	G	C	C	C	C	T	C	T	A	G	G	T	A	G	T	T	C	T	G	G	T	G	G
	G	A	T	T	T	G	T	C	T	G	C	C	C	C	T	C	T	A	G	G	T	A	G	T	T	C	T	G	G	T	G	G

Alignment Name: Untitled4
Length: 3102

Position markers: 2890, 2900, 2910

Sequence	Alignment
AT2G43500.9	C A G T A A T G G T T A T C T T G G A A C A G G C T T A T G A -
AT2G43500.10	C A G T A A T G G T T A T C T T G G A A C A G G C T T A T G A -
AT2G43500.11	C A G T A A T G G T T A T C T T G G A A C A G G C T T A T G A -
STRG.10463.14	C A G T A A T G G T T A T C T T G G A A C A G G C T T A T G A C
STRG.10463.9	C A G T A A T G G T T A T C T T G G A A C A G G C T T A T G A C
	C A G T A A T G G T T A T C T T G G A A C A G G C T T A T G A C

Position markers: 2920, 2930, 2940

Sequence	Alignment
AT2G43500.9	- -
AT2G43500.10	- -
AT2G43500.11	- -
STRG.10463.14	G T C G T A A G A C A T A G A C A C A C A C A G T T A T G T A T
STRG.10463.9	G T C G T A A G A C A T A G A C A C A C A C A G T T A T G T A T
	G T C G T A A G A C A T A G A C A C A C A C A G T T A T G T A T

Position markers: 2950, 2960, 2970

Sequence	Alignment
AT2G43500.9	- -
AT2G43500.10	- -
AT2G43500.11	- -
STRG.10463.14	T C C C A G T G A A A G A A T G T T G T T T A T T T C T C T A G
STRG.10463.9	T C C C A G T G A A A G A A T G T T G T T T A T T T C T C T A G
	T C C C A G T G A A A G A A T G T T G T T T A T T T C T C T A G

Position markers: 2980, 2990, 3000

Sequence	Alignment
AT2G43500.9	- -
AT2G43500.10	- -
AT2G43500.11	- -
STRG.10463.14	A T A T T A G T A T G C T T A T A A A T A G G C A T G A A G G A
STRG.10463.9	A T A T T A G T A T G C T T A T A A A T A G G C A T G A A G G A
	A T A T T A G T A T G C T T A T A A A T A G G C A T G A A G G A

Position markers: 3010, 3020, 3030, 3040

Sequence	Alignment
AT2G43500.9	- -
AT2G43500.10	- -
AT2G43500.11	- -
STRG.10463.14	G A A A G A C A A T T T T G G T A T A G T G G A G T T C A G C A
STRG.10463.9	G A A A G A C A A T T T T G G T A T A G T G G A G T T C A G C A
	G A A A G A C A A T T T T G G T A T A G T G G A G T T C A G C A

Position markers: 3050, 3060, 3070

Sequence	Alignment
AT2G43500.9	- -
AT2G43500.10	- -
AT2G43500.11	- -
STRG.10463.14	G A A A A T G T A T A T G T T T T T T C G T T T T A T A T G A A
STRG.10463.9	G A A A A T G T A T A T G T T T T T T C G T T T T A T A T G A A
	G A A A A T G T A T A T G T T T T T T C G T T T T A T A T G A A

Alignment Name: Untitled4
Length: 3102

								3080										3090										3100		
AT2G43500.9	-	-	-	-	-	-	-	-	-	-	-	-	-	-	-	-	-	-	-	-	-	-	-	-	-	-	-	-	-	-
AT2G43500.10	-	-	-	-	-	-	-	-	-	-	-	-	-	-	-	-	-	-	-	-	-	-	-	-	-	-	-	-	-	-
AT2G43500.11	-	-	-	-	-	-	-	-	-	-	-	-	-	-	-	-	-	-	-	-	-	-	-	-	-	-	-	-	-	-
STRG.10463.14	T	C	A	G	A	G	A	A	T	A	A	A	A	G	T	T	G	G	A	T	G	T	T	A	T	A	T	C	T	A
STRG.10463.9	T	C	A	G	A	G	A	A	T	A	A	A	A	G	T	T	G	G	A	T	G	T	T	A	T	A	T	C	T	A
	T	C	A	G	A	G	A	A	T	A	A	A	A	G	T	T	G	G	A	T	G	T	T	A	T	A	T	C	T	A
