# Supplementary material for: Concordant Gene Expression and Alternative Splicing Regulation under Abiotic Stresses in Arabidopsis
Source: Genes (Basel). 2024 May 23;15(6):675. doi: 10.3390/genes15060675 (PMC11202685; doi:10.3390/genes15060675)
Supplement: Supplementary file 1 [file genes-15-00675-s001.zip › Figure S44.pdf]

Figure S44. Multiple sequence alignment at the amino acid level for annotated and new isoforms of *A. thaliana* locus XLOC_008527 generated under different multifactorial stress combinations where isoforms AT2G43500.11 and STRG.10463.9 showed expression pattern HL↑, isoform STRG. 10463.14 showed expression pattern all stress combinations↑, while expression of isoforms AT2G43500.9 and AT2G43500.10 was arbitrary. H = heat stress, L = high light stress. The figure emphasizes Exon 1 alignment as referred to in Figure S20.

Alignment Name: Untitled5
Length: 1034

Start1

```
                              1                  10                  20                  30
Translation of AT2G4350(     M E N P F A S R E K G F G N Y S D F P T E Q M D G L S S N F G S
Translation of AT2G4350(     M E N P F A S R E K G F G N Y S D F P T E Q M D G L S S N F G S
Translation of AT2G4350(     M E N P F A S R E K G F G N Y S D F P T E Q M D G L S S N F G S
Translation of STRG.104      M E N P F A S R E K G F G N Y S D F P T E Q M D G L S S N F G S
Translation of STRG.104      M E N P F A S R E K G F G N Y S D F P T E Q M D G L S S N F G S

                             M E N P F A S R E K G F G N Y S D F P T E Q M D G L S S N F G S
```

```
                                           40                  50                  60
Translation of AT2G4350(     G V R N L I S D D M F N P S S E L M N F D S L A A W C N S P S A
Translation of AT2G4350(     G V R N L I S D D M F N P S S E L M N F D S L A A W C N S P S A
Translation of AT2G4350(     G V R N L I S D D M F N P S S E L M N F D S L A A W C N S P S A
Translation of STRG.104      G V R N L I S D D M F N P S S E L M N F D S L A A W C N S P S A
Translation of STRG.104      G V R N L I S D D M F N P S S E L M N F D S L A A W C N S P S A

                             G V R N L I S D D M F N P S S E L M N F D S L A A W C N S P S A
```

```
                                       70                  80                  90
Translation of AT2G4350(     T D I L F A Q Y G L S N S Q P M P F G A F T S F H V A D P K A T
Translation of AT2G4350(     T D I L F A Q Y G L S N S Q P M P F G A F T S F H V A D P K A T
Translation of AT2G4350(     T D I L F A Q Y G L S N S Q P M P F G A F T S F H V A D P K A T
Translation of STRG.104      T D I L F A Q Y G L S N S Q P M P F G A F T S F H V A D P K A T
Translation of STRG.104      T D I L F A Q Y G L S N S Q P M P F G A F T S F H V A D P K A T

                             T D I L F A Q Y G L S N S Q P M P F G A F T S F H V A D P K A T
```

```
                                   100                 110                 120
Translation of AT2G4350(     S L T R S F Y D L E S S Y Y G E E R S S A Q E M N S Q F H R S S
Translation of AT2G4350(     S L T R S F Y D L E S S Y Y G E E R S S A Q E M N S Q F H R S S
Translation of AT2G4350(     S L T R S F Y D L E S S Y Y G E E R S S A Q E M N S Q F H R S S
Translation of STRG.104      S L T R S F Y D L E S S Y Y G E E R S S A Q E M N S Q F H R S S
Translation of STRG.104      S L T R S F Y D L E S S Y Y G E E R S S A Q E M N S Q F H R S S

                             S L T R S F Y D L E S S Y Y G E E R S S A Q E M N S Q F H R S S
```

```
                               130                 140                 150                 160
Translation of AT2G4350(     D S D E L S G K R R K V V N Q K I G F P N V L N C T I P R S L S
Translation of AT2G4350(     D S D E L S G K R R K V V N Q K I G F P N V L N C T I P R S L S
Translation of AT2G4350(     D S D E L S G K R R K V V N Q K I G F P N V L N C T I P R S L S
Translation of STRG.104      D S D E L S G K R R K V V N Q K I G F P N V L N C T I P R S L S
Translation of STRG.104      D S D E L S G K R R K V V N Q K I G F P N V L N C T I P R S L S

                             D S D E L S G K R R K V V N Q K I G F P N V L N C T I P R S L S
```

```
                                             170                 180                 190
Translation of AT2G4350(     H S L D E K M L K A L S L F M E S S G S G E G I L A Q V W T P I
Translation of AT2G4350(     H S L D E K M L K A L S L F M E S S G S G E G I L A Q V W T P I
Translation of AT2G4350(     H S L D E K M L K A L S L F M E S S G S G E G I L A Q V W T P I
Translation of STRG.104      H S L D E K M L K A L S L F M E S S G S G E G I L A Q V W T P I
Translation of STRG.104      H S L D E K M L K A L S L F M E S S G S G E G I L A Q V W T P I

                             H S L D E K M L K A L S L F M E S S G S G E G I L A Q V W T P I
```

Alignment Name: Untitled5
Length: 1034

								200										210										220				
Translation of AT2G435	K	T	G	D	Q	Y	L	L	S	T	C	D	Q	A	Y	L	L	D	P	R	F	S	Q	Y	R	E	V	S	R	R	F	T
Translation of AT2G435	K	T	G	D	Q	Y	L	L	S	T	C	D	Q	A	Y	L	L	D	P	R	F	S	Q	Y	R	E	V	S	R	R	F	T
Translation of AT2G435	K	T	G	D	Q	Y	L	L	S	T	C	D	Q	A	Y	L	L	D	P	R	F	S	Q	Y	R	E	V	S	R	R	F	T
Translation of STRG.104	K	T	G	D	Q	Y	L	L	S	T	C	D	Q	A	Y	L	L	D	P	R	F	S	Q	Y	R	E	V	S	R	R	F	T
Translation of STRG.104	K	T	G	D	Q	Y	L	L	S	T	C	D	Q	A	Y	L	L	D	P	R	F	S	Q	Y	R	E	V	S	R	R	F	T
	K	T	G	D	Q	Y	L	L	S	T	C	D	Q	A	Y	L	L	D	P	R	F	S	Q	Y	R	E	V	S	R	R	F	T

						230										240										250						
Translation of AT2G435	F	A	A	E	A	N	Q	C	S	F	P	G	L	P	G	R	V	F	I	S	G	V	P	E	W	T	S	N	V	M	Y	Y
Translation of AT2G435	F	A	A	E	A	N	Q	C	S	F	P	G	L	P	G	R	V	F	I	S	G	V	P	E	W	T	S	N	V	M	Y	Y
Translation of AT2G435	F	A	A	E	A	N	Q	C	S	F	P	G	L	P	G	R	V	F	I	S	G	V	P	E	W	T	S	N	V	M	Y	Y
Translation of STRG.104	F	A	A	E	A	N	Q	C	S	F	P	G	L	P	G	R	V	F	I	S	G	V	P	E	W	T	S	N	V	M	Y	Y
Translation of STRG.104	F	A	A	E	A	N	Q	C	S	F	P	G	L	P	G	R	V	F	I	S	G	V	P	E	W	T	S	N	V	M	Y	Y
	F	A	A	E	A	N	Q	C	S	F	P	G	L	P	G	R	V	F	I	S	G	V	P	E	W	T	S	N	V	M	Y	Y

				260										270										280								
Translation of AT2G435	K	T	D	E	Y	L	R	M	K	H	A	I	D	N	E	V	R	G	S	I	A	I	P	I	L	E	A	S	G	T	S	C
Translation of AT2G435	K	T	D	E	Y	L	R	M	K	H	A	I	D	N	E	V	R	G	S	I	A	I	P	I	L	E	A	S	G	T	S	C
Translation of AT2G435	K	T	D	E	Y	L	R	M	K	H	A	I	D	N	E	V	R	G	S	I	A	I	P	I	L	E	A	S	G	T	S	C
Translation of STRG.104	K	T	D	E	Y	L	R	M	K	H	A	I	D	N	E	V	R	G	S	I	A	I	P	I	L	E	A	S	G	T	S	C
Translation of STRG.104	K	T	D	E	Y	L	R	M	K	H	A	I	D	N	E	V	R	G	S	I	A	I	P	I	L	E	A	S	G	T	S	C
	K	T	D	E	Y	L	R	M	K	H	A	I	D	N	E	V	R	G	S	I	A	I	P	I	L	E	A	S	G	T	S	C

End1/Start4
315/316

		290									300										310										320
Translation of AT2G435	C	A	V	M	E	L	V	T	S	K	E	K	P	N	F	D	M	E	M	D	S	V	C	R	A	L	Q	A	V	N	L	R
Translation of AT2G435	C	A	V	M	E	L	V	T	S	K	E	K	P	N	F	D	M	E	M	D	S	V	C	R	A	L	Q	A	V	N	L	R
Translation of AT2G435	C	A	V	M	E	L	V	T	S	K	E	K	P	N	F	D	M	E	M	D	S	V	C	R	A	L	Q	A	V	N	L	R
Translation of STRG.104	C	A	V	M	E	L	V	T	S	K	E	K	P	N	F	D	M	E	M	D	S	V	C	R	A	L	Q	L	P	G	Q	L
Translation of STRG.104	C	A	V	M	E	L	V	T	S	K	E	K	P	N	F	D	M	E	M	D	S	V	C	R	A	L	Q	G	M	R	I	F
	C	A	V	M	E	L	V	T	S	K	E	K	P	N	F	D	M	E	M	D	S	V	C	R	A	L	Q	a	v	n	l	r

										330										340										350		
Translation of AT2G435	T	A	A	I	P	R	P	Q	Y	L	S	S	S	Q	R	D	A	L	A	E	I	Q	D	V	L	R	T	V	C	H	A	H
Translation of AT2G435	T	A	A	I	P	R	P	Q	Y	L	S	S	S	Q	R	D	A	L	A	E	I	Q	D	V	L	R	T	V	C	H	A	H
Translation of AT2G435	T	A	A	I	P	R	P	Q	Y	L	S	S	S	Q	R	D	A	L	A	E	I	Q	D	V	L	R	T	V	C	H	A	H
Translation of STRG.104	W	R	Q	F	V	V	V	A	T	*	G	C	K	L	T	D	S	S	H	S	S	S	S	V	S	F	K	*	S	K	R	C
Translation of STRG.104	V	G	L	R	P	A	*	V	L	C	L	C	Q	*	K	L	P	G	Q	L	W	R	Q	F	V	V	V	A	T	*	G	C
	t	a	a	i	p	r	p	q	y	l	s	s	s	q	r	d	a	l	a	e	i	q	d	v	l	r	t	v	c	h	a	h

								360										370										380				
Translation of AT2G435	K	L	P	L	A	L	A	W	I	P	C	R	K	D	Q	S	I	R	V	S	G	Q	K	S	G	E	N	C	I	L	C	I
Translation of AT2G435	K	L	P	L	A	L	A	W	I	P	C	R	K	D	Q	S	I	R	V	S	G	Q	K	S	G	E	N	C	I	L	C	I
Translation of AT2G435	K	L	P	L	A	L	A	W	I	P	C	R	K	D	Q	S	I	R	V	S	G	Q	K	S	G	E	N	C	I	L	C	I
Translation of STRG.104	L	S	*	N	T	R	C	S	S	N	S	M	S	C	T	Q	V	A	L	S	S	C	L	D	S	L	*	K	G	S	I	Y
Translation of STRG.104	K	L	T	D	S	S	H	S	S	S	S	V	S	F	K	*	S	K	R	C	L	S	*	N	T	R	C	S	S	N	S	M
	k	l	p	l	a	l	a	w	i	p	c	r	k	d	q	s	i	r	v	s	g	q	k	s	g	e	n	c	i	l	c	i

```
                                 390                 400                 410
Translation of AT2G435   E E T A C Y V N D M E M E G F V H A C L E H C L R E K E G I V G
Translation of AT2G435   E E T A C Y V N D M E M E G F V H A C L E H C L R E K E G I V G
Translation of AT2G435   E E T A C Y V N D M E M E G F V H A C L E H C L R E K E G I V G
Translation of STRG.104  K G F W T K I W * K L Y T L H R G D S L L C E R Y G D G R L C A
Translation of STRG.104  S C T Q V A L S S C L D S L * K G S I Y K G F W T K I W * K L Y

                         e e t a c y v n d m e m e g f v h a c l e h c l r e k e g i v g
```

```
                             420                 430                 440
Translation of AT2G435   K A F I S N Q P F F S S D V K A Y D I S E Y P I V Q H A R K Y G
Translation of AT2G435   K A F I S N Q P F F S S D V K A Y D I S E Y P I V Q H A R K Y G
Translation of AT2G435   K A F I S N Q P F F S S D V K A Y D I S E Y P I V Q H A R K Y G
Translation of STRG.104  R M F G A L S K R K R R N C C M L E S T V * M L L S L * N * G A
Translation of STRG.104  T L H R G D S L L C E R Y G D G R L C A R M F G A L S K R K R R

                         k a f i s n q p f f s s d v k a y d i s e y p i v q h a r K y g
```

```
                           450                 460                 470                 480
Translation of AT2G435   L N A A V A I K L R S T Y T G E D D Y I L E L F L P V S M K G S
Translation of AT2G435   L N A A V A I K L R S T Y T G E D D Y I L E L F L P V S M K G S
Translation of AT2G435   L N A A V A I K L R S T Y T G E D D Y I L E L F L P V S M K G S
Translation of STRG.104  L T L V K M I T Y L N C S C L * V * K E A W N N N F Y * T A F R
Translation of STRG.104  N C W * S F H I Q P A V L F F * C E G I * H Q * I P Y C S A C S

                         l n a a v a i k l r s t y t g E d d y i l e l f l p v s m k g s
```

```
                                       490                 500                 510
Translation of AT2G435   L E Q Q L L L D S L S G T M Q R I C R T L R T V S E V G S T K K
Translation of AT2G435   L E Q Q L L L D S L S G T M Q R I C R T L R T V S E V G S T K K
Translation of AT2G435   L E Q Q L L L D S L S G T M Q R I C R T L R T V S E V G S T K K
Translation of STRG.104  V Q C R E F V E L * E L F Q K W G Q L K K K G L N L D F G V V I
Translation of STRG.104  K V R S E C C C R Y K T E E H L H W * R * L H T * T V L A C K Y

                         l e q q l l l d s l s g t m q r i c r t l r t v s e v g s t k k
```

```
                                     520                 530                 540
Translation of AT2G435   E G T K P G F R S S D M S N F P Q T T S S E N F Q T I S L D S E
Translation of AT2G435   E G T K P G F R S S D M S N F P Q T T S S E N F Q T I S L D S E
Translation of AT2G435   E G T K P G F R S S D M S N F P Q T T S S E N F Q T I S L D S E
Translation of STRG.104  C L I S R R Q R L Q K I F R Q Y H W I P S L T L L E A C F R V C
Translation of STRG.104  E R K L G T T T S I R Q P F G Y N A E N L S N F E N C F R S G V

                         e g t k p g f r s s d m s n f p q t t s s e n f q t i s l d s e
```

```
                                 550                 560                 570
Translation of AT2G435   F N S T R S M F S G M S S D K E N S I T V S Q G T L E Q D V S K
Translation of AT2G435   F N S T R S M F S G M S S D K E N S I T V S Q G T L E Q D V S K
Translation of AT2G435   F N S T R S M F S G M S S D K E N S I T V S Q G T L E Q D V S K
Translation of STRG.104  P L I K K T V S Q Y L K A L W S R M * A K Q E H Q R R R K A L Q
Translation of STRG.104  N * K R R D * T W I S E * * Y V * F P A D N V F R K F S D N I I

                         f n s t r s m f s g m s s d k e n s i t v s q g t l e q d v s k
```

Alignment Name: Untitled5
Length: 1034

				580										590										600								
Translation of AT2G435(	A	R	T	P	E	K	K	K	S	T	T	E	K	N	V	S	L	S	A	L	Q	Q	H	F	S	G	S	L	K	D	A	A
Translation of AT2G435(	A	R	T	P	E	K	K	K	S	T	T	E	K	N	V	S	L	S	A	L	Q	Q	H	F	S	G	S	L	K	D	A	A
Translation of AT2G435(	A	R	T	P	E	K	K	K	S	T	T	E	K	N	V	S	L	S	A	L	Q	Q	H	F	S	G	S	L	K	D	A	A
Translation of STRG.104	R	K	M	*	A	*	A	L	S	N	N	T	S	L	G	V	*	R	M	L	Q	K	A	L	V	F	V	Q	L	H	*	N
Translation of STRG.104	G	F	R	V	*	L	Y	*	K	H	V	F	G	Y	V	L	*	*	R	K	Q	Y	H	S	I	S	R	H	F	G	A	G
	a	r	t	p	e	k	k	k	s	t	t	e	k	n	v	s	L	s	a	l	Q	q	h	f	s	g	s	l	k	d	A	a

		610										620										630										640
Translation of AT2G435(	K	S	L	G	V	C	P	T	T	L	K	R	I	C	R	Q	H	G	I	M	R	W	P	S	R	K	I	N	K	V	N	R
Translation of AT2G435(	K	S	L	G	V	C	P	T	T	L	K	R	I	C	R	Q	H	G	I	M	R	W	P	S	R	K	I	N	K	V	N	R
Translation of AT2G435(	K	S	L	G	V	C	P	T	T	L	K	R	I	C	R	Q	H	G	I	M	R	W	P	S	R	K	I	N	K	V	N	R
Translation of STRG.104	G	Y	A	G	N	M	G	S	*	G	G	H	L	V	R	L	T	K	*	T	G	H	*	G	K	Y	R	R	Y	W	T	R
Translation of STRG.104	C	E	Q	S	K	N	T	R	E	E	E	K	H	Y	R	E	K	C	E	L	K	R	S	P	T	T	L	L	W	E	S	K
	k	s	l	g	v	c	p	t	t	l	k	r	i	c	R	q	h	g	i	m	r	w	p	s	r	k	i	n	k	v	n	r

										650										660										670		
Translation of AT2G435(	S	L	R	K	I	Q	T	V	L	D	S	V	Q	G	V	E	G	G	L	K	F	D	S	A	T	G	E	F	I	A	V	R
Translation of AT2G435(	S	L	R	K	I	Q	T	V	L	D	S	V	Q	G	V	E	G	G	L	K	F	D	S	A	T	G	E	F	I	A	V	R
Translation of AT2G435(	S	L	R	K	I	Q	T	V	L	D	S	V	Q	G	V	E	G	G	L	K	F	D	S	A	T	G	E	F	I	A	V	R
Translation of STRG.104	S	K	V	*	K	E	D	*	S	S	T	Q	Q	L	A	N	S	L	Q	L	D	L	L	F	K	K	L	I	P	K	R	V
Translation of STRG.104	G	C	C	K	K	P	W	C	L	S	N	Y	I	E	T	D	M	Q	A	T	W	D	H	E	V	A	I	S	*	D	*	Q
	s	l	r	K	i	q	t	v	l	d	s	v	q	g	v	e	g	g	l	k	f	d	s	a	t	g	e	f	i	a	v	r

								680										690										700				
Translation of AT2G435(	P	F	I	Q	E	I	D	T	Q	K	G	L	S	S	L	D	N	D	A	H	A	R	R	S	Q	E	D	M	P	D	D	T
Translation of AT2G435(	P	F	I	Q	E	I	D	T	Q	K	G	L	S	S	L	D	N	D	A	H	A	R	R	S	Q	E	D	M	P	D	D	T
Translation of AT2G435(	P	F	I	Q	E	I	D	T	Q	K	G	L	S	S	L	D	N	D	A	H	A	R	R	S	Q	E	D	M	P	D	D	T
Translation of STRG.104	C	R	L	L	I	M	M	H	M	Q	E	E	V	R	R	I	C	L	T	I	L	H	S	S	S	R	K	L	N	L	S	T
Translation of STRG.104	S	E	Q	V	T	K	E	N	T	D	G	T	G	L	G	P	R	C	R	R	R	T	K	V	R	L	S	N	W	R	I	H
	p	f	i	q	e	i	d	t	q	k	g	l	s	s	l	d	n	d	a	h	a	r	r	s	q	e	d	m	p	d	d	t

						710										720										730						
Translation of AT2G435(	S	F	K	L	Q	E	A	K	S	V	D	N	A	I	K	L	E	E	D	T	T	M	N	Q	A	R	P	G	S	F	M	E
Translation of AT2G435(	S	F	K	L	Q	E	A	K	S	V	D	N	A	I	K	L	E	E	D	T	T	M	N	Q	A	R	P	G	S	F	M	E
Translation of AT2G435(	S	F	K	L	Q	E	A	K	S	V	D	N	A	I	K	L	E	E	D	T	T	M	N	Q	A	R	P	G	S	F	M	E
Translation of STRG.104	M	P	L	S	*	R	R	I	Q	P	*	I	K	Q	D	Q	D	H	S	W	R	L	M	L	V	V	S	H	G	L	G	W
Translation of STRG.104	C	S	*	T	F	Y	S	R	N	*	Y	P	K	G	S	V	V	S	*	*	*	C	T	C	K	K	K	S	G	G	Y	A
	s	f	k	l	q	e	a	k	s	v	d	n	a	i	k	l	e	e	d	t	t	m	n	q	a	r	p	g	s	f	m	e

				740										750										760								
Translation of AT2G435(	V	N	A	S	G	Q	P	W	A	W	M	A	K	E	S	G	L	N	G	S	E	G	I	K	S	V	C	N	L	S	S	V
Translation of AT2G435(	V	N	A	S	G	Q	P	W	A	W	M	A	K	E	S	G	L	N	G	S	E	G	I	K	S	V	C	N	L	S	S	V
Translation of AT2G435(	V	N	A	S	G	Q	P	W	A	W	M	A	K	E	S	G	L	N	G	S	E	G	I	K	S	V	C	N	L	S	S	V
Translation of STRG.104	P	K	S	L	A	*	M	A	V	K	E	*	R	A	F	A	T	*	A	L	W	K	F	Q	M	E	W	I	Q	Q	S	D
Translation of STRG.104	*	R	Y	F	I	Q	A	P	G	S	*	I	C	R	Q	C	H	*	V	R	G	G	Y	N	H	E	S	S	K	T	R	I
	v	n	a	s	g	Q	p	w	a	w	m	a	k	e	s	g	l	N	g	s	e	g	i	k	s	v	c	n	l	s	s	v

Alignment Name: Untitled5
Length: 1034

		770									780										790										800
Translation of AT2G435(	E	I	S	D	G	M	D	P	T	I	R	C	S	G	S	I	V	E	P	N	Q	S	M	S	C	S	I	S	D	S	S	N
Translation of AT2G435(	E	I	S	D	G	M	D	P	T	I	R	C	S	G	S	I	V	E	P	N	Q	S	M	S	C	S	I	S	D	S	S	N
Translation of AT2G435(	E	I	S	D	G	M	D	P	T	I	R	C	S	G	S	I	V	E	P	N	Q	S	M	S	C	S	I	S	D	S	S	N
Translation of STRG.104	A	V	A	V	L	L	N	L	T	N	P	C	H	A	A	Y	Q	I	H	Q	M	A	Q	A	Q	F	C	V	E	A	H	L
Translation of STRG.104	I	H	G	G	*	C	*	W	S	A	M	G	L	D	G	Q	R	V	W	L	E	W	Q	*	R	N	K	E	R	L	Q	L
	e	i	s	d	g	m	d	p	t	i	r	c	s	g	s	i	v	e	p	n	q	s	m	s	c	s	i	s	d	s	s	n

									810										820										830		
Translation of AT2G435(	G	S	G	A	V	L	R	G	S	S	S	T	S	M	E	D	W	N	Q	M	R	T	H	N	S	N	S	S	E	S	G	S
Translation of AT2G435(	G	S	G	A	V	L	R	G	S	S	S	T	S	M	E	D	W	N	Q	M	R	T	H	N	S	N	S	S	E	S	G	S
Translation of AT2G435(	G	S	G	A	V	L	R	G	S	S	S	T	S	M	E	D	W	N	Q	M	R	T	H	N	S	N	S	S	E	S	G	S
Translation of STRG.104	L	P	W	K	I	G	T	K	*	E	P	T	T	V	I	A	A	R	V	D	Q	Q	R	*	S	*	R	P	V	I	E	K
Translation of STRG.104	K	L	C	G	N	F	R	W	N	G	S	N	N	P	M	Q	W	Q	Y	C	*	T	*	P	I	H	V	M	Q	H	I	R
	g	s	g	a	v	l	r	g	s	s	s	t	s	m	e	d	w	n	q	m	r	t	h	n	s	n	s	s	e	s	g	s

							840										850										860				
Translation of AT2G435(	T	T	L	I	V	K	A	S	Y	R	E	D	T	V	R	F	K	F	E	P	S	V	G	C	P	Q	L	Y	K	E	V	G
Translation of AT2G435(	T	T	L	I	V	K	A	S	Y	R	E	D	T	V	R	F	K	F	E	P	S	V	G	C	P	Q	L	Y	K	E	V	G
Translation of AT2G435(	T	T	L	I	V	K	A	S	Y	R	E	D	T	V	R	F	K	F	E	P	S	V	G	C	P	Q	L	Y	K	E	V	G
Translation of STRG.104	T	L	Y	V	S	S	S	S	H	Q	L	G	V	L	S	S	T	K	K	L	E	N	V	L	N	C	R	T	G	R	F	S
Translation of STRG.104	F	I	K	W	L	R	R	S	S	A	W	K	L	I	Y	F	H	G	R	L	E	P	N	E	N	P	Q	Q	*	*	Q	R
	t	t	l	i	v	k	a	S	y	r	e	d	t	v	r	f	k	f	e	p	s	v	g	c	p	q	l	y	k	e	v	g

						870										880										890					
Translation of AT2G435(	K	R	F	K	L	Q	D	G	S	F	Q	L	K	Y	L	D	D	E	E	E	W	V	M	L	V	T	D	S	D	L	Q	E
Translation of AT2G435(	K	R	F	K	L	Q	D	G	S	F	Q	L	K	Y	L	D	D	E	E	E	W	V	M	L	V	T	D	S	D	L	Q	E
Translation of AT2G435(	K	R	F	K	L	Q	D	G	S	F	Q	L	K	Y	L	D	D	E	E	E	W	V	M	L	V	T	D	S	D	L	Q	E
Translation of STRG.104	*	S	T	W	M	M	K	K	N	G	*	C	W	L	Q	I	L	I	S	K	N	V	W	R	Y	Y	M	V	W	E	N	T
Translation of STRG.104	E	W	I	N	N	A	D	R	K	G	Q	L	*	R	R	H	C	T	F	Q	V	R	A	I	S	W	V	S	S	A	L	Q
	k	r	f	k	l	q	d	g	s	f	Q	l	k	y	l	d	d	e	e	e	w	v	m	l	v	t	d	s	d	l	q	e

				900										910										920							
Translation of AT2G435(	C	L	E	I	L	H	G	M	G	K	H	S	V	K	F	L	V	R	D	L	S	A	P	L	G	S	S	G	G	S	N	G
Translation of AT2G435(	C	L	E	I	L	H	G	M	G	K	H	S	V	K	F	L	V	R	D	L	S	A	P	L	G	S	S	G	G	S	N	G
Translation of AT2G435(	C	L	E	I	L	H	G	M	G	K	H	S	V	K	F	L	V	R	D	L	S	A	P	L	G	S	S	G	G	S	N	G
Translation of STRG.104	R	*	S	F	S	F	V	I	C	L	P	L	*	V	V	L	V	A	V	M	V	I	L	E	Q	A	Y	D	V	V	R	H
Translation of STRG.104	R	S	W	K	T	F	*	T	A	G	R	V	V	S	A	E	V	L	G	*	*	R	R	M	G	D	A	G	Y	R	F	*
	c	l	e	i	l	h	g	m	g	k	h	s	V	k	f	l	V	r	d	l	s	a	p	l	g	s	s	g	g	s	n	g

		930									940										950										960
Translation of AT2G435(	Y	L	G	T	G	L	*	-	-	-	-	-	-	-	-	-	-	-	-	-	-	-	-	-	-	-	-	-	-	-	-	-
Translation of AT2G435(	Y	L	G	T	G	L	*	-	-	-	-	-	-	-	-	-	-	-	-	-	-	-	-	-	-	-	-	-	-	-	-	-
Translation of AT2G435(	Y	L	G	T	G	L	*	-	-	-	-	-	-	-	-	-	-	-	-	-	-	-	-	-	-	-	-	-	-	-	-	-
Translation of STRG.104	R	H	T	Q	L	C	I	P	S	E	R	M	L	F	I	S	L	D	I	S	M	L	I	N	R	H	E	G	E	R	Q	F
Translation of STRG.104	S	P	R	M	F	G	D	I	T	W	Y	G	K	T	L	G	E	V	S	R	S	*	F	V	C	P	S	R	*	F	W	W
	y	l	g	t	g	l	X	X	X	X	X	X	X	X	X	X	X	X	X	X	X	L	X	X	X	X	X	X	E	X	X	X

									970										980										990		
Translation of AT2G435(	-	-	-	-	-	-	-	-	-	-	-	-	-	-	-	-	-	-	-	-	-	-	-	-	-	-	-	-	-	-	-	-
Translation of AT2G435(	-	-	-	-	-	-	-	-	-	-	-	-	-	-	-	-	-	-	-	-	-	-	-	-	-	-	-	-	-	-	-	-
Translation of AT2G435(	-	-	-	-	-	-	-	-	-	-	-	-	-	-	-	-	-	-	-	-	-	-	-	-	-	-	-	-	-	-	-	-
Translation of STRG.104	W	Y	S	G	V	Q	Q	K	M	Y	M	F	F	R	F	I	*	I	R	E	*	K	L	D	V	I	S	-	-	-	-	-
Translation of STRG.104	Q	*	W	L	S	W	N	R	L	M	T	S	*	D	I	D	T	H	S	Y	V	F	P	V	K	E	C	C	L	F	L	*
	X	Y	X	X	X	X	X	X	X	X	X	X	F	X	X	X	T	X	X	X	V	X	X	X	X	X	X	C	L	F	L	-

								1000										1010										1020				
Translation of AT2G435(	-	-	-	-	-	-	-	-	-	-	-	-	-	-	-	-	-	-	-	-	-	-	-	-	-	-	-	-	-	-	-	-
Translation of AT2G435(	-	-	-	-	-	-	-	-	-	-	-	-	-	-	-	-	-	-	-	-	-	-	-	-	-	-	-	-	-	-	-	-
Translation of AT2G435(	-	-	-	-	-	-	-	-	-	-	-	-	-	-	-	-	-	-	-	-	-	-	-	-	-	-	-	-	-	-	-	-
Translation of STRG.104	-	-	-	-	-	-	-	-	-	-	-	-	-	-	-	-	-	-	-	-	-	-	-	-	-	-	-	-	-	-	-	-
Translation of STRG.104	I	L	V	C	L	*	I	G	M	K	E	K	D	N	F	G	I	V	E	F	S	R	K	C	I	C	F	F	V	L	Y	E
	I	L	V	C	L	-	I	G	M	K	E	K	D	N	F	G	I	V	E	F	S	R	K	C	I	C	F	F	V	L	Y	E

						1030				
Translation of AT2G435(	-	-	-	-	-	-	-	-	-	-
Translation of AT2G435(	-	-	-	-	-	-	-	-	-	-
Translation of AT2G435(	-	-	-	-	-	-	-	-	-	-
Translation of STRG.104	-	-	-	-	-	-	-	-	-	-
Translation of STRG.104	S	E	N	K	S	W	M	L	Y	L
	S	E	N	K	S	W	M	L	Y	L
