# Supplementary material for: Concordant Gene Expression and Alternative Splicing Regulation under Abiotic Stresses in Arabidopsis
Source: Genes (Basel). 2024 May 23;15(6):675. doi: 10.3390/genes15060675 (PMC11202685; doi:10.3390/genes15060675)
Supplement: Supplementary file 1 [file genes-15-00675-s001.zip › Figure S45.pdf]

Figure S45. Multiple sequence alignment at the amino acid level for annotated and new isoforms of *A. thaliana* locus XLOC_008527 generated under different multifactorial stress combinations where isoforms AT2G43500.11 and STRG.10463.9 showed expression pattern HL↑, isoform STRG.10463.14 showed expression pattern all stress combinations↑, while expression of isoforms AT2G43500.9 and AT2G43500.10 was arbitrary. H = heat stress, L = high light stress. The figure emphasizes Exon 2 alignment as referred to in Figure S20.

Alignment Name: Untitled6
Length: 717

Start2

	1									10										20										30		
Translation of AT2G435(	-	-	-	-	-	-	-	-	-	-	-	-	-	-	-	-	-	-	-	-	-	-	-	-	-	-	-	-	-	-	-	-
Translation of AT2G435(	-	-	-	-	-	-	-	-	-	-	-	-	-	-	-	-	-	-	-	-	-	-	-	-	-	-	-	-	-	-	-	-
Translation of AT2G435(	-	-	-	-	-	-	-	-	-	-	-	-	-	-	-	-	-	-	-	-	-	-	-	-	-	-	-	-	-	-	-	-
Translation of STRG.104	-	-	-	-	-	-	-	-	-	-	-	-	-	-	-	-	-	-	-	-	-	-	-	-	-	-	L	P	G	Q	L	W
Translation of STRG.104	V	*	E	S	L	L	A	*	D	L	P	E	S	S	A	F	V	N	E	S	F	Q	G	S	S	G	D	S	L	L	W	L
	V	-	E	S	L	L	A	-	D	L	P	E	S	S	A	F	V	N	E	S	F	Q	G	S	S	G	X	X	X	X	X	X

End2/Start4 33/34 — End4/Start5 46/47

	33/34							40						46/47				50										60				
Translation of AT2G435(	-	A	V	N	L	R	T	A	A	I	P	R	P	Q	Y	L	S	S	S	Q	R	D	A	L	A	E	I	Q	D	V	L	R
Translation of AT2G435(	-	A	V	N	L	R	T	A	A	I	P	R	P	Q	Y	L	S	S	S	Q	R	D	A	L	A	E	I	Q	D	V	L	R
Translation of AT2G435(	-	A	V	N	L	R	T	A	A	I	P	R	P	Q	Y	L	S	S	S	Q	R	D	A	L	A	E	I	Q	D	V	L	R
Translation of STRG.104	R	Q	F	V	V	V	A	T	*	G	C	K	L	T	D	S	S	H	S	S	S	S	V	S	F	K	*	S	K	R	C	L
Translation of STRG.104	Q	P	E	A	V	N	L	R	T	A	A	I	P	R	P	Q	Y	L	S	S	S	Q	R	D	A	L	A	E	I	Q	D	V
	X	a	v	n	l	r	t	a	a	i	p	r	p	q	y	l	s	s	S	q	r	d	a	l	a	e	i	q	d	v	l	r

						70										80										90						
Translation of AT2G435(	T	V	C	H	A	H	K	L	P	L	A	L	A	W	I	P	C	R	K	D	Q	S	I	R	V	S	G	Q	K	S	G	E
Translation of AT2G435(	T	V	C	H	A	H	K	L	P	L	A	L	A	W	I	P	C	R	K	D	Q	S	I	R	V	S	G	Q	K	S	G	E
Translation of AT2G435(	T	V	C	H	A	H	K	L	P	L	A	L	A	W	I	P	C	R	K	D	Q	S	I	R	V	S	G	Q	K	S	G	E
Translation of STRG.104	S	*	N	T	R	C	S	S	N	S	M	S	C	T	Q	V	A	L	S	S	C	L	D	S	L	*	K	G	S	I	Y	K
Translation of STRG.104	L	R	T	V	C	H	A	H	K	L	P	L	A	L	A	W	I	P	C	R	K	D	Q	S	I	R	V	S	G	Q	K	S
	t	v	c	h	a	h	k	l	p	l	a	l	a	w	i	p	c	r	k	d	q	s	i	r	v	s	g	q	k	s	g	e

				100										110										120								
Translation of AT2G435(	N	C	I	L	C	I	E	E	T	A	C	Y	V	N	D	M	E	M	E	G	F	V	H	A	C	L	E	H	C	L	R	E
Translation of AT2G435(	N	C	I	L	C	I	E	E	T	A	C	Y	V	N	D	M	E	M	E	G	F	V	H	A	C	L	E	H	C	L	R	E
Translation of AT2G435(	N	C	I	L	C	I	E	E	T	A	C	Y	V	N	D	M	E	M	E	G	F	V	H	A	C	L	E	H	C	L	R	E
Translation of STRG.104	G	F	W	T	K	I	W	*	K	L	Y	T	L	H	R	G	D	S	L	L	C	E	R	Y	G	D	G	R	L	C	A	R
Translation of STRG.104	G	E	N	C	I	L	C	I	E	E	T	A	C	Y	V	N	D	M	E	M	E	G	F	V	H	A	C	L	E	H	C	L
	n	c	i	l	c	i	e	e	t	a	c	y	v	n	d	m	e	m	e	g	f	v	h	a	c	l	e	h	c	l	r	e

		130										140										150										160
Translation of AT2G435(	K	E	G	I	V	G	-	-	-	-	-	K	A	F	I	S	N	Q	P	F	F	S	S	D	V	K	A	Y	D	I	S	E
Translation of AT2G435(	K	E	G	I	V	G	-	-	-	-	-	K	A	F	I	S	N	Q	P	F	F	S	S	D	V	K	A	Y	D	I	S	E
Translation of AT2G435(	K	E	G	I	V	G	-	-	-	-	-	K	A	F	I	S	N	Q	P	F	F	S	S	D	V	K	A	Y	D	I	S	E
Translation of STRG.104	M	F	G	A	L	S	K	R	K	R	R	N	C	C	M	L	E	S	T	V	*	M	L	L	S	L	*	N	*	G	A	L
Translation of STRG.104	R	E	K	E	G	I	-	-	-	-	-	V	G	K	A	F	I	S	N	Q	P	F	F	S	S	D	V	K	A	Y	D	I
	k	e	g	i	v	g	K	R	K	R	R	k	a	f	i	s	n	q	p	f	f	s	s	d	v	k	a	y	d	i	s	e

										170										180										190		
Translation of AT2G435(	Y	P	I	V	Q	H	A	R	K	Y	G	L	N	A	A	V	A	I	K	L	R	S	T	Y	T	G	E	D	D	Y	I	L
Translation of AT2G435(	Y	P	I	V	Q	H	A	R	K	Y	G	L	N	A	A	V	A	I	K	L	R	S	T	Y	T	G	E	D	D	Y	I	L
Translation of AT2G435(	Y	P	I	V	Q	H	A	R	K	Y	G	L	N	A	A	V	A	I	K	L	R	S	T	Y	T	G	E	D	D	Y	I	L
Translation of STRG.104	T	L	V	K	M	I	T	Y	L	N	C	S	C	L	*	V	*	K	E	A	W	N	N	N	F	Y	*	T	A	F	R	V
Translation of STRG.104	S	E	Y	P	I	V	Q	H	A	R	K	Y	G	L	N	A	A	V	A	I	K	L	R	S	T	Y	T	G	E	D	D	Y
	y	p	i	v	q	h	a	r	k	y	g	l	n	a	a	v	A	i	k	l	r	s	t	y	t	g	e	d	d	y	i	l

Alignment Name: Untitled6
Length: 717

								200										210										220				
Translation of AT2G4350	E	L	F	L	P	V	S	M	K	G	S	L	E	Q	Q	L	L	L	D	S	L	S	G	T	M	Q	R	I	C	R	T	L
Translation of AT2G4350	E	L	F	L	P	V	S	M	K	G	S	L	E	Q	Q	L	L	L	D	S	L	S	G	T	M	Q	R	I	C	R	T	L
Translation of AT2G4350	E	L	F	L	P	V	S	M	K	G	S	L	E	Q	Q	L	L	L	D	S	L	S	G	T	M	Q	R	I	C	R	T	L
Translation of STRG.104	Q	C	R	E	F	V	E	L	*	E	L	F	Q	K	W	G	Q	L	K	K	K	G	L	N	L	D	F	G	V	V	I	C
Translation of STRG.104	I	L	E	L	F	L	P	V	S	M	K	G	S	L	E	Q	Q	L	L	L	D	S	L	S	G	T	M	Q	R	I	C	R
	e	l	f	l	p	v	s	m	k	g	s	l	e	q	q	l	l	L	d	s	l	s	g	t	m	q	r	i	c	r	t	l

						230										240										250						
Translation of AT2G4350	R	T	V	S	E	V	G	S	T	K	K	E	G	T	K	P	G	F	R	S	S	D	M	S	N	F	P	Q	T	T	S	S
Translation of AT2G4350	R	T	V	S	E	V	G	S	T	K	K	E	G	T	K	P	G	F	R	S	S	D	M	S	N	F	P	Q	T	T	S	S
Translation of AT2G4350	R	T	V	S	E	V	G	S	T	K	K	E	G	T	K	P	G	F	R	S	S	D	M	S	N	F	P	Q	T	T	S	S
Translation of STRG.104	L	I	S	R	R	Q	R	L	Q	K	-	-	I	F	R	Q	Y	H	W	I	P	S	L	T	L	L	E	A	C	F	R	V
Translation of STRG.104	T	L	R	T	V	S	E	V	G	S	T	K	K	E	G	T	K	P	G	F	R	S	S	D	M	S	N	F	P	Q	T	T
	r	t	v	s	e	v	g	s	t	k	k	e	g	t	k	p	g	f	r	s	s	d	m	s	n	f	p	q	t	t	s	s

				260										270										280								
Translation of AT2G4350	E	N	F	Q	T	I	S	L	D	S	E	F	N	S	T	R	S	M	F	S	G	M	S	S	D	K	E	N	S	I	T	V
Translation of AT2G4350	E	N	F	Q	T	I	S	L	D	S	E	F	N	S	T	R	S	M	F	S	G	M	S	S	D	K	E	N	S	I	T	V
Translation of AT2G4350	E	N	F	Q	T	I	S	L	D	S	E	F	N	S	T	R	S	M	F	S	G	M	S	S	D	K	E	N	S	I	T	V
Translation of STRG.104	C	P	L	I	K	K	T	V	S	Q	Y	L	K	A	L	W	S	R	M	*	A	K	Q	E	H	Q	R	R	R	K	A	L
Translation of STRG.104	S	S	E	N	F	Q	T	I	S	L	D	S	E	F	N	S	T	R	S	M	F	S	G	M	S	S	D	K	E	N	S	I
	e	n	f	q	t	i	s	l	d	s	e	f	n	s	t	r	s	m	f	s	g	m	s	s	d	k	e	n	s	i	t	v

		290										300										310										320
Translation of AT2G4350	S	Q	G	T	L	E	Q	D	V	S	K	A	R	T	P	E	K	K	K	S	T	T	E	K	N	V	S	L	S	A	L	Q
Translation of AT2G4350	S	Q	G	T	L	E	Q	D	V	S	K	A	R	T	P	E	K	K	K	S	T	T	E	K	N	V	S	L	S	A	L	Q
Translation of AT2G4350	S	Q	G	T	L	E	Q	D	V	S	K	A	R	T	P	E	K	K	K	S	T	T	E	K	N	V	S	L	S	A	L	Q
Translation of STRG.104	Q	R	K	M	*	A	*	A	L	S	N	N	T	S	L	G	V	*	R	M	L	Q	K	A	L	V	F	V	Q	L	H	*
Translation of STRG.104	T	V	S	Q	G	T	L	E	Q	D	V	S	K	A	R	T	P	E	K	K	K	S	T	T	E	K	N	V	S	L	S	A
	s	q	g	t	l	e	q	d	v	s	k	a	r	t	p	e	k	k	k	s	t	t	e	k	n	v	s	l	s	a	l	q

										330										340										350		
Translation of AT2G4350	Q	H	F	S	G	S	L	K	D	A	A	K	S	L	G	V	C	P	T	T	L	K	R	I	C	R	Q	H	G	I	M	R
Translation of AT2G4350	Q	H	F	S	G	S	L	K	D	A	A	K	S	L	G	V	C	P	T	T	L	K	R	I	C	R	Q	H	G	I	M	R
Translation of AT2G4350	Q	H	F	S	G	S	L	K	D	A	A	K	S	L	G	V	C	P	T	T	L	K	R	I	C	R	Q	H	G	I	M	R
Translation of STRG.104	N	G	Y	A	G	N	M	G	S	*	G	G	H	L	V	R	L	T	K	*	T	G	H	*	G	K	Y	R	R	Y	W	T
Translation of STRG.104	L	Q	Q	H	F	S	G	S	L	K	D	A	A	K	S	L	G	V	C	P	T	T	L	K	R	I	C	R	Q	H	G	I
	q	h	f	s	g	s	l	k	d	a	a	k	s	l	g	v	c	p	t	t	l	k	r	i	c	r	q	h	g	i	m	r

								360										370										380				
Translation of AT2G4350	W	P	S	R	K	I	N	-	K	V	N	R	S	L	R	K	I	Q	T	V	L	D	S	V	Q	G	V	E	G	G	L	K
Translation of AT2G4350	W	P	S	R	K	I	N	-	K	V	N	R	S	L	R	K	I	Q	T	V	L	D	S	V	Q	G	V	E	G	G	L	K
Translation of AT2G4350	W	P	S	R	K	I	N	-	K	V	N	R	S	L	R	K	I	Q	T	V	L	D	S	V	Q	G	V	E	G	G	L	K
Translation of STRG.104	R	S	K	V	*	K	E	D	*	S	S	T	Q	Q	L	A	N	S	L	Q	L	D	L	L	F	K	K	L	I	P	K	R
Translation of STRG.104	M	R	W	P	S	R	K	-	I	N	K	V	N	R	S	L	R	K	I	Q	T	V	L	D	S	V	Q	G	V	E	G	G
	w	p	s	r	k	i	n	D	k	v	n	r	s	l	r	k	i	q	t	v	l	d	s	v	q	g	v	e	g	g	l	k

Alignment Name: Untitled6
Length: 717

						390										400										410						
Translation of AT2G435	F	D	S	A	T	G	E	F	I	A	V	R	P	F	I	Q	E	I	D	T	Q	K	G	L	S	S	L	D	N	D	A	H
Translation of AT2G435	F	D	S	A	T	G	E	F	I	A	V	R	P	F	I	Q	E	I	D	T	Q	K	G	L	S	S	L	D	N	D	A	H
Translation of AT2G435	F	D	S	A	T	G	E	F	I	A	V	R	P	F	I	Q	E	I	D	T	Q	K	G	L	S	S	L	D	N	D	A	H
Translation of STRG.104	V	C	R	L	L	I	M	M	H	M	Q	E	E	V	R	R	I	C	L	T	I	L	H	S	S	S	R	K	L	N	L	S
Translation of STRG.104	L	K	F	D	S	A	T	G	E	F	I	A	V	R	P	F	I	Q	E	I	D	T	Q	K	G	L	S	S	L	D	N	D
	f	d	s	a	t	g	e	f	i	a	v	r	p	f	i	q	e	i	d	t	q	k	g	l	s	s	l	d	n	d	a	h

				420										430										440								
Translation of AT2G435	A	R	R	S	Q	E	D	M	P	D	D	T	S	F	K	L	Q	E	A	K	S	V	D	N	A	I	K	L	E	E	D	T
Translation of AT2G435	A	R	R	S	Q	E	D	M	P	D	D	T	S	F	K	L	Q	E	A	K	S	V	D	N	A	I	K	L	E	E	D	T
Translation of AT2G435	A	R	R	S	Q	E	D	M	P	D	D	T	S	F	K	L	Q	E	A	K	S	V	D	N	A	I	K	L	E	E	D	T
Translation of STRG.104	T	M	P	L	S	*	R	R	I	Q	P	*	I	K	Q	D	Q	D	H	S	W	R	L	M	L	V	V	S	H	G	L	G
Translation of STRG.104	A	H	A	R	R	S	Q	E	D	M	P	D	D	T	S	F	K	L	Q	E	A	K	S	V	D	N	A	I	K	L	E	E
	a	r	r	s	q	e	d	m	p	d	d	t	s	f	k	l	q	e	a	k	s	v	d	n	a	i	k	l	e	e	d	t

		450										460										470										480
Translation of AT2G435	T	M	N	Q	A	R	P	G	S	F	M	E	V	N	A	S	G	Q	P	W	A	W	M	A	K	E	S	G	L	N	G	S
Translation of AT2G435	T	M	N	Q	A	R	P	G	S	F	M	E	V	N	A	S	G	Q	P	W	A	W	M	A	K	E	S	G	L	N	G	S
Translation of AT2G435	T	M	N	Q	A	R	P	G	S	F	M	E	V	N	A	S	G	Q	P	W	A	W	M	A	K	E	S	G	L	N	G	S
Translation of STRG.104	W	P	K	S	L	A	*	M	A	V	K	E	*	R	A	F	A	T	*	A	L	W	K	F	Q	M	E	W	I	Q	Q	S
Translation of STRG.104	D	T	T	M	N	Q	A	R	P	G	S	F	M	E	V	N	A	S	G	Q	P	W	A	W	M	A	K	E	S	G	L	N
	t	m	n	q	a	r	p	g	s	f	m	e	v	n	a	s	g	q	p	w	a	W	m	a	k	e	s	g	l	n	g	s

										490										500										510		
Translation of AT2G435	E	G	I	K	S	V	C	N	L	S	S	V	E	I	S	D	G	M	D	P	T	I	R	C	S	G	S	I	V	E	P	N
Translation of AT2G435	E	G	I	K	S	V	C	N	L	S	S	V	E	I	S	D	G	M	D	P	T	I	R	C	S	G	S	I	V	E	P	N
Translation of AT2G435	E	G	I	K	S	V	C	N	L	S	S	V	E	I	S	D	G	M	D	P	T	I	R	C	S	G	S	I	V	E	P	N
Translation of STRG.104	D	A	V	A	V	L	L	N	L	T	N	P	C	H	A	A	Y	Q	I	H	Q	M	A	Q	A	Q	F	C	V	E	A	H
Translation of STRG.104	G	S	E	G	I	K	S	V	C	N	L	S	S	V	E	I	S	D	G	M	D	P	T	I	R	C	S	G	S	I	V	E
	e	g	i	k	s	v	c	n	l	s	s	v	e	i	s	d	g	m	d	p	t	i	r	c	s	g	s	i	v	e	p	n

								520										530										540				
Translation of AT2G435	Q	S	M	S	C	S	I	S	D	S	S	N	G	S	G	A	V	L	R	G	S	S	S	T	S	M	E	D	W	N	Q	M
Translation of AT2G435	Q	S	M	S	C	S	I	S	D	S	S	N	G	S	G	A	V	L	R	G	S	S	S	T	S	M	E	D	W	N	Q	M
Translation of AT2G435	Q	S	M	S	C	S	I	S	D	S	S	N	G	S	G	A	V	L	R	G	S	S	S	T	S	M	E	D	W	N	Q	M
Translation of STRG.104	L	L	P	W	K	I	G	T	K	*	E	P	T	T	V	I	A	A	R	V	D	Q	Q	R	*	S	*	R	P	V	I	E
Translation of STRG.104	P	N	Q	S	M	S	C	S	I	S	D	S	S	N	G	S	G	A	V	L	R	G	S	S	S	T	S	M	E	D	W	N
	q	s	m	s	c	s	i	s	d	S	s	n	g	s	g	a	v	l	r	g	s	s	s	t	S	m	e	d	w	n	q	m

						550										560										570						
Translation of AT2G435	R	T	H	N	S	N	S	S	E	S	G	-	-	S	T	T	L	I	V	K	A	S	Y	R	E	D	T	V	R	F	K	F
Translation of AT2G435	R	T	H	N	S	N	S	S	E	S	G	-	-	S	T	T	L	I	V	K	A	S	Y	R	E	D	T	V	R	F	K	F
Translation of AT2G435	R	T	H	N	S	N	S	S	E	S	G	-	-	S	T	T	L	I	V	K	A	S	Y	R	E	D	T	V	R	F	K	F
Translation of STRG.104	K	T	L	Y	V	S	S	S	S	H	Q	L	G	V	L	S	S	T	K	K	L	E	N	V	L	N	C	R	T	G	R	F
Translation of STRG.104	Q	M	R	T	H	N	S	N	S	S	E	-	-	S	G	S	T	T	L	I	V	K	A	S	Y	R	E	D	T	V	R	F
	r	t	h	n	s	n	S	s	e	s	g	L	G	s	t	t	l	i	v	k	a	s	y	r	e	d	t	v	r	f	k	F

Alignment Name: Untitled6
Length: 717

				580										590										600								
Translation of AT2G4350	E	P	S	V	G	C	P	Q	L	Y	K	E	V	G	K	R	F	K	L	Q	D	G	S	F	Q	L	K	Y	L	D	D	E
Translation of AT2G4350	E	P	S	V	G	C	P	Q	L	Y	K	E	V	G	K	R	F	K	L	Q	D	G	S	F	Q	L	K	Y	L	D	D	E
Translation of AT2G4350	E	P	S	V	G	C	P	Q	L	Y	K	E	V	G	K	R	F	K	L	Q	D	G	S	F	Q	L	K	Y	L	D	D	E
Translation of STRG.104	S	*	S	T	W	M	M	K	K	N	G	*	C	W	L	Q	I	L	I	S	K	N	V	W	R	Y	Y	M	V	W	E	N
Translation of STRG.104	K	F	E	P	S	V	G	C	P	Q	L	Y	K	E	V	G	K	R	F	K	L	Q	D	G	S	F	Q	L	K	Y	L	D
	e	p	s	v	g	c	p	q	l	y	k	e	v	g	k	r	f	k	l	q	d	g	s	f	q	l	k	y	l	d	d	e

		610										620										630										640
Translation of AT2G4350	E	E	W	V	M	L	V	T	D	S	D	L	Q	E	C	L	E	I	L	H	G	M	G	K	H	S	V	K	F	L	V	R
Translation of AT2G4350	E	E	W	V	M	L	V	T	D	S	D	L	Q	E	C	L	E	I	L	H	G	M	G	K	H	S	V	K	F	L	V	R
Translation of AT2G4350	E	E	W	V	M	L	V	T	D	S	D	L	Q	E	C	L	E	I	L	H	G	M	G	K	H	S	V	K	F	L	V	R
Translation of STRG.104	T	R	*	S	F	S	F	V	I	C	L	P	L	*	V	V	L	V	A	V	M	V	I	L	E	Q	A	Y	D	V	V	R
Translation of STRG.104	D	E	E	E	W	V	M	L	V	T	D	S	D	L	Q	E	C	L	E	I	L	H	G	M	G	K	H	S	V	K	F	L
	e	e	w	v	m	l	v	t	d	s	d	l	q	e	c	l	e	i	l	h	g	m	g	k	h	s	v	k	f	l	v	r

										650										660										670		
Translation of AT2G4350	D	L	S	A	P	L	G	S	S	G	G	S	N	G	Y	L	G	T	G	L	-	-	-	-	-	-	-	-	-	-	-	-
Translation of AT2G4350	D	L	S	A	P	L	G	S	S	G	G	S	N	G	Y	L	G	T	G	L	-	-	-	-	-	-	-	-	-	-	-	-
Translation of AT2G4350	D	L	S	A	P	L	G	S	S	G	G	S	N	G	Y	L	G	T	G	L	-	-	-	-	-	-	-	-	-	-	-	-
Translation of STRG.104	H	R	H	T	Q	L	C	I	P	S	E	R	M	L	F	I	S	L	D	I	S	M	L	I	-	-	-	-	-	-	-	-
Translation of STRG.104	V	R	D	L	S	A	P	L	G	S	S	G	G	S	N	G	Y	L	G	T	G	L	*	R	R	K	T	*	T	H	T	V
	d	l	s	a	p	l	g	s	s	g	g	s	n	g	y	l	g	t	g	l	X	X	L	X	R	K	T	-	T	H	T	V

								680										690										700				
Translation of AT2G4350	-	-	-	-	-	-	-	-	-	-	-	-	-	-	-	-	-	-	-	-	-	-	-	-	-	-	-	-	-	-	-	-
Translation of AT2G4350	-	-	-	-	-	-	-	-	-	-	-	-	-	-	-	-	-	-	-	-	-	-	-	-	-	-	-	-	-	-	-	-
Translation of AT2G4350	-	-	-	-	-	-	-	-	-	-	-	-	-	-	-	-	-	-	-	-	-	-	-	-	-	-	-	-	-	-	-	-
Translation of STRG.104	-	-	-	-	-	-	-	-	-	-	-	-	-	-	-	-	-	-	-	-	-	-	-	-	-	-	-	-	-	-	-	-
Translation of STRG.104	M	Y	S	Q	*	K	N	V	V	Y	F	S	R	Y	*	Y	A	Y	K	*	A	*	R	R	K	T	I	L	V	*	W	S
	M	Y	S	Q	-	K	N	V	V	Y	F	S	R	Y	-	Y	A	Y	K	-	A	-	R	R	K	T	I	L	V	-	W	S

						710							
Translation of AT2G4350	-	-	-	-	-	-	-	-	-	-	-	-	-
Translation of AT2G4350	-	-	-	-	-	-	-	-	-	-	-	-	-
Translation of AT2G4350	-	-	-	-	-	-	-	-	-	-	-	-	-
Translation of STRG.104	-	-	-	-	-	-	-	-	-	-	-	-	-
Translation of STRG.104	S	A	E	N	V	Y	V	F	S	F	Y	M	N
	S	A	E	N	V	Y	V	F	S	F	Y	M	N
