# Supplementary material for: Concordant Gene Expression and Alternative Splicing Regulation under Abiotic Stresses in Arabidopsis
Source: Genes (Basel). 2024 May 23;15(6):675. doi: 10.3390/genes15060675 (PMC11202685; doi:10.3390/genes15060675)
Supplement: Supplementary file 1 [file genes-15-00675-s001.zip › Figure S46.pdf]

Figure S46. Multiple sequence alignment at the amino acid level for annotated and new isoforms of *A. thaliana* locus XLOC_008527 generated under different multifactorial stress combinations where isoforms AT2G43500.11 and STRG.10463.9 showed expression pattern HL↑, isoform STRG.10463.14 showed expression pattern all stress combinations↑, while expression of isoforms AT2G43500.9 and AT2G43500.10 was arbitrary. H = heat stress, L = high light stress. The figure emphasizes Exons 3 and 4 alignment as referred to in Figure S20.

Alignment Name: Untitled8
Length: 702

Start3/Exon skipping 1 — End3/Start4 15/16 — End4/Start5 28/29

										10										20										30		
Translation of AT2G435(	-	-	-	-	-	-	-	-	-	-	-	-	-	-	-	A	V	N	L	R	T	A	A	I	P	R	P	Q	Y	L	S	S
Translation of AT2G435(	-	-	-	-	-	-	-	-	-	-	-	-	-	-	-	A	V	N	L	R	T	A	A	I	P	R	P	Q	Y	L	S	S
Translation of AT2G435(	-	-	-	-	-	-	-	-	-	-	-	-	-	-	-	A	V	N	L	R	T	A	A	I	P	R	P	Q	Y	L	S	S
Translation of STRG.104	F	Q	G	S	S	G	D	S	L	L	W	L	Q	P	E	A	V	N	L	R	T	A	A	I	P	R	P	Q	Y	L	S	S
Translation of STRG.104	F	Q	G	S	S	G	D	S	L	L	W	L	Q	P	E	A	V	N	L	R	T	A	A	I	P	R	P	Q	Y	L	S	S
	F	Q	G	S	S	G	D	S	L	L	W	L	Q	P	E	A	V	N	L	R	T	A	A	I	P	R	P	Q	Y	L	S	S

								40										50										60				
Translation of AT2G435(	S	Q	R	D	A	L	A	E	I	Q	D	V	L	R	T	V	C	H	A	H	K	L	P	L	A	L	A	W	I	P	C	R
Translation of AT2G435(	S	Q	R	D	A	L	A	E	I	Q	D	V	L	R	T	V	C	H	A	H	K	L	P	L	A	L	A	W	I	P	C	R
Translation of AT2G435(	S	Q	R	D	A	L	A	E	I	Q	D	V	L	R	T	V	C	H	A	H	K	L	P	L	A	L	A	W	I	P	C	R
Translation of STRG.104	S	Q	R	D	A	L	A	E	I	Q	D	V	L	R	T	V	C	H	A	H	K	L	P	L	A	L	A	W	I	P	C	R
Translation of STRG.104	S	Q	R	D	A	L	A	E	I	Q	D	V	L	R	T	V	C	H	A	H	K	L	P	L	A	L	A	W	I	P	C	R
	S	Q	R	D	A	L	A	E	I	Q	D	V	L	R	T	V	C	H	A	H	K	L	P	L	A	L	A	W	I	P	C	R

						70										80										90						
Translation of AT2G435(	K	D	Q	S	I	R	V	S	G	Q	K	S	G	E	N	C	I	L	C	I	E	E	T	A	C	Y	V	N	D	M	E	M
Translation of AT2G435(	K	D	Q	S	I	R	V	S	G	Q	K	S	G	E	N	C	I	L	C	I	E	E	T	A	C	Y	V	N	D	M	E	M
Translation of AT2G435(	K	D	Q	S	I	R	V	S	G	Q	K	S	G	E	N	C	I	L	C	I	E	E	T	A	C	Y	V	N	D	M	E	M
Translation of STRG.104	K	D	Q	S	I	R	V	S	G	Q	K	S	G	E	N	C	I	L	C	I	E	E	T	A	C	Y	V	N	D	M	E	M
Translation of STRG.104	K	D	Q	S	I	R	V	S	G	Q	K	S	G	E	N	C	I	L	C	I	E	E	T	A	C	Y	V	N	D	M	E	M
	K	D	Q	S	I	R	V	S	G	Q	K	S	G	E	N	C	I	L	C	I	E	E	T	A	C	Y	V	N	D	M	E	M

Intron retention/Frame shift 117

				100										110										120								
Translation of AT2G435(	E	G	F	V	H	A	C	L	E	H	C	L	R	E	K	E	G	I	V	G	K	A	F	I	S	N	Q	P	F	F	S	S
Translation of AT2G435(	E	G	F	V	H	A	C	L	E	H	C	L	R	E	K	E	G	I	V	G	K	A	F	I	S	N	Q	P	F	F	S	S
Translation of AT2G435(	E	G	F	V	H	A	C	L	E	H	C	L	R	E	K	E	G	I	V	G	K	A	F	I	S	N	Q	P	F	F	S	S
Translation of STRG.104	E	G	F	V	H	A	C	L	E	H	C	L	R	E	K	E	G	I	V	A	C	S	K	V	R	S	E	C	C	C	R	Y
Translation of STRG.104	E	G	F	V	H	A	C	L	E	H	C	L	R	E	K	E	G	I	V	G	K	A	F	I	S	N	Q	P	F	F	S	S
	E	G	F	V	H	A	C	L	E	H	C	L	R	E	K	E	G	I	V	g	k	a	f	i	s	n	q	p	f	f	s	s

		130										140										150										160
Translation of AT2G435(	D	V	K	A	Y	D	I	S	E	Y	P	I	V	Q	H	A	R	K	Y	G	L	N	A	A	V	A	I	K	L	R	S	T
Translation of AT2G435(	D	V	K	A	Y	D	I	S	E	Y	P	I	V	Q	H	A	R	K	Y	G	L	N	A	A	V	A	I	K	L	R	S	T
Translation of AT2G435(	D	V	K	A	Y	D	I	S	E	Y	P	I	V	Q	H	A	R	K	Y	G	L	N	A	A	V	A	I	K	L	R	S	T
Translation of STRG.104	K	T	E	E	H	L	H	W	*	R	*	L	H	T	*	T	V	L	A	C	K	Y	E	R	K	L	G	T	T	T	S	I
Translation of STRG.104	D	V	K	A	Y	D	I	S	E	Y	P	I	V	Q	H	A	R	K	Y	G	L	N	A	A	V	A	I	K	L	R	S	T
	d	v	k	a	y	d	i	s	E	y	P	i	v	q	H	a	r	k	y	g	l	n	a	a	v	a	i	k	l	r	S	t

										170										180										190		
Translation of AT2G435(	Y	T	G	E	D	D	Y	I	L	E	L	F	L	P	V	S	M	K	G	S	L	E	Q	Q	L	L	L	D	S	L	S	G
Translation of AT2G435(	Y	T	G	E	D	D	Y	I	L	E	L	F	L	P	V	S	M	K	G	S	L	E	Q	Q	L	L	L	D	S	L	S	G
Translation of AT2G435(	Y	T	G	E	D	D	Y	I	L	E	L	F	L	P	V	S	M	K	G	S	L	E	Q	Q	L	L	L	D	S	L	S	G
Translation of STRG.104	R	Q	P	F	G	Y	N	A	E	N	L	S	N	F	E	N	C	F	R	S	G	V	N	*	K	R	R	D	*	T	W	I
Translation of STRG.104	Y	T	G	E	D	D	Y	I	L	E	L	F	L	P	V	S	M	K	G	S	L	E	Q	Q	L	L	L	D	S	L	S	G
	y	t	g	e	d	d	y	i	l	e	L	f	l	p	v	s	m	k	g	S	l	e	q	Q	l	l	l	D	S	l	s	g

Alignment Name: Untitled8
Length: 702

								200										210										220				
Translation of AT2G435(	T	M	Q	R	I	C	R	T	L	R	T	V	S	E	V	G	S	T	K	K	E	G	T	K	P	G	F	R	S	S	D	M
Translation of AT2G435(	T	M	Q	R	I	C	R	T	L	R	T	V	S	E	V	G	S	T	K	K	E	G	T	K	P	G	F	R	S	S	D	M
Translation of AT2G435(	T	M	Q	R	I	C	R	T	L	R	T	V	S	E	V	G	S	T	K	K	E	G	T	K	P	G	F	R	S	S	D	M
Translation of STRG.104	S	E	*	*	Y	V	*	F	P	A	D	N	V	F	R	K	F	S	D	N	I	I	G	F	R	V	*	L	Y	*	K	H
Translation of STRG.104	T	M	Q	R	I	C	R	T	L	R	T	V	S	E	V	G	S	T	K	K	E	G	T	K	P	G	F	R	S	S	D	M
	t	m	Q	R	i	c	R	t	l	r	t	v	s	e	v	g	s	t	k	k	e	g	t	k	p	g	F	r	s	S	d	m

						230										240										250						
Translation of AT2G435(	S	N	F	P	Q	T	T	S	S	E	N	F	Q	T	I	S	L	D	S	E	F	N	S	T	R	S	M	F	S	G	M	S
Translation of AT2G435(	S	N	F	P	Q	T	T	S	S	E	N	F	Q	T	I	S	L	D	S	E	F	N	S	T	R	S	M	F	S	G	M	S
Translation of AT2G435(	S	N	F	P	Q	T	T	S	S	E	N	F	Q	T	I	S	L	D	S	E	F	N	S	T	R	S	M	F	S	G	M	S
Translation of STRG.104	V	F	G	Y	V	L	*	*	R	K	Q	Y	H	S	I	S	R	H	F	G	A	G	C	E	Q	S	K	N	T	R	E	E
Translation of STRG.104	S	N	F	P	Q	T	T	S	S	E	N	F	Q	T	I	S	L	D	S	E	F	N	S	T	R	S	M	F	S	G	M	S
	s	n	f	p	q	t	T	S	s	e	n	f	q	t	I	S	l	d	s	e	f	n	s	t	r	S	m	f	s	g	m	s

				260										270										280								
Translation of AT2G435(	S	D	K	E	N	S	I	T	V	S	Q	G	T	L	E	Q	D	V	S	K	A	R	T	P	E	K	K	K	S	T	T	E
Translation of AT2G435(	S	D	K	E	N	S	I	T	V	S	Q	G	T	L	E	Q	D	V	S	K	A	R	T	P	E	K	K	K	S	T	T	E
Translation of AT2G435(	S	D	K	E	N	S	I	T	V	S	Q	G	T	L	E	Q	D	V	S	K	A	R	T	P	E	K	K	K	S	T	T	E
Translation of STRG.104	E	K	H	Y	R	E	K	C	E	L	K	R	S	P	T	T	L	L	W	E	S	K	G	C	C	K	K	P	W	C	L	S
Translation of STRG.104	S	D	K	E	N	S	I	T	V	S	Q	G	T	L	E	Q	D	V	S	K	A	R	T	P	E	K	K	K	S	T	T	E
	s	d	k	e	n	s	i	t	v	s	q	g	t	l	e	q	d	v	s	k	a	r	t	p	e	K	K	k	s	t	t	e

		290										300										310										320
Translation of AT2G435(	K	N	V	S	L	S	A	L	Q	Q	H	F	S	G	S	L	K	D	A	A	K	S	L	G	V	C	P	-	-	-	-	-
Translation of AT2G435(	K	N	V	S	L	S	A	L	Q	Q	H	F	S	G	S	L	K	D	A	A	K	S	L	G	V	C	P	-	-	-	-	-
Translation of AT2G435(	K	N	V	S	L	S	A	L	Q	Q	H	F	S	G	S	L	K	D	A	A	K	S	L	G	V	C	P	-	-	-	-	-
Translation of STRG.104	N	Y	I	E	T	D	M	Q	A	T	W	D	H	E	V	A	I	S	*	D	*	Q	S	E	Q	V	T	K	E	N	T	D
Translation of STRG.104	K	N	V	S	L	S	A	L	Q	Q	H	F	S	G	S	L	K	D	A	A	K	S	L	G	V	C	P	-	-	-	-	-
	k	n	v	s	l	s	a	l	q	q	h	f	s	g	s	l	k	d	A	a	K	s	l	g	v	c	p	K	E	N	T	D

									330										340										350			
Translation of AT2G435(	-	-	T	T	L	K	R	I	C	R	Q	H	G	I	M	R	W	P	S	R	K	I	N	K	V	N	R	S	L	R	K	I
Translation of AT2G435(	-	-	T	T	L	K	R	I	C	R	Q	H	G	I	M	R	W	P	S	R	K	I	N	K	V	N	R	S	L	R	K	I
Translation of AT2G435(	-	-	T	T	L	K	R	I	C	R	Q	H	G	I	M	R	W	P	S	R	K	I	N	K	V	N	R	S	L	R	K	I
Translation of STRG.104	G	T	G	L	G	P	R	C	R	R	R	T	K	V	R	L	S	N	W	R	I	H	C	S	*	T	F	Y	S	R	N	*
Translation of STRG.104	-	-	T	T	L	K	R	I	C	R	Q	H	G	I	M	R	W	P	S	R	K	I	N	K	V	N	R	S	L	R	K	I
	G	T	t	t	l	k	R	i	c	R	q	h	g	i	m	r	w	p	s	R	k	i	n	k	V	n	r	s	l	R	k	I

								360										370										380				
Translation of AT2G435(	Q	T	V	L	D	S	V	Q	G	V	E	G	G	L	K	F	D	S	A	T	G	-	-	-	-	E	F	I	A	V	R	P
Translation of AT2G435(	Q	T	V	L	D	S	V	Q	G	V	E	G	G	L	K	F	D	S	A	T	G	-	-	-	-	E	F	I	A	V	R	P
Translation of AT2G435(	Q	T	V	L	D	S	V	Q	G	V	E	G	G	L	K	F	D	S	A	T	G	-	-	-	-	E	F	I	A	V	R	P
Translation of STRG.104	Y	P	K	G	S	V	V	S	*	*	*	C	T	C	K	K	K	S	G	G	Y	A	*	R	Y	F	I	Q	A	P	G	S
Translation of STRG.104	Q	T	V	L	D	S	V	Q	G	V	E	G	G	L	K	F	D	S	A	T	G	-	-	-	-	E	F	I	A	V	R	P
	q	t	v	l	d	s	V	q	G	V	E	g	g	l	K	f	d	S	a	t	g	A	-	R	Y	e	f	i	A	v	r	p

Alignment Name: Untitled8
Length: 702

						390										400										410						
Translation of AT2G435(	F	I	Q	E	I	D	T	Q	K	G	L	S	S	L	D	N	D	A	H	A	R	R	S	Q	E	D	M	P	D	D	T	S
Translation of AT2G435(	F	I	Q	E	I	D	T	Q	K	G	L	S	S	L	D	N	D	A	H	A	R	R	S	Q	E	D	M	P	D	D	T	S
Translation of AT2G435(	F	I	Q	E	I	D	T	Q	K	G	L	S	S	L	D	N	D	A	H	A	R	R	S	Q	E	D	M	P	D	D	T	S
Translation of STRG.104	*	I	C	R	Q	C	H	*	V	R	G	G	Y	N	H	E	S	S	K	T	R	I	I	H	G	G	*	C	*	W	S	A
Translation of STRG.104	F	I	Q	E	I	D	T	Q	K	G	L	S	S	L	D	N	D	A	H	A	R	R	S	Q	E	D	M	P	D	D	T	S
	F	I	q	e	i	d	t	Q	k	g	l	s	s	l	d	n	d	a	h	a	R	r	s	q	e	d	M	p	D	d	t	s

				420										430										440								
Translation of AT2G435(	F	K	L	Q	E	A	K	S	V	D	N	A	I	K	L	E	E	D	T	T	M	N	Q	A	R	P	G	S	F	M	E	V
Translation of AT2G435(	F	K	L	Q	E	A	K	S	V	D	N	A	I	K	L	E	E	D	T	T	M	N	Q	A	R	P	G	S	F	M	E	V
Translation of AT2G435(	F	K	L	Q	E	A	K	S	V	D	N	A	I	K	L	E	E	D	T	T	M	N	Q	A	R	P	G	S	F	M	E	V
Translation of STRG.104	M	G	L	D	G	Q	R	-	V	W	L	E	W	Q	*	R	N	K	E	R	L	Q	L	K	L	C	G	N	F	R	W	N
Translation of STRG.104	F	K	L	Q	E	A	K	S	V	D	N	A	I	K	L	E	E	D	T	T	M	N	Q	A	R	P	G	S	F	M	E	V
	f	k	L	q	e	a	k	S	V	d	n	a	i	k	L	e	e	d	t	t	m	n	q	a	r	p	G	s	F	m	e	v

		450										460										470										480
Translation of AT2G435(	N	A	S	G	Q	P	W	A	W	M	A	K	E	S	G	L	N	G	S	E	G	I	K	S	V	C	N	L	S	S	V	E
Translation of AT2G435(	N	A	S	G	Q	P	W	A	W	M	A	K	E	S	G	L	N	G	S	E	G	I	K	S	V	C	N	L	S	S	V	E
Translation of AT2G435(	N	A	S	G	Q	P	W	A	W	M	A	K	E	S	G	L	N	G	S	E	G	I	K	S	V	C	N	L	S	S	V	E
Translation of STRG.104	G	S	N	N	P	M	Q	W	Q	Y	C	*	T	*	P	I	H	V	M	Q	H	I	R	-	-	-	-	-	F	I	K	W
Translation of STRG.104	N	A	S	G	Q	P	W	A	W	M	A	K	E	S	G	L	N	G	S	E	G	I	K	S	V	C	N	L	S	S	V	E
	n	a	s	g	q	p	w	a	w	m	a	K	e	S	g	l	n	g	s	e	g	I	k	S	V	C	N	L	s	s	v	e

										490										500										510		
Translation of AT2G435(	I	S	D	G	M	D	P	T	I	R	C	S	G	S	I	V	E	P	N	Q	S	M	S	C	S	I	S	D	S	S	N	G
Translation of AT2G435(	I	S	D	G	M	D	P	T	I	R	C	S	G	S	I	V	E	P	N	Q	S	M	S	C	S	I	S	D	S	S	N	G
Translation of AT2G435(	I	S	D	G	M	D	P	T	I	R	C	S	G	S	I	V	E	P	N	Q	S	M	S	C	S	I	S	D	S	S	N	G
Translation of STRG.104	L	R	R	S	S	A	W	K	L	I	Y	F	H	G	R	L	E	P	N	E	N	P	Q	Q	*	*	Q	R	E	W	I	N
Translation of STRG.104	I	S	D	G	M	D	P	T	I	R	C	S	G	S	I	V	E	P	N	Q	S	M	S	C	S	I	S	D	S	S	N	G
	i	s	d	g	m	d	p	t	i	r	c	s	g	s	i	v	E	P	N	q	s	m	s	c	S	I	s	d	s	s	n	g

								520										530										540				
Translation of AT2G435(	S	G	A	V	L	R	G	S	S	S	T	S	M	E	D	W	N	Q	M	R	T	H	N	S	N	S	S	E	S	G	S	T
Translation of AT2G435(	S	G	A	V	L	R	G	S	S	S	T	S	M	E	D	W	N	Q	M	R	T	H	N	S	N	S	S	E	S	G	S	T
Translation of AT2G435(	S	G	A	V	L	R	G	S	S	S	T	S	M	E	D	W	N	Q	M	R	T	H	N	S	N	S	S	E	S	G	S	T
Translation of STRG.104	N	A	D	R	K	G	Q	L	*	R	R	H	C	T	F	Q	V	R	A	I	S	W	V	S	S	A	L	Q	R	S	W	K
Translation of STRG.104	S	G	A	V	L	R	G	S	S	S	T	S	M	E	D	W	N	Q	M	R	T	H	N	S	N	S	S	E	S	G	S	T
	s	g	a	v	l	r	g	s	S	s	t	s	m	e	d	w	n	q	m	r	t	h	n	S	n	s	s	e	s	g	s	t

						550										560										570						
Translation of AT2G435(	T	L	I	V	K	A	S	Y	R	E	D	T	V	R	F	K	F	E	P	S	V	G	C	P	Q	L	Y	K	E	V	G	K
Translation of AT2G435(	T	L	I	V	K	A	S	Y	R	E	D	T	V	R	F	K	F	E	P	S	V	G	C	P	Q	L	Y	K	E	V	G	K
Translation of AT2G435(	T	L	I	V	K	A	S	Y	R	E	D	T	V	R	F	K	F	E	P	S	V	G	C	P	Q	L	Y	K	E	V	G	K
Translation of STRG.104	T	F	*	T	A	G	R	V	V	S	A	E	V	L	G	*	*	R	R	M	G	D	A	G	Y	R	F	*	S	P	-	-
Translation of STRG.104	T	L	I	V	K	A	S	Y	R	E	D	T	V	R	F	K	F	E	P	S	V	G	C	P	Q	L	Y	K	E	V	G	K
	T	l	I	v	k	a	s	y	r	e	d	t	V	r	f	K	F	e	p	s	v	g	c	p	q	l	y	K	e	v	G	K

Alignment Name: Untitled8
Length: 702

				580										590										600								
Translation of AT2G435(	R	F	K	L	Q	D	G	S	F	Q	L	K	Y	L	D	D	E	E	E	W	V	M	L	V	T	D	S	D	L	Q	E	C
Translation of AT2G435(	R	F	K	L	Q	D	G	S	F	Q	L	K	Y	L	D	D	E	E	E	W	V	M	L	V	T	D	S	D	L	Q	E	C
Translation of AT2G435(	R	F	K	L	Q	D	G	S	F	Q	L	K	Y	L	D	D	E	E	E	W	V	M	L	V	T	D	S	D	L	Q	E	C
Translation of STRG.104	R	M	F	G	D	I	T	W	Y	G	K	T	L	G	E	V	S	R	S	*	F	V	C	P	S	R	*	F	W	W	Q	*
Translation of STRG.104	R	F	K	L	Q	D	G	S	F	Q	L	K	Y	L	D	D	E	E	E	W	V	M	L	V	T	D	S	D	L	Q	E	C
	R	f	k	l	q	d	g	s	f	q	l	k	y	l	d	d	e	e	e	W	v	m	l	v	t	d	S	d	l	q	e	C

		610									620										630										640
Translation of AT2G435(	L	E	I	L	H	G	M	G	K	H	S	V	K	F	L	V	R	D	L	S	A	P	L	G	S	S	G	G	S	N	G	Y
Translation of AT2G435(	L	E	I	L	H	G	M	G	K	H	S	V	K	F	L	V	R	D	L	S	A	P	L	G	S	S	G	G	S	N	G	Y
Translation of AT2G435(	L	E	I	L	H	G	M	G	K	H	S	V	K	F	L	V	R	D	L	S	A	P	L	G	S	S	G	G	S	N	G	Y
Translation of STRG.104	W	L	S	W	N	R	L	M	T	S	*	D	I	D	T	H	S	Y	V	F	P	V	K	E	C	C	L	F	L	*	I	L
Translation of STRG.104	L	E	I	L	H	G	M	G	K	H	S	V	K	F	L	V	R	D	L	S	A	P	L	G	S	S	G	G	S	N	G	Y
	l	e	i	l	h	g	m	g	k	h	S	v	k	f	l	v	r	d	l	s	a	p	l	g	s	s	g	g	s	N	g	y

										650										660										670		
Translation of AT2G435(	L	G	T	G	L	-	-	-	-	-	-	-	-	-	-	-	-	-	-	-	-	-	-	-	-	-	-	-	-	-	-	-
Translation of AT2G435(	L	G	T	G	L	-	-	-	-	-	-	-	-	-	-	-	-	-	-	-	-	-	-	-	-	-	-	-	-	-	-	-
Translation of AT2G435(	L	G	T	G	L	-	-	-	-	-	-	-	-	-	-	-	-	-	-	-	-	-	-	-	-	-	-	-	-	-	-	-
Translation of STRG.104	-	-	-	-	-	-	-	-	-	-	-	-	-	-	-	-	-	-	-	-	-	-	-	-	-	-	-	-	-	-	-	-
Translation of STRG.104	L	G	T	G	L	*	R	R	K	T	*	T	H	T	V	M	Y	S	Q	*	K	N	V	V	Y	F	S	R	Y	*	Y	A
	L	G	T	G	L	-	R	R	K	T	-	T	H	T	V	M	Y	S	Q	-	K	N	V	V	Y	F	S	R	Y	-	Y	A

								680										690										700		
Translation of AT2G435(	-	-	-	-	-	-	-	-	-	-	-	-	-	-	-	-	-	-	-	-	-	-	-	-	-	-	-	-	-	-
Translation of AT2G435(	-	-	-	-	-	-	-	-	-	-	-	-	-	-	-	-	-	-	-	-	-	-	-	-	-	-	-	-	-	-
Translation of AT2G435(	-	-	-	-	-	-	-	-	-	-	-	-	-	-	-	-	-	-	-	-	-	-	-	-	-	-	-	-	-	-
Translation of STRG.104	-	-	-	-	-	-	-	-	-	-	-	-	-	-	-	-	-	-	-	-	-	-	-	-	-	-	-	-	-	-
Translation of STRG.104	Y	K	*	A	*	R	R	K	T	I	L	V	*	W	S	S	A	E	N	V	Y	V	F	S	F	Y	M	N	Q	R
	Y	K	-	A	-	R	R	K	T	I	L	V	-	W	S	S	A	E	N	V	Y	V	F	S	F	Y	M	N	Q	R
