# Supplementary material for: Concordant Gene Expression and Alternative Splicing Regulation under Abiotic Stresses in Arabidopsis
Source: Genes (Basel). 2024 May 23;15(6):675. doi: 10.3390/genes15060675 (PMC11202685; doi:10.3390/genes15060675)
Supplement: Supplementary file 1 [file genes-15-00675-s001.zip › Figure S47.pdf]

Figure S47. Multiple sequence alignment at the amino acid level for annotated and new isoforms of *A. thaliana* locus XLOC_008527 generated under different multifactorial stress combinations where isoforms AT2G43500.11 and STRG.10463.9 showed expression pattern HL↑, isoform STRG.10463.14 showed expression pattern all stress combinations↑, while expression of isoforms AT2G43500.9 and AT2G43500.10 was arbitrary. H = heat stress, L = high light stress. The figure emphasizes Exons 5/6/7/8 alignment as referred to in Figure S20.

Alignment Name: Untitled3
Length: 670

Start5

	1									10										20										30		
Translation of AT2G435	Y	L	S	S	S	Q	R	D	A	L	A	E	I	Q	D	V	L	R	T	V	C	H	A	H	K	L	P	L	A	L	A	W
Translation of AT2G435	Y	L	S	S	S	Q	R	D	A	L	A	E	I	Q	D	V	L	R	T	V	C	H	A	H	K	L	P	L	A	L	A	W
Translation of AT2G435	Y	L	S	S	S	Q	R	D	A	L	A	E	I	Q	D	V	L	R	T	V	C	H	A	H	K	L	P	L	A	L	A	W
Translation of STRG.104	Y	L	S	S	S	Q	R	D	A	L	A	E	I	Q	D	V	L	R	T	V	C	H	A	H	K	L	P	L	A	L	A	W
Translation of STRG.104	Y	L	S	S	S	Q	R	D	A	L	A	E	I	Q	D	V	L	R	T	V	C	H	A	H	K	L	P	L	A	L	A	W
	Y	L	S	S	S	Q	R	D	A	L	A	E	I	Q	D	V	L	R	T	V	C	H	A	H	K	L	P	L	A	L	A	W

								40										50										60				
Translation of AT2G435	I	P	C	R	K	D	Q	S	I	R	V	S	G	Q	K	S	G	E	N	C	I	L	C	I	E	E	T	A	C	Y	V	N
Translation of AT2G435	I	P	C	R	K	D	Q	S	I	R	V	S	G	Q	K	S	G	E	N	C	I	L	C	I	E	E	T	A	C	Y	V	N
Translation of AT2G435	I	P	C	R	K	D	Q	S	I	R	V	S	G	Q	K	S	G	E	N	C	I	L	C	I	E	E	T	A	C	Y	V	N
Translation of STRG.104	I	P	C	R	K	D	Q	S	I	R	V	S	G	Q	K	S	G	E	N	C	I	L	C	I	E	E	T	A	C	Y	V	N
Translation of STRG.104	I	P	C	R	K	D	Q	S	I	R	V	S	G	Q	K	S	G	E	N	C	I	L	C	I	E	E	T	A	C	Y	V	N
	I	P	C	R	K	D	Q	S	I	R	V	S	G	Q	K	S	G	E	N	C	I	L	C	I	E	E	T	A	C	Y	V	N

						70										80										90						
Translation of AT2G435	D	M	E	M	E	G	F	V	H	A	C	L	E	H	C	L	R	E	K	E	G	I	V	G	K	A	F	I	S	N	Q	P
Translation of AT2G435	D	M	E	M	E	G	F	V	H	A	C	L	E	H	C	L	R	E	K	E	G	I	V	G	K	A	F	I	S	N	Q	P
Translation of AT2G435	D	M	E	M	E	G	F	V	H	A	C	L	E	H	C	L	R	E	K	E	G	I	V	G	K	A	F	I	S	N	Q	P
Translation of STRG.104	D	M	E	M	E	G	F	V	H	A	C	L	E	H	C	L	R	E	K	E	G	I	V	A	C	S	K	V	R	S	E	C
Translation of STRG.104	D	M	E	M	E	G	F	V	H	A	C	L	E	H	C	L	R	E	K	E	G	I	V	G	K	A	F	I	S	N	Q	P
	D	M	E	M	E	G	F	V	H	A	C	L	E	H	C	L	R	E	K	E	G	I	V	g	k	a	f	i	s	n	q	p

				100										110										120								
Translation of AT2G435	F	F	S	S	D	V	K	A	Y	D	I	S	E	Y	P	I	V	Q	H	A	R	K	Y	G	L	N	A	A	V	A	I	K
Translation of AT2G435	F	F	S	S	D	V	K	A	Y	D	I	S	E	Y	P	I	V	Q	H	A	R	K	Y	G	L	N	A	A	V	A	I	K
Translation of AT2G435	F	F	S	S	D	V	K	A	Y	D	I	S	E	Y	P	I	V	Q	H	A	R	K	Y	G	L	N	A	A	V	A	I	K
Translation of STRG.104	C	C	R	Y	K	T	E	E	H	L	H	W	*	R	*	L	H	T	*	T	V	L	A	C	K	Y	E	R	K	L	G	T
Translation of STRG.104	F	F	S	S	D	V	K	A	Y	D	I	S	E	Y	P	I	V	Q	H	A	R	K	Y	G	L	N	A	A	V	A	I	K
	f	f	s	s	d	v	k	a	y	d	i	s	E	y	P	i	v	q	H	a	r	k	y	g	l	n	a	a	v	a	i	k

		130										140										150										160
Translation of AT2G435	L	R	S	T	Y	T	G	E	D	D	Y	I	L	E	L	F	L	P	V	S	M	K	G	S	L	E	Q	Q	L	L	L	D
Translation of AT2G435	L	R	S	T	Y	T	G	E	D	D	Y	I	L	E	L	F	L	P	V	S	M	K	G	S	L	E	Q	Q	L	L	L	D
Translation of AT2G435	L	R	S	T	Y	T	G	E	D	D	Y	I	L	E	L	F	L	P	V	S	M	K	G	S	L	E	Q	Q	L	L	L	D
Translation of STRG.104	T	T	S	I	R	Q	P	F	G	Y	N	A	E	N	L	S	N	F	E	N	C	F	R	S	G	V	N	*	K	R	R	D
Translation of STRG.104	L	R	S	T	Y	T	G	E	D	D	Y	I	L	E	L	F	L	P	V	S	M	K	G	S	L	E	Q	Q	L	L	L	D
	l	r	S	t	y	t	g	e	d	d	y	i	l	e	L	f	l	p	v	s	m	k	g	S	l	e	q	Q	l	l	l	D

										170										180										190		
Translation of AT2G435	S	L	S	G	T	M	Q	R	I	C	R	T	L	R	T	V	S	E	V	G	S	T	K	K	E	G	T	K	P	G	F	R
Translation of AT2G435	S	L	S	G	T	M	Q	R	I	C	R	T	L	R	T	V	S	E	V	G	S	T	K	K	E	G	T	K	P	G	F	R
Translation of AT2G435	S	L	S	G	T	M	Q	R	I	C	R	T	L	R	T	V	S	E	V	G	S	T	K	K	E	G	T	K	P	G	F	R
Translation of STRG.104	*	T	W	I	S	E	*	*	Y	V	*	F	P	A	D	N	V	F	R	K	F	S	D	N	I	I	G	F	R	V	*	L
Translation of STRG.104	S	L	S	G	T	M	Q	R	I	C	R	T	L	R	T	V	S	E	V	G	S	T	K	K	E	G	T	K	P	G	F	R
	S	l	s	g	t	m	Q	R	i	c	R	t	l	r	t	v	s	e	v	g	s	t	k	k	e	g	t	k	p	g	F	r

								200										210										220				
Translation of AT2G435(	S	S	D	M	S	N	F	P	Q	T	T	S	S	E	N	F	Q	T	I	S	L	D	S	E	F	N	S	T	R	S	M	F
Translation of AT2G435(	S	S	D	M	S	N	F	P	Q	T	T	S	S	E	N	F	Q	T	I	S	L	D	S	E	F	N	S	T	R	S	M	F
Translation of AT2G435(	S	S	D	M	S	N	F	P	Q	T	T	S	S	E	N	F	Q	T	I	S	L	D	S	E	F	N	S	T	R	S	M	F
Translation of STRG.104	Y	*	K	H	V	F	G	Y	V	L	*	*	R	K	Q	Y	H	S	I	S	R	H	F	G	A	G	C	E	Q	S	K	N
Translation of STRG.104	S	S	D	M	S	N	F	P	Q	T	T	S	S	E	N	F	Q	T	I	S	L	D	S	E	F	N	S	T	R	S	M	F
	s	S	d	m	s	n	f	p	q	t	T	S	s	e	n	f	q	t	I	S	l	d	s	e	f	n	s	t	r	S	m	f

						230										240										250						
Translation of AT2G435(	S	G	M	S	S	D	K	E	N	S	I	T	V	S	Q	G	T	L	E	Q	D	V	S	K	A	R	T	P	E	K	K	K
Translation of AT2G435(	S	G	M	S	S	D	K	E	N	S	I	T	V	S	Q	G	T	L	E	Q	D	V	S	K	A	R	T	P	E	K	K	K
Translation of AT2G435(	S	G	M	S	S	D	K	E	N	S	I	T	V	S	Q	G	T	L	E	Q	D	V	S	K	A	R	T	P	E	K	K	K
Translation of STRG.104	T	R	E	E	E	K	H	Y	R	E	K	C	E	L	K	R	S	P	T	T	L	L	W	E	S	K	G	C	C	K	K	P
Translation of STRG.104	S	G	M	S	S	D	K	E	N	S	I	T	V	S	Q	G	T	L	E	Q	D	V	S	K	A	R	T	P	E	K	K	K
	s	g	m	s	s	d	k	e	n	s	i	t	v	s	q	g	t	l	e	q	d	v	s	k	a	r	t	p	e	K	K	k

Start domain RWP-RK
259

				260										270										280								
Translation of AT2G435(	S	T	T	E	K	N	V	S	L	S	A	L	Q	Q	H	F	S	G	S	L	K	D	A	A	K	S	L	G	V	C	P	T
Translation of AT2G435(	S	T	T	E	K	N	V	S	L	S	A	L	Q	Q	H	F	S	G	S	L	K	D	A	A	K	S	L	G	V	C	P	T
Translation of AT2G435(	S	T	T	E	K	N	V	S	L	S	A	L	Q	Q	H	F	S	G	S	L	K	D	A	A	K	S	L	G	V	C	P	T
Translation of STRG.104	W	C	L	S	N	Y	I	E	T	D	M	Q	A	T	W	D	H	E	V	A	I	S	*	D	*	Q	S	E	Q	V	T	K
Translation of STRG.104	S	T	T	E	K	N	V	S	L	S	A	L	Q	Q	H	F	S	G	S	L	K	D	A	A	K	S	L	G	V	C	P	T
	s	t	t	e	k	n	v	s	l	s	a	l	q	q	h	f	s	g	s	l	k	d	A	a	K	s	l	g	v	c	p	t

End domain RWP-RK
310

		290										300										310										320
Translation of AT2G435(	T	L	K	R	I	C	R	Q	H	G	I	M	R	W	P	S	R	K	I	N	K	V	N	R	S	L	R	K	I	Q	T	V
Translation of AT2G435(	T	L	K	R	I	C	R	Q	H	G	I	M	R	W	P	S	R	K	I	N	K	V	N	R	S	L	R	K	I	Q	T	V
Translation of AT2G435(	T	L	K	R	I	C	R	Q	H	G	I	M	R	W	P	S	R	K	I	N	K	V	N	R	S	L	R	K	I	Q	T	V
Translation of STRG.104	E	N	T	D	G	T	G	L	G	P	R	C	R	R	R	T	K	V	R	L	S	N	W	R	I	H	C	S	*	T	F	Y
Translation of STRG.104	T	L	K	R	I	C	R	Q	H	G	I	M	R	W	P	S	R	K	I	N	K	V	N	R	S	L	R	K	I	Q	T	V
	t	l	k	r	i	c	r	q	h	g	i	m	R	w	p	s	r	k	i	n	k	v	n	R	s	l	r	k	I	q	t	v

										330										340										350		
Translation of AT2G435(	L	D	S	V	Q	G	V	E	G	G	L	K	F	D	S	A	T	G	E	F	I	A	V	R	P	F	I	Q	E	I	D	T
Translation of AT2G435(	L	D	S	V	Q	G	V	E	G	G	L	K	F	D	S	A	T	G	E	F	I	A	V	R	P	F	I	Q	E	I	D	T
Translation of AT2G435(	L	D	S	V	Q	G	V	E	G	G	L	K	F	D	S	A	T	G	E	F	I	A	V	R	P	F	I	Q	E	I	D	T
Translation of STRG.104	S	R	N	*	Y	P	K	G	S	V	V	S	*	*	*	C	T	C	K	K	K	S	G	G	Y	A	*	R	Y	F	I	Q
Translation of STRG.104	L	D	S	V	Q	G	V	E	G	G	L	K	F	D	S	A	T	G	E	F	I	A	V	R	P	F	I	Q	E	I	D	T
	l	d	s	V	q	g	v	e	g	g	l	k	F	D	S	a	T	g	e	f	i	a	v	r	p	f	I	q	e	i	d	t

								360										370										380				
Translation of AT2G435(	Q	K	G	L	S	S	L	D	N	D	A	H	A	R	R	S	Q	E	D	M	P	D	D	T	S	F	K	L	Q	E	A	K
Translation of AT2G435(	Q	K	G	L	S	S	L	D	N	D	A	H	A	R	R	S	Q	E	D	M	P	D	D	T	S	F	K	L	Q	E	A	K
Translation of AT2G435(	Q	K	G	L	S	S	L	D	N	D	A	H	A	R	R	S	Q	E	D	M	P	D	D	T	S	F	K	L	Q	E	A	K
Translation of STRG.104	A	P	G	S	*	I	C	R	Q	C	H	*	V	R	G	G	Y	N	H	E	S	S	K	T	R	I	I	H	G	G	*	C
Translation of STRG.104	Q	K	G	L	S	S	L	D	N	D	A	H	A	R	R	S	Q	E	D	M	P	D	D	T	S	F	K	L	Q	E	A	K
	q	k	G	l	S	s	l	d	n	d	a	H	a	R	r	s	q	e	d	m	p	d	d	T	s	f	k	l	q	e	A	k

Alignment Name: Untitled3
Length: 670

						390										400										410						
Translation of AT2G4350	S	V	D	N	A	I	K	L	E	E	D	T	T	M	N	Q	A	R	P	G	S	F	M	E	V	N	A	S	G	Q	P	W
Translation of AT2G4350	S	V	D	N	A	I	K	L	E	E	D	T	T	M	N	Q	A	R	P	G	S	F	M	E	V	N	A	S	G	Q	P	W
Translation of AT2G4350	S	V	D	N	A	I	K	L	E	E	D	T	T	M	N	Q	A	R	P	G	S	F	M	E	V	N	A	S	G	Q	P	W
Translation of STRG.104	*	W	S	A	M	G	L	D	G	Q	R	V	W	L	E	W	Q	*	R	N	K	E	R	L	Q	L	K	L	C	G	N	F
Translation of STRG.104	S	V	D	N	A	I	K	L	E	E	D	T	T	M	N	Q	A	R	P	G	S	F	M	E	V	N	A	S	G	Q	P	W
	S	v	d	n	a	i	k	l	e	e	d	t	t	m	n	q	a	R	p	g	s	f	m	e	v	n	a	s	g	q	p	w

				420										430										440								
Translation of AT2G4350	A	W	M	A	K	E	S	G	L	N	G	S	E	G	I	K	S	V	C	N	L	S	S	V	E	I	S	D	G	M	D	P
Translation of AT2G4350	A	W	M	A	K	E	S	G	L	N	G	S	E	G	I	K	S	V	C	N	L	S	S	V	E	I	S	D	G	M	D	P
Translation of AT2G4350	A	W	M	A	K	E	S	G	L	N	G	S	E	G	I	K	S	V	C	N	L	S	S	V	E	I	S	D	G	M	D	P
Translation of STRG.104	R	W	N	G	S	N	N	P	M	Q	W	Q	Y	C	*	T	*	P	I	H	V	M	Q	H	I	R	F	I	K	W	L	R
Translation of STRG.104	A	W	M	A	K	E	S	G	L	N	G	S	E	G	I	K	S	V	C	N	L	S	S	V	E	I	S	D	G	M	D	P
	a	W	m	a	k	e	s	g	l	n	g	s	e	g	I	k	S	v	c	n	l	s	s	v	e	i	s	d	g	m	d	p

		450										460										470										480
Translation of AT2G4350	T	I	R	C	S	G	S	I	V	E	P	N	Q	S	M	S	C	S	I	S	D	S	S	N	G	S	G	A	V	L	R	G
Translation of AT2G4350	T	I	R	C	S	G	S	I	V	E	P	N	Q	S	M	S	C	S	I	S	D	S	S	N	G	S	G	A	V	L	R	G
Translation of AT2G4350	T	I	R	C	S	G	S	I	V	E	P	N	Q	S	M	S	C	S	I	S	D	S	S	N	G	S	G	A	V	L	R	G
Translation of STRG.104	R	S	S	A	W	K	L	I	Y	F	H	G	R	L	E	P	N	E	N	P	Q	Q	*	*	Q	R	E	W	I	N	N	A
Translation of STRG.104	T	I	R	C	S	G	S	I	V	E	P	N	Q	S	M	S	C	S	I	S	D	S	S	N	G	S	G	A	V	L	R	G
	t	i	r	c	s	g	s	I	v	e	p	n	q	s	m	s	c	s	i	s	d	s	S	N	g	s	g	a	v	l	r	g

Start domain PB1

										490						496				500										510		
Translation of AT2G4350	S	S	S	T	S	M	E	D	W	N	Q	M	R	T	H	N	S	N	S	S	E	S	G	S	T	T	L	I	V	K	A	S
Translation of AT2G4350	S	S	S	T	S	M	E	D	W	N	Q	M	R	T	H	N	S	N	S	S	E	S	G	S	T	T	L	I	V	K	A	S
Translation of AT2G4350	S	S	S	T	S	M	E	D	W	N	Q	M	R	T	H	N	S	N	S	S	E	S	G	S	T	T	L	I	V	K	A	S
Translation of STRG.104	D	R	K	G	Q	L	*	R	R	H	C	T	F	Q	V	R	A	I	S	W	V	S	S	A	L	Q	R	S	W	K	T	F
Translation of STRG.104	S	S	S	T	S	M	E	D	W	N	Q	M	R	T	H	N	S	N	S	S	E	S	G	S	T	T	L	I	V	K	A	S
	s	s	s	t	s	m	E	d	w	n	q	m	r	t	h	n	s	n	S	s	e	S	g	s	t	t	l	i	v	K	a	s

								520										530										540				
Translation of AT2G4350	Y	R	E	D	T	V	R	F	K	F	E	P	S	V	G	C	P	Q	L	Y	K	E	V	G	K	R	F	K	L	Q	D	G
Translation of AT2G4350	Y	R	E	D	T	V	R	F	K	F	E	P	S	V	G	C	P	Q	L	Y	K	E	V	G	K	R	F	K	L	Q	D	G
Translation of AT2G4350	Y	R	E	D	T	V	R	F	K	F	E	P	S	V	G	C	P	Q	L	Y	K	E	V	G	K	R	F	K	L	Q	D	G
Translation of STRG.104	*	T	A	G	R	V	V	S	A	E	V	L	G	*	*	R	R	M	G	D	A	G	Y	R	F	*	S	P	R	M	F	G
Translation of STRG.104	Y	R	E	D	T	V	R	F	K	F	E	P	S	V	G	C	P	Q	L	Y	K	E	V	G	K	R	F	K	L	Q	D	G
	Y	r	e	d	t	V	r	f	k	f	e	p	s	V	G	c	p	q	l	y	k	e	v	g	k	R	f	k	l	q	d	G

						550										560										570						
Translation of AT2G4350	S	F	Q	L	K	Y	L	D	D	E	E	E	W	V	M	L	V	T	D	S	D	L	Q	E	C	L	E	I	L	H	G	M
Translation of AT2G4350	S	F	Q	L	K	Y	L	D	D	E	E	E	W	V	M	L	V	T	D	S	D	L	Q	E	C	L	E	I	L	H	G	M
Translation of AT2G4350	S	F	Q	L	K	Y	L	D	D	E	E	E	W	V	M	L	V	T	D	S	D	L	Q	E	C	L	E	I	L	H	G	M
Translation of STRG.104	D	I	T	W	Y	G	K	T	L	G	E	V	S	R	S	*	F	V	C	P	S	R	*	F	W	W	Q	*	W	L	S	W
Translation of STRG.104	S	F	Q	L	K	Y	L	D	D	E	E	E	W	V	M	L	V	T	D	S	D	L	Q	E	C	L	E	I	L	H	G	M
	s	f	q	l	k	y	l	d	d	e	E	e	w	v	m	L	v	t	d	s	d	l	Q	e	c	l	e	I	l	h	g	m

Alignment Name: Untitled3
Length: 670

End domain PB1 577 End5 606

				580										590										600								
Translation of AT2G435(	G	K	H	S	V	K	F	L	V	R	D	L	S	A	P	L	G	S	S	G	G	S	N	G	Y	L	G	T	G	L	*	-
Translation of AT2G435(	G	K	H	S	V	K	F	L	V	R	D	L	S	A	P	L	G	S	S	G	G	S	N	G	Y	L	G	T	G	L	*	-
Translation of AT2G435(	G	K	H	S	V	K	F	L	V	R	D	L	S	A	P	L	G	S	S	G	G	S	N	G	Y	L	G	T	G	L	*	-
Translation of STRG.104	N	R	L	M	T	S	*	D	I	D	T	H	S	Y	V	F	P	V	K	E	C	C	L	F	L	*	I	L	V	C	L	*
Translation of STRG.104	G	K	H	S	V	K	F	L	V	R	D	L	S	A	P	L	G	S	S	G	G	S	N	G	Y	L	G	T	G	L	*	R
	g	k	h	s	v	k	F	l	v	r	d	l	S	a	p	l	g	s	s	g	g	s	n	g	y	L	g	t	g	l	L	R

		610										620										630										640
Translation of AT2G435(	-	-	-	-	-	-	-	-	-	-	-	-	-	-	-	-	-	-	-	-	-	-	-	-	-	-	-	-	-	-	-	-
Translation of AT2G435(	-	-	-	-	-	-	-	-	-	-	-	-	-	-	-	-	-	-	-	-	-	-	-	-	-	-	-	-	-	-	-	-
Translation of AT2G435(	-	-	-	-	-	-	-	-	-	-	-	-	-	-	-	-	-	-	-	-	-	-	-	-	-	-	-	-	-	-	-	-
Translation of STRG.104	I	G	M	K	E	K	D	N	F	G	I	V	E	F	S	R	K	C	I	C	F	F	V	L	Y	E	S	E	N	K	S	W
Translation of STRG.104	R	K	T	*	T	H	T	V	M	Y	S	Q	*	K	N	V	V	Y	F	S	R	Y	*	Y	A	Y	K	*	A	*	R	R
	X	X	X	K	X	X	X	X	X	X	X	X	E	X	X	X	X	X	X	X	X	X	V	X	X	X	X	E	X	K	X	X

										650										660										670
Translation of AT2G435(	-	-	-	-	-	-	-	-	-	-	-	-	-	-	-	-	-	-	-	-	-	-	-	-	-	-	-	-	-	-
Translation of AT2G435(	-	-	-	-	-	-	-	-	-	-	-	-	-	-	-	-	-	-	-	-	-	-	-	-	-	-	-	-	-	-
Translation of AT2G435(	-	-	-	-	-	-	-	-	-	-	-	-	-	-	-	-	-	-	-	-	-	-	-	-	-	-	-	-	-	-
Translation of STRG.104	M	L	Y	L	-	-	-	-	-	-	-	-	-	-	-	-	-	-	-	-	-	-	-	-	-	-	-	-	-	-
Translation of STRG.104	K	T	I	L	V	*	W	S	S	A	E	N	V	Y	V	F	S	F	Y	M	N	Q	R	I	K	V	G	C	Y	I
	X	X	X	L	V	-	W	S	S	A	E	N	V	Y	V	F	S	F	Y	M	N	Q	R	I	K	V	G	C	Y	I
